# Sustained population decline of rodents is linked to accelerated climate warming and human disturbance

**DOI:** 10.1186/s12862-022-02056-z

**Published:** 2022-08-22

**Authors:** Xinru Wan, Chuan Yan, Zhenyu Wang, Zhibin Zhang

**Affiliations:** 1 State Key Laboratory of Integrated Management of Pest Insects and Rodents, Institute of Zoology, Chinese Academy of Sciences, Beijing, 100101 China; 2 College of Life Sciences, Jiangxi Normal University, Nanchang, 330022 China; 3 CAS Centre for Excellence in Biotic Interactions, University of Chinese Academy of Sciences, Beijing, 100049 China

**Keywords:** Rodent, Climate change, Human disturbance, Population dynamics, Ecosystem transition or dysfunction

## Abstract

**Background:**

During the past three decades, sustained population decline or disappearance of cycles in small rodents have been observed. Both anthropogenic disturbance and climate warming are likely to be potential drivers of population decline, but quantitative analysis on their distinct effects is still lacking.

**Results:**

Using time series monitoring of 115 populations (80 populations from 18 known rodent species, 35 mixed populations from unknown species) from 1980 in China (spanning 20–33 yrs), we analyzed association of human disturbances and climate warming with population dynamics of these rodent species. We found 54 of 115 populations showed a decreasing trend since 1980, and 16 of 115 showed an increasing trend. Human disturbances and climate warming showed significant positive associations with the population declines of most rodent species, and the population declines were more pronounced in habitats with more intensified human disturbance such as cities and farmlands or in high-latitude regions which experienced more increase of temperature.

**Conclusions:**

Our results indicate that the large-scale sustained population decline of small mammals in various ecosystems driven by the rapid increase of both climate warming and human disturbance is likely a signal of ecosystem dysfunction or transition. There is an urgent need to assess the risks of accelerated climate warming and human disturbance imposes on our ecosystems.

**Supplementary Information:**

The online version contains supplementary material available at 10.1186/s12862-022-02056-z.

## Background

Global average temperature of the earth has increased approximately 1.09 °C in the past 150 years, which has accelerated the melt of glaciers and ice since the 1980s [1, 2]. At the same time, human disturbance such as logging, grazing, and farming has caused extensive damage to our ecosystems. Under the impacts of accelerated global change, 16–33% vertebrates are globally threatened or endangered [3]. In response to climate warming, the ranges of many terrestrial animals shifted toward higher latitude or elevation area [4, 5]. Climate warming and human disturbance were thought to be the causative drivers of the large-scale population decline of endangered species [6].

Small rodents make up approx. 42% of mammalian species. Many rodent species are pests to human society because they cause damage to our agriculture, forestry, grasslands, and transmit many diseases to people [7]. However, they also play an important role in maintaining ecosystem function and services [8]. As keystone species, rodents are preyed upon by many predators [9], and play important roles in plant seedling regeneration as seed dispersers [10]. They also response quickly to global change due to their short lifespan and high reproductive capacity, and are often used as an important indicator of ecosystem function considering their large abundance, high diversity and wide distribution. Sustained decline of populations and collapse of communities of small rodents may be an early signal of ecosystem dysfunction or transition.

Population abundance of many small rodents species oscillate greatly from year to year under influences by both extrinsic and intrinsic factors [11, 12]. Recent studies found that the population cycles of voles and lemmings in Europe are collapsing or disappearing [13–17], and population of common hamsters has declined 74% since 1970s in Europe [18], likely caused by climate warming or human disturbance. However, a few other studies argue against climate warming as the cause for the loss of cycles [14, 19]. Thus, it is questionable if the observed population decline is a global phenomenon or a regional one. Because climate warming is closely associated with increased human disturbances during past decades, it is also necessary to distinguish the distinctive effects of human disturbance and climate warming in causing population declines of small rodents.

The purpose of this study aims to evaluate associations of climate warming and human impacts with population dynamics of small rodents in China by using 115 time series of historical data (spanning 20–33 yrs) since 1980 based on the literature. Because rodents are important pests in many ecosystems of China, they are well monitored or studied by governmental agencies or research institutions. The time series of our study covers various ecosystems and climate zones across China, which experienced different pressures of climate warming and human impacts, thus, provide an opportunity to disentangle their distinct effects on population dynamics of small rodents.

## Results

### Changing trend of rodent abundance

**Fig. 1:**
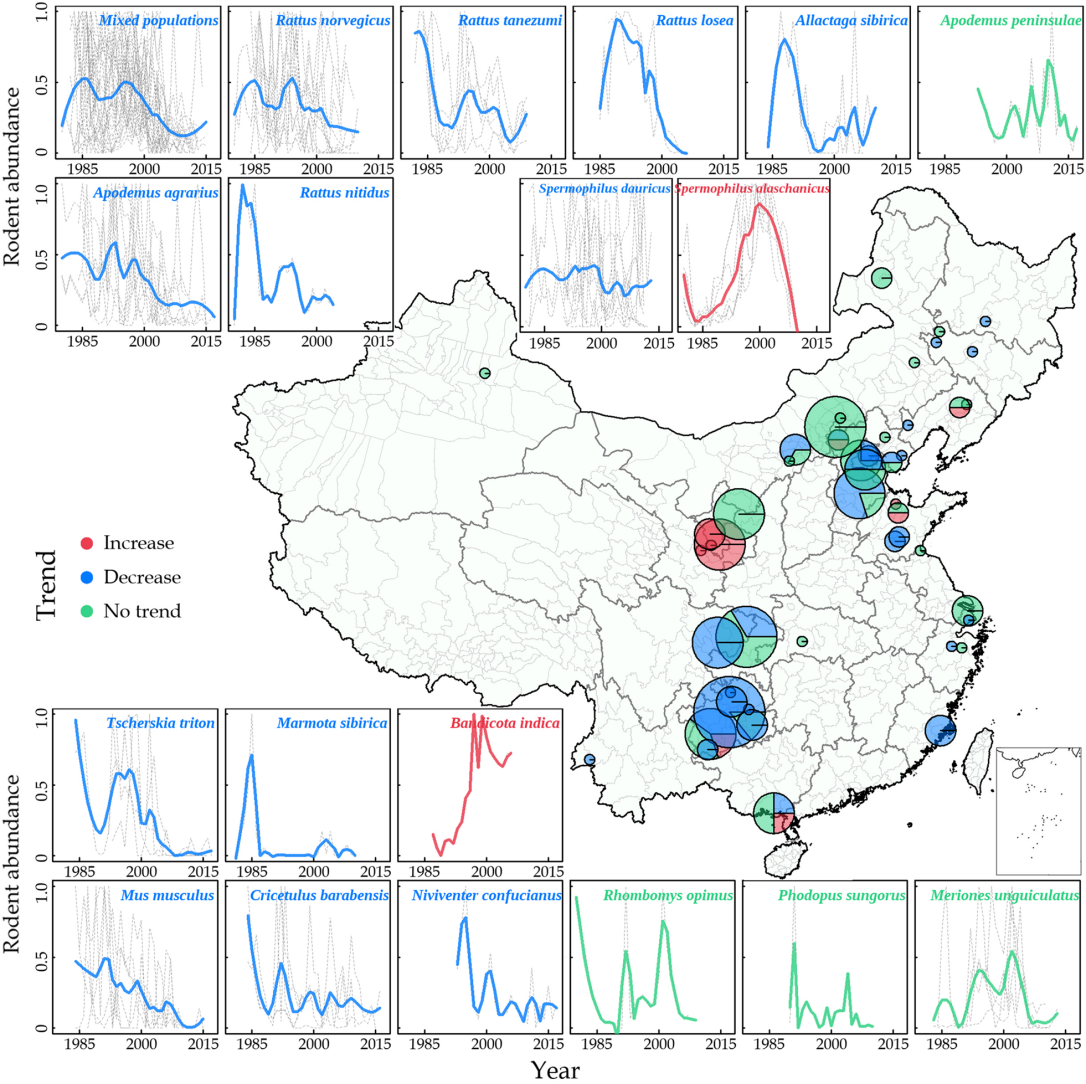
Spatial distribution of 115 time series (80 populations from 18 known rodent species, 35 mixed populations from unknown species) of rodents in China and the changing trend of 18 rodent species. The pies in the map show the sites of the 115 time series, the size of the pies indicate the number of time series, and the size of slices of each pie indicate the proportion of increase (red), decrease (blue), and no trend (green) time series. The surrounding panels around the map show the changing trend of normalized rodent abundance of the 18 rodent species and the mixed populations. The color of symbols or lines indicates the changing trend of the time series: increase (red), decrease (blue), and no trend (green). The dashed gray lines of each panel indicate the rodent abundance of each species from multiple sites

In this study, 115 time series (80 populations from 18 known rodent species, 35 mixed populations from unknown species) with durations ranging from 20 to 33 years (from 2737 datum) since 1980 were reconstructed, covering a broad range and various ecosystems of China (Fig. 1, Additional file 1: Figs. S1, S2, Table S1).

**Fig. 2:**
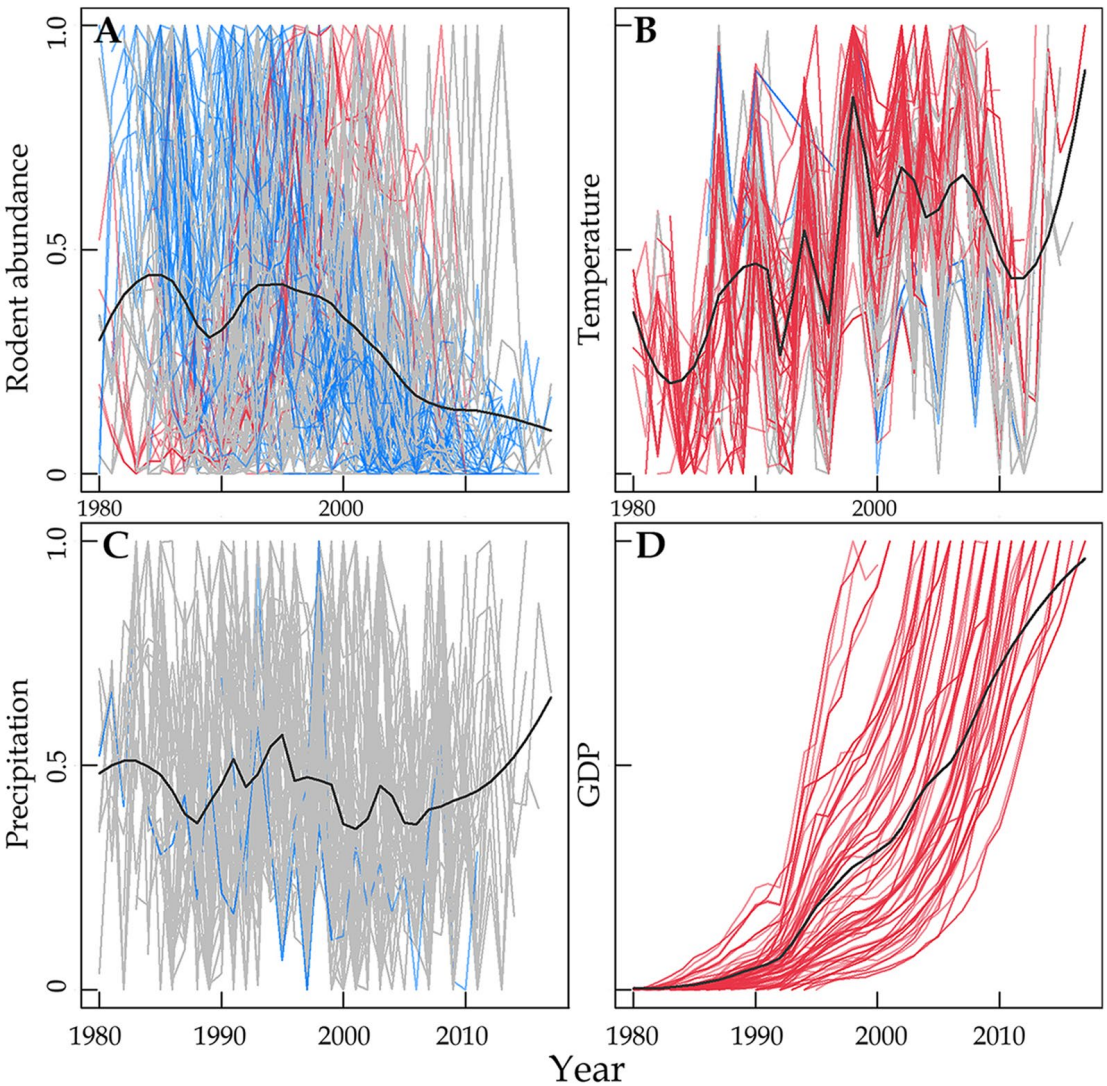
Normalized time series of rodent abundance, temperature, precipitation, and GDP of each location. **A** Annual rodent abundance of 115 populations (the same below); **B** annual mean temperature; **C** annual precipitation; **D** prefectural annual gross domestic product (GDP). Line color indicates the changing trend of rodent abundance and environmental variables for a given population (red: increase; blue: decrease; grey: no trend). Black solid line indicates loess regression with span = 0.25

**Fig. 3:**
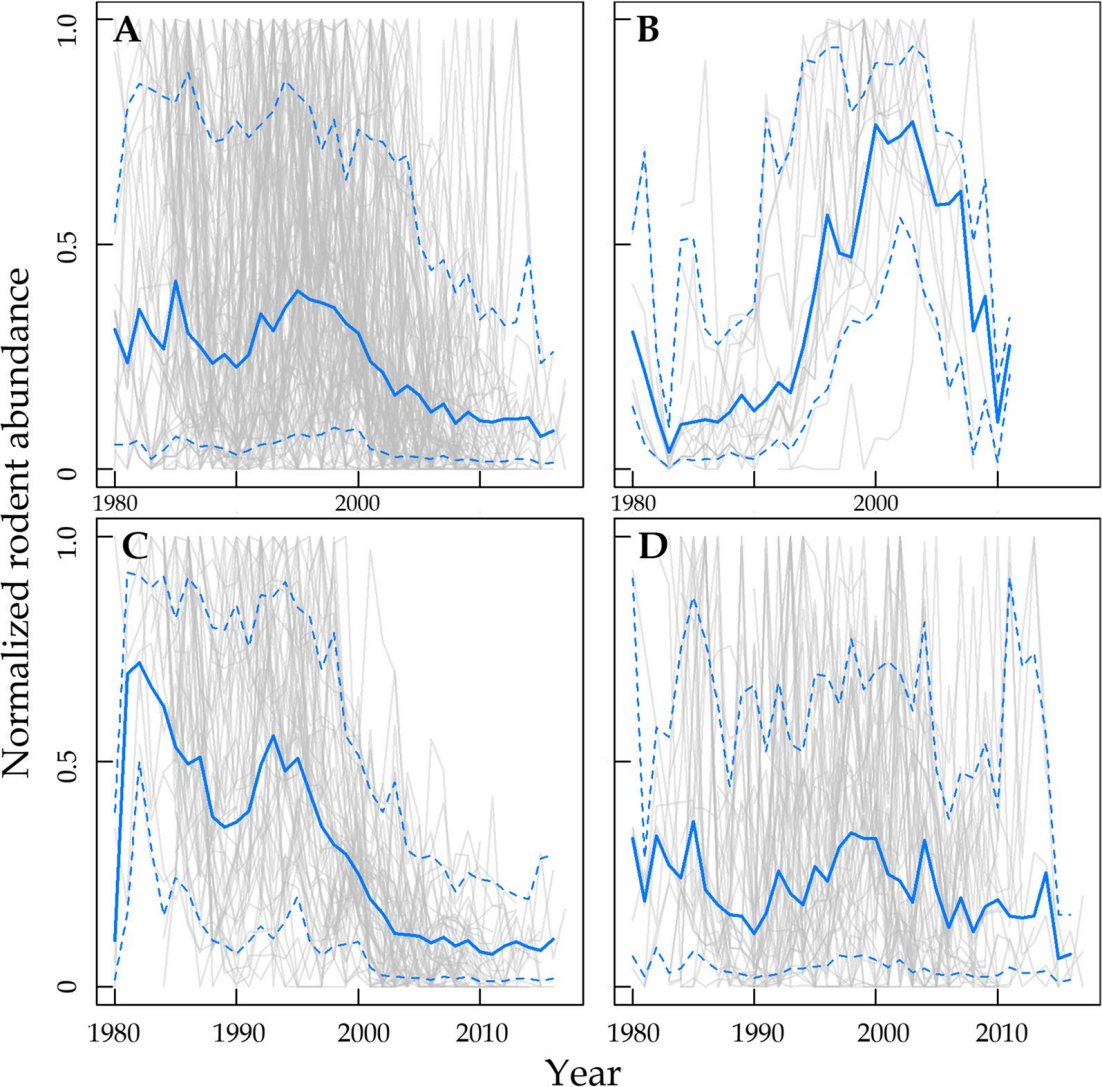
Changing trend of rodent abundance (normalized) of 115 time series of rodents in China.** A** Pooled populations, **B** increase populations, **C** decrease populations, **D** no trend populations. Grey solid lines represent normalized rodent population dynamics; Blue solid line represents 50% moving kernel-smoothed quantile, upper and down blue dashed line represent 90% and 10% quantiles. Pooled population means all populations including 35 mixed populations and 80 populations of 18 single species. Wavelet analysis for the population cyclicity of pooled populations (panel A) was shown in Additional file 1: Fig. S3

Nearly half of the time series (54 of 115) showed decreasing trend since 1980, but only 13.9% (16 of 115) showed increasing trend, while others did not show any significant trend (Fig. 2A, Fig. 3, Additional file 1: Table S2, S3). For the 16 populations showing increasing trend, they also showed obvious population decline since 2000 (Fig. 3B). Most of the annual temperatures of the study sites showed increasing trend (Fig. 2B), and all GDP showed accelerated increasing trend (Fig. 2D), while most precipitation time series showed no trend, only a few showed decreasing trend (Fig. 2C).

The pooled time series of rodent abundance showed a steady decline, with two obvious population peaks at 1988 and 1996 and rapid population decline since 1996 (Fig. 3A). Increasing populations show increase since 1980’s but decline since 2000 (Fig. 3B); decreasing populations showed a consistent decline since 1980 (Fig. 3C). The pooled rodent abundance showed a significant periodicity at 2–4 years, nonsignificant at 4–12 years during 1980–1987, but the amplitude of population cyclicity showed decrease for periodicity at 2–4 years after 1997 and at 4–12 years after 1990 (Additional file 1: Fig. S3).

Correlation analysis indicated that GDP showed significant and negative correlations with population abundance of pooled population, mixed populations and 10 of the known species, but positive correlation with two species (*Bandicota indica* and *Spermophilus alashanicus*). Temperature showed significant and negative correlations with pooled population and 6 species, but positive correlation with one species (*Spermophilus alashanicus*). Precipitation showed a significant and positive correlation with population abundance of the pooled populations and one species (*Rattus tanezumi*) (Additional file 1: Fig. S2, Table S4).

Using GAM analysis, city/farmland habitat and latitude showed significant and negative effects on the changing trend of rodent abundance (*p* < 0.001 for city/farmland habitat; *p* < 0.01 for latitude), whereas body mass did not show significant effect on the changing trend (*p* > 0.05).

### Association of human disturbance and climate with rodent abundance

Using spatial–temporal general additive model (GAM) analysis, the association of climate change and human disturbance with rodent abundance were analyzed for each species (Table 1). We found the rodent abundance of last year showed significant and positive associations with rodent abundance of the pooled populations, mixed populations and 18 known species (except for *Rhombomys opimus*, *Niviventer confucianus*, *Apodemus peninsulae*, *Rattus nitidus*, and *Phodopus sungorus*). GDP showed significant and negative associations with rodent abundance of the pooled population and 7 known species including *Rattus norvegicus*, *Apodemus agrarius*, *Mus musculus*, *Meriones unguiculatus*, *Rattus losea*, *Rattus nitidus*, and *Tscherskia triton* (Table 1).

**Table 1:** Associations of rodent abundance with the intrinsic factor (rodent abundance of last year), human disturbance factor (annual GDP), climate factors (annual mean temperature, annual precipitation), habitat, and spatial autocorrelation based on analyses using Eq. (1)

Family	Species/groups	Number of time series	Rodent abundance of last year	Annual GDP	Annual mean temperature	Annual Precipitation	Habitat	Spatial auto correlation
	Pooled populations	115	0.62***	**−** 0.07***	**−** **0.03**^**#**^	0.03*^#^	+	+
	Mixed populations	35	0.69***		**−** 0.07**^#^			
Muridae	*Rattus norvegicus*	13	0.48***	**−** 0.12*	**−** 0.1*		+	
	*Apodemus agrarius*	12	0.48***	**−** 0.15**	**−** 0.14**^#^	**0.09**^**#**^	+	
	*Mus musculus*	6	0.25**	**−** 0.25**		0.14	+	+
	*Rattus tanezumi*	6	0.64***		**−** 0.11			
	*Meriones unguiculatus*	5	0.54***	**−** 0.18*			NA	NA
	*Rattus losea*	2	0.43**	**−** 0.61***			NA	NA
	*Rhombomys opimus*	1					NA	NA
	*Niviventer confucianus*	1		**−** 0.25			NA	NA
	*Apodemus peninsulae*	1			**−** 0.39*^#^		NA	NA
	*Rattus nitidus*	1	0.35	**−** 0.4*			NA	NA
	*Bandicota indica*	1	0.82***				NA	NA
Sciuridae	*Spermophilus dauricus*	10	0.55***			**0.1**^**#**^		
	*Spermophilus alashanicus*	8	0.82***		0.16**^#^		NA	NA
	*Marmota sibirica*	1	0.36*		**−** **0.27**^**#**^		NA	NA
Cricetidae	*Cricetulus barabensis*	5	0.27***			0.19**^#^	+	
	*Tscherskia triton*	4	0.5***	**−** 0.28**			NA	NA
	*Phodopus sungorus*	1					NA	NA
Dipodidae	*Allactaga sibirica*	2	0.49***			**−** 0.24	NA	NA

Bold values indicated the coefficients represent the significant effects (*p* < 0.05). + denotes the significant effects of habitat and spatial autocorrelation (*p < 0.05, **p < 0.01, ***p < 0.001). ^#^ denotes 1-yr delayed effected of climate factors. NA denotes not analyzed due to small number of locations

Temperature showed significant and negative associations with rodent abundance of the pooled population, mixed populations and 3 known rodent species including *Rattus norvegicus*, *Apodemus agrarius*, and *Apodemus peninsulae* in the current year or with one-year time lag; only one significant and positive association was found for one species (*Spermophilus alashanicus*) with 1-yr time lag (Table 1). Precipitation showed significant and positive associations with rodent abundance of the pooled populations and *Cricetulus barabensis* in the current year or with one-year time lag (Table 1).

Habitat type showed significant association with rodent abundance of the pooled populations and 4 known species including *Rattus norvegicus*, *Apodemus agrarius*, *Mus musculus*, and *Cricetulus barabensis*, and spatial auto correlation showed significant association with rodent abundance of the pooled populations and *Mus musculus* (Table 1).

Using GAM analysis, the effects of body mass, habitat, and latitude on the association coefficients between population abundance and variables of climate change and human disturbance were analyzed (Additional file 1: Table S5). We found 4 habitat types (grassland—farmland mosaic, city, farmland, and forest) showed negative effects on the associations coefficients between GDP on rodent abundance, with the order of city > farmland > grassland—farmland mosaic = forest (Additional file 1: Table S5). Two habitat types (city and farmland habitat) showed negative effects on the association coefficients between temperature and rodent abundance (Additional file 1: Table S5).

## Discussion

By analyzing multiple long-term time series of rodent abundances across different ecosystems and climate zones in China, we demonstrated that about half of the rodent populations showed sustained decline during the past three decades, and both climate warming and human disturbance were linked to the population decline of rodents in China; such negative association with population abundance of rodents were more pronounced in habitats with intensified human disturbance or in high-latitude regions which experienced more climate warming. Besides, we found most populations showed significant density-dependency effects, and precipitation showed positive effects on rodent abundance of many species. Our results suggest that the observed large-scale population decline of small mammals is likely a global phenomenon driven by intensified human disturbance and climate warming, and rodent population in intensified human disturbance habitats such as city and farmland were more vulnerable. The observed population decline is likely a signal that our ecosystems is heavily disrupted by both human disturbance and climate warming. Under the on-going accelerated global change, we predict that the previously predominant rodent species will become less abundant, and will be replaced with other less abundant species or invaded species, resulting in ecosystem transitions or dysfunction.

### Effect of human disturbances

Large mammals are facing population decline or local extinction under intensified human disturbances, with 322 terrestrial vertebrate species going extinct during past 500 years [3]. In contrast to large mammals, small rodents are much more resistant to human disturbances due to their large populations, high reproductive rates, high dispersal rate but small body size [20].

Previous studies indicate that moderate human disturbance could benefit rodent species by providing favorable conditions to trigger population increases. For example, livestock grazing significantly increases rodent abundance and their damage to the arid grasslands of Inner Mongolia [21] and meadow ecosystems in the Qinghai-Tibetan plateau [22] in China; this is because grazing reduces the grass height, which benefits small rodents in the grassland that favor open habitats for social communication or more alertness to predation.

However, over-disturbance by human activities may reverse its positive effects on rodents. Zhang et al*.* found the population increase rate of Brandt’ voles had a dome-shaped response: too low or high vegetation cover reduced the increase rate, suggesting over-grazing would impose negative impact on small rodents in grasslands [23]. By manipulation of grazing in large enclosures in Inner Mongolia, Li et al*.* demonstrated that successive grazing significantly reduced population abundance of the voles by decreasing the food quality and quantity, which explains why the voles became rare in most part of Inner Mongolia during the past two decades [24]. In Europe, the population declines of hamsters was found to be linked to intensified and monoculture farming [13–18].

In this study, we found GDP, the only available indicator of human disturbances in the study sites, showed significant and negative associations with pooled populations and populations of nearly half (7 out of 18) of the rodent species, indicating that the intensified human disturbance during the past three decades reversed its positive effect on rodents under moderate human interferences. Meanwhile, negative effects of temperature and GDP on rodent populations were more pronounced in habitats with more intensified human disturbances such as city or farmland, further supporting the observation that accelerated human disturbance showed negative effects on population of rodents. Of the species showing sustained population decline and were negatively affected by GDP, they also mainly inhabited in the heavily disturbed areas nearby humans and consume anthropogenic foods, including *Rattus norvegicus*, *Apodemus agrarius*, *Mus musculus*, *Rattus losea*, *Rattus nitidus* and *Tscherskia triton* living in farmlands and villages. *Niviventer confucianus* mainly dominated in deforested areas, while *Meriones unguiculatus* dominated in heavily grazed areas (e.g. converted into semi-desert habitats) in grasslands. Human disturbance showed non-significant effects on the other species mostly live in habitats where human disturbance is less extensive.

It is notable that GDP may not represent the effect of other human disturbance, such as rodent control, or land use. Rodents have been extensively controlled by using various methods such as rodenticides, traps, or habitat management, which may contribute to the population decline of some rodent species, and in some cases, they may outweigh the positive effects of climate [25]. In city or village area, with the development of economy as represented by GDP, the living condition has been well improved due to increased sanitation measures, extensive baiting program, and adaptation of concrete building, which significantly suppressed population density of rodents.

### Effect of temperature

It is generally thought that a warmer climate would benefit rodent populations by increasing plant growth (for their food resources) or winter survival of rodents or early breeding, but shorter winters may dampen lemmings and vole’s populations cycles in Arctic area [26–28]. Pucek et al*.* found high summer temperature of the previous year triggered seed masting of trees, and then population outbreaks of rodents in the following year [29], consistent with those findings, the positive association between climate warming and population abundance was also found for *Spermophilus alashanicus* likely due to increase of vegetation under climate warming [30, 31].

In contrast with results of previous studies, our study demonstrated that temperature showed significant and negative associations with the pooled population, mixed population and populations of 4 known rodent species, including *Rattus norvegicus*, *Apodemus agrarius*, *Apodemus peninsulae*, and *Marmota sibirica*, which are from diversified habitats including farmland (*A. agrarius, R. tanezumi*), human settlement (*R. norvegicus*), forest (*A. peninsulae, agrarius*), grassland (*M. sibirica*). Recent studies also found temperature showed a negative association with marmots in Subei County of northern China with 0–3 years lag [32]. Their sustained population decline during past three decades was likely caused by sustained climate warming which is far from it optimum point for rodents. Climate warming approaching to optimum temperature would promote rodent abundance, however extreme warming would reduce rodent abundance. Using millennia data, Johnson, et al*.* found climate warming depressed population cycling of larch bud moth in Europe by altering their temperature from its optimal point. A few recent studies demonstrated that high temperatures could prohibit the recruitment and pregnancy rate of rodents in Inner Mongolia [35], reduce maturation rates and reproductive copulation attempts of rodents in South Africa [36], or alter the spatial distribution [37]. Temperature showed a dome-shaped effects on *Apodemus agrarius* with the optimum temperature of 21 °C in northern China [33], 22.1–25 °C in southern China [34]. Furthermore, we found more population showed decline in high-latitude regions of China. This is likely because the high-latitude region experienced more increase of temperature during past decades [38], which caused extra negative effects on population of rodents in these regions. In Europe, the population decline of lemmings was suggested to be affected by climate warming [17] which prevented forage under the snow coverage due to the hard ice layers caused by snow melting. A recent study revealed that climate warming was associated with the pole-ward contraction of southern boundary of Brandt’s voles, particularly when maximum air temperature is closely to the thermal neutral zone [41].

Sustained climate warming may disrupt the life-history of rodents if activity cycles do not match well with plant phenology. For example, Inouye et al*.* found under continued warming, the hibernating yellow-bellied marmots (*Marmota flaviventris*) appeared above ground 38 days earlier than observed 23 years ago [42]. Climate warming could pose stronger effects on intensified human influence habitats (such as cities and farmland), accelerate the decline of rodent population, because the habitat fragmentation caused by human activates could prevent the movement of animals under climate change [43].

Apart from direct effects, temperatures may have indirect effects which are opposite to that of direct effects. For example, Tian et al*.* found droughts boosted outbreaks of locusts in ancient China during AD 1–1900, but climate warming depressed its outbreaks by reducing drought frequency [44]. Jiang et al*.* found that temperature not only had a direct positive effect on rodent abundance in Inner Mongolia, but also had a negative indirect effect via altering the vegetation coverage on some rodent species, because many rodents in the grasslands do not like high or dense grass because they are less alerted to potential attacks by predators and their social communications is more difficult [45].

### Effects of precipitation and density-dependency

Precipitation is generally thought to promote population growth of small rodents by increasing food resources or shelters [46, 47]. Previous studies found that precipitation was highly associated with rodent populations in both desert and tropical ecosystems [48]. Precipitation could facilitate rodent abundance of *Peromyscus leucopus* in North America, and for rodents in Arizona, via the increase of food resources both as vegetation biomass and seed production [49]. The positive association between precipitation and rodent abundance was found in southern and eastern Australia [50], western South America [51], Africa [52] and Inner Mongolia, China [53]. In this study, we found precipitation showed positive (current or with 1-yr time lag) associations with pooled population and four rodent species, including *Apodemus agrarius*, *Mus musculus*, *Spermophilus dauricus*, and *Cricetulus barabensis*, which is consistent with some previous studies, For example, cold and high precipitation increased the capture rate of *Apodemus flavicollis* in Poland [54], and high precipitation promoted population abundance of *Mus musculus* and *Spermophilus dauricus* by increased vegetation coverage and food production in Australia and northern China [55, 56].

Density-dependency is well recognized as a major regulator of population dynamics [57, 58]. Density dependence effects were reported in populations of hares and lynx in the boreal forests of North America and Scandinavia [6, 59], multimammate rat in fallow land of Tanzania [52], ungulates in The Rocky Mountains of the USA [60], and Brandt’s voles in the grasslands of China [61]. In this study, we found most rodent species showed positive density-dependency effects from their previous abundance to the population abundance in the following year. For those showing non-significant density-dependency effects (i.e. *Rhombomys opimus*, *Niviventer confucianus*, *Apodemus peninsulae*, and *Phodopus sungorus*), there is only one long-term time series available for those species.

## Conclusions and implications

Rodents have been listed as major pests to humans. The good news is that the observed sustained population declines for 54 out of 115 rodent populations due to climate warming or human disturbance could benefit pest management programs in some rodent-infested regions or ecosystems. It is predicted that with on-going accelerated climate warming and human disturbance, the rodent damage problems will be alleviated in the observed regions. However, climate warming would increase rodent problems at higher elevations or higher latitudes due to their invasions into these regions. Because climate warming would bring more precipitation in arid regions of western China [38], it may also increase rodent abundance and lead to a higher prevalence of rodent-borne diseases such as Hemorrhagic Fever with Renal Syndrome (HFRS) in these regions [62], climate proxies could be useful for the forecast and control of rodent-associated zoonosis [63].

The bad news is that the large-scale of population declines of rodents may impose risk to natural ecosystems. Under the successive climate warming in the past decades, the period from 1983 to 2012 (almost overlap with the study period) was likely the warmest 30-year period of the last 1400 years in the Northern Hemisphere [64]. A previous study indicated that global warming negatively affected abundance of lynx (*Lynx canadensis*) population and damped the 10-year cycles [6]. The natural cycles of rodents are 3–5 or 9–11 years, the observed sustained population declines for over 2 or 3 decades suggest that the natural cycles of rodents are disrupted by the accelerated climate warming and intensified human disturbance. Rodents are relatively more resistant to environmental disturbance and make up a large proportion of mammalian fauna, thus, the collapse of the rodent community may be a warning signal of ecosystem transition or dysfunction. Rodents are important consumers, prey of many predator species, and seed dispersers for trees [65]. They play an important role in maintaining ecosystem function as keystone species in many ecosystems [66]. Collapse of rodent communities will significantly disrupt ecosystem function and ecosystem services. Therefore, it is necessary to conserve some beneficial or rare species while managing the rodent pest species.

## Materials and methods

### Rodent abundance data

We obtained rodent abundance data from the published scientific literature and survey reports since 1963, covering 20 provinces in China. Each datum consisted of following information: reference, species name, location (province, prefecture, and county), habitat type, abundance type (population density, capture rate, etc.), year, and abundance value. We only used data since 1980 to test the effects of climate warming and human disturbance (as represented by Gross Domestic Production, GDP) on change of population abundance of small rodents because successive rodent abundance data are rare before 1980. After 1980, the earth experienced rapid climate warming, and China experienced rapid economic growth. Analysis focusing on this period would help to identify their distinct effects on rodents.

Rodent abundance data was verified by using conservative data, for example, we removed data with uncertain species (i.e. unclear species names), time resolution larger than one year, location resolution larger than prefecture level. To detect the trend and changes of rodent population abundance at each location, we only used time series of commonly-seen (presence over half years of over the study period) species with the survey period equal or larger than 20 years and the data was surveyed by the same research team. There are four types of rodent abundance in the literature and survey reports: trap success (i.e. capture rate %, making up 79% of the data), population density (numbers of rodents per hectare, making up 20% of the data), and relative population density (active holes per hectare, making up 1% of the data). There are six habitat types: forest, grassland, farmland, grassland—farmland mosaic, city, and mixed habitats. We assigned latitude and longitude coordinates of geographical locations to the capital location of the prefecture using Amap (lbs.amap.com). Population abundance of multiple locations in a given county was calculated by the average of all locations in the same habitat. Yearly population abundance was calculated by the average of all monthly or seasonal data for a given year. After data verification, a total of 115 time series (80 populations from 18 known rodent species, 35 mixed populations from unknown species) was used in this study for further analysis.

### Anthropogenic and climate proxy data

China experienced rapid economic growth as reflected by the gross domestic product (GDP) since the economic reform and opening up in 1978, accompanying with a high-speed of industrialization and urbanization. GDP is closely correlated with urban land expansion [67]. Therefore, GDP in each prefecture was used to represent human impact during 1980–2016. The GDP data was collected from national, provincial, and local statistical yearbooks or work reports compiled by statistical bureau or government. A small proportion of GDP data (4.5%) data was not available in the early 1980s for a few prefectures (making up 17.9%). We calculated these GDP based on the GDP of the next year and the average of the provincial GDP growth rate.

We used annual mean air temperature and annual precipitation from Chinese surface meteorological stations as climate proxy. Those data were obtained from the dataset of monthly surface observation (http://data.cma.cn/data/cdcdetail/dataCode/SURF_CLI_CHN_MUL_MON.html), which was derived from monthly reports by each provincial meteorological department. The annual mean air temperature was calculated by the average of monthly air temperature, and the annual precipitation was calculated by the sum of monthly precipitation, a small proportion of temperature data (0.16%) data was not available in a few months, we assigned the temperature based on 50-yr mean temperature.

### Statistical analysis

Generalized additive models (GAM) were used to model the effects of human disturbance (annual GDP) and climate change (annual mean air temperature and annual precipitation) on population abundance of each species (or mixtures of populations with several unknown species) [68]. Time series of rodent abundance and environmental variables were normalized by (x-min)/range to remove the impact of spatial differences of environmental variables [69]. Density-dependency and 1-yr time delayed effect of population abundance of rodents and climate were included in the modeling analysis. Variables with strong and significant correlation (*r* < − 0.6 or *r* > 0.6; *p* < 0.05) with the other variables were removed to avoid collinearity effects (Additional file 1: Fig. S4). To model the effects of human disturbance and climate change on rodent abundance of each rodent species, a global Gaussian GAMs of the population abundance ${\mathrm{Y}}_{\mathrm{t}}$(${\mathrm{Y}}_{\mathrm{t}}$) for each species against the density dependence, human disturbance, climate (temperature, and precipitation) was fitted by using the formula:

1 $${Y}_{t}={a}_{t}+{b}_{t-1}{D}_{t-1}+{c}_{t}{A}_{t}+{d}_{t}{T}_{t}+{e}_{t-1}{T}_{t-1}+{f}_{t}{P}_{t}+{g}_{t-1}{P}_{t-1}+H+s\left(Lon, Lat\right){+\varepsilon }_{t}$$

where, ${Y}_{t}$ was the normalized population abundance at time *t*, ${D}_{t-1}$ was the population abundance of last year, ${A}_{t}$ was the anthropogenic effects (i.e. GDP),${T}_{t}$ was the temperature, ${P}_{t}$ was the precipitation, $H$ was the habitat,$s\left(Lon, Lat\right)$ was a 2D smooth function (with *k* value, dimension of the basis = 4) for modeling the spatial autocorrelation effects [70]; ${\varepsilon }_{t}$ was uncorrelated random errors of zero mean and finite variance. ${a}_{t}$, ${b}_{t-1}$, ${c}_{t}$,$d$, ${e}_{t}$, ${f}_{t}$, and ${g}_{t}$ were constants (${a}_{t}$ was an intercept, ${b}_{t-1}$ and ${c}_{t}$ represented density dependent and anthropogenic (GDP) effects, ${d}_{t}$ and ${f}_{t}$, represented effects of temperature and precipitation, ${e}_{t-1}$ and ${g}_{t-1}$ represented one year delayed effects of temperature and precipitation on the population abundance ${Y}_{t}$ of species (or group)). For each species (or group), habitat and spatial autocorrelation were not considered if there were data with less than 5 prefectures. ${D}_{t-1}$ represents the density dependency which would help to minimize the temporal autocorrelation of time series.

A sub-model set of all possible sub-models was generated from the global model, the model selection criterion is AIC_C_ (Akaike Information Criterion correction for small sample size, which is commonly used and suitable for modelling linear responses, Additional file 1: Table S6) of each sub-model [71]. The process of GAM model analysis was shown in a schematic diagram (Additional file 1: Fig. S5).

To model the effects of body mass of rodents, habitat type, and latitudinal of a local population on the changing trend of rodent abundance, and their effects on association coefficients between population abundance and variables of human disturbance and climate change in Eq. 1, a Gaussian GAMs was fitted by using the formula:

2 $$Y=a+bB+cLat{+H+\varepsilon }_{t}$$

where, $Y$ was the changing trend (1 denotes increase, − 1 for decrease, and 0 for no trend) of rodent abundance, or association coefficients (i.e. ${c}_{t}$, ${d}_{t}$, ${e}_{t-1}$, ${f}_{t}$, and ${g}_{t-1}$ in Eq. 1 after model selection). $B$ was the body mass (g, log-transformed) of rodent species, $Lat$ was the latitude of a local population, $H$ was the habitat type; ${\varepsilon }_{t}$ was uncorrelated random errors of zero mean and finite variance. $a$, $b$, and $c$ were constants ($a$ was an intercept, $b$ and $c$ represented the effects of body mass of rodent and latitude of the local population).

We used linear regression to detect the changing trend of population abundance of each known rodent species or from mixed populations of unknown species. We quantified temporal variation in each time series by a kernel-smoothed estimate of the 10%, 50%, and 90% quantile of the normalized data sliding along the time series, which showed the amplitude of abundance valley (≥ 10%), hillside (≥ 50%), and peak (≥ 90%). We used wavelet analysis to identify nonstationary periodic characteristics of the frequency and amplitude of detrended population oscillations vary through time [72]. The wavelet power spectrum represents the integration of population oscillation strength over the influence period (time scale) and the series spans [73].

Linear regression was carried out via stats library in R (v.4.1.3) [74]. Kernel-smoothed quantiles estimation was carried out via KernSmooth library (v. 2.23-15) in R (v. 4.1.3) [74]. Generalized Additive Models (GAM) were carried out using the mgcv library (v. 1.8-15) [75] and MuMIn library (v. 1.43.6) [76] in R (v. 4.1.3). Wavelet analysis and time series detrending were carried out via biwavelet (v.0.20.19) and mFilter (v.0.1-5) library in R (v. 4.1.3) [77, 78].

## Supplementary Information

**Additional file 1: **Tables S1 to S6. Figure S1 to S5.

**Additional file 2: Data S1.** Data used in this study.

## Data Availability

Data used in this study was provided in Additional file 2: Data S1.
