# Supplementary material for: Sustained population decline of rodents is linked to accelerated climate warming and human disturbance
Source: BMC Ecol Evol. 2022 Aug 22;22:102. doi: 10.1186/s12862-022-02056-z (PMC9394043; doi:10.1186/s12862-022-02056-z)
Supplement: Supplementary file 1 — Additional file 1: Tables S1 to S6. Figure S1 to S5. [file 12862_2022_2056_MOESM1_ESM.docx]

SUPPLEMENTAL INFORMATION

**Sustained population decline of rodents is linked to accelerated climate warming and human disturbance**

Xinru Wan ^1^, Chuan Yan ^1^, Zhenyu Wang ^2^, Zhibin Zhang^1, 3*^

^1^ State Key Laboratory of Integrated Management on Pest Insects and Rodents, Institute of Zoology, Chinese Academy of Sciences, Beijing 100101, China.

^2^ College of Life Sciences, Jiangxi Normal University, Nanchang 330022, China.

^3^ CAS Centre for Excellence in Biotic Interactions, University of Chinese Academy of Sciences, Beijing 100049, China.

* Corresponding author: zhangzb@ioz.ac.cn

**Contents**

Supplementary Tables S1 to S6

Supplementary Figure S1 to S5

**Supplementary Table S1 Summary of distribution, habitat, biomass, activity rhythm and litter size of 18 rodent species in this study.** Data was extracted from 4 Chinese books: *A Field Guide to the Mammals of China*, *Fauna Sinica (Mammalia, vol 8: Carnivora)*, *A Guide to the Mammals of China*, and *Color Knowledge Graph to the Mammals of China*.

| Latin Name of species | Chinese Name | English Name | Distribution  in China | Main Habitat | Biomass  (g) | Activity Rhythm | Litter size |
| --- | --- | --- | --- | --- | --- | --- | --- |
| *Rattus norvegicus* | 褐家鼠 | Brown Rat | All | City, farmland | 325 | All | 5-14 |
| *Apodemus agrarius* | 黑线姬鼠 | Striped Field Mouse | All | Farmland, forest | 32.5 | Night | 2-10 |
| *Mus musculus* | 小家鼠 | House Mouse | All | City, farmland | 13.5 | Night | 5-7 |
| *Rattus tanezumi* | 黄胸鼠 | Tanezumi Rat | South | City, farmland | 108.5 | Night | 3-7 |
| *Meriones unguiculatus* | 长爪沙鼠 | Mongolian Gerbil | Northeast | Grassland, farmland, dessert | 54.7 | Day | 5-11 |
| *Rattus losea* | 黄毛鼠 | Lesser Rice Rat | South | Farmland | 97.5 | All | 2-13 |
| *Rhombomys opimus* | 大沙鼠 | Great Gerbil | Northwest | Dessert | 181.5 | Day | 5-6 |
| *Niviventer confucianus* | 社鼠 | Chinese White-bellied Rat | All | Forest, farmland | 92.5 | Night | 1-9 |
| *Apodemus peninsulae* | 朝鲜姬鼠 | Korean Filed Mouse | North and  southwest | Forest, farmland | 33 | Night | 4-9 |
| *Rattus nitidus* | 大足鼠 | White-Footed Indochinese Rat | South | City, farmland | 125 | All | 4-15 |
| *Bandicota indica* | 板齿鼠 | Greater Bandicoot Rat | South | Farmland | 525 | Night | 2-10 |
| *Spermophilus dauricus* | 达乌尔黄鼠 | Daurian Ground Squirrel | Northeast | Grassland, dessert | 226 | Day | 1-9 |
| *Spermophilus alashanicus* | 阿拉善黄鼠 | Alashan Ground Squirrel | Northwest | Grassland, dessert | 208 | Day | 1-9 |
| *Marmota sibirica* | 西伯利亚旱獭 | Tarbagan Marmot | Northeast | Grassland | 5000 | Day | 4-6 |
| *Cricetulus barabensis* | 黑线仓鼠 | Striped Hamster | North | Grassland, dessert, farmland | 28 | Night | 2-10 |
| *Tscherskia triton* | 大仓鼠 | Greater Long-Tailed Hamster | Central And  Northeast | Farmland, grassland | 108.2 | Night | 8-10 |
| *Phodopus sungorus* | 黑线毛足鼠 | Striped hairy-footed hamster | Northeast | Grassland, desert | 23 | Night | 4-9 |
| *Allactaga sibirica* | 五趾跳鼠 | Mongolian Five-toed Jerboa | North | Grassland, dessert | 109 | Night | 2-9 |

**Supplementary Table S2 Summary on the changing trend of 115 time series** **(80 populations from 18 known rodent species, 35 mixed populations from unknown species) of rodents in China based on linear regression.**

| Species/groups | Time series number of changing trend | | | |
| --- | --- | --- | --- | --- |
|  | Increase | Decrease | No trend | Sum |
| Pooled populations | 16 | 54 | 45 | 115 |
| Mixed populations | 4 | 20 | 11 | 35 |
| *Rattus norvegicus* | 2 | 7 | 4 | 13 |
| *Apodemus agrarius* | 0 | 5 | 7 | 12 |
| *Spermophilus dauricus* | 1 | 4 | 5 | 10 |
| *Spermophilus alashanicus* | 8 | 0 | 0 | 8 |
| *Rattus tanezumi* | 0 | 3 | 3 | 6 |
| *Mus musculus* | 0 | 4 | 2 | 6 |
| *Cricetulus barabensis* | 0 | 2 | 3 | 5 |
| *Meriones unguiculatus* | 0 | 1 | 4 | 5 |
| *Tscherskia triton* | 0 | 3 | 1 | 4 |
| *Allactaga sibirica* | 0 | 1 | 1 | 2 |
| *Rattus losea* | 0 | 2 | 0 | 2 |
| *Phodopus sungorus* | 0 | 0 | 1 | 1 |
| *Marmota sibirica* | 0 | 0 | 1 | 1 |
| *Bandicota indica* | 1 | 0 | 0 | 1 |
| *Rattus nitidus* | 0 | 1 | 0 | 1 |
| *Apodemus peninsulae* | 0 | 0 | 1 | 1 |
| *Niviventer confucianus* | 0 | 1 | 0 | 1 |
| *Rhombomys opimus* | 0 | 0 | 1 | 1 |

**Supplementary Table S3 Summary on the sampling effort and changing trend of 115 time series of rodents in China based on linear regression.**

| Refe  rence | Species | Sampling effort | | | | Changing trend |
| --- | --- | --- | --- | --- | --- | --- |
|  |  | Frequency | Area (hm^2^) | Number of traps | Number of captured |  |
| [1] | *Apodemus agrarius* | Semiannual (Apr.&Sep.) |  | 42114 | 5098 | Decrease |
| [1] | *Rattus norvegicus* | Semiannual (Apr.&Sep.) |  | 21592 | 1783 | Decrease |
| [2] | *Spermophilus alaschanicus* | Semiannual (Apr.&Jul.) | 2194 |  | 3003 | Increase |
| [3] | *Apodemus agrarius* | Monthly (May-Oct.) |  | 35084 | 3421 | No trend |
| [3] | *Mus musculus* | Monthly (May-Oct.) |  | 21601 | 1452 | Decrease |
| [3] | *Rattus norvegicus* | Monthly (May-Oct.) |  | 21601 | 1452 | No trend |
| [3] | *Rattus norvegicus* | Monthly (May-Oct.) |  | 35084 | 3421 | Decrease |
| [4] | Mixed population | NA |  | 74658 | 2193 | Decrease |
| [4] | Mixed population | NA |  | 77032 | 2739 | Decrease |
| [5] | Mixed population | NA |  | 14206 | 863 | Decrease |
| [5] | Mixed population | NA |  | 14206 | 863 | Decrease |
| [6] | *Spermophilus alaschanicus* | Semiannual (Apr.&Jul.) |  | NA | NA | Increase |
| [7] | *Meriones unguiculatus* | Semiannual (Apr.&Oct.) | 3430 |  | 25345 | No trend |
| [7] | Mixed population | Monthly |  | 18221 | 833 | No trend |
| [7] | Mixed population | Monthly |  | 41709 | 2508 | No trend |
| [8] | Mixed population | Semiannual (Apr.&Oct.) |  | 39154 | 1913 | No trend |
| [9] | *Mus musculus* | Monthly |  | 167800 | 9845 | No trend |
| [9] | *Rattus norvegicus* | Thrice-monthly |  | 167800 | 9845 | Decrease |
| [9] | *Rattus norvegicus* | Monthly |  | 167800 | 9845 | Increase |
| [9] | *Rattus tanezumi* | Monthly |  | 167800 | 9845 | Decrease |
| [9] | *Rattus tanezumi* | Monthly |  | 167800 | 9845 | No trend |
| [10] | *Spermophilus alaschanicus* | Semiannual (Apr.&Jul.) | 2310 |  | 2252 | Increase |
| [11] | *Rattus losea* | Monthly |  | 158462 | 20631 | Decrease |
| [11] | *Rattus norvegicus* | Monthly |  | 158462 | 20631 | Decrease |
| [11] | *Rattus tanezumi* | Monthly |  | 158462 | 20631 | Decrease |
| [12] | *Spermophilus alaschanicus* | Semiannual (Apr.&Jul.) |  | NA | NA | Increase |
| [13] | *Meriones unguiculatus* | Semiannual (Apr.&Oct.) | 3740 |  | 1331 | Decrease |
| [13] | *Spermophilus dauricus* | Semiannual (Apr.&Oct.) | 3740 |  | 1331 | Increase |
| [14] | Mixed population | Monthly |  | 44226 | 1325 | Decrease |
| [14] | Mixed population | Monthly |  | 88448 | 1848 | Decrease |
| [15] | Mixed population | Semiannual (spring&fall) |  | 19105 | 610 | No trend |
| [16] | *Apodemus agrarius* | Monthly |  | 99215 | 2394 | No trend |
| [16] | *Mus musculus* | Monthly |  | 99215 | 2394 | No trend |
| [16] | *Rattus norvegicus* | Monthly |  | 99215 | 2394 | No trend |
| [17] | *Apodemus agrarius* | Monthly |  | 126000 | 1932 | Decrease |
| [17] | *Tscherskia triton* | Monthly |  | 126000 | 2242 | Decrease |
| [18] | Mixed population | Semiannual (spring&fall) |  | NA | NA | Increase |
| [19] | *Allactaga sibirica* | Monthly (Apr.‐Oct.) |  | 184800 | NA | Decrease |
| [19] | *Meriones unguiculatus* | Monthly (Apr.‐Oct.) |  | 184800 | NA | No trend |
| [19] | *Cricetulus barabensis* | Monthly (Apr.‐Oct.) |  | 184800 | NA | Decrease |
| [20] | *Apodemus agrarius* | Semiannual (Apr.&Sep.) |  | 66700 | NA | Decrease |
| [20] | *Rattus nitidus* | Semiannual (Apr.&Sep.) |  | 66700 | NA | Decrease |
| [20] | *Rattus norvegicus* | Semiannual (Apr.&Sep.) |  | 66700 | NA | Decrease |
| [21] | *Spermophilus dauricus* | Monthly (Apr.‐Sep.) | NA |  | NA | Decrease |
| [21] | *Spermophilus dauricus* | Monthly (Apr.‐Sep.) | NA |  | NA | No trend |
| [21] | *Spermophilus dauricus* | Monthly (Apr.‐Sep.) | NA |  | NA | Decrease |
| [21] | *Spermophilus dauricus* | Monthly (Apr.‐Sep.) | NA |  | NA | No trend |
| [21] | *Spermophilus dauricus* | Monthly (Apr.‐Sep.) | NA |  | NA | No trend |
| [22] | *Rhombomys opimus* | Semiannual (spring&fall) | NA |  | NA | No trend |
| [23] | *Rattus tanezumi* | NA |  | 3312525 | 77517 | Decrease |
| [24] | Mixed population | Monthly |  | 18000 | 1152 | No trend |
| [25] | *Spermophilus alaschanicus* | NA | NA |  | NA | Increase |
| [26] | *Bandicota indica* | Monthly |  | 138439 | 14652 | Increase |
| [26] | *Rattus losea* | Monthly |  | 138439 | 14652 | Decrease |
| [26] | *Rattus norvegicus* | Monthly |  | 138439 | 14652 | No trend |
| [26] | *Rattus tanezumi* | Monthly |  | 138439 | 14652 | No trend |
| [27] | *Spermophilus alaschanicus* | Annual | 7955 |  | 10423 | Increase |
| [28] | Mixed population | NA |  | 16850 | 559 | Decrease |
| [29] | *Meriones unguiculatus* | Monthly | 6529 |  | 10241 | No trend |
| [29] | *Spermophilus dauricus* | Monthly | 6529 |  | 10241 | No trend |
| [30] | *Allactaga sibirica* | Monthly |  | 82506 | 969 | No trend |
| [30] | *Cricetulus barabensis* | Monthly |  | 82506 | 969 | No trend |
| [30] | *Meriones unguiculatus* | Monthly |  | 82506 | 969 | No trend |
| [30] | *Phodopus sungorus* | Monthly |  | 82506 | 969 | No trend |
| [31] | *Marmota sibirica* | Semiannual (May&Jul.) |  | 97128 | 2271 | No trend |
| [31] | *Spermophilus dauricus* | Semiannual (May&Jul.) |  | 8220 | 2462 | No trend |
| [32] | Mixed population | Semiannual (spring&fall) |  | 10558 | 749 | No trend |
| [32] | Mixed population | Semiannual (spring&fall) |  | 10458 | 671 | Increase |
| [33] | *Apodemus agrarius* | Monthly (Apr.‐Sep.) |  | 41933 | 3926 | No trend |
| [33] | *Rattus norvegicus* | Monthly (Apr.‐Sep.) |  | 41933 | 3926 | No trend |
| [34] | Mixed population | Monthly |  | 45241 | 3740 | Decrease |
| [34] | Mixed population | Monthly |  | 77086 | 4592 | Decrease |
| [35] | *Apodemus agrarius* | Monthly |  | NA | NA | Decrease |
| [36] | *Spermophilus alaschanicus* | NA | 2080 |  | 1872 | Increase |
| [37] | Mixed population | Semiannual (spring&fall) |  | 18032 | 1681 | Increase |
| [37] | Mixed population | Semiannual (spring&fall) |  | 18032 | 1681 | Increase |
| [37] | *Spermophilus alaschanicus* | Semiannual (spring&fall) | 1340 |  | 1305 | Increase |
| [38] | *Spermophilus dauricus* | Semiannual (Apr.&Jul.) | 996 |  | 93 | Decrease |
| [39] | Mixed population | Monthly |  | NA | NA | Decrease |
| [40] | *Apodemus agrarius* | Semiannual (spring&fall) |  | 32251 | 1320 | No trend |
| [40] | *Apodemus peninsulae* | Semiannual (spring&fall) |  | 32251 | 1320 | No trend |
| [40] | *Niviventer confucianus* | Semiannual (spring&fall) |  | 32251 | 1320 | Decrease |
| [40] | *Tscherskia triton* | Semiannual (spring&fall) |  | 32251 | 1320 | No trend |
| [40] | *Apodemus agrarius* | Monthly |  | 237600 | 12900 | No trend |
| [40] | *Cricetulus barabensis* | Monthly |  | 237600 | 12900 | Decrease |
| [40] | *Mus musculus* | Monthly |  | 237600 | 12900 | Decrease |
| [40] | *Rattus norvegicus* | Monthly |  | 237600 | 12900 | Decrease |
| [40] | *Tscherskia triton* | Monthly |  | 237600 | 12900 | Decrease |
| [40] | *Apodemus agrarius* | Monthly |  | 237600 | 12900 | No trend |
| [40] | *Cricetulus barabensis* | Monthly |  | 237600 | 12900 | No trend |
| [40] | *Mus musculus* | Monthly |  | 237600 | 12900 | Decrease |
| [40] | *Tscherskia triton* | Monthly |  | 237600 | 12900 | Decrease |
| [41] | Mixed population | Semiannual (Apr.&Oct.) |  | 46509 | 2634 | No trend |
| [41] | Mixed population | Semiannual (Apr.&Oct.) | 3534 |  | 26170 | No trend |
| [42] | Mixed population | Monthly |  | 280800 | 3548 | Decrease |
| [43] | *Apodemus agrarius* | Semiannual (spring&fall) |  | 106265 | 7555 | No trend |
| [43] | *Rattus norvegicus* | Semiannual (spring&fall) |  | 106265 | 7555 | Increase |
| [44] | Mixed population | NA |  | NA | NA | No trend |
| [45] | Mixed population | Monthly (Apr.‐Jul.) |  | 10400 | 746 | No trend |
| [46] | Mixed population | NA |  | 40589 | 2189 | No trend |
| [46] | Mixed population | NA |  | 34691 | 1187 | Decrease |
| [47] | *Spermophilus dauricus* | Semiannual (Apr.&Jul.) | 5014 |  | 3733 | Decrease |
| [48] | Mixed population | Seasonal (Mar., Jun., Sep.) |  | 33303 | 2935 | Decrease |
| [49] | *Mus musculus* | Monthly |  | 75303 | 5738 | Decrease |
| [49] | *Rattus norvegicus* | Monthly |  | 75303 | 5738 | Decrease |
| [49] | *Rattus tanezumi* | Monthly |  | 75303 | 5738 | No trend |
| [49] | Mixed population | Monthly |  | 75303 | 5738 | Decrease |
| [50] | Mixed population | Monthly |  | 82763 | 6051 | Decrease |
| [50] | Mixed population | Monthly |  | 79840 | 3836 | Decrease |
| [51] | *Apodemus agrarius* | Monthly |  | NA | 3986 | Decrease |
| [52] | *Cricetulus barabensis* | Semiannual (Apr.&Oct.) |  | 264600 | NA | No trend |
| [53] | Mixed population | Monthly |  | 140219 | 7496 | Decrease |
| [54] | Mixed population | Monthly |  | 16604 | 751 | Decrease |
| [54] | Mixed population | Monthly |  | 32111 | 915 | Decrease |
| [54] | Mixed population | Monthly |  | 48715 | 1666 | Decrease |

**Reference for Table S3**

1. Zhang Zonghui, Luo Junyi, Wu Peng, Tian Hongrui, Jiang Beiping, Huang Shunhe, Luo Chengyuan.: HFRS surveillance in Nanchong, 1961-2010. Journal of Preventive Medicine Information 2012,28(04) (in Chinese)

2. Li Yong, Ji Zonglin: Analysis of plague surveillance results in Yuanzhou District, Guyuan City, Ningxia during 1977-2008. Endemic disease bulletin 2009,24(04) (in Chinese)

3. Liu Ziyuan, Liu Chengfu, Cui Lianying, Wu Wenbo, Liu Dengquan: Study on population composition and quantity change of rodents in Kaijang county of Sichuan province. Chinese journal of Vector Biology and Control 2009,20(04) (in Chinese)

4. Li Jianhua, Yang Bingyi, He Wei.: Epidemiological investigation on hemorrhagic fever with renal syndrime in Pingyi county from 1980 to 2006. Chinese journal of Vector Biology and Control 2009,20(01) (in Chinese)

5. Duan Wanhong, He Xingming: Analysis on plague monitoring results of Haiyuan from 1981 to 2010. Journal of Preventive Medicine Information 2005,(01) (in Chinese)

6. Duan Wanhong, He Xingming: Analysis of plague surveillance results in Haiyuan County, Ningxia during 1981-2010. Journal of medical pest control 2011,27(06) (in Chinese)

7. Li Xingqing, LI Weihua, Chen Baobao, Bai Jiangchun: Analysis of plague surveillance in Dingbian County during 1983-2010. Medical animal control 2011,27(07) (in Chinese)

8. Wei Shuhui, Wang Jing, Chu Hongna, Shang Jing, Zhao Guoliang, Zeng Qiang: Epidemiological investigation on hemorrhagic fever with renal syndrome in Chengde city from 1984 to 2008. Chinese journal of Vector Biology and Control 2010,21(06) (in Chinese)

9. Zhou Xianming, Pan Hui, Yi Zhiyong, Fan Yunxiu: Rodent population and its change regulation in Guanling county from 1984 to 2009. Guizhou Agricultural Sciences 2010,38(03) (in Chinese)

10. Chen Zhilin: Surveillance and analysis of plague in Caowa, Haiyuan County during 1985-2004. Ningxia Medical Journal 2007,(05) (in Chinese)

11. Zhang Bolin, Huang Wenhua, Lin Hongzhi, Chen Xuerong, Cai Yuemin: Surveilance on rat flea in Licheng district, Putian city, Fujian province during 1985 ~ 2004. Chinese Journal of Local Epidemiology 2005,(05) (in Chinese)

12. Qiao Fugui, Fu Weicheng, Bai Xueli, Wang Xinghu, Zhu Liangjun: Analysis of plague monitoring of Xiji county from 1986 to 2005. Endemic disease bulletin 2007,(06) (in Chinese)

13. Wang Zaishan: Analysis of plague surveillance results from 1992 to 2011 in Zhangbei County, Hebei Province. Hebei pharmaceutical 2014,36(06) (in Chinese)

14. Wei Yingmin, Liu Qing, Yang Dehui, Li Dingchao: Changes of rodent composition and population number in agricultural areas of Anlong County from 1994 to 2015. Modern agricultural technology 2016,(12) (in Chinese)

15. Zhang Ming, Lei,Wang: Surveillance of hemorrhagic fever with renal syndrome in Ganyu, 1995-2014. Modern preventive medicine 2016,43(07) (in Chinese)

16. Hu Jianzhong, Cheng Yu, Pan Quanxing, WANG Minggen, Tan Xiufang, Zhou Minfang, Wang Guoqing, Shen Weiguo: Investigation on the regularity of rodent infestation and evaluation of control effect in farmland of Baoshan District. Shanghai Agricultural Science and Technology 2007,(04) (in Chinese)

17. Yuan Zhiqiang, Dong Jie, Qiao Yan, Jia Haishan: The comparation of propagation ability between two mouse populations in farm fields of Shunyi district in Beijing. Advances in biotechnology 2016,6(02) (in Chinese)

18. Zhang Jinjun, Lin Tao, Wu Jie, Zhang Meiyin, Hu Liyun: Analysis of epidemic factors of hemorrhage fever in Binzhou city from 1984-2004. Preventive Medicine Forum 2005,(06) (in Chinese)

19. Dong Weihui, Hou Xixian, Yang Yuping, Wang Liqing, Xie Yongfeng: Studies on the population dynamics and prediction of several predominant rodents on grassland and farmland. Chinese Journal of Grassland 2008,(05) (in Chinese)

20. Liao Wenbo, Hu Jinchu, Li Cao, Liu Tao, Jiang Beiping: Structure of small mammal community and its periodic fluctuations in the central Sichuan square hills. Journal of Agricultural University of Hebei 2005,(02) (in Chinese)

21. Zhou Xiaolei: Preliminary study on forecasting and early warning for the *Spennophilus dauricus* focus in China. Chinese Center for Disease Control and Prevention (in Chinese)

22. Dang Huicai, Guo Zhengcai: Population dynamics and periodicity of gerbils. Xinjiang Animal Husbandry 2010,(11) (in Chinese)

23. Gong Suming, Luo Qisong, Liang Wei, Yang Zhenyuan, Duan Peizhi, Luo Jiali, Tian Zongmao, Cun Yongliang, Chang Lianmei: Monitoring and analysis of plague in Dehong prefecture from 1982 to 2009. Medical animal control 2011,27(02) (in Chinese)

24. Liu Xiaoping: Epidemiological trend of hemorrhagic fever with renal syndrome in Fushun area during 1985-2004. Strait Journal of Preventive Medicine 2005,(04) (in Chinese)

25. Quan Guoxi, Chen Gong: Review and analysis of plague in 45 years in Huining County, Gansu Province. Endemic disease bulletin 2009,24(03) (in Chinese)

26. Zhou Shuwu, Liang Jiangming, Zeng J Un, Liang Xiangfa, Wang Baihuan, Wen Ping: Study on the host and medium flea form the plague focus in Hepu county, Guangxi province. Chinese journal of Vector Biology and Control 2008,(03) (in Chinese)

27. Tian Dexi, Gao Jinhua, Tian Xingmei, Liu Ping: Investigation and analysis on the population and animal plague of yellow squirrels. Chinese Journal of Endemic Disease Control 2005,(01) (in Chinese)

28. Hu Haikuan: Surveillance of epidemic hemorrhagic fever in Lulong county of Hebei province during 1988 - 2009. Occupational and Health 2010,26(21) (in Chinese)

29. Yan Dong, Shi Xianming, Wang Zaishan, Cui Yaoren, Liu Guanchun, Zheng Nan, Li Yugui, Chen Yongming, Lan Xiaoyu, Chong Yanmin: Research on the population structure of plague host animals in plague foci in Hebei province. Chinese health insecticide equipment 2016,22(04) (in Chinese)

30. Yan Dong, Shi Xianming, Cui Yaoren, Xiang Youqing, Zhao Yugang, Liu Guanchun, Chen Li,Ren Fengqi, Chen Yongming: Investigation of small nocturnal rodents in a natural plague focus in Hebei province. Chinese journal of Vector Biology and Control 2011,22(04) (in Chinese)

31. Wang Liqing, Yang Yuping, Zhang Fushun, Hou Xixian, Dong Weihui: Dynamics of the rodent community in Hohhot district. Medical animal control 2012,28(04) (in Chinese)

32. Geng Guihua, Xu Sen, Z Hou Wei: Analysis on surveillance data of epidemic hemorrhage fever in Huantai county from 1983 to 2004. Preventive Medicine Forum 2006,(03) (in Chinese)

33. Liu Dengquan, Liu Ziyuan, Deng Xuejin.: A study for host animals of hemorrhagic fever with renal syndrome in Kaijiang county. Modern preventive medicine 2000,(04) (in Chinese)

34. Wen Bingzhi, Fan Jiangshun, Wen Jiaming, Li Zhongliang: Distribution and occurrence rule of agricultural rodents in Leishan county from 1985 to 2011. Guizhou Agricultural Sciences 2012,40(03) (in Chinese)

35. Wen Bingzhi, Yang Zaixue: The population dynamics and reproductive parameters of *Apodemus agrarius* in Leishan county. Journal of Mountain Agricultural Biology 2012,31(01) (in Chinese)

36. Wang Jizu, Jin Lijun, Li Shengming: A report on density dynamics of Yellow weasel in Alxa during 1977-1999, Guyuan County, Ningxia. Endemic disease bulletin 2001,(03) (in Chinese)

37. Zhao Jiude, Zhao Xinyong, Li Yong, Luo Feng: Retrospection and consideration of plague prevention and control in Guyuan County, Ningxia during 1981-2001. Endemic disease bulletin 2002,(04) (in Chinese)

38. Li Qi, Liu Zhonghai: Investigation of plague natural foci in Nongan County. Chinese Journal of Endemic Disease Control 2013,28(04) (in Chinese)

39. Cai Guoliang, Zhang Huadan, Xu Tieping, Zhu Jinxin, Qi Yonghuan, Wu Senxian: Species, distribution and fluctuation of rats on fields. Acta Agriculturae Zhejiangensis 2006,(03) (in Chinese)

40. Zhang Zhibin et al.,: Survey report by Institute of Zoology, Chinese Academy of Sciences. (in Chinese)

41. An Cuihong, Fan Suoping, Sun Yangxin: A 30-year surveillance of plague epizootic in plague epidemic area in Shaanxi province, China. Chinese journal of Vector Biology and Control 2014,25(04) (in Chinese)

42. Fei Lei, Wang Zuguo, Yao Yao, Xu Xiangming, Gu Pinqiang: Population change of farmland rodent and the influences of climate and cultivation factors in Fengxian District of Shanghai, China. Chinese Journal of Applied Ecology 2015,26(02) (in Chinese)

43. Chen Huizhong, Xu Tao, Dai Mengyang, Zhang Jin: Study on host change trend of hemorrhagic fever with renal syndrome in Shenyang, 1984 -2006. Chinese journal of Disease Control 2009,13(01) (in Chinese)

44. Zhu XiaoChun: Epidemiological analysis of hemorrhagic fever with renal syndrome in Shengzhou city. Zhejiang Preventive Medicine 2004,(04) (in Chinese)

45. Pan Hui Ming, Cheng Deming, Ruan Shao Yu, Hu Taifu, Wang Chenquan: Influence of the rodent density to the leptospirosis epidemic. Chinese journal of Vector Biology and Control 2003,(01) (in Chinese)

46. Wang Xiaohong, Xia Hongbo, Li Chenglin, He Jinkui: Surveillance and analysis of hemorrhagic fever with renal syndrome in Tangshan city during 1984-2005. Hebei pharmaceutical 2009,31(01) (in Chinese)

47. Zhang Peng, Zhang Fang, Song Guofu, Liu Zhencai: Analysis of variation trend of rodents and fleas in Plague foci of Taonan City. Chinese Journal of Endemic Disease Control 2012,27(06) (in Chinese)

48. Xie Datong, Zhang Yongzhi, Liang Changwei, Lou Fang: Analysis of rodent surveillance results from 1986 to 2013 in Tongzi County. Plant doctor 2014,27(04) (in Chinese)

49. Limei, Panshichang: Morphological characteristics and population dynamics of Rattus norvegicus in Xifeng county. Journal of Mountain Agricultural Biology 2010,29(02) (in Chinese)

50. Pan Shichang, Li Mei, Song Zhishu: Species composition and population density dynamics of rats in Xifeng county. Acta Agriculturae Jiangxi 2013,25(08) (in Chinese)

51. Pan Shichang, Li Mei: Occurrence dynamics of farmland *Apodemus agrarius* population quantity in Xifeng county. Guizhou Agricultural Sciences 2016,44(10) (in Chinese)

52. Wu Wenhua, Fu Heping, Wu Xiaodong, Dong Weihui. Xu Shengli: Forecasting the population dynamics of *Cricetulus barabansis* and *Meriones unguiculatus* by time series method. Journal of Inner Mongolia Agricultural University (Natural Science Edition) 2007,(04) (in Chinese)

53. Zheng Yuanli, Yang Zaixue, Hu Zhixian: Population composition and its fluctuation of agricultural rodent species in Yuqing county. Journal of Mountain Agricultural Biology 2011,30(05) (in Chinese)

54. Yang Gaogan: Study on the species and population changes of rodents in Zunyi County. Modern agricultural technology 2014,(11) (in Chinese)

**Supplementary Table S4 Correlations between rodent abundance and annual GDP, annual mean temperature, annual precipitation for the pooled populations, mixed populations, and populations of single species of rodents.** Bold values indicated the coefficients represent the significant correlation (* *p* < 0.05, ** *p* < 0.01, *** *p* < 0.001).

| Species/groups | Sample size | Annual GDP | Annual mean temperature | Annual precipitation |
| --- | --- | --- | --- | --- |
| Pooled populations | 2737 | **-0.21 ***** | **-0.08 ***** | **0.04 *** |
| Mixed populations | 812 | **-0.23 ***** | -0.07 . | 0.04 |
| *Rattus norvegicus* | 305 | **-0.29 ***** | **-0.23 ***** | 0.08 |
| *Apodemus agrarius* | 295 | **-0.4 ***** | **-0.18 **** | 0.02 |
| *Mus musculus* | 142 | **-0.37 ***** | **-0.2 *** | 0.14 |
| *Rattus tanezumi* | 142 | **-0.33 ***** | **-0.33 ***** | **0.18 *** |
| *Meriones unguiculatus* | 117 | **-0.29 **** | -0.01 | 0.16 . |
| *Rattus losea* | 40 | **-0.8 ***** | **-0.33 *** | 0.11 |
| *Rhombomys opimus* | 27 | -0.17 | -0.19 | -0.23 |
| *Niviventer confucianus* | 24 | -0.38 . | -0.1 | -0.05 |
| *Apodemus peninsulae* | 24 | -0.01 | -0.05 | 0 |
| *Rattus nitidus* | 25 | **-0.5 *** | -0.35 . | 0.32 |
| *Bandicota indica* | 20 | **0.82 ***** | 0.14 | 0.02 |
| *Spermophilus dauricus* | 252 | **-0.15 *** | 0.1 | 0.09 |
| *Spermophilus alashanicus* | 192 | **0.45 ***** | **0.56 ***** | -0.1 |
| *Marmota sibirica* | 30 | -0.21 | -0.34 . | 0.17 |
| *Cricetulus barabensis* | 122 | **-0.23 **** | **-0.3 ***** | 0.01 |
| *Tscherskia triton* | 102 | **-0.58 ***** | 0 | -0.01 |
| *Phodopus sungorus* | 21 | -0.31 | -0.15 | 0.21 |
| *Allactaga sibirica* | 45 | -0.24 | -0.18 | -0.23 |

**Supplementary Table S5 Effects of body mass, habitat, and latitude on the associations coefficients between population abundance and variables of climate change and human disturbance.** The analysis is based on GAM analysis using Equation (2) (* *p* <0.05，** *p* <0.01，*** *p* <0.001).

| Variables | Biomass  (g) | Habitat | | | | Longitude |
| --- | --- | --- | --- | --- | --- | --- |
|  |  | Grassland- farmland mosaic | City | Farmland | Forest |  |
| GDP | 5.60E-08 | -1.63E-06  *** | -2.12E-06  *** | -1.79E-06  *** | -1.63E-06  ** | -2.45E-08 |
| Temperature | -0.01358 | 0.2316456 | -0.407553  * | -0.321655  ** | -0.101389 | -0.013951 |
| Precipitation | 7.02E-05 | -0.000172 | -0.000729 | -0.000974 | NA | 2.66E-07 |

**Supplementary Table S6 Associations** **of rodent abundance with the intrinsic factor, human disturbance factor, climate factors, habitat, and spatial autocorrelation based on analyses using Equation (1) and their AICc of sub-models for model selection.** Only sub-models with lowest 5 AICc were presented due to page limit. Bold values indicated the coefficients represent the effects. + denotes the significant effects of habitat and Spatial autocorrelation (*p* < 0.05). # denotes 1-yr time lag of climate factors. NA denotes not analyzed due to small number of locations. Only sub-model with lowest AICc for each species/groups was selected for further analysis.

| Species/groups | Rodent abundance of last year | Annual GDP | Annual mean temperature | Annual precipitation | Habitat | Spatial auto correlation | AICc |
| --- | --- | --- | --- | --- | --- | --- | --- |
| Pooled populations | 0.62 | -0.03# | 0.03# | -0.07 | + | + | -491.65 |
| Pooled populations | 0.62 | -0.03# | 0.03# | -0.07 |  | + | -490.66 |
| Pooled populations | 0.62 | -0.04# | 0.03# | -0.06 | + |  | -490.24 |
| Pooled populations | 0.62 | -0.03# | 0.01, 0.03# | -0.07 | + | + | -490.09 |
| Pooled populations | 0.62 |  | 0.04# | -0.07 | + | + | -489.84 |
| Mixed populations | 0.69 | -0.07# |  |  |  |  | -277.4 |
| Mixed populations | 0.69 | -0.07# | 0.03# |  |  |  | -276.89 |
| Mixed populations | 0.69 | -0.07# |  | -0.02 |  |  | -275.9 |
| Mixed populations | 0.69 | 0.02, -0.08# |  |  |  |  | -275.67 |
| Mixed populations | 0.69 | -0.07# | 0 |  |  |  | -275.4 |
| *Rattus norvegicus* | 0.48 | -0.1 |  | -0.12 | + |  | -17.88 |
| *Rattus norvegicus* | 0.49 | -0.11, 0.04# |  | -0.13 | + |  | -16.32 |
| *Rattus norvegicus* | 0.48 | -0.1 | 0.03 | -0.12 | + |  | -16.17 |
| *Rattus norvegicus* | 0.48 | -0.1 | -0.01# | -0.12 | + |  | -15.81 |
| *Rattus norvegicus* | 0.49 |  |  | -0.16 | + |  | -15.74 |
| *Apodemus agrarius* | 0.48 | -0.14# | 0.09# | -0.15 | + |  | -47.92 |
| *Apodemus agrarius* | 0.51 | -0.13# | 0.08# | -0.15 |  |  | -47.59 |
| *Apodemus agrarius* | 0.51 | -0.15# |  | -0.15 |  |  | -47.13 |
| *Apodemus agrarius* | 0.51 | -0.13# | -0.06, 0.09# | -0.15 |  |  | -46.95 |
| *Apodemus agrarius* | 0.49 | -0.16# |  | -0.15 | + |  | -46.79 |
| *Mus musculus* | 0.25 |  | 0.14 | -0.25 | + | + | -0.08 |
| *Mus musculus* | 0.26 |  | 0.13 | -0.24 |  | + | 0.6 |
| *Mus musculus* | 0.25 |  |  | -0.26 | + | + | 0.91 |
| *Mus musculus* | 0.27 |  |  | -0.26 |  | + | 1.09 |
| *Mus musculus* | 0.25 | 0.03 | 0.14 | -0.26 | + | + | 1.99 |
| *Rattus tanezumi* | 0.64 | -0.11 |  |  |  |  | -79.53 |
| *Rattus tanezumi* | 0.63 | -0.1 | 0.08 |  |  |  | -78.83 |
| *Rattus tanezumi* | 0.66 |  |  |  |  |  | -77.99 |
| *Rattus tanezumi* | 0.65 |  | 0.09 |  |  |  | -77.92 |
| *Rattus tanezumi* | 0.65 | -0.11 | -0.04# |  |  |  | -77.83 |
| *Meriones unguiculatus* | 0.54 |  |  | -0.18 |  |  | -15.37 |
| *Meriones unguiculatus* | 0.55 | -0.1# |  | -0.16 |  |  | -15.01 |
| *Meriones unguiculatus* | 0.54 |  | 0.04 | -0.17 |  |  | -13.41 |
| *Meriones unguiculatus* | 0.54 |  | 0.03# | -0.17 |  |  | -13.35 |
| *Meriones unguiculatus* | 0.54 | -0.01 |  | -0.17 |  |  | -13.19 |
| *Rattus losea* | 0.43 |  |  | -0.61 | NA | NA | -17.66 |
| *Rattus losea* | 0.45 |  | -0.14# | -0.6 | NA | NA | -16.75 |
| *Rattus losea* | 0.42 | 0.05# |  | -0.64 | NA | NA | -15.23 |
| *Rattus losea* | 0.42 | 0.05 |  | -0.64 | NA | NA | -15.18 |
| *Rattus losea* | 0.44 |  | -0.03 | -0.6 | NA | NA | -15.1 |
| *Rhombomys opimus* | NA |  |  |  | NA | NA | 7.58 |
| *Rhombomys opimus* | NA |  | -0.25 |  | NA | NA | 8.89 |
| *Rhombomys opimus* | 0.19 |  |  |  | NA | NA | 8.91 |
| *Rhombomys opimus* | NA |  | -0.2# |  | NA | NA | 9.39 |
| *Rhombomys opimus* | NA | -0.11 |  |  | NA | NA | 9.94 |
| *Niviventer confucianus* | NA |  |  | -0.25 | NA | NA | -1.66 |
| *Niviventer confucianus* | NA |  |  |  | NA | NA | -1.2 |
| *Niviventer confucianus* | 0.3 |  |  |  | NA | NA | -0.78 |
| *Niviventer confucianus* | NA | -0.22# |  |  | NA | NA | -0.08 |
| *Niviventer confucianus* | 0.19 |  |  | -0.2 | NA | NA | 0.47 |
| *Apodemus peninsulae* | NA | -0.39# |  |  | NA | NA | -1.17 |
| *Apodemus peninsulae* | NA | -0.5# |  | 0.2 | NA | NA | -0.04 |
| *Apodemus peninsulae* | NA |  |  |  | NA | NA | 0.94 |
| *Apodemus peninsulae* | NA | 0.11, -0.43# |  |  | NA | NA | 1.35 |
| *Apodemus peninsulae* | NA | -0.39# | -0.06# |  | NA | NA | 1.61 |
| *Rattus nitidus* | 0.35 |  |  | -0.4 | NA | NA | 2.85 |
| *Rattus nitidus* | NA |  | 0.36, 0.41# | -0.42 | NA | NA | 3.08 |
| *Rattus nitidus* | NA |  | 0.36# | -0.5 | NA | NA | 3.48 |
| *Rattus nitidus* | NA |  |  | -0.58 | NA | NA | 3.82 |
| *Rattus nitidus* | 0.29 |  | 0.29# | -0.37 | NA | NA | 3.83 |
| *Bandicota indica* | 0.82 |  |  |  | NA | NA | -4.12 |
| *Bandicota indica* | 0.83 |  | 0.14 |  | NA | NA | -1.8 |
| *Bandicota indica* | 0.83 | -0.09# |  |  | NA | NA | -1.26 |
| *Bandicota indica* | 0.84 | -0.08 |  |  | NA | NA | -1.15 |
| *Bandicota indica* | 0.82 |  | -0.04# |  | NA | NA | -0.95 |
| *Spermophilus dauricus* | 0.55 |  | 0.1# |  |  |  | -3.82 |
| *Spermophilus dauricus* | 0.54 | 0.07 | 0.11# |  |  |  | -3.28 |
| *Spermophilus dauricus* | 0.54 |  | 0.09# | -0.07 |  |  | -3.07 |
| *Spermophilus dauricus* | 0.55 | 0.11, -0.08# | 0.1# |  |  |  | -2.97 |
| *Spermophilus dauricus* | 0.52 |  |  | -0.09 |  | + | -2.75 |
| *Spermophilus alaschanicus* | 0.82 | 0.16# |  |  |  |  | -135.02 |
| *Spermophilus alaschanicus* | 0.81 | 0.17# | 0.02# |  |  |  | -133.08 |
| *Spermophilus alaschanicus* | 0.82 | 0.16# | 0.02 |  |  |  | -133.05 |
| *Spermophilus alaschanicus* | 0.82 | 0.17# | 0.03, 0.03# |  |  |  | -131.29 |
| *Spermophilus alaschanicus* | 0.89 |  |  |  |  |  | -128.55 |
| *Marmota sibirica* | 0.36 | -0.27# |  |  | NA | NA | -12.6 |
| *Marmota sibirica* | 0.41 | -0.25# | 0.18 |  | NA | NA | -11.83 |
| *Marmota sibirica* | 0.38 | -0.26 |  |  | NA | NA | -11.69 |
| *Marmota sibirica* | 0.48 |  |  |  | NA | NA | -11.55 |
| *Marmota sibirica* | 0.52 |  | 0.2 |  | NA | NA | -11.24 |
| *Cricetulus barabensis* | 0.31 |  | 0.22# |  |  |  | -52.1 |
| *Cricetulus barabensis* | 0.3 |  | 0.22# | -0.06 |  |  | -50.94 |
| *Cricetulus barabensis* | 0.29 | -0.06# | 0.21# |  |  |  | -50.75 |
| *Cricetulus barabensis* | 0.3 | -0.05 | 0.22# |  |  |  | -50.4 |
| *Cricetulus barabensis* | 0.31 |  | -0.01, 0.22# |  |  |  | -49.93 |
| *Tscherskia triton* | 0.5 |  |  | -0.28 | NA | NA | -21.74 |
| *Tscherskia triton* | 0.5 |  | 0.09# | -0.3 | NA | NA | -21.02 |
| *Tscherskia triton* | 0.5 | -0.09# |  | -0.29 | NA | NA | -21 |
| *Tscherskia triton* | 0.49 | -0.08# | 0.08# | -0.3 | NA | NA | -19.94 |
| *Tscherskia triton* | 0.51 | -0.03 |  | -0.28 | NA | NA | -19.73 |
| *Phodopus sungorus* | NA |  |  |  | NA | NA | 0.52 |
| *Phodopus sungorus* | NA |  |  | -0.27 | NA | NA | 0.88 |
| *Phodopus sungorus* | -0.29 |  |  | -0.34 | NA | NA | 2.24 |
| *Phodopus sungorus* | NA |  | 0.16 |  | NA | NA | 2.47 |
| *Phodopus sungorus* | -0.17 |  |  |  | NA | NA | 2.75 |
| *Allactaga sibirica* | 0.49 |  | -0.24 |  | NA | NA | 10.08 |
| *Allactaga sibirica* | 0.49 |  |  |  | NA | NA | 10.73 |
| *Allactaga sibirica* | 0.46 | -0.19 | -0.28 |  | NA | NA | 11.14 |
| *Allactaga sibirica* | 0.45 |  | -0.26 | -0.17 | NA | NA | 11.21 |
| *Allactaga sibirica* | 0.45 |  |  | -0.14 | NA | NA | 12.26 |


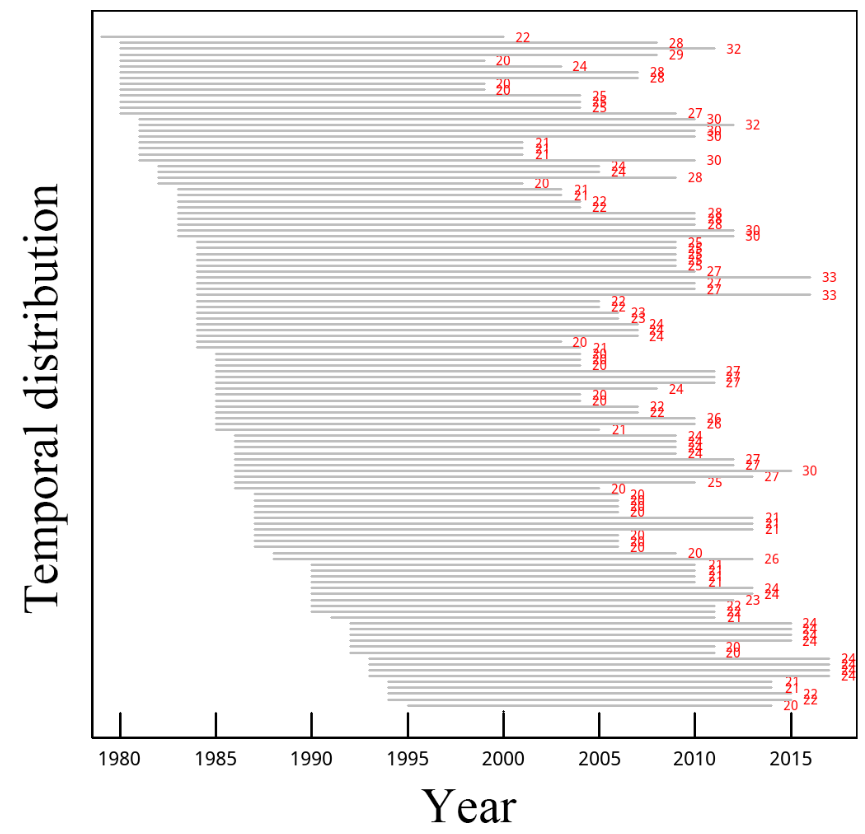


**Supplementary Figure S1. Temporal duration of 115 time series.** Grey line represents the period of each time series. Mixed population means time series of different rodent species in one local time (n =35).


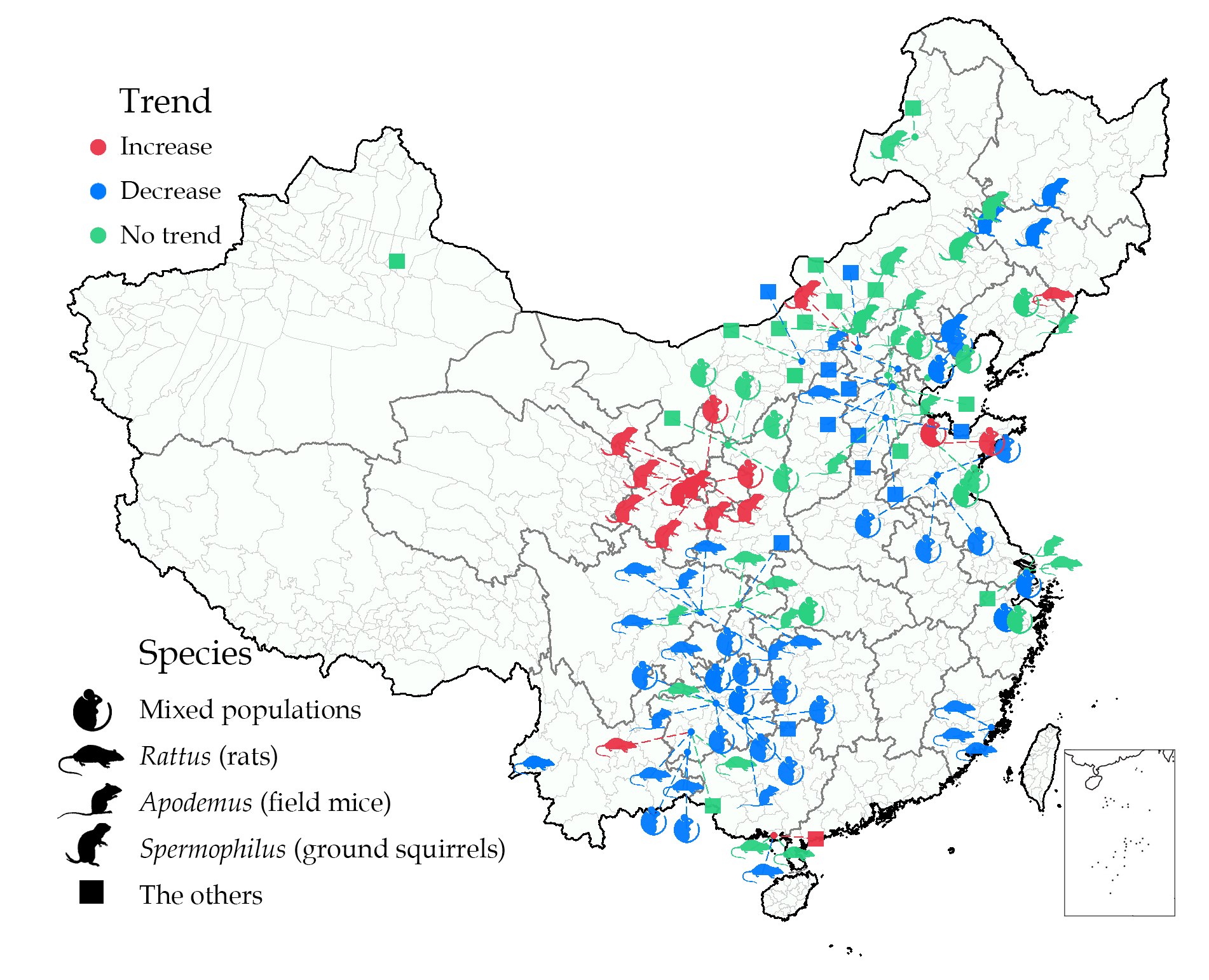


**Supplementary Figure S2. Spatial distribution of 115 time series (80 populations from 18 known rodent species, 35 mixed populations from unknown species) of rodents in China and the changing trend of 18 rodent species.** The symbols on the map show the sites of the 115 time series, and the shapes of the symbols indicate the rodent groups. The color of symbols or lines indicates the changing trend of the time series: increase (red), decrease (blue), and no trend (green).


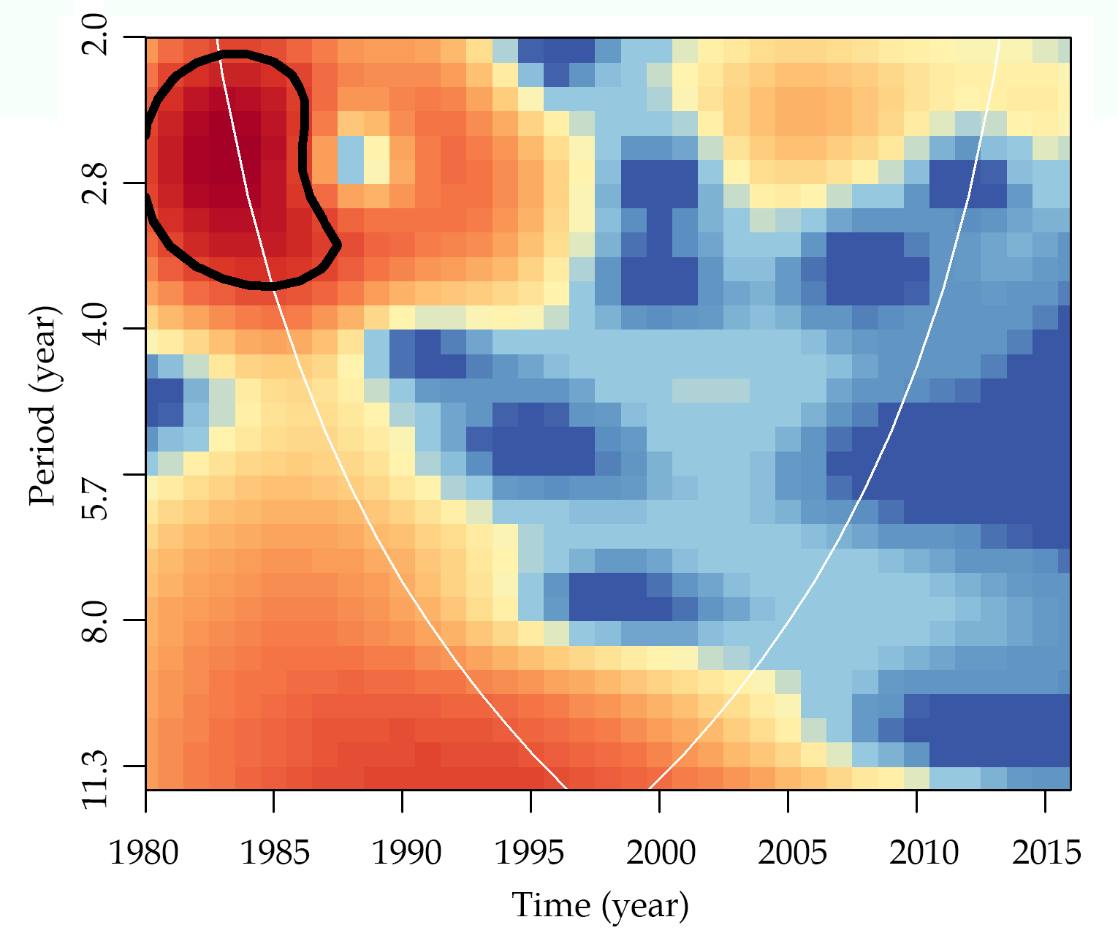


**Supplementary Figure S3. Wavelet power spectra for the pooled rodent abundance during 1980-2020.** Time series of pooled rodent abundance (as represented by the blue solid line of Fig.3A) were detrended to focus on the analysis of population cyclicity. The colors from blue to red indicate the increasing power, and the solid black contours represent the significance of local power relative to the noise at a 0.05 level.


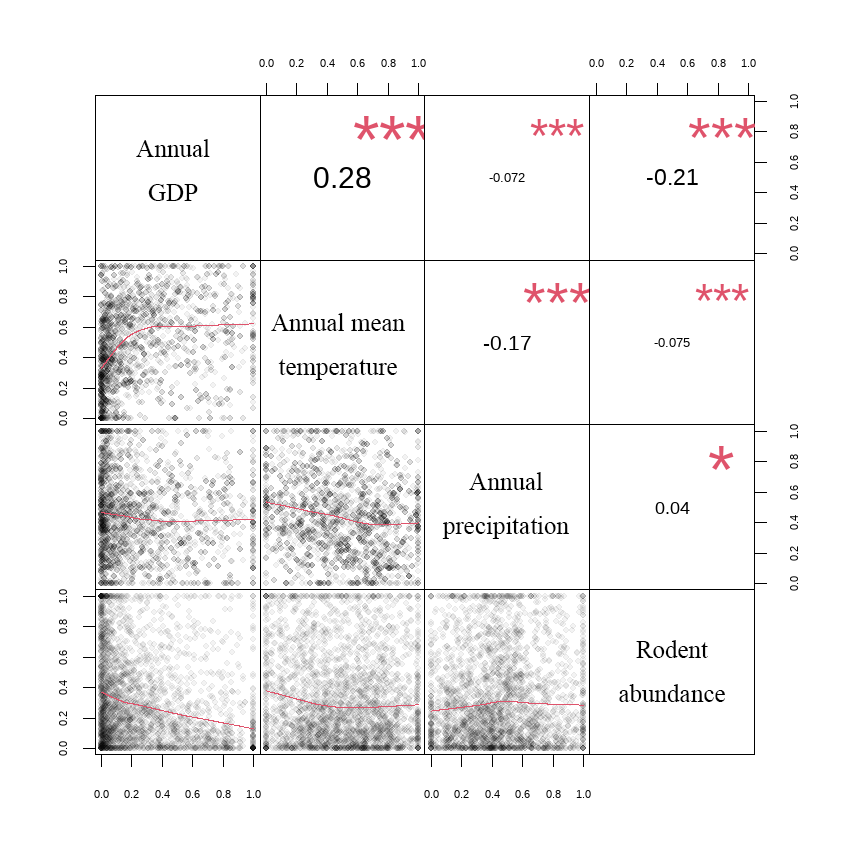


**Supplementary Figure S4. Pearson’s correlation between rodent abundance, annual mean temperature, annual precipitation, and annual GDP for pooled time series data.** **p* < 0.05, ***p* < 0.01, ****p* < 0.001.


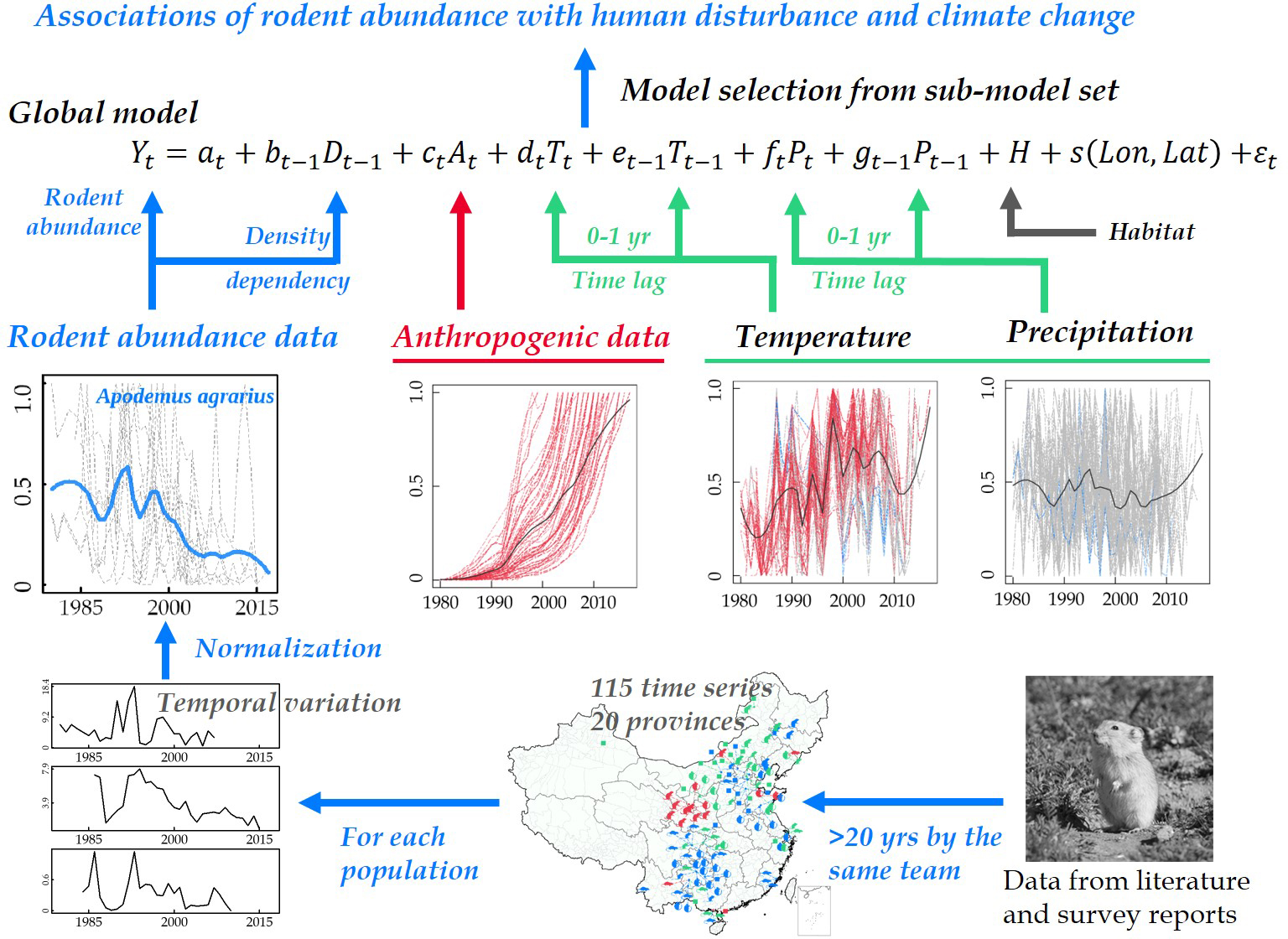


**Supplementary Figure S5. The schematic diagram of the process of model analysis.**
