# Supplementary material for: Sustained population decline of rodents is linked to accelerated climate warming and human disturbance
Source: BMC Ecol Evol. 2022 Aug 22;22:102. doi: 10.1186/s12862-022-02056-z (PMC9394043; doi:10.1186/s12862-022-02056-z)
Supplement: Supplementary file 2 — Additional file 2: Data S1. Data used in this study. [file 12862_2022_2056_MOESM2_ESM.docx]

SUPPLEMENTAL INFORMATION

**Sustained population decline of rodents is linked to accelerated climate warming and human disturbance**

Xinru Wan ^1^, Chuan Yan ^1^, Zhenyu Wang ^2^, Zhibin Zhang^1, 3*^

^1^ State Key Laboratory of Integrated Management on Pest Insects and Rodents, Institute of Zoology, Chinese Academy of Sciences, Beijing 100101, China.

^2^ College of Life Sciences, Jiangxi Normal University, Nanchang 330022, China.

^3^ CAS Centre for Excellence in Biotic Interactions, University of Chinese Academy of Sciences, Yuquan Road, Beijing 100049, China.

* Corresponding author: zhangzb@ioz.ac.cn

**Contents**

Data table: Data S1

Data table:

Data S1. Data used in this study.

| NO. | Location | Species | Habitat | Data type | Year | Abundance |
| --- | --- | --- | --- | --- | --- | --- |
| 1 | Beijing | *Apodemus agrarius* | Farmland | Trap success | 1994 | 0.91 |
| 2 | Beijing | *Apodemus agrarius* | Farmland | Trap success | 1995 | 0.62 |
| 3 | Beijing | *Apodemus agrarius* | Farmland | Trap success | 1996 | 0.43 |
| 4 | Beijing | *Apodemus agrarius* | Farmland | Trap success | 1997 | 0.46 |
| 5 | Beijing | *Apodemus agrarius* | Farmland | Trap success | 1998 | 1.00 |
| 6 | Beijing | *Apodemus agrarius* | Farmland | Trap success | 1999 | 0.56 |
| 7 | Beijing | *Apodemus agrarius* | Farmland | Trap success | 2000 | 0.40 |
| 8 | Beijing | *Apodemus agrarius* | Farmland | Trap success | 2001 | 0.36 |
| 9 | Beijing | *Apodemus agrarius* | Farmland | Trap success | 2002 | 0.30 |
| 10 | Beijing | *Apodemus agrarius* | Farmland | Trap success | 2003 | 0.13 |
| 11 | Beijing | *Apodemus agrarius* | Farmland | Trap success | 2004 | 0.44 |
| 12 | Beijing | *Apodemus agrarius* | Farmland | Trap success | 2005 | 0.25 |
| 13 | Beijing | *Apodemus agrarius* | Farmland | Trap success | 2006 | 0.13 |
| 14 | Beijing | *Apodemus agrarius* | Farmland | Trap success | 2007 | 0.00 |
| 15 | Beijing | *Apodemus agrarius* | Farmland | Trap success | 2008 | 0.07 |
| 16 | Beijing | *Apodemus agrarius* | Farmland | Trap success | 2009 | 0.15 |
| 17 | Beijing | *Apodemus agrarius* | Farmland | Trap success | 2010 | 0.03 |
| 18 | Beijing | *Apodemus agrarius* | Farmland | Trap success | 2011 | 0.02 |
| 19 | Beijing | *Apodemus agrarius* | Farmland | Trap success | 2012 | 0.01 |
| 20 | Beijing | *Apodemus agrarius* | Farmland | Trap success | 2013 | 0.09 |
| 21 | Beijing | *Apodemus agrarius* | Farmland | Trap success | 2014 | 0.07 |
| 22 | Beijing | *Tscherskia triton* | Farmland | Trap success | 1994 | 0.92 |
| 23 | Beijing | *Tscherskia triton* | Farmland | Trap success | 1995 | 0.40 |
| 24 | Beijing | *Tscherskia triton* | Farmland | Trap success | 1996 | 0.50 |
| 25 | Beijing | *Tscherskia triton* | Farmland | Trap success | 1997 | 0.41 |
| 26 | Beijing | *Tscherskia triton* | Farmland | Trap success | 1998 | 0.98 |
| 27 | Beijing | *Tscherskia triton* | Farmland | Trap success | 1999 | 1.00 |
| 28 | Beijing | *Tscherskia triton* | Farmland | Trap success | 2000 | 0.27 |
| 29 | Beijing | *Tscherskia triton* | Farmland | Trap success | 2001 | 0.24 |
| 30 | Beijing | *Tscherskia triton* | Farmland | Trap success | 2002 | 0.18 |
| 31 | Beijing | *Tscherskia triton* | Farmland | Trap success | 2003 | 0.19 |
| 32 | Beijing | *Tscherskia triton* | Farmland | Trap success | 2004 | 0.12 |
| 33 | Beijing | *Tscherskia triton* | Farmland | Trap success | 2005 | 0.04 |
| 34 | Beijing | *Tscherskia triton* | Farmland | Trap success | 2006 | 0.01 |
| 35 | Beijing | *Tscherskia triton* | Farmland | Trap success | 2007 | 0.00 |
| 36 | Beijing | *Tscherskia triton* | Farmland | Trap success | 2008 | 0.00 |
| 37 | Beijing | *Tscherskia triton* | Farmland | Trap success | 2009 | 0.00 |
| 38 | Beijing | *Tscherskia triton* | Farmland | Trap success | 2010 | 0.00 |
| 39 | Beijing | *Tscherskia triton* | Farmland | Trap success | 2011 | 0.00 |
| 40 | Beijing | *Tscherskia triton* | Farmland | Trap success | 2012 | 0.00 |
| 41 | Beijing | *Tscherskia triton* | Farmland | Trap success | 2013 | 0.00 |
| 42 | Beijing | *Tscherskia triton* | Farmland | Trap success | 2014 | 0.00 |
| 43 | Beijing | *Apodemus agrarius* | Forest | Trap success | 1993 | 0.04 |
| 44 | Beijing | *Apodemus agrarius* | Forest | Trap success | 1995 | 0.00 |
| 45 | Beijing | *Apodemus agrarius* | Forest | Trap success | 1996 | 0.39 |
| 46 | Beijing | *Apodemus agrarius* | Forest | Trap success | 1997 | 0.79 |
| 47 | Beijing | *Apodemus agrarius* | Forest | Trap success | 1998 | 0.04 |
| 48 | Beijing | *Apodemus agrarius* | Forest | Trap success | 1999 | 0.30 |
| 49 | Beijing | *Apodemus agrarius* | Forest | Trap success | 2000 | 0.33 |
| 50 | Beijing | *Apodemus agrarius* | Forest | Trap success | 2001 | 1.00 |
| 51 | Beijing | *Apodemus agrarius* | Forest | Trap success | 2002 | 0.12 |
| 52 | Beijing | *Apodemus agrarius* | Forest | Trap success | 2003 | 0.00 |
| 53 | Beijing | *Apodemus agrarius* | Forest | Trap success | 2004 | 0.00 |
| 54 | Beijing | *Apodemus agrarius* | Forest | Trap success | 2005 | 0.05 |
| 55 | Beijing | *Apodemus agrarius* | Forest | Trap success | 2006 | 0.01 |
| 56 | Beijing | *Apodemus agrarius* | Forest | Trap success | 2007 | 0.03 |
| 57 | Beijing | *Apodemus agrarius* | Forest | Trap success | 2008 | 0.04 |
| 58 | Beijing | *Apodemus agrarius* | Forest | Trap success | 2009 | 0.02 |
| 59 | Beijing | *Apodemus agrarius* | Forest | Trap success | 2010 | 0.00 |
| 60 | Beijing | *Apodemus agrarius* | Forest | Trap success | 2011 | 0.11 |
| 61 | Beijing | *Apodemus agrarius* | Forest | Trap success | 2012 | 0.00 |
| 62 | Beijing | *Apodemus agrarius* | Forest | Trap success | 2013 | 0.02 |
| 63 | Beijing | *Apodemus agrarius* | Forest | Trap success | 2014 | 0.05 |
| 64 | Beijing | *Apodemus agrarius* | Forest | Trap success | 2015 | 0.00 |
| 65 | Beijing | *Apodemus agrarius* | Forest | Trap success | 2016 | 0.04 |
| 66 | Beijing | *Apodemus agrarius* | Forest | Trap success | 2017 | 0.08 |
| 67 | Beijing | *Apodemus peninsulae* | Forest | Trap success | 1993 | 0.45 |
| 68 | Beijing | *Apodemus peninsulae* | Forest | Trap success | 1995 | 0.30 |
| 69 | Beijing | *Apodemus peninsulae* | Forest | Trap success | 1996 | 0.22 |
| 70 | Beijing | *Apodemus peninsulae* | Forest | Trap success | 1997 | 0.07 |
| 71 | Beijing | *Apodemus peninsulae* | Forest | Trap success | 1998 | 0.14 |
| 72 | Beijing | *Apodemus peninsulae* | Forest | Trap success | 1999 | 0.11 |
| 73 | Beijing | *Apodemus peninsulae* | Forest | Trap success | 2000 | 0.16 |
| 74 | Beijing | *Apodemus peninsulae* | Forest | Trap success | 2001 | 0.38 |
| 75 | Beijing | *Apodemus peninsulae* | Forest | Trap success | 2002 | 0.26 |
| 76 | Beijing | *Apodemus peninsulae* | Forest | Trap success | 2003 | 0.32 |
| 77 | Beijing | *Apodemus peninsulae* | Forest | Trap success | 2004 | 0.10 |
| 78 | Beijing | *Apodemus peninsulae* | Forest | Trap success | 2005 | 0.11 |
| 79 | Beijing | *Apodemus peninsulae* | Forest | Trap success | 2006 | 0.67 |
| 80 | Beijing | *Apodemus peninsulae* | Forest | Trap success | 2007 | 0.28 |
| 81 | Beijing | *Apodemus peninsulae* | Forest | Trap success | 2008 | 0.00 |
| 82 | Beijing | *Apodemus peninsulae* | Forest | Trap success | 2009 | 0.60 |
| 83 | Beijing | *Apodemus peninsulae* | Forest | Trap success | 2010 | 0.32 |
| 84 | Beijing | *Apodemus peninsulae* | Forest | Trap success | 2011 | 1.00 |
| 85 | Beijing | *Apodemus peninsulae* | Forest | Trap success | 2012 | 0.12 |
| 86 | Beijing | *Apodemus peninsulae* | Forest | Trap success | 2013 | 0.30 |
| 87 | Beijing | *Apodemus peninsulae* | Forest | Trap success | 2014 | 0.27 |
| 88 | Beijing | *Apodemus peninsulae* | Forest | Trap success | 2015 | 0.14 |
| 89 | Beijing | *Apodemus peninsulae* | Forest | Trap success | 2016 | 0.01 |
| 90 | Beijing | *Apodemus peninsulae* | Forest | Trap success | 2017 | 0.20 |
| 91 | Beijing | *Niviventer confucianus* | Forest | Trap success | 1993 | 0.42 |
| 92 | Beijing | *Niviventer confucianus* | Forest | Trap success | 1995 | 1.00 |
| 93 | Beijing | *Niviventer confucianus* | Forest | Trap success | 1996 | 0.44 |
| 94 | Beijing | *Niviventer confucianus* | Forest | Trap success | 1997 | 0.18 |
| 95 | Beijing | *Niviventer confucianus* | Forest | Trap success | 1998 | 0.06 |
| 96 | Beijing | *Niviventer confucianus* | Forest | Trap success | 1999 | 0.26 |
| 97 | Beijing | *Niviventer confucianus* | Forest | Trap success | 2000 | 0.29 |
| 98 | Beijing | *Niviventer confucianus* | Forest | Trap success | 2001 | 0.53 |
| 99 | Beijing | *Niviventer confucianus* | Forest | Trap success | 2002 | 0.18 |
| 100 | Beijing | *Niviventer confucianus* | Forest | Trap success | 2003 | 0.11 |
| 101 | Beijing | *Niviventer confucianus* | Forest | Trap success | 2004 | 0.11 |
| 102 | Beijing | *Niviventer confucianus* | Forest | Trap success | 2005 | 0.11 |
| 103 | Beijing | *Niviventer confucianus* | Forest | Trap success | 2006 | 0.23 |
| 104 | Beijing | *Niviventer confucianus* | Forest | Trap success | 2007 | 0.14 |
| 105 | Beijing | *Niviventer confucianus* | Forest | Trap success | 2008 | 0.13 |
| 106 | Beijing | *Niviventer confucianus* | Forest | Trap success | 2009 | 0.08 |
| 107 | Beijing | *Niviventer confucianus* | Forest | Trap success | 2010 | 0.03 |
| 108 | Beijing | *Niviventer confucianus* | Forest | Trap success | 2011 | 0.42 |
| 109 | Beijing | *Niviventer confucianus* | Forest | Trap success | 2012 | 0.00 |
| 110 | Beijing | *Niviventer confucianus* | Forest | Trap success | 2013 | 0.16 |
| 111 | Beijing | *Niviventer confucianus* | Forest | Trap success | 2014 | 0.06 |
| 112 | Beijing | *Niviventer confucianus* | Forest | Trap success | 2015 | 0.30 |
| 113 | Beijing | *Niviventer confucianus* | Forest | Trap success | 2016 | 0.06 |
| 114 | Beijing | *Niviventer confucianus* | Forest | Trap success | 2017 | 0.17 |
| 115 | Beijing | *Tscherskia triton* | Forest | Trap success | 1993 | 0.11 |
| 116 | Beijing | *Tscherskia triton* | Forest | Trap success | 1995 | 0.25 |
| 117 | Beijing | *Tscherskia triton* | Forest | Trap success | 1996 | 0.06 |
| 118 | Beijing | *Tscherskia triton* | Forest | Trap success | 1997 | 0.49 |
| 119 | Beijing | *Tscherskia triton* | Forest | Trap success | 1998 | 0.36 |
| 120 | Beijing | *Tscherskia triton* | Forest | Trap success | 1999 | 0.00 |
| 121 | Beijing | *Tscherskia triton* | Forest | Trap success | 2000 | 0.00 |
| 122 | Beijing | *Tscherskia triton* | Forest | Trap success | 2001 | 0.12 |
| 123 | Beijing | *Tscherskia triton* | Forest | Trap success | 2002 | 1.00 |
| 124 | Beijing | *Tscherskia triton* | Forest | Trap success | 2003 | 0.03 |
| 125 | Beijing | *Tscherskia triton* | Forest | Trap success | 2004 | 0.00 |
| 126 | Beijing | *Tscherskia triton* | Forest | Trap success | 2005 | 0.00 |
| 127 | Beijing | *Tscherskia triton* | Forest | Trap success | 2006 | 0.18 |
| 128 | Beijing | *Tscherskia triton* | Forest | Trap success | 2007 | 0.03 |
| 129 | Beijing | *Tscherskia triton* | Forest | Trap success | 2008 | 0.00 |
| 130 | Beijing | *Tscherskia triton* | Forest | Trap success | 2009 | 0.00 |
| 131 | Beijing | *Tscherskia triton* | Forest | Trap success | 2010 | 0.00 |
| 132 | Beijing | *Tscherskia triton* | Forest | Trap success | 2011 | 0.00 |
| 133 | Beijing | *Tscherskia triton* | Forest | Trap success | 2012 | 0.12 |
| 134 | Beijing | *Tscherskia triton* | Forest | Trap success | 2013 | 0.03 |
| 135 | Beijing | *Tscherskia triton* | Forest | Trap success | 2014 | 0.00 |
| 136 | Beijing | *Tscherskia triton* | Forest | Trap success | 2015 | 0.06 |
| 137 | Beijing | *Tscherskia triton* | Forest | Trap success | 2016 | 0.12 |
| 138 | Beijing | *Tscherskia triton* | Forest | Trap success | 2017 | 0.00 |
| 139 | Fujian Province | *Rattus losea* | City | Trap success | 1985 | 0.24 |
| 140 | Fujian Province | *Rattus losea* | City | Trap success | 1986 | 0.76 |
| 141 | Fujian Province | *Rattus losea* | City | Trap success | 1987 | 0.28 |
| 142 | Fujian Province | *Rattus losea* | City | Trap success | 1988 | 0.79 |
| 143 | Fujian Province | *Rattus losea* | City | Trap success | 1989 | 1.00 |
| 144 | Fujian Province | *Rattus losea* | City | Trap success | 1990 | 0.97 |
| 145 | Fujian Province | *Rattus losea* | City | Trap success | 1991 | 0.73 |
| 146 | Fujian Province | *Rattus losea* | City | Trap success | 1992 | 0.63 |
| 147 | Fujian Province | *Rattus losea* | City | Trap success | 1993 | 0.66 |
| 148 | Fujian Province | *Rattus losea* | City | Trap success | 1994 | 0.67 |
| 149 | Fujian Province | *Rattus losea* | City | Trap success | 1995 | 0.85 |
| 150 | Fujian Province | *Rattus losea* | City | Trap success | 1996 | 0.49 |
| 151 | Fujian Province | *Rattus losea* | City | Trap success | 1997 | 0.77 |
| 152 | Fujian Province | *Rattus losea* | City | Trap success | 1998 | 0.94 |
| 153 | Fujian Province | *Rattus losea* | City | Trap success | 1999 | 0.34 |
| 154 | Fujian Province | *Rattus losea* | City | Trap success | 2000 | 0.23 |
| 155 | Fujian Province | *Rattus losea* | City | Trap success | 2001 | 0.06 |
| 156 | Fujian Province | *Rattus losea* | City | Trap success | 2002 | 0.01 |
| 157 | Fujian Province | *Rattus losea* | City | Trap success | 2003 | 0.00 |
| 158 | Fujian Province | *Rattus losea* | City | Trap success | 2004 | 0.01 |
| 159 | Fujian Province | *Rattus norvegicus* | City | Trap success | 1985 | 0.90 |
| 160 | Fujian Province | *Rattus norvegicus* | City | Trap success | 1986 | 0.54 |
| 161 | Fujian Province | *Rattus norvegicus* | City | Trap success | 1987 | 0.60 |
| 162 | Fujian Province | *Rattus norvegicus* | City | Trap success | 1988 | 0.29 |
| 163 | Fujian Province | *Rattus norvegicus* | City | Trap success | 1989 | 0.20 |
| 164 | Fujian Province | *Rattus norvegicus* | City | Trap success | 1990 | 0.30 |
| 165 | Fujian Province | *Rattus norvegicus* | City | Trap success | 1991 | 0.43 |
| 166 | Fujian Province | *Rattus norvegicus* | City | Trap success | 1992 | 0.62 |
| 167 | Fujian Province | *Rattus norvegicus* | City | Trap success | 1993 | 0.80 |
| 168 | Fujian Province | *Rattus norvegicus* | City | Trap success | 1994 | 0.90 |
| 169 | Fujian Province | *Rattus norvegicus* | City | Trap success | 1995 | 0.67 |
| 170 | Fujian Province | *Rattus norvegicus* | City | Trap success | 1996 | 1.00 |
| 171 | Fujian Province | *Rattus norvegicus* | City | Trap success | 1997 | 0.59 |
| 172 | Fujian Province | *Rattus norvegicus* | City | Trap success | 1998 | 0.44 |
| 173 | Fujian Province | *Rattus norvegicus* | City | Trap success | 1999 | 0.36 |
| 174 | Fujian Province | *Rattus norvegicus* | City | Trap success | 2000 | 0.49 |
| 175 | Fujian Province | *Rattus norvegicus* | City | Trap success | 2001 | 0.23 |
| 176 | Fujian Province | *Rattus norvegicus* | City | Trap success | 2002 | 0.12 |
| 177 | Fujian Province | *Rattus norvegicus* | City | Trap success | 2003 | 0.00 |
| 178 | Fujian Province | *Rattus norvegicus* | City | Trap success | 2004 | 0.05 |
| 179 | Fujian Province | *Rattus tanezumi* | City | Trap success | 1985 | 0.80 |
| 180 | Fujian Province | *Rattus tanezumi* | City | Trap success | 1986 | 1.00 |
| 181 | Fujian Province | *Rattus tanezumi* | City | Trap success | 1987 | 0.93 |
| 182 | Fujian Province | *Rattus tanezumi* | City | Trap success | 1988 | 0.84 |
| 183 | Fujian Province | *Rattus tanezumi* | City | Trap success | 1989 | 0.58 |
| 184 | Fujian Province | *Rattus tanezumi* | City | Trap success | 1990 | 0.71 |
| 185 | Fujian Province | *Rattus tanezumi* | City | Trap success | 1991 | 0.34 |
| 186 | Fujian Province | *Rattus tanezumi* | City | Trap success | 1992 | 0.20 |
| 187 | Fujian Province | *Rattus tanezumi* | City | Trap success | 1993 | 0.07 |
| 188 | Fujian Province | *Rattus tanezumi* | City | Trap success | 1994 | 0.00 |
| 189 | Fujian Province | *Rattus tanezumi* | City | Trap success | 1995 | 0.04 |
| 190 | Fujian Province | *Rattus tanezumi* | City | Trap success | 1996 | 0.11 |
| 191 | Fujian Province | *Rattus tanezumi* | City | Trap success | 1997 | 0.04 |
| 192 | Fujian Province | *Rattus tanezumi* | City | Trap success | 1998 | 0.06 |
| 193 | Fujian Province | *Rattus tanezumi* | City | Trap success | 1999 | 0.00 |
| 194 | Fujian Province | *Rattus tanezumi* | City | Trap success | 2000 | 0.04 |
| 195 | Fujian Province | *Rattus tanezumi* | City | Trap success | 2001 | 0.15 |
| 196 | Fujian Province | *Rattus tanezumi* | City | Trap success | 2002 | 0.13 |
| 197 | Fujian Province | *Rattus tanezumi* | City | Trap success | 2003 | 0.11 |
| 198 | Fujian Province | *Rattus tanezumi* | City | Trap success | 2004 | 0.15 |
| 199 | Gansu province | *Spermophilus alaschanicus* | Grassland | Population density | 1980 | 0.41 |
| 200 | Gansu province | *Spermophilus alaschanicus* | Grassland | Population density | 1981 | 0.35 |
| 201 | Gansu province | *Spermophilus alaschanicus* | Grassland | Population density | 1982 | 0.10 |
| 202 | Gansu province | *Spermophilus alaschanicus* | Grassland | Population density | 1983 | 0.03 |
| 203 | Gansu province | *Spermophilus alaschanicus* | Grassland | Population density | 1984 | 0.08 |
| 204 | Gansu province | *Spermophilus alaschanicus* | Grassland | Population density | 1985 | 0.09 |
| 205 | Gansu province | *Spermophilus alaschanicus* | Grassland | Population density | 1986 | 0.12 |
| 206 | Gansu province | *Spermophilus alaschanicus* | Grassland | Population density | 1987 | 0.09 |
| 207 | Gansu province | *Spermophilus alaschanicus* | Grassland | Population density | 1988 | 0.28 |
| 208 | Gansu province | *Spermophilus alaschanicus* | Grassland | Population density | 1989 | 0.28 |
| 209 | Gansu province | *Spermophilus alaschanicus* | Grassland | Population density | 1990 | 0.33 |
| 210 | Gansu province | *Spermophilus alaschanicus* | Grassland | Population density | 1991 | 0.43 |
| 211 | Gansu province | *Spermophilus alaschanicus* | Grassland | Population density | 1992 | 0.44 |
| 212 | Gansu province | *Spermophilus alaschanicus* | Grassland | Population density | 1993 | 0.31 |
| 213 | Gansu province | *Spermophilus alaschanicus* | Grassland | Population density | 1994 | 0.64 |
| 214 | Gansu province | *Spermophilus alaschanicus* | Grassland | Population density | 1995 | 0.92 |
| 215 | Gansu province | *Spermophilus alaschanicus* | Grassland | Population density | 1996 | 0.97 |
| 216 | Gansu province | *Spermophilus alaschanicus* | Grassland | Population density | 1997 | 0.69 |
| 217 | Gansu province | *Spermophilus alaschanicus* | Grassland | Population density | 1998 | 1.00 |
| 218 | Gansu province | *Spermophilus alaschanicus* | Grassland | Population density | 1999 | 0.78 |
| 219 | Gansu province | *Spermophilus alaschanicus* | Grassland | Population density | 2000 | 0.92 |
| 220 | Gansu province | *Spermophilus alaschanicus* | Grassland | Population density | 2001 | 1.00 |
| 221 | Gansu province | *Spermophilus alaschanicus* | Grassland | Population density | 2002 | 0.78 |
| 222 | Gansu province | *Spermophilus alaschanicus* | Grassland | Population density | 2003 | 0.92 |
| 223 | Gansu province | *Spermophilus alaschanicus* | Grassland | Population density | 2005 | 0.46 |
| 224 | Gansu province | *Spermophilus alaschanicus* | Grassland | Population density | 2006 | 0.34 |
| 225 | Gansu province | *Spermophilus alaschanicus* | Grassland | Population density | 2007 | 0.25 |
| 226 | Gansu province | *Spermophilus alaschanicus* | Grassland | Population density | 2008 | 0.00 |
| 227 | Guangxi Zhuang Autonomous Region | *Bandicota indica* | City | Trap success | 1987 | 0.15 |
| 228 | Guangxi Zhuang Autonomous Region | *Bandicota indica* | City | Trap success | 1988 | 0.08 |
| 229 | Guangxi Zhuang Autonomous Region | *Bandicota indica* | City | Trap success | 1989 | 0.00 |
| 230 | Guangxi Zhuang Autonomous Region | *Bandicota indica* | City | Trap success | 1990 | 0.10 |
| 231 | Guangxi Zhuang Autonomous Region | *Bandicota indica* | City | Trap success | 1991 | 0.11 |
| 232 | Guangxi Zhuang Autonomous Region | *Bandicota indica* | City | Trap success | 1992 | 0.09 |
| 233 | Guangxi Zhuang Autonomous Region | *Bandicota indica* | City | Trap success | 1993 | 0.19 |
| 234 | Guangxi Zhuang Autonomous Region | *Bandicota indica* | City | Trap success | 1994 | 0.24 |
| 235 | Guangxi Zhuang Autonomous Region | *Bandicota indica* | City | Trap success | 1995 | 0.41 |
| 236 | Guangxi Zhuang Autonomous Region | *Bandicota indica* | City | Trap success | 1996 | 0.46 |
| 237 | Guangxi Zhuang Autonomous Region | *Bandicota indica* | City | Trap success | 1997 | 1.00 |
| 238 | Guangxi Zhuang Autonomous Region | *Bandicota indica* | City | Trap success | 1998 | 0.62 |
| 239 | Guangxi Zhuang Autonomous Region | *Bandicota indica* | City | Trap success | 1999 | 0.99 |
| 240 | Guangxi Zhuang Autonomous Region | *Bandicota indica* | City | Trap success | 2000 | 0.84 |
| 241 | Guangxi Zhuang Autonomous Region | *Bandicota indica* | City | Trap success | 2001 | 0.74 |
| 242 | Guangxi Zhuang Autonomous Region | *Bandicota indica* | City | Trap success | 2002 | 0.69 |
| 243 | Guangxi Zhuang Autonomous Region | *Bandicota indica* | City | Trap success | 2003 | 0.65 |
| 244 | Guangxi Zhuang Autonomous Region | *Bandicota indica* | City | Trap success | 2004 | 0.63 |
| 245 | Guangxi Zhuang Autonomous Region | *Bandicota indica* | City | Trap success | 2005 | 0.72 |
| 246 | Guangxi Zhuang Autonomous Region | *Bandicota indica* | City | Trap success | 2006 | 0.72 |
| 247 | Guangxi Zhuang Autonomous Region | *Rattus losea* | City | Trap success | 1987 | 0.92 |
| 248 | Guangxi Zhuang Autonomous Region | *Rattus losea* | City | Trap success | 1988 | 0.95 |
| 249 | Guangxi Zhuang Autonomous Region | *Rattus losea* | City | Trap success | 1989 | 0.89 |
| 250 | Guangxi Zhuang Autonomous Region | *Rattus losea* | City | Trap success | 1990 | 0.90 |
| 251 | Guangxi Zhuang Autonomous Region | *Rattus losea* | City | Trap success | 1991 | 1.00 |
| 252 | Guangxi Zhuang Autonomous Region | *Rattus losea* | City | Trap success | 1992 | 0.99 |
| 253 | Guangxi Zhuang Autonomous Region | *Rattus losea* | City | Trap success | 1993 | 0.90 |
| 254 | Guangxi Zhuang Autonomous Region | *Rattus losea* | City | Trap success | 1994 | 0.90 |
| 255 | Guangxi Zhuang Autonomous Region | *Rattus losea* | City | Trap success | 1995 | 0.65 |
| 256 | Guangxi Zhuang Autonomous Region | *Rattus losea* | City | Trap success | 1996 | 0.35 |
| 257 | Guangxi Zhuang Autonomous Region | *Rattus losea* | City | Trap success | 1997 | 0.40 |
| 258 | Guangxi Zhuang Autonomous Region | *Rattus losea* | City | Trap success | 1998 | 0.11 |
| 259 | Guangxi Zhuang Autonomous Region | *Rattus losea* | City | Trap success | 1999 | 0.28 |
| 260 | Guangxi Zhuang Autonomous Region | *Rattus losea* | City | Trap success | 2000 | 0.22 |
| 261 | Guangxi Zhuang Autonomous Region | *Rattus losea* | City | Trap success | 2001 | 0.17 |
| 262 | Guangxi Zhuang Autonomous Region | *Rattus losea* | City | Trap success | 2002 | 0.14 |
| 263 | Guangxi Zhuang Autonomous Region | *Rattus losea* | City | Trap success | 2003 | 0.12 |
| 264 | Guangxi Zhuang Autonomous Region | *Rattus losea* | City | Trap success | 2004 | 0.04 |
| 265 | Guangxi Zhuang Autonomous Region | *Rattus losea* | City | Trap success | 2005 | 0.00 |
| 266 | Guangxi Zhuang Autonomous Region | *Rattus losea* | City | Trap success | 2006 | 0.00 |
| 267 | Guangxi Zhuang Autonomous Region | *Rattus norvegicus* | City | Trap success | 1987 | 0.33 |
| 268 | Guangxi Zhuang Autonomous Region | *Rattus norvegicus* | City | Trap success | 1988 | 0.25 |
| 269 | Guangxi Zhuang Autonomous Region | *Rattus norvegicus* | City | Trap success | 1989 | 0.41 |
| 270 | Guangxi Zhuang Autonomous Region | *Rattus norvegicus* | City | Trap success | 1990 | 0.16 |
| 271 | Guangxi Zhuang Autonomous Region | *Rattus norvegicus* | City | Trap success | 1991 | 0.32 |
| 272 | Guangxi Zhuang Autonomous Region | *Rattus norvegicus* | City | Trap success | 1992 | 0.54 |
| 273 | Guangxi Zhuang Autonomous Region | *Rattus norvegicus* | City | Trap success | 1993 | 0.06 |
| 274 | Guangxi Zhuang Autonomous Region | *Rattus norvegicus* | City | Trap success | 1994 | 1.00 |
| 275 | Guangxi Zhuang Autonomous Region | *Rattus norvegicus* | City | Trap success | 1995 | 0.40 |
| 276 | Guangxi Zhuang Autonomous Region | *Rattus norvegicus* | City | Trap success | 1996 | 0.08 |
| 277 | Guangxi Zhuang Autonomous Region | *Rattus norvegicus* | City | Trap success | 1997 | 0.16 |
| 278 | Guangxi Zhuang Autonomous Region | *Rattus norvegicus* | City | Trap success | 1998 | 0.00 |
| 279 | Guangxi Zhuang Autonomous Region | *Rattus norvegicus* | City | Trap success | 1999 | 0.10 |
| 280 | Guangxi Zhuang Autonomous Region | *Rattus norvegicus* | City | Trap success | 2000 | 0.43 |
| 281 | Guangxi Zhuang Autonomous Region | *Rattus norvegicus* | City | Trap success | 2001 | 0.25 |
| 282 | Guangxi Zhuang Autonomous Region | *Rattus norvegicus* | City | Trap success | 2002 | 0.13 |
| 283 | Guangxi Zhuang Autonomous Region | *Rattus norvegicus* | City | Trap success | 2003 | 0.07 |
| 284 | Guangxi Zhuang Autonomous Region | *Rattus norvegicus* | City | Trap success | 2004 | 0.38 |
| 285 | Guangxi Zhuang Autonomous Region | *Rattus norvegicus* | City | Trap success | 2005 | 0.27 |
| 286 | Guangxi Zhuang Autonomous Region | *Rattus norvegicus* | City | Trap success | 2006 | 0.09 |
| 287 | Guangxi Zhuang Autonomous Region | *Rattus tanezumi* | City | Trap success | 1987 | 0.43 |
| 288 | Guangxi Zhuang Autonomous Region | *Rattus tanezumi* | City | Trap success | 1988 | 0.08 |
| 289 | Guangxi Zhuang Autonomous Region | *Rattus tanezumi* | City | Trap success | 1989 | 0.01 |
| 290 | Guangxi Zhuang Autonomous Region | *Rattus tanezumi* | City | Trap success | 1990 | 0.10 |
| 291 | Guangxi Zhuang Autonomous Region | *Rattus tanezumi* | City | Trap success | 1991 | 0.34 |
| 292 | Guangxi Zhuang Autonomous Region | *Rattus tanezumi* | City | Trap success | 1992 | 0.32 |
| 293 | Guangxi Zhuang Autonomous Region | *Rattus tanezumi* | City | Trap success | 1993 | 1.00 |
| 294 | Guangxi Zhuang Autonomous Region | *Rattus tanezumi* | City | Trap success | 1994 | 0.38 |
| 295 | Guangxi Zhuang Autonomous Region | *Rattus tanezumi* | City | Trap success | 1995 | 0.46 |
| 296 | Guangxi Zhuang Autonomous Region | *Rattus tanezumi* | City | Trap success | 1996 | 0.48 |
| 297 | Guangxi Zhuang Autonomous Region | *Rattus tanezumi* | City | Trap success | 1997 | 0.37 |
| 298 | Guangxi Zhuang Autonomous Region | *Rattus tanezumi* | City | Trap success | 1998 | 0.21 |
| 299 | Guangxi Zhuang Autonomous Region | *Rattus tanezumi* | City | Trap success | 1999 | 0.38 |
| 300 | Guangxi Zhuang Autonomous Region | *Rattus tanezumi* | City | Trap success | 2000 | 0.20 |
| 301 | Guangxi Zhuang Autonomous Region | *Rattus tanezumi* | City | Trap success | 2001 | 0.15 |
| 302 | Guangxi Zhuang Autonomous Region | *Rattus tanezumi* | City | Trap success | 2002 | 0.12 |
| 303 | Guangxi Zhuang Autonomous Region | *Rattus tanezumi* | City | Trap success | 2003 | 0.05 |
| 304 | Guangxi Zhuang Autonomous Region | *Rattus tanezumi* | City | Trap success | 2004 | 0.00 |
| 305 | Guangxi Zhuang Autonomous Region | *Rattus tanezumi* | City | Trap success | 2005 | 0.04 |
| 306 | Guangxi Zhuang Autonomous Region | *Rattus tanezumi* | City | Trap success | 2006 | 0.02 |
| 307 | Guizhou Province | *Mus musculus* | Farmland | Trap success | 1984 | 0.00 |
| 308 | Guizhou Province | *Mus musculus* | Farmland | Trap success | 1985 | 0.00 |
| 309 | Guizhou Province | *Mus musculus* | Farmland | Trap success | 1986 | 0.09 |
| 310 | Guizhou Province | *Mus musculus* | Farmland | Trap success | 1987 | 1.00 |
| 311 | Guizhou Province | *Mus musculus* | Farmland | Trap success | 1988 | 0.09 |
| 312 | Guizhou Province | *Mus musculus* | Farmland | Trap success | 1989 | 0.09 |
| 313 | Guizhou Province | *Mus musculus* | Farmland | Trap success | 1990 | 0.04 |
| 314 | Guizhou Province | *Mus musculus* | Farmland | Trap success | 1991 | 0.09 |
| 315 | Guizhou Province | *Mus musculus* | Farmland | Trap success | 1992 | 0.21 |
| 316 | Guizhou Province | *Mus musculus* | Farmland | Trap success | 1993 | 0.26 |
| 317 | Guizhou Province | *Mus musculus* | Farmland | Trap success | 1994 | 0.06 |
| 318 | Guizhou Province | *Mus musculus* | Farmland | Trap success | 1995 | 0.57 |
| 319 | Guizhou Province | *Mus musculus* | Farmland | Trap success | 1996 | 0.45 |
| 320 | Guizhou Province | *Mus musculus* | Farmland | Trap success | 1997 | 0.51 |
| 321 | Guizhou Province | *Mus musculus* | Farmland | Trap success | 1998 | 0.49 |
| 322 | Guizhou Province | *Mus musculus* | Farmland | Trap success | 1999 | 0.13 |
| 323 | Guizhou Province | *Mus musculus* | Farmland | Trap success | 2000 | 0.09 |
| 324 | Guizhou Province | *Mus musculus* | Farmland | Trap success | 2001 | 0.00 |
| 325 | Guizhou Province | *Mus musculus* | Farmland | Trap success | 2002 | 0.21 |
| 326 | Guizhou Province | *Mus musculus* | Farmland | Trap success | 2003 | 0.00 |
| 327 | Guizhou Province | *Mus musculus* | Farmland | Trap success | 2005 | 0.13 |
| 328 | Guizhou Province | *Mus musculus* | Farmland | Trap success | 2006 | 0.00 |
| 329 | Guizhou Province | *Mus musculus* | Farmland | Trap success | 2007 | 0.49 |
| 330 | Guizhou Province | *Mus musculus* | Farmland | Trap success | 2008 | 0.04 |
| 331 | Guizhou Province | *Mus musculus* | Farmland | Trap success | 2009 | 0.04 |
| 332 | Guizhou Province | *Rattus norvegicus* | City | Trap success | 1984 | 0.00 |
| 333 | Guizhou Province | *Rattus norvegicus* | City | Trap success | 1985 | 0.26 |
| 334 | Guizhou Province | *Rattus norvegicus* | City | Trap success | 1986 | 1.00 |
| 335 | Guizhou Province | *Rattus norvegicus* | City | Trap success | 1987 | 0.67 |
| 336 | Guizhou Province | *Rattus norvegicus* | City | Trap success | 1988 | 0.51 |
| 337 | Guizhou Province | *Rattus norvegicus* | City | Trap success | 1989 | 0.21 |
| 338 | Guizhou Province | *Rattus norvegicus* | City | Trap success | 1990 | 0.31 |
| 339 | Guizhou Province | *Rattus norvegicus* | City | Trap success | 1991 | 0.24 |
| 340 | Guizhou Province | *Rattus norvegicus* | City | Trap success | 1992 | 0.32 |
| 341 | Guizhou Province | *Rattus norvegicus* | City | Trap success | 1993 | 0.40 |
| 342 | Guizhou Province | *Rattus norvegicus* | City | Trap success | 1994 | 0.53 |
| 343 | Guizhou Province | *Rattus norvegicus* | City | Trap success | 1995 | 0.55 |
| 344 | Guizhou Province | *Rattus norvegicus* | City | Trap success | 1996 | 0.38 |
| 345 | Guizhou Province | *Rattus norvegicus* | City | Trap success | 1997 | 0.42 |
| 346 | Guizhou Province | *Rattus norvegicus* | City | Trap success | 1998 | 0.49 |
| 347 | Guizhou Province | *Rattus norvegicus* | City | Trap success | 1999 | 0.33 |
| 348 | Guizhou Province | *Rattus norvegicus* | City | Trap success | 2000 | 0.27 |
| 349 | Guizhou Province | *Rattus norvegicus* | City | Trap success | 2001 | 0.22 |
| 350 | Guizhou Province | *Rattus norvegicus* | City | Trap success | 2002 | 0.19 |
| 351 | Guizhou Province | *Rattus norvegicus* | City | Trap success | 2003 | 0.20 |
| 352 | Guizhou Province | *Rattus norvegicus* | City | Trap success | 2005 | 0.09 |
| 353 | Guizhou Province | *Rattus norvegicus* | City | Trap success | 2006 | 0.06 |
| 354 | Guizhou Province | *Rattus norvegicus* | City | Trap success | 2007 | 0.05 |
| 355 | Guizhou Province | *Rattus norvegicus* | City | Trap success | 2008 | 0.05 |
| 356 | Guizhou Province | *Rattus norvegicus* | City | Trap success | 2009 | 0.09 |
| 357 | Guizhou Province | *Rattus norvegicus* | Farmland | Trap success | 1984 | 0.19 |
| 358 | Guizhou Province | *Rattus norvegicus* | Farmland | Trap success | 1985 | 0.47 |
| 359 | Guizhou Province | *Rattus norvegicus* | Farmland | Trap success | 1986 | 0.00 |
| 360 | Guizhou Province | *Rattus norvegicus* | Farmland | Trap success | 1987 | 0.00 |
| 361 | Guizhou Province | *Rattus norvegicus* | Farmland | Trap success | 1988 | 0.05 |
| 362 | Guizhou Province | *Rattus norvegicus* | Farmland | Trap success | 1989 | 0.21 |
| 363 | Guizhou Province | *Rattus norvegicus* | Farmland | Trap success | 1990 | 0.06 |
| 364 | Guizhou Province | *Rattus norvegicus* | Farmland | Trap success | 1991 | 0.09 |
| 365 | Guizhou Province | *Rattus norvegicus* | Farmland | Trap success | 1992 | 0.18 |
| 366 | Guizhou Province | *Rattus norvegicus* | Farmland | Trap success | 1993 | 0.13 |
| 367 | Guizhou Province | *Rattus norvegicus* | Farmland | Trap success | 1994 | 0.96 |
| 368 | Guizhou Province | *Rattus norvegicus* | Farmland | Trap success | 1995 | 0.85 |
| 369 | Guizhou Province | *Rattus norvegicus* | Farmland | Trap success | 1996 | 0.31 |
| 370 | Guizhou Province | *Rattus norvegicus* | Farmland | Trap success | 1997 | 0.51 |
| 371 | Guizhou Province | *Rattus norvegicus* | Farmland | Trap success | 1998 | 0.40 |
| 372 | Guizhou Province | *Rattus norvegicus* | Farmland | Trap success | 1999 | 0.42 |
| 373 | Guizhou Province | *Rattus norvegicus* | Farmland | Trap success | 2000 | 0.74 |
| 374 | Guizhou Province | *Rattus norvegicus* | Farmland | Trap success | 2001 | 1.00 |
| 375 | Guizhou Province | *Rattus norvegicus* | Farmland | Trap success | 2002 | 0.89 |
| 376 | Guizhou Province | *Rattus norvegicus* | Farmland | Trap success | 2003 | 0.75 |
| 377 | Guizhou Province | *Rattus norvegicus* | Farmland | Trap success | 2005 | 0.43 |
| 378 | Guizhou Province | *Rattus norvegicus* | Farmland | Trap success | 2006 | 0.28 |
| 379 | Guizhou Province | *Rattus norvegicus* | Farmland | Trap success | 2007 | 0.65 |
| 380 | Guizhou Province | *Rattus norvegicus* | Farmland | Trap success | 2008 | 0.49 |
| 381 | Guizhou Province | *Rattus norvegicus* | Farmland | Trap success | 2009 | 0.39 |
| 382 | Guizhou Province | *Rattus tanezumi* | City | Trap success | 1984 | 1.00 |
| 383 | Guizhou Province | *Rattus tanezumi* | City | Trap success | 1985 | 0.73 |
| 384 | Guizhou Province | *Rattus tanezumi* | City | Trap success | 1986 | 0.43 |
| 385 | Guizhou Province | *Rattus tanezumi* | City | Trap success | 1987 | 0.25 |
| 386 | Guizhou Province | *Rattus tanezumi* | City | Trap success | 1988 | 0.30 |
| 387 | Guizhou Province | *Rattus tanezumi* | City | Trap success | 1989 | 0.30 |
| 388 | Guizhou Province | *Rattus tanezumi* | City | Trap success | 1990 | 0.25 |
| 389 | Guizhou Province | *Rattus tanezumi* | City | Trap success | 1991 | 0.29 |
| 390 | Guizhou Province | *Rattus tanezumi* | City | Trap success | 1992 | 0.35 |
| 391 | Guizhou Province | *Rattus tanezumi* | City | Trap success | 1993 | 0.42 |
| 392 | Guizhou Province | *Rattus tanezumi* | City | Trap success | 1994 | 0.48 |
| 393 | Guizhou Province | *Rattus tanezumi* | City | Trap success | 1995 | 0.82 |
| 394 | Guizhou Province | *Rattus tanezumi* | City | Trap success | 1996 | 0.75 |
| 395 | Guizhou Province | *Rattus tanezumi* | City | Trap success | 1997 | 0.51 |
| 396 | Guizhou Province | *Rattus tanezumi* | City | Trap success | 1998 | 0.39 |
| 397 | Guizhou Province | *Rattus tanezumi* | City | Trap success | 1999 | 0.37 |
| 398 | Guizhou Province | *Rattus tanezumi* | City | Trap success | 2000 | 0.29 |
| 399 | Guizhou Province | *Rattus tanezumi* | City | Trap success | 2001 | 0.28 |
| 400 | Guizhou Province | *Rattus tanezumi* | City | Trap success | 2002 | 0.31 |
| 401 | Guizhou Province | *Rattus tanezumi* | City | Trap success | 2003 | 0.21 |
| 402 | Guizhou Province | *Rattus tanezumi* | City | Trap success | 2005 | 0.03 |
| 403 | Guizhou Province | *Rattus tanezumi* | City | Trap success | 2006 | 0.00 |
| 404 | Guizhou Province | *Rattus tanezumi* | City | Trap success | 2007 | 0.06 |
| 405 | Guizhou Province | *Rattus tanezumi* | City | Trap success | 2008 | 0.08 |
| 406 | Guizhou Province | *Rattus tanezumi* | City | Trap success | 2009 | 0.09 |
| 407 | Guizhou Province | *Rattus tanezumi* | Farmland | Trap success | 1984 | 0.88 |
| 408 | Guizhou Province | *Rattus tanezumi* | Farmland | Trap success | 1985 | 0.81 |
| 409 | Guizhou Province | *Rattus tanezumi* | Farmland | Trap success | 1986 | 0.11 |
| 410 | Guizhou Province | *Rattus tanezumi* | Farmland | Trap success | 1987 | 0.12 |
| 411 | Guizhou Province | *Rattus tanezumi* | Farmland | Trap success | 1988 | 0.00 |
| 412 | Guizhou Province | *Rattus tanezumi* | Farmland | Trap success | 1989 | 0.11 |
| 413 | Guizhou Province | *Rattus tanezumi* | Farmland | Trap success | 1990 | 0.06 |
| 414 | Guizhou Province | *Rattus tanezumi* | Farmland | Trap success | 1991 | 0.11 |
| 415 | Guizhou Province | *Rattus tanezumi* | Farmland | Trap success | 1992 | 0.22 |
| 416 | Guizhou Province | *Rattus tanezumi* | Farmland | Trap success | 1993 | 0.53 |
| 417 | Guizhou Province | *Rattus tanezumi* | Farmland | Trap success | 1994 | 1.00 |
| 418 | Guizhou Province | *Rattus tanezumi* | Farmland | Trap success | 1995 | 0.61 |
| 419 | Guizhou Province | *Rattus tanezumi* | Farmland | Trap success | 1996 | 0.76 |
| 420 | Guizhou Province | *Rattus tanezumi* | Farmland | Trap success | 1997 | 0.53 |
| 421 | Guizhou Province | *Rattus tanezumi* | Farmland | Trap success | 1998 | 0.37 |
| 422 | Guizhou Province | *Rattus tanezumi* | Farmland | Trap success | 1999 | 0.28 |
| 423 | Guizhou Province | *Rattus tanezumi* | Farmland | Trap success | 2000 | 0.51 |
| 424 | Guizhou Province | *Rattus tanezumi* | Farmland | Trap success | 2001 | 0.59 |
| 425 | Guizhou Province | *Rattus tanezumi* | Farmland | Trap success | 2002 | 0.68 |
| 426 | Guizhou Province | *Rattus tanezumi* | Farmland | Trap success | 2003 | 0.51 |
| 427 | Guizhou Province | *Rattus tanezumi* | Farmland | Trap success | 2005 | 0.21 |
| 428 | Guizhou Province | *Rattus tanezumi* | Farmland | Trap success | 2006 | 0.12 |
| 429 | Guizhou Province | *Rattus tanezumi* | Farmland | Trap success | 2007 | 0.33 |
| 430 | Guizhou Province | *Rattus tanezumi* | Farmland | Trap success | 2008 | 0.37 |
| 431 | Guizhou Province | *Rattus tanezumi* | Farmland | Trap success | 2009 | 0.18 |
| 432 | Guizhou Province | *Mus musculus* | City | Trap success | 1986 | 0.49 |
| 433 | Guizhou Province | *Mus musculus* | City | Trap success | 1987 | 0.47 |
| 434 | Guizhou Province | *Mus musculus* | City | Trap success | 1988 | 0.16 |
| 435 | Guizhou Province | *Mus musculus* | City | Trap success | 1989 | 0.27 |
| 436 | Guizhou Province | *Mus musculus* | City | Trap success | 1990 | 0.59 |
| 437 | Guizhou Province | *Mus musculus* | City | Trap success | 1991 | 1.00 |
| 438 | Guizhou Province | *Mus musculus* | City | Trap success | 1992 | 0.58 |
| 439 | Guizhou Province | *Mus musculus* | City | Trap success | 1993 | 0.85 |
| 440 | Guizhou Province | *Mus musculus* | City | Trap success | 1994 | 0.52 |
| 441 | Guizhou Province | *Mus musculus* | City | Trap success | 1995 | 0.56 |
| 442 | Guizhou Province | *Mus musculus* | City | Trap success | 1996 | 0.48 |
| 443 | Guizhou Province | *Mus musculus* | City | Trap success | 1997 | 0.38 |
| 444 | Guizhou Province | *Mus musculus* | City | Trap success | 1998 | 0.33 |
| 445 | Guizhou Province | *Mus musculus* | City | Trap success | 1999 | 0.31 |
| 446 | Guizhou Province | *Mus musculus* | City | Trap success | 2000 | 0.25 |
| 447 | Guizhou Province | *Mus musculus* | City | Trap success | 2001 | 0.30 |
| 448 | Guizhou Province | *Mus musculus* | City | Trap success | 2002 | 0.24 |
| 449 | Guizhou Province | *Mus musculus* | City | Trap success | 2003 | 0.10 |
| 450 | Guizhou Province | *Mus musculus* | City | Trap success | 2004 | 0.06 |
| 451 | Guizhou Province | *Mus musculus* | City | Trap success | 2005 | 0.20 |
| 452 | Guizhou Province | *Mus musculus* | City | Trap success | 2006 | 0.07 |
| 453 | Guizhou Province | *Mus musculus* | City | Trap success | 2007 | 0.11 |
| 454 | Guizhou Province | *Mus musculus* | City | Trap success | 2008 | 0.01 |
| 455 | Guizhou Province | *Mus musculus* | City | Trap success | 2009 | 0.00 |
| 456 | Guizhou Province | *Rattus norvegicus* | City | Trap success | 1986 | 1.00 |
| 457 | Guizhou Province | *Rattus norvegicus* | City | Trap success | 1987 | 0.75 |
| 458 | Guizhou Province | *Rattus norvegicus* | City | Trap success | 1988 | 0.11 |
| 459 | Guizhou Province | *Rattus norvegicus* | City | Trap success | 1989 | 0.17 |
| 460 | Guizhou Province | *Rattus norvegicus* | City | Trap success | 1990 | 0.14 |
| 461 | Guizhou Province | *Rattus norvegicus* | City | Trap success | 1991 | 0.30 |
| 462 | Guizhou Province | *Rattus norvegicus* | City | Trap success | 1992 | 0.71 |
| 463 | Guizhou Province | *Rattus norvegicus* | City | Trap success | 1993 | 0.70 |
| 464 | Guizhou Province | *Rattus norvegicus* | City | Trap success | 1994 | 0.48 |
| 465 | Guizhou Province | *Rattus norvegicus* | City | Trap success | 1995 | 0.61 |
| 466 | Guizhou Province | *Rattus norvegicus* | City | Trap success | 1996 | 0.42 |
| 467 | Guizhou Province | *Rattus norvegicus* | City | Trap success | 1997 | 0.30 |
| 468 | Guizhou Province | *Rattus norvegicus* | City | Trap success | 1998 | 0.31 |
| 469 | Guizhou Province | *Rattus norvegicus* | City | Trap success | 1999 | 0.24 |
| 470 | Guizhou Province | *Rattus norvegicus* | City | Trap success | 2000 | 0.18 |
| 471 | Guizhou Province | *Rattus norvegicus* | City | Trap success | 2001 | 0.17 |
| 472 | Guizhou Province | *Rattus norvegicus* | City | Trap success | 2002 | 0.12 |
| 473 | Guizhou Province | *Rattus norvegicus* | City | Trap success | 2003 | 0.05 |
| 474 | Guizhou Province | *Rattus norvegicus* | City | Trap success | 2004 | 0.00 |
| 475 | Guizhou Province | *Rattus norvegicus* | City | Trap success | 2005 | 0.06 |
| 476 | Guizhou Province | *Rattus norvegicus* | City | Trap success | 2006 | 0.02 |
| 477 | Guizhou Province | *Rattus norvegicus* | City | Trap success | 2007 | 0.04 |
| 478 | Guizhou Province | *Rattus norvegicus* | City | Trap success | 2008 | 0.03 |
| 479 | Guizhou Province | *Rattus norvegicus* | City | Trap success | 2009 | 0.13 |
| 480 | Guizhou Province | *Rattus tanezumi* | City | Trap success | 1986 | 1.00 |
| 481 | Guizhou Province | *Rattus tanezumi* | City | Trap success | 1987 | 0.18 |
| 482 | Guizhou Province | *Rattus tanezumi* | City | Trap success | 1988 | 0.01 |
| 483 | Guizhou Province | *Rattus tanezumi* | City | Trap success | 1989 | 0.03 |
| 484 | Guizhou Province | *Rattus tanezumi* | City | Trap success | 1990 | 0.03 |
| 485 | Guizhou Province | *Rattus tanezumi* | City | Trap success | 1991 | 0.00 |
| 486 | Guizhou Province | *Rattus tanezumi* | City | Trap success | 1992 | 0.01 |
| 487 | Guizhou Province | *Rattus tanezumi* | City | Trap success | 1993 | 0.19 |
| 488 | Guizhou Province | *Rattus tanezumi* | City | Trap success | 1994 | 0.03 |
| 489 | Guizhou Province | *Rattus tanezumi* | City | Trap success | 1995 | 0.58 |
| 490 | Guizhou Province | *Rattus tanezumi* | City | Trap success | 1996 | 0.38 |
| 491 | Guizhou Province | *Rattus tanezumi* | City | Trap success | 1997 | 0.50 |
| 492 | Guizhou Province | *Rattus tanezumi* | City | Trap success | 1998 | 0.46 |
| 493 | Guizhou Province | *Rattus tanezumi* | City | Trap success | 1999 | 0.50 |
| 494 | Guizhou Province | *Rattus tanezumi* | City | Trap success | 2000 | 0.61 |
| 495 | Guizhou Province | *Rattus tanezumi* | City | Trap success | 2001 | 0.41 |
| 496 | Guizhou Province | *Rattus tanezumi* | City | Trap success | 2002 | 0.54 |
| 497 | Guizhou Province | *Rattus tanezumi* | City | Trap success | 2003 | 0.02 |
| 498 | Guizhou Province | *Rattus tanezumi* | City | Trap success | 2004 | 0.15 |
| 499 | Guizhou Province | *Rattus tanezumi* | City | Trap success | 2005 | 0.19 |
| 500 | Guizhou Province | *Rattus tanezumi* | City | Trap success | 2006 | 0.09 |
| 501 | Guizhou Province | *Rattus tanezumi* | City | Trap success | 2007 | 0.25 |
| 502 | Guizhou Province | *Rattus tanezumi* | City | Trap success | 2008 | 0.31 |
| 503 | Guizhou Province | *Rattus tanezumi* | City | Trap success | 2009 | 0.39 |
| 504 | Guizhou Province | rodent | City | Trap success | 1986 | 1.00 |
| 505 | Guizhou Province | rodent | City | Trap success | 1987 | 0.64 |
| 506 | Guizhou Province | rodent | City | Trap success | 1988 | 0.08 |
| 507 | Guizhou Province | rodent | City | Trap success | 1989 | 0.17 |
| 508 | Guizhou Province | rodent | City | Trap success | 1990 | 0.29 |
| 509 | Guizhou Province | rodent | City | Trap success | 1991 | 0.56 |
| 510 | Guizhou Province | rodent | City | Trap success | 1992 | 0.62 |
| 511 | Guizhou Province | rodent | City | Trap success | 1993 | 0.78 |
| 512 | Guizhou Province | rodent | City | Trap success | 1994 | 0.46 |
| 513 | Guizhou Province | rodent | City | Trap success | 1995 | 0.69 |
| 514 | Guizhou Province | rodent | City | Trap success | 1996 | 0.49 |
| 515 | Guizhou Province | rodent | City | Trap success | 1997 | 0.41 |
| 516 | Guizhou Province | rodent | City | Trap success | 1998 | 0.38 |
| 517 | Guizhou Province | rodent | City | Trap success | 1999 | 0.34 |
| 518 | Guizhou Province | rodent | City | Trap success | 2000 | 0.30 |
| 519 | Guizhou Province | rodent | City | Trap success | 2001 | 0.27 |
| 520 | Guizhou Province | rodent | City | Trap success | 2002 | 0.25 |
| 521 | Guizhou Province | rodent | City | Trap success | 2003 | 0.02 |
| 522 | Guizhou Province | rodent | City | Trap success | 2004 | 0.00 |
| 523 | Guizhou Province | rodent | City | Trap success | 2005 | 0.11 |
| 524 | Guizhou Province | rodent | City | Trap success | 2006 | 0.00 |
| 525 | Guizhou Province | rodent | City | Trap success | 2007 | 0.07 |
| 526 | Guizhou Province | rodent | City | Trap success | 2008 | 0.04 |
| 527 | Guizhou Province | rodent | City | Trap success | 2009 | 0.11 |
| 528 | Guizhou Province | rodent | City | Trap success | 1986 | 1.00 |
| 529 | Guizhou Province | rodent | City | Trap success | 1987 | 0.64 |
| 530 | Guizhou Province | rodent | City | Trap success | 1988 | 0.09 |
| 531 | Guizhou Province | rodent | City | Trap success | 1989 | 0.16 |
| 532 | Guizhou Province | rodent | City | Trap success | 1990 | 0.26 |
| 533 | Guizhou Province | rodent | City | Trap success | 1991 | 0.50 |
| 534 | Guizhou Province | rodent | City | Trap success | 1992 | 0.56 |
| 535 | Guizhou Province | rodent | City | Trap success | 1993 | 0.84 |
| 536 | Guizhou Province | rodent | City | Trap success | 1994 | 0.42 |
| 537 | Guizhou Province | rodent | City | Trap success | 1995 | 0.66 |
| 538 | Guizhou Province | rodent | City | Trap success | 1996 | 0.45 |
| 539 | Guizhou Province | rodent | City | Trap success | 1997 | 0.37 |
| 540 | Guizhou Province | rodent | City | Trap success | 1998 | 0.34 |
| 541 | Guizhou Province | rodent | City | Trap success | 1999 | 0.31 |
| 542 | Guizhou Province | rodent | City | Trap success | 2000 | 0.28 |
| 543 | Guizhou Province | rodent | City | Trap success | 2001 | 0.25 |
| 544 | Guizhou Province | rodent | City | Trap success | 2002 | 0.23 |
| 545 | Guizhou Province | rodent | City | Trap success | 2003 | 0.00 |
| 546 | Guizhou Province | rodent | City | Trap success | 2004 | 0.00 |
| 547 | Guizhou Province | rodent | City | Trap success | 2005 | 0.10 |
| 548 | Guizhou Province | rodent | City | Trap success | 2006 | 0.01 |
| 549 | Guizhou Province | rodent | City | Trap success | 2007 | 0.07 |
| 550 | Guizhou Province | rodent | City | Trap success | 2008 | 0.03 |
| 551 | Guizhou Province | rodent | City | Trap success | 2009 | 0.10 |
| 552 | Guizhou Province | rodent | City | Trap success | 2010 | 0.07 |
| 553 | Guizhou Province | rodent | City | Trap success | 2011 | 0.04 |
| 554 | Guizhou Province | rodent | City | Trap success | 2012 | 0.04 |
| 555 | Guizhou Province | rodent | Farmland | Trap success | 1986 | 0.89 |
| 556 | Guizhou Province | rodent | Farmland | Trap success | 1987 | 0.89 |
| 557 | Guizhou Province | rodent | Farmland | Trap success | 1988 | 0.00 |
| 558 | Guizhou Province | rodent | Farmland | Trap success | 1989 | 0.06 |
| 559 | Guizhou Province | rodent | Farmland | Trap success | 1990 | 0.16 |
| 560 | Guizhou Province | rodent | Farmland | Trap success | 1991 | 0.22 |
| 561 | Guizhou Province | rodent | Farmland | Trap success | 1992 | 0.75 |
| 562 | Guizhou Province | rodent | Farmland | Trap success | 1993 | 1.00 |
| 563 | Guizhou Province | rodent | Farmland | Trap success | 1994 | 0.96 |
| 564 | Guizhou Province | rodent | Farmland | Trap success | 1995 | 0.75 |
| 565 | Guizhou Province | rodent | Farmland | Trap success | 1996 | 0.77 |
| 566 | Guizhou Province | rodent | Farmland | Trap success | 1997 | 0.58 |
| 567 | Guizhou Province | rodent | Farmland | Trap success | 1998 | 0.57 |
| 568 | Guizhou Province | rodent | Farmland | Trap success | 1999 | 0.38 |
| 569 | Guizhou Province | rodent | Farmland | Trap success | 2000 | 0.26 |
| 570 | Guizhou Province | rodent | Farmland | Trap success | 2001 | 0.23 |
| 571 | Guizhou Province | rodent | Farmland | Trap success | 2002 | 0.37 |
| 572 | Guizhou Province | rodent | Farmland | Trap success | 2003 | 0.15 |
| 573 | Guizhou Province | rodent | Farmland | Trap success | 2004 | 0.01 |
| 574 | Guizhou Province | rodent | Farmland | Trap success | 2005 | 0.07 |
| 575 | Guizhou Province | rodent | Farmland | Trap success | 2006 | 0.12 |
| 576 | Guizhou Province | rodent | Farmland | Trap success | 2007 | 0.20 |
| 577 | Guizhou Province | rodent | Farmland | Trap success | 2008 | 0.15 |
| 578 | Guizhou Province | rodent | Farmland | Trap success | 2009 | 0.20 |
| 579 | Guizhou Province | rodent | Farmland | Trap success | 2010 | 0.13 |
| 580 | Guizhou Province | rodent | Farmland | Trap success | 2011 | 0.01 |
| 581 | Guizhou Province | rodent | Farmland | Trap success | 2012 | 0.00 |
| 582 | Guizhou Province | *Apodemus agrarius* | Farmland | Trap success | 1986 | 0.90 |
| 583 | Guizhou Province | *Apodemus agrarius* | Farmland | Trap success | 1987 | 0.85 |
| 584 | Guizhou Province | *Apodemus agrarius* | Farmland | Trap success | 1988 | 0.09 |
| 585 | Guizhou Province | *Apodemus agrarius* | Farmland | Trap success | 1989 | 0.20 |
| 586 | Guizhou Province | *Apodemus agrarius* | Farmland | Trap success | 1990 | 0.30 |
| 587 | Guizhou Province | *Apodemus agrarius* | Farmland | Trap success | 1991 | 0.37 |
| 588 | Guizhou Province | *Apodemus agrarius* | Farmland | Trap success | 1992 | 0.88 |
| 589 | Guizhou Province | *Apodemus agrarius* | Farmland | Trap success | 1993 | 0.91 |
| 590 | Guizhou Province | *Apodemus agrarius* | Farmland | Trap success | 1994 | 1.00 |
| 591 | Guizhou Province | *Apodemus agrarius* | Farmland | Trap success | 1995 | 0.77 |
| 592 | Guizhou Province | *Apodemus agrarius* | Farmland | Trap success | 1996 | 0.79 |
| 593 | Guizhou Province | *Apodemus agrarius* | Farmland | Trap success | 1997 | 0.68 |
| 594 | Guizhou Province | *Apodemus agrarius* | Farmland | Trap success | 1998 | 0.66 |
| 595 | Guizhou Province | *Apodemus agrarius* | Farmland | Trap success | 1999 | 0.46 |
| 596 | Guizhou Province | *Apodemus agrarius* | Farmland | Trap success | 2000 | 0.35 |
| 597 | Guizhou Province | *Apodemus agrarius* | Farmland | Trap success | 2001 | 0.32 |
| 598 | Guizhou Province | *Apodemus agrarius* | Farmland | Trap success | 2002 | 0.45 |
| 599 | Guizhou Province | *Apodemus agrarius* | Farmland | Trap success | 2003 | 0.22 |
| 600 | Guizhou Province | *Apodemus agrarius* | Farmland | Trap success | 2004 | 0.10 |
| 601 | Guizhou Province | *Apodemus agrarius* | Farmland | Trap success | 2005 | 0.24 |
| 602 | Guizhou Province | *Apodemus agrarius* | Farmland | Trap success | 2006 | 0.26 |
| 603 | Guizhou Province | *Apodemus agrarius* | Farmland | Trap success | 2007 | 0.27 |
| 604 | Guizhou Province | *Apodemus agrarius* | Farmland | Trap success | 2008 | 0.25 |
| 605 | Guizhou Province | *Apodemus agrarius* | Farmland | Trap success | 2009 | 0.35 |
| 606 | Guizhou Province | *Apodemus agrarius* | Farmland | Trap success | 2010 | 0.24 |
| 607 | Guizhou Province | *Apodemus agrarius* | Farmland | Trap success | 2011 | 0.18 |
| 608 | Guizhou Province | *Apodemus agrarius* | Farmland | Trap success | 2012 | 0.17 |
| 609 | Guizhou Province | *Apodemus agrarius* | Farmland | Trap success | 2013 | 0.05 |
| 610 | Guizhou Province | *Apodemus agrarius* | Farmland | Trap success | 2014 | 0.21 |
| 611 | Guizhou Province | *Apodemus agrarius* | Farmland | Trap success | 2015 | 0.00 |
| 612 | Guizhou Province | rodent | City | Trap success | 1985 | 0.97 |
| 613 | Guizhou Province | rodent | City | Trap success | 1986 | 0.90 |
| 614 | Guizhou Province | rodent | City | Trap success | 1987 | 0.84 |
| 615 | Guizhou Province | rodent | City | Trap success | 1988 | 0.78 |
| 616 | Guizhou Province | rodent | City | Trap success | 1989 | 0.67 |
| 617 | Guizhou Province | rodent | City | Trap success | 1990 | 0.76 |
| 618 | Guizhou Province | rodent | City | Trap success | 1991 | 1.00 |
| 619 | Guizhou Province | rodent | City | Trap success | 1992 | 0.64 |
| 620 | Guizhou Province | rodent | City | Trap success | 1993 | 0.89 |
| 621 | Guizhou Province | rodent | City | Trap success | 1994 | 0.88 |
| 622 | Guizhou Province | rodent | City | Trap success | 1995 | 0.90 |
| 623 | Guizhou Province | rodent | City | Trap success | 1996 | 0.72 |
| 624 | Guizhou Province | rodent | City | Trap success | 1997 | 0.80 |
| 625 | Guizhou Province | rodent | City | Trap success | 1998 | 0.69 |
| 626 | Guizhou Province | rodent | City | Trap success | 1999 | 0.52 |
| 627 | Guizhou Province | rodent | City | Trap success | 2000 | 0.49 |
| 628 | Guizhou Province | rodent | City | Trap success | 2001 | 0.41 |
| 629 | Guizhou Province | rodent | City | Trap success | 2002 | 0.25 |
| 630 | Guizhou Province | rodent | City | Trap success | 2003 | 0.23 |
| 631 | Guizhou Province | rodent | City | Trap success | 2004 | 0.00 |
| 632 | Guizhou Province | rodent | City | Trap success | 2005 | 0.05 |
| 633 | Guizhou Province | rodent | City | Trap success | 2006 | 0.10 |
| 634 | Guizhou Province | rodent | City | Trap success | 2007 | 0.09 |
| 635 | Guizhou Province | rodent | City | Trap success | 2008 | 0.02 |
| 636 | Guizhou Province | rodent | City | Trap success | 2009 | 0.18 |
| 637 | Guizhou Province | rodent | City | Trap success | 2010 | 0.14 |
| 638 | Guizhou Province | rodent | City | Trap success | 2011 | 0.12 |
| 639 | Guizhou Province | rodent | Farmland | Trap success | 1985 | 0.60 |
| 640 | Guizhou Province | rodent | Farmland | Trap success | 1986 | 0.44 |
| 641 | Guizhou Province | rodent | Farmland | Trap success | 1987 | 0.66 |
| 642 | Guizhou Province | rodent | Farmland | Trap success | 1988 | 0.61 |
| 643 | Guizhou Province | rodent | Farmland | Trap success | 1989 | 0.81 |
| 644 | Guizhou Province | rodent | Farmland | Trap success | 1990 | 0.72 |
| 645 | Guizhou Province | rodent | Farmland | Trap success | 1991 | 0.73 |
| 646 | Guizhou Province | rodent | Farmland | Trap success | 1992 | 0.78 |
| 647 | Guizhou Province | rodent | Farmland | Trap success | 1993 | 0.82 |
| 648 | Guizhou Province | rodent | Farmland | Trap success | 1994 | 0.94 |
| 649 | Guizhou Province | rodent | Farmland | Trap success | 1995 | 0.98 |
| 650 | Guizhou Province | rodent | Farmland | Trap success | 1996 | 1.00 |
| 651 | Guizhou Province | rodent | Farmland | Trap success | 1997 | 0.79 |
| 652 | Guizhou Province | rodent | Farmland | Trap success | 1998 | 0.91 |
| 653 | Guizhou Province | rodent | Farmland | Trap success | 1999 | 0.56 |
| 654 | Guizhou Province | rodent | Farmland | Trap success | 2000 | 0.56 |
| 655 | Guizhou Province | rodent | Farmland | Trap success | 2001 | 0.41 |
| 656 | Guizhou Province | rodent | Farmland | Trap success | 2002 | 0.39 |
| 657 | Guizhou Province | rodent | Farmland | Trap success | 2003 | 0.36 |
| 658 | Guizhou Province | rodent | Farmland | Trap success | 2004 | 0.00 |
| 659 | Guizhou Province | rodent | Farmland | Trap success | 2005 | 0.25 |
| 660 | Guizhou Province | rodent | Farmland | Trap success | 2006 | 0.13 |
| 661 | Guizhou Province | rodent | Farmland | Trap success | 2007 | 0.14 |
| 662 | Guizhou Province | rodent | Farmland | Trap success | 2008 | 0.11 |
| 663 | Guizhou Province | rodent | Farmland | Trap success | 2009 | 0.29 |
| 664 | Guizhou Province | rodent | Farmland | Trap success | 2010 | 0.09 |
| 665 | Guizhou Province | rodent | Farmland | Trap success | 2011 | 0.05 |
| 666 | Guizhou Province | *Apodemus agrarius* | Farmland | Trap success | 1985 | 0.39 |
| 667 | Guizhou Province | *Apodemus agrarius* | Farmland | Trap success | 1986 | 0.28 |
| 668 | Guizhou Province | *Apodemus agrarius* | Farmland | Trap success | 1987 | 0.38 |
| 669 | Guizhou Province | *Apodemus agrarius* | Farmland | Trap success | 1988 | 0.32 |
| 670 | Guizhou Province | *Apodemus agrarius* | Farmland | Trap success | 1989 | 0.37 |
| 671 | Guizhou Province | *Apodemus agrarius* | Farmland | Trap success | 1990 | 0.40 |
| 672 | Guizhou Province | *Apodemus agrarius* | Farmland | Trap success | 1991 | 0.55 |
| 673 | Guizhou Province | *Apodemus agrarius* | Farmland | Trap success | 1992 | 0.98 |
| 674 | Guizhou Province | *Apodemus agrarius* | Farmland | Trap success | 1993 | 0.67 |
| 675 | Guizhou Province | *Apodemus agrarius* | Farmland | Trap success | 1994 | 0.83 |
| 676 | Guizhou Province | *Apodemus agrarius* | Farmland | Trap success | 1995 | 1.00 |
| 677 | Guizhou Province | *Apodemus agrarius* | Farmland | Trap success | 1996 | 0.92 |
| 678 | Guizhou Province | *Apodemus agrarius* | Farmland | Trap success | 1997 | 0.82 |
| 679 | Guizhou Province | *Apodemus agrarius* | Farmland | Trap success | 1998 | 0.83 |
| 680 | Guizhou Province | *Apodemus agrarius* | Farmland | Trap success | 1999 | 0.58 |
| 681 | Guizhou Province | *Apodemus agrarius* | Farmland | Trap success | 2000 | 0.54 |
| 682 | Guizhou Province | *Apodemus agrarius* | Farmland | Trap success | 2001 | 0.45 |
| 683 | Guizhou Province | *Apodemus agrarius* | Farmland | Trap success | 2002 | 0.41 |
| 684 | Guizhou Province | *Apodemus agrarius* | Farmland | Trap success | 2003 | 0.41 |
| 685 | Guizhou Province | *Apodemus agrarius* | Farmland | Trap success | 2004 | 0.11 |
| 686 | Guizhou Province | *Apodemus agrarius* | Farmland | Trap success | 2005 | 0.26 |
| 687 | Guizhou Province | *Apodemus agrarius* | Farmland | Trap success | 2006 | 0.06 |
| 688 | Guizhou Province | *Apodemus agrarius* | Farmland | Trap success | 2007 | 0.05 |
| 689 | Guizhou Province | *Apodemus agrarius* | Farmland | Trap success | 2008 | 0.01 |
| 690 | Guizhou Province | *Apodemus agrarius* | Farmland | Trap success | 2009 | 0.06 |
| 691 | Guizhou Province | *Apodemus agrarius* | Farmland | Trap success | 2010 | 0.04 |
| 692 | Guizhou Province | *Apodemus agrarius* | Farmland | Trap success | 2011 | 0.00 |
| 693 | Guizhou Province | rodent | City | Trap success | 1994 | 0.81 |
| 694 | Guizhou Province | rodent | City | Trap success | 1995 | 0.73 |
| 695 | Guizhou Province | rodent | City | Trap success | 1996 | 0.92 |
| 696 | Guizhou Province | rodent | City | Trap success | 1997 | 1.00 |
| 697 | Guizhou Province | rodent | City | Trap success | 1998 | 0.87 |
| 698 | Guizhou Province | rodent | City | Trap success | 1999 | 0.51 |
| 699 | Guizhou Province | rodent | City | Trap success | 2000 | 0.31 |
| 700 | Guizhou Province | rodent | City | Trap success | 2001 | 0.07 |
| 701 | Guizhou Province | rodent | City | Trap success | 2002 | 0.00 |
| 702 | Guizhou Province | rodent | City | Trap success | 2003 | 0.00 |
| 703 | Guizhou Province | rodent | City | Trap success | 2004 | 0.12 |
| 704 | Guizhou Province | rodent | City | Trap success | 2005 | 0.16 |
| 705 | Guizhou Province | rodent | City | Trap success | 2006 | 0.23 |
| 706 | Guizhou Province | rodent | City | Trap success | 2007 | 0.25 |
| 707 | Guizhou Province | rodent | City | Trap success | 2008 | 0.17 |
| 708 | Guizhou Province | rodent | City | Trap success | 2009 | 0.13 |
| 709 | Guizhou Province | rodent | City | Trap success | 2010 | 0.15 |
| 710 | Guizhou Province | rodent | City | Trap success | 2011 | 0.18 |
| 711 | Guizhou Province | rodent | City | Trap success | 2012 | 0.18 |
| 712 | Guizhou Province | rodent | City | Trap success | 2013 | 0.08 |
| 713 | Guizhou Province | rodent | City | Trap success | 2014 | 0.10 |
| 714 | Guizhou Province | rodent | City | Trap success | 2015 | 0.12 |
| 715 | Guizhou Province | rodent | Farmland | Trap success | 1994 | 0.45 |
| 716 | Guizhou Province | rodent | Farmland | Trap success | 1995 | 0.33 |
| 717 | Guizhou Province | rodent | Farmland | Trap success | 1996 | 0.78 |
| 718 | Guizhou Province | rodent | Farmland | Trap success | 1997 | 1.00 |
| 719 | Guizhou Province | rodent | Farmland | Trap success | 1998 | 0.66 |
| 720 | Guizhou Province | rodent | Farmland | Trap success | 1999 | 0.65 |
| 721 | Guizhou Province | rodent | Farmland | Trap success | 2000 | 0.60 |
| 722 | Guizhou Province | rodent | Farmland | Trap success | 2001 | 0.13 |
| 723 | Guizhou Province | rodent | Farmland | Trap success | 2002 | 0.00 |
| 724 | Guizhou Province | rodent | Farmland | Trap success | 2003 | 0.12 |
| 725 | Guizhou Province | rodent | Farmland | Trap success | 2004 | 0.06 |
| 726 | Guizhou Province | rodent | Farmland | Trap success | 2005 | 0.27 |
| 727 | Guizhou Province | rodent | Farmland | Trap success | 2006 | 0.30 |
| 728 | Guizhou Province | rodent | Farmland | Trap success | 2007 | 0.41 |
| 729 | Guizhou Province | rodent | Farmland | Trap success | 2008 | 0.18 |
| 730 | Guizhou Province | rodent | Farmland | Trap success | 2009 | 0.24 |
| 731 | Guizhou Province | rodent | Farmland | Trap success | 2010 | 0.27 |
| 732 | Guizhou Province | rodent | Farmland | Trap success | 2011 | 0.24 |
| 733 | Guizhou Province | rodent | Farmland | Trap success | 2012 | 0.18 |
| 734 | Guizhou Province | rodent | Farmland | Trap success | 2013 | 0.02 |
| 735 | Guizhou Province | rodent | Farmland | Trap success | 2014 | 0.07 |
| 736 | Guizhou Province | rodent | Farmland | Trap success | 2015 | 0.05 |
| 737 | Guizhou Province | rodent | Mixed | Trap success | 1986 | 0.72 |
| 738 | Guizhou Province | rodent | Mixed | Trap success | 1987 | 0.88 |
| 739 | Guizhou Province | rodent | Mixed | Trap success | 1988 | 0.57 |
| 740 | Guizhou Province | rodent | Mixed | Trap success | 1989 | 0.75 |
| 741 | Guizhou Province | rodent | Mixed | Trap success | 1990 | 0.97 |
| 742 | Guizhou Province | rodent | Mixed | Trap success | 1991 | 0.71 |
| 743 | Guizhou Province | rodent | Mixed | Trap success | 1992 | 0.79 |
| 744 | Guizhou Province | rodent | Mixed | Trap success | 1994 | 1.00 |
| 745 | Guizhou Province | rodent | Mixed | Trap success | 1995 | 0.47 |
| 746 | Guizhou Province | rodent | Mixed | Trap success | 1996 | 0.81 |
| 747 | Guizhou Province | rodent | Mixed | Trap success | 1997 | 0.27 |
| 748 | Guizhou Province | rodent | Mixed | Trap success | 1998 | 0.37 |
| 749 | Guizhou Province | rodent | Mixed | Trap success | 1999 | 0.08 |
| 750 | Guizhou Province | rodent | Mixed | Trap success | 2000 | 0.26 |
| 751 | Guizhou Province | rodent | Mixed | Trap success | 2001 | 0.06 |
| 752 | Guizhou Province | rodent | Mixed | Trap success | 2002 | 0.01 |
| 753 | Guizhou Province | rodent | Mixed | Trap success | 2003 | 0.04 |
| 754 | Guizhou Province | rodent | Mixed | Trap success | 2004 | 0.04 |
| 755 | Guizhou Province | rodent | Mixed | Trap success | 2005 | 0.01 |
| 756 | Guizhou Province | rodent | Mixed | Trap success | 2006 | 0.12 |
| 757 | Guizhou Province | rodent | Mixed | Trap success | 2007 | 0.14 |
| 758 | Guizhou Province | rodent | Mixed | Trap success | 2008 | 0.13 |
| 759 | Guizhou Province | rodent | Mixed | Trap success | 2009 | 0.11 |
| 760 | Guizhou Province | rodent | Mixed | Trap success | 2010 | 0.07 |
| 761 | Guizhou Province | rodent | Mixed | Trap success | 2011 | 0.07 |
| 762 | Guizhou Province | rodent | Mixed | Trap success | 2012 | 0.00 |
| 763 | Guizhou Province | rodent | Mixed | Trap success | 2013 | 0.11 |
| 764 | Guizhou Province | rodent | Mixed | Trap success | 1986 | 1.00 |
| 765 | Guizhou Province | rodent | Mixed | Trap success | 1987 | 0.44 |
| 766 | Guizhou Province | rodent | Mixed | Trap success | 1988 | 0.33 |
| 767 | Guizhou Province | rodent | Mixed | Trap success | 1989 | 0.50 |
| 768 | Guizhou Province | rodent | Mixed | Trap success | 1990 | 0.55 |
| 769 | Guizhou Province | rodent | Mixed | Trap success | 1991 | 0.39 |
| 770 | Guizhou Province | rodent | Mixed | Trap success | 1992 | 0.33 |
| 771 | Guizhou Province | rodent | Mixed | Trap success | 1993 | 0.37 |
| 772 | Guizhou Province | rodent | Mixed | Trap success | 1994 | 0.26 |
| 773 | Guizhou Province | rodent | Mixed | Trap success | 1995 | 0.28 |
| 774 | Guizhou Province | rodent | Mixed | Trap success | 1996 | 0.34 |
| 775 | Guizhou Province | rodent | Mixed | Trap success | 1997 | 0.19 |
| 776 | Guizhou Province | rodent | Mixed | Trap success | 1998 | 0.14 |
| 777 | Guizhou Province | rodent | Mixed | Trap success | 1999 | 0.05 |
| 778 | Guizhou Province | rodent | Mixed | Trap success | 2000 | 0.14 |
| 779 | Guizhou Province | rodent | Mixed | Trap success | 2001 | 0.13 |
| 780 | Guizhou Province | rodent | Mixed | Trap success | 2002 | 0.13 |
| 781 | Guizhou Province | rodent | Mixed | Trap success | 2003 | 0.03 |
| 782 | Guizhou Province | rodent | Mixed | Trap success | 2004 | 0.10 |
| 783 | Guizhou Province | rodent | Mixed | Trap success | 2005 | 0.28 |
| 784 | Guizhou Province | rodent | Mixed | Trap success | 2006 | 0.34 |
| 785 | Guizhou Province | rodent | Mixed | Trap success | 2007 | 0.18 |
| 786 | Guizhou Province | rodent | Mixed | Trap success | 2008 | 0.11 |
| 787 | Guizhou Province | rodent | Mixed | Trap success | 2009 | 0.04 |
| 788 | Guizhou Province | rodent | Mixed | Trap success | 2010 | 0.00 |
| 789 | Guizhou Province | rodent | City | Trap success | 1987 | 1.00 |
| 790 | Guizhou Province | rodent | City | Trap success | 1988 | 0.84 |
| 791 | Guizhou Province | rodent | City | Trap success | 1989 | 0.82 |
| 792 | Guizhou Province | rodent | City | Trap success | 1990 | 1.00 |
| 793 | Guizhou Province | rodent | City | Trap success | 1997 | 0.20 |
| 794 | Guizhou Province | rodent | City | Trap success | 1998 | 0.12 |
| 795 | Guizhou Province | rodent | City | Trap success | 1999 | 0.14 |
| 796 | Guizhou Province | rodent | City | Trap success | 2000 | 0.43 |
| 797 | Guizhou Province | rodent | City | Trap success | 2001 | 0.00 |
| 798 | Guizhou Province | rodent | City | Trap success | 2002 | 0.00 |
| 799 | Guizhou Province | rodent | City | Trap success | 2003 | 0.58 |
| 800 | Guizhou Province | rodent | City | Trap success | 2004 | 0.21 |
| 801 | Guizhou Province | rodent | City | Trap success | 2005 | 0.19 |
| 802 | Guizhou Province | rodent | City | Trap success | 2006 | 0.17 |
| 803 | Guizhou Province | rodent | City | Trap success | 2007 | 0.15 |
| 804 | Guizhou Province | rodent | City | Trap success | 2008 | 0.15 |
| 805 | Guizhou Province | rodent | City | Trap success | 2009 | 0.12 |
| 806 | Guizhou Province | rodent | City | Trap success | 2010 | 0.10 |
| 807 | Guizhou Province | rodent | City | Trap success | 2011 | 0.10 |
| 808 | Guizhou Province | rodent | City | Trap success | 2012 | 0.21 |
| 809 | Guizhou Province | rodent | City | Trap success | 2013 | 0.19 |
| 810 | Guizhou Province | rodent | Farmland | Trap success | 1987 | 1.00 |
| 811 | Guizhou Province | rodent | Farmland | Trap success | 1988 | 0.72 |
| 812 | Guizhou Province | rodent | Farmland | Trap success | 1989 | 0.71 |
| 813 | Guizhou Province | rodent | Farmland | Trap success | 1990 | 0.72 |
| 814 | Guizhou Province | rodent | Farmland | Trap success | 1997 | 0.09 |
| 815 | Guizhou Province | rodent | Farmland | Trap success | 1998 | 0.15 |
| 816 | Guizhou Province | rodent | Farmland | Trap success | 1999 | 0.19 |
| 817 | Guizhou Province | rodent | Farmland | Trap success | 2000 | 0.36 |
| 818 | Guizhou Province | rodent | Farmland | Trap success | 2001 | 0.00 |
| 819 | Guizhou Province | rodent | Farmland | Trap success | 2002 | 0.03 |
| 820 | Guizhou Province | rodent | Farmland | Trap success | 2003 | 0.49 |
| 821 | Guizhou Province | rodent | Farmland | Trap success | 2004 | 0.17 |
| 822 | Guizhou Province | rodent | Farmland | Trap success | 2005 | 0.03 |
| 823 | Guizhou Province | rodent | Farmland | Trap success | 2006 | 0.12 |
| 824 | Guizhou Province | rodent | Farmland | Trap success | 2007 | 0.16 |
| 825 | Guizhou Province | rodent | Farmland | Trap success | 2008 | 0.15 |
| 826 | Guizhou Province | rodent | Farmland | Trap success | 2009 | 0.13 |
| 827 | Guizhou Province | rodent | Farmland | Trap success | 2010 | 0.13 |
| 828 | Guizhou Province | rodent | Farmland | Trap success | 2011 | 0.06 |
| 829 | Guizhou Province | rodent | Farmland | Trap success | 2012 | 0.13 |
| 830 | Guizhou Province | rodent | Farmland | Trap success | 2013 | 0.17 |
| 831 | Guizhou Province | rodent | Mixed | Trap success | 1987 | 1.00 |
| 832 | Guizhou Province | rodent | Mixed | Trap success | 1988 | 0.75 |
| 833 | Guizhou Province | rodent | Mixed | Trap success | 1989 | 0.74 |
| 834 | Guizhou Province | rodent | Mixed | Trap success | 1990 | 0.82 |
| 835 | Guizhou Province | rodent | Mixed | Trap success | 1997 | 0.13 |
| 836 | Guizhou Province | rodent | Mixed | Trap success | 1998 | 0.13 |
| 837 | Guizhou Province | rodent | Mixed | Trap success | 1999 | 0.16 |
| 838 | Guizhou Province | rodent | Mixed | Trap success | 2000 | 0.38 |
| 839 | Guizhou Province | rodent | Mixed | Trap success | 2001 | 0.00 |
| 840 | Guizhou Province | rodent | Mixed | Trap success | 2002 | 0.02 |
| 841 | Guizhou Province | rodent | Mixed | Trap success | 2003 | 0.48 |
| 842 | Guizhou Province | rodent | Mixed | Trap success | 2004 | 0.19 |
| 843 | Guizhou Province | rodent | Mixed | Trap success | 2005 | 0.10 |
| 844 | Guizhou Province | rodent | Mixed | Trap success | 2006 | 0.14 |
| 845 | Guizhou Province | rodent | Mixed | Trap success | 2007 | 0.15 |
| 846 | Guizhou Province | rodent | Mixed | Trap success | 2008 | 0.15 |
| 847 | Guizhou Province | rodent | Mixed | Trap success | 2009 | 0.12 |
| 848 | Guizhou Province | rodent | Mixed | Trap success | 2010 | 0.11 |
| 849 | Guizhou Province | rodent | Mixed | Trap success | 2011 | 0.07 |
| 850 | Guizhou Province | rodent | Mixed | Trap success | 2012 | 0.16 |
| 851 | Guizhou Province | rodent | Mixed | Trap success | 2013 | 0.17 |
| 852 | Hebei Province | rodent | Mixed | Trap success | 1985 | 0.38 |
| 853 | Hebei Province | rodent | Mixed | Trap success | 1986 | 0.76 |
| 854 | Hebei Province | rodent | Mixed | Trap success | 1987 | 1.00 |
| 855 | Hebei Province | rodent | Mixed | Trap success | 1988 | 0.32 |
| 856 | Hebei Province | rodent | Mixed | Trap success | 1989 | 0.21 |
| 857 | Hebei Province | rodent | Mixed | Trap success | 1990 | 0.18 |
| 858 | Hebei Province | rodent | Mixed | Trap success | 1991 | 0.22 |
| 859 | Hebei Province | rodent | Mixed | Trap success | 1992 | 0.40 |
| 860 | Hebei Province | rodent | Mixed | Trap success | 1993 | 0.24 |
| 861 | Hebei Province | rodent | Mixed | Trap success | 1994 | 0.48 |
| 862 | Hebei Province | rodent | Mixed | Trap success | 1995 | 0.33 |
| 863 | Hebei Province | rodent | Mixed | Trap success | 1996 | 0.20 |
| 864 | Hebei Province | rodent | Mixed | Trap success | 1997 | 0.15 |
| 865 | Hebei Province | rodent | Mixed | Trap success | 1998 | 0.43 |
| 866 | Hebei Province | rodent | Mixed | Trap success | 1999 | 0.49 |
| 867 | Hebei Province | rodent | Mixed | Trap success | 2000 | 0.63 |
| 868 | Hebei Province | rodent | Mixed | Trap success | 2001 | 0.73 |
| 869 | Hebei Province | rodent | Mixed | Trap success | 2002 | 0.58 |
| 870 | Hebei Province | rodent | Mixed | Trap success | 2003 | 0.57 |
| 871 | Hebei Province | rodent | Mixed | Trap success | 2004 | 0.17 |
| 872 | Hebei Province | rodent | Mixed | Trap success | 2005 | 0.00 |
| 873 | Hebei Province | rodent | Mixed | Trap success | 2006 | 0.29 |
| 874 | Hebei Province | rodent | Mixed | Trap success | 2007 | 0.11 |
| 875 | Hebei Province | rodent | Mixed | Trap success | 2008 | 0.08 |
| 876 | Hebei Province | *Apodemus agrarius* | Farmland | Trap success | 1984 | 0.32 |
| 877 | Hebei Province | *Apodemus agrarius* | Farmland | Trap success | 1985 | 0.41 |
| 878 | Hebei Province | *Apodemus agrarius* | Farmland | Trap success | 1986 | 0.99 |
| 879 | Hebei Province | *Apodemus agrarius* | Farmland | Trap success | 1987 | 0.23 |
| 880 | Hebei Province | *Apodemus agrarius* | Farmland | Trap success | 1988 | 0.05 |
| 881 | Hebei Province | *Apodemus agrarius* | Farmland | Trap success | 1989 | 0.01 |
| 882 | Hebei Province | *Apodemus agrarius* | Farmland | Trap success | 1990 | 0.03 |
| 883 | Hebei Province | *Apodemus agrarius* | Farmland | Trap success | 1991 | 0.11 |
| 884 | Hebei Province | *Apodemus agrarius* | Farmland | Trap success | 1992 | 0.47 |
| 885 | Hebei Province | *Apodemus agrarius* | Farmland | Trap success | 1993 | 1.00 |
| 886 | Hebei Province | *Apodemus agrarius* | Farmland | Trap success | 1994 | 0.37 |
| 887 | Hebei Province | *Apodemus agrarius* | Farmland | Trap success | 1995 | 0.45 |
| 888 | Hebei Province | *Apodemus agrarius* | Farmland | Trap success | 1996 | 0.43 |
| 889 | Hebei Province | *Apodemus agrarius* | Farmland | Trap success | 1997 | 0.26 |
| 890 | Hebei Province | *Apodemus agrarius* | Farmland | Trap success | 1998 | 0.26 |
| 891 | Hebei Province | *Apodemus agrarius* | Farmland | Trap success | 1999 | 0.46 |
| 892 | Hebei Province | *Apodemus agrarius* | Farmland | Trap success | 2000 | 0.29 |
| 893 | Hebei Province | *Apodemus agrarius* | Farmland | Trap success | 2001 | 0.40 |
| 894 | Hebei Province | *Apodemus agrarius* | Farmland | Trap success | 2002 | 0.03 |
| 895 | Hebei Province | *Apodemus agrarius* | Farmland | Trap success | 2003 | 0.10 |
| 896 | Hebei Province | *Apodemus agrarius* | Farmland | Trap success | 2004 | 0.08 |
| 897 | Hebei Province | *Apodemus agrarius* | Farmland | Trap success | 2005 | 0.08 |
| 898 | Hebei Province | *Apodemus agrarius* | Farmland | Trap success | 2006 | 0.10 |
| 899 | Hebei Province | *Apodemus agrarius* | Farmland | Trap success | 2007 | 0.39 |
| 900 | Hebei Province | *Apodemus agrarius* | Farmland | Trap success | 2008 | 0.28 |
| 901 | Hebei Province | *Apodemus agrarius* | Farmland | Trap success | 2009 | 0.12 |
| 902 | Hebei Province | *Apodemus agrarius* | Farmland | Trap success | 2010 | 0.00 |
| 903 | Hebei Province | *Cricetulus barabensis* | Farmland | Trap success | 1984 | 0.47 |
| 904 | Hebei Province | *Cricetulus barabensis* | Farmland | Trap success | 1985 | 0.42 |
| 905 | Hebei Province | *Cricetulus barabensis* | Farmland | Trap success | 1986 | 0.66 |
| 906 | Hebei Province | *Cricetulus barabensis* | Farmland | Trap success | 1987 | 0.39 |
| 907 | Hebei Province | *Cricetulus barabensis* | Farmland | Trap success | 1988 | 0.24 |
| 908 | Hebei Province | *Cricetulus barabensis* | Farmland | Trap success | 1989 | 0.08 |
| 909 | Hebei Province | *Cricetulus barabensis* | Farmland | Trap success | 1990 | 0.02 |
| 910 | Hebei Province | *Cricetulus barabensis* | Farmland | Trap success | 1991 | 0.25 |
| 911 | Hebei Province | *Cricetulus barabensis* | Farmland | Trap success | 1992 | 1.00 |
| 912 | Hebei Province | *Cricetulus barabensis* | Farmland | Trap success | 1993 | 0.53 |
| 913 | Hebei Province | *Cricetulus barabensis* | Farmland | Trap success | 1994 | 0.47 |
| 914 | Hebei Province | *Cricetulus barabensis* | Farmland | Trap success | 1995 | 0.30 |
| 915 | Hebei Province | *Cricetulus barabensis* | Farmland | Trap success | 1996 | 0.23 |
| 916 | Hebei Province | *Cricetulus barabensis* | Farmland | Trap success | 1997 | 0.11 |
| 917 | Hebei Province | *Cricetulus barabensis* | Farmland | Trap success | 1998 | 0.22 |
| 918 | Hebei Province | *Cricetulus barabensis* | Farmland | Trap success | 1999 | 0.30 |
| 919 | Hebei Province | *Cricetulus barabensis* | Farmland | Trap success | 2000 | 0.09 |
| 920 | Hebei Province | *Cricetulus barabensis* | Farmland | Trap success | 2001 | 0.02 |
| 921 | Hebei Province | *Cricetulus barabensis* | Farmland | Trap success | 2002 | 0.01 |
| 922 | Hebei Province | *Cricetulus barabensis* | Farmland | Trap success | 2003 | 0.05 |
| 923 | Hebei Province | *Cricetulus barabensis* | Farmland | Trap success | 2004 | 0.21 |
| 924 | Hebei Province | *Cricetulus barabensis* | Farmland | Trap success | 2005 | 0.00 |
| 925 | Hebei Province | *Cricetulus barabensis* | Farmland | Trap success | 2006 | 0.00 |
| 926 | Hebei Province | *Cricetulus barabensis* | Farmland | Trap success | 2007 | 0.01 |
| 927 | Hebei Province | *Cricetulus barabensis* | Farmland | Trap success | 2008 | 0.01 |
| 928 | Hebei Province | *Cricetulus barabensis* | Farmland | Trap success | 2009 | 0.08 |
| 929 | Hebei Province | *Cricetulus barabensis* | Farmland | Trap success | 2010 | 0.06 |
| 930 | Hebei Province | *Cricetulus barabensis* | Farmland | Trap success | 2011 | 0.02 |
| 931 | Hebei Province | *Cricetulus barabensis* | Farmland | Trap success | 2012 | 0.06 |
| 932 | Hebei Province | *Cricetulus barabensis* | Farmland | Trap success | 2013 | 0.03 |
| 933 | Hebei Province | *Cricetulus barabensis* | Farmland | Trap success | 2014 | 0.10 |
| 934 | Hebei Province | *Cricetulus barabensis* | Farmland | Trap success | 2015 | 0.10 |
| 935 | Hebei Province | *Cricetulus barabensis* | Farmland | Trap success | 2016 | 0.26 |
| 936 | Hebei Province | *Mus musculus* | Farmland | Trap success | 1984 | 1.00 |
| 937 | Hebei Province | *Mus musculus* | Farmland | Trap success | 1985 | 0.54 |
| 938 | Hebei Province | *Mus musculus* | Farmland | Trap success | 1986 | 0.35 |
| 939 | Hebei Province | *Mus musculus* | Farmland | Trap success | 1987 | 0.24 |
| 940 | Hebei Province | *Mus musculus* | Farmland | Trap success | 1988 | 0.25 |
| 941 | Hebei Province | *Mus musculus* | Farmland | Trap success | 1989 | 0.03 |
| 942 | Hebei Province | *Mus musculus* | Farmland | Trap success | 1990 | 0.04 |
| 943 | Hebei Province | *Mus musculus* | Farmland | Trap success | 1991 | 0.00 |
| 944 | Hebei Province | *Mus musculus* | Farmland | Trap success | 1992 | 0.08 |
| 945 | Hebei Province | *Mus musculus* | Farmland | Trap success | 1993 | 0.10 |
| 946 | Hebei Province | *Mus musculus* | Farmland | Trap success | 1994 | 0.23 |
| 947 | Hebei Province | *Mus musculus* | Farmland | Trap success | 1995 | 0.22 |
| 948 | Hebei Province | *Mus musculus* | Farmland | Trap success | 1996 | 0.12 |
| 949 | Hebei Province | *Mus musculus* | Farmland | Trap success | 1997 | 0.02 |
| 950 | Hebei Province | *Mus musculus* | Farmland | Trap success | 1998 | 0.06 |
| 951 | Hebei Province | *Mus musculus* | Farmland | Trap success | 1999 | 0.26 |
| 952 | Hebei Province | *Mus musculus* | Farmland | Trap success | 2000 | 0.18 |
| 953 | Hebei Province | *Mus musculus* | Farmland | Trap success | 2001 | 0.07 |
| 954 | Hebei Province | *Mus musculus* | Farmland | Trap success | 2002 | 0.02 |
| 955 | Hebei Province | *Mus musculus* | Farmland | Trap success | 2003 | 0.06 |
| 956 | Hebei Province | *Mus musculus* | Farmland | Trap success | 2004 | 0.03 |
| 957 | Hebei Province | *Mus musculus* | Farmland | Trap success | 2005 | 0.03 |
| 958 | Hebei Province | *Mus musculus* | Farmland | Trap success | 2006 | 0.02 |
| 959 | Hebei Province | *Mus musculus* | Farmland | Trap success | 2007 | 0.06 |
| 960 | Hebei Province | *Mus musculus* | Farmland | Trap success | 2008 | 0.00 |
| 961 | Hebei Province | *Mus musculus* | Farmland | Trap success | 2009 | 0.03 |
| 962 | Hebei Province | *Mus musculus* | Farmland | Trap success | 2010 | 0.00 |
| 963 | Hebei Province | *Rattus norvegicus* | Farmland | Trap success | 1984 | 1.00 |
| 964 | Hebei Province | *Rattus norvegicus* | Farmland | Trap success | 1985 | 0.50 |
| 965 | Hebei Province | *Rattus norvegicus* | Farmland | Trap success | 1986 | 0.93 |
| 966 | Hebei Province | *Rattus norvegicus* | Farmland | Trap success | 1987 | 0.75 |
| 967 | Hebei Province | *Rattus norvegicus* | Farmland | Trap success | 1988 | 0.32 |
| 968 | Hebei Province | *Rattus norvegicus* | Farmland | Trap success | 1989 | 0.09 |
| 969 | Hebei Province | *Rattus norvegicus* | Farmland | Trap success | 1990 | 0.00 |
| 970 | Hebei Province | *Rattus norvegicus* | Farmland | Trap success | 1991 | 0.07 |
| 971 | Hebei Province | *Rattus norvegicus* | Farmland | Trap success | 1992 | 0.07 |
| 972 | Hebei Province | *Rattus norvegicus* | Farmland | Trap success | 1993 | 0.07 |
| 973 | Hebei Province | *Rattus norvegicus* | Farmland | Trap success | 1994 | 0.06 |
| 974 | Hebei Province | *Rattus norvegicus* | Farmland | Trap success | 1995 | 0.11 |
| 975 | Hebei Province | *Rattus norvegicus* | Farmland | Trap success | 1996 | 0.02 |
| 976 | Hebei Province | *Rattus norvegicus* | Farmland | Trap success | 1997 | 0.09 |
| 977 | Hebei Province | *Rattus norvegicus* | Farmland | Trap success | 1998 | 0.23 |
| 978 | Hebei Province | *Rattus norvegicus* | Farmland | Trap success | 1999 | 0.12 |
| 979 | Hebei Province | *Rattus norvegicus* | Farmland | Trap success | 2000 | 0.02 |
| 980 | Hebei Province | *Rattus norvegicus* | Farmland | Trap success | 2001 | 0.00 |
| 981 | Hebei Province | *Rattus norvegicus* | Farmland | Trap success | 2002 | 0.01 |
| 982 | Hebei Province | *Rattus norvegicus* | Farmland | Trap success | 2003 | 0.00 |
| 983 | Hebei Province | *Rattus norvegicus* | Farmland | Trap success | 2004 | 0.01 |
| 984 | Hebei Province | *Rattus norvegicus* | Farmland | Trap success | 2005 | 0.00 |
| 985 | Hebei Province | *Rattus norvegicus* | Farmland | Trap success | 2006 | 0.00 |
| 986 | Hebei Province | *Rattus norvegicus* | Farmland | Trap success | 2007 | 0.00 |
| 987 | Hebei Province | *Rattus norvegicus* | Farmland | Trap success | 2008 | 0.00 |
| 988 | Hebei Province | *Rattus norvegicus* | Farmland | Trap success | 2009 | 0.00 |
| 989 | Hebei Province | *Rattus norvegicus* | Farmland | Trap success | 2010 | 0.00 |
| 990 | Hebei Province | *Tscherskia triton* | Farmland | Trap success | 1984 | 0.73 |
| 991 | Hebei Province | *Tscherskia triton* | Farmland | Trap success | 1985 | 0.77 |
| 992 | Hebei Province | *Tscherskia triton* | Farmland | Trap success | 1986 | 0.87 |
| 993 | Hebei Province | *Tscherskia triton* | Farmland | Trap success | 1987 | 0.50 |
| 994 | Hebei Province | *Tscherskia triton* | Farmland | Trap success | 1988 | 0.24 |
| 995 | Hebei Province | *Tscherskia triton* | Farmland | Trap success | 1989 | 0.04 |
| 996 | Hebei Province | *Tscherskia triton* | Farmland | Trap success | 1990 | 0.01 |
| 997 | Hebei Province | *Tscherskia triton* | Farmland | Trap success | 1991 | 0.02 |
| 998 | Hebei Province | *Tscherskia triton* | Farmland | Trap success | 1992 | 0.18 |
| 999 | Hebei Province | *Tscherskia triton* | Farmland | Trap success | 1993 | 1.00 |
| 1000 | Hebei Province | *Tscherskia triton* | Farmland | Trap success | 1994 | 0.61 |
| 1001 | Hebei Province | *Tscherskia triton* | Farmland | Trap success | 1995 | 0.57 |
| 1002 | Hebei Province | *Tscherskia triton* | Farmland | Trap success | 1996 | 0.88 |
| 1003 | Hebei Province | *Tscherskia triton* | Farmland | Trap success | 1997 | 0.54 |
| 1004 | Hebei Province | *Tscherskia triton* | Farmland | Trap success | 1998 | 0.30 |
| 1005 | Hebei Province | *Tscherskia triton* | Farmland | Trap success | 1999 | 0.33 |
| 1006 | Hebei Province | *Tscherskia triton* | Farmland | Trap success | 2000 | 0.16 |
| 1007 | Hebei Province | *Tscherskia triton* | Farmland | Trap success | 2001 | 0.07 |
| 1008 | Hebei Province | *Tscherskia triton* | Farmland | Trap success | 2002 | 0.01 |
| 1009 | Hebei Province | *Tscherskia triton* | Farmland | Trap success | 2003 | 0.01 |
| 1010 | Hebei Province | *Tscherskia triton* | Farmland | Trap success | 2004 | 0.05 |
| 1011 | Hebei Province | *Tscherskia triton* | Farmland | Trap success | 2005 | 0.01 |
| 1012 | Hebei Province | *Tscherskia triton* | Farmland | Trap success | 2006 | 0.00 |
| 1013 | Hebei Province | *Tscherskia triton* | Farmland | Trap success | 2007 | 0.00 |
| 1014 | Hebei Province | *Tscherskia triton* | Farmland | Trap success | 2008 | 0.00 |
| 1015 | Hebei Province | *Tscherskia triton* | Farmland | Trap success | 2009 | 0.00 |
| 1016 | Hebei Province | *Tscherskia triton* | Farmland | Trap success | 2010 | 0.00 |
| 1017 | Hebei Province | *Tscherskia triton* | Farmland | Trap success | 2011 | 0.00 |
| 1018 | Hebei Province | *Tscherskia triton* | Farmland | Trap success | 2012 | 0.00 |
| 1019 | Hebei Province | *Tscherskia triton* | Farmland | Trap success | 2013 | 0.00 |
| 1020 | Hebei Province | *Tscherskia triton* | Farmland | Trap success | 2014 | 0.00 |
| 1021 | Hebei Province | *Tscherskia triton* | Farmland | Trap success | 2015 | 0.00 |
| 1022 | Hebei Province | *Tscherskia triton* | Farmland | Trap success | 2016 | 0.00 |
| 1023 | Hebei Province | *Apodemus agrarius* | Farmland | Trap success | 1992 | 1.00 |
| 1024 | Hebei Province | *Apodemus agrarius* | Farmland | Trap success | 1993 | 0.37 |
| 1025 | Hebei Province | *Apodemus agrarius* | Farmland | Trap success | 1994 | 0.12 |
| 1026 | Hebei Province | *Apodemus agrarius* | Farmland | Trap success | 1995 | 0.00 |
| 1027 | Hebei Province | *Apodemus agrarius* | Farmland | Trap success | 1996 | 0.00 |
| 1028 | Hebei Province | *Apodemus agrarius* | Farmland | Trap success | 1997 | 0.00 |
| 1029 | Hebei Province | *Apodemus agrarius* | Farmland | Trap success | 1998 | 0.11 |
| 1030 | Hebei Province | *Apodemus agrarius* | Farmland | Trap success | 1999 | 0.10 |
| 1031 | Hebei Province | *Apodemus agrarius* | Farmland | Trap success | 2000 | 0.00 |
| 1032 | Hebei Province | *Apodemus agrarius* | Farmland | Trap success | 2001 | 0.05 |
| 1033 | Hebei Province | *Apodemus agrarius* | Farmland | Trap success | 2002 | 0.08 |
| 1034 | Hebei Province | *Apodemus agrarius* | Farmland | Trap success | 2003 | 0.00 |
| 1035 | Hebei Province | *Apodemus agrarius* | Farmland | Trap success | 2004 | 0.66 |
| 1036 | Hebei Province | *Apodemus agrarius* | Farmland | Trap success | 2005 | 0.39 |
| 1037 | Hebei Province | *Apodemus agrarius* | Farmland | Trap success | 2006 | 0.26 |
| 1038 | Hebei Province | *Apodemus agrarius* | Farmland | Trap success | 2007 | 0.24 |
| 1039 | Hebei Province | *Apodemus agrarius* | Farmland | Trap success | 2008 | 0.89 |
| 1040 | Hebei Province | *Apodemus agrarius* | Farmland | Trap success | 2009 | 0.25 |
| 1041 | Hebei Province | *Apodemus agrarius* | Farmland | Trap success | 2010 | 0.21 |
| 1042 | Hebei Province | *Apodemus agrarius* | Farmland | Trap success | 2011 | 0.22 |
| 1043 | Hebei Province | *Apodemus agrarius* | Farmland | Trap success | 2012 | 0.78 |
| 1044 | Hebei Province | *Apodemus agrarius* | Farmland | Trap success | 2013 | 1.00 |
| 1045 | Hebei Province | *Apodemus agrarius* | Farmland | Trap success | 2014 | 0.56 |
| 1046 | Hebei Province | *Apodemus agrarius* | Farmland | Trap success | 2015 | 0.00 |
| 1047 | Hebei Province | *Cricetulus barabensis* | Farmland | Trap success | 1992 | 1.00 |
| 1048 | Hebei Province | *Cricetulus barabensis* | Farmland | Trap success | 1993 | 0.19 |
| 1049 | Hebei Province | *Cricetulus barabensis* | Farmland | Trap success | 1994 | 0.10 |
| 1050 | Hebei Province | *Cricetulus barabensis* | Farmland | Trap success | 1995 | 0.00 |
| 1051 | Hebei Province | *Cricetulus barabensis* | Farmland | Trap success | 1996 | 0.00 |
| 1052 | Hebei Province | *Cricetulus barabensis* | Farmland | Trap success | 1997 | 0.00 |
| 1053 | Hebei Province | *Cricetulus barabensis* | Farmland | Trap success | 1998 | 0.02 |
| 1054 | Hebei Province | *Cricetulus barabensis* | Farmland | Trap success | 1999 | 0.01 |
| 1055 | Hebei Province | *Cricetulus barabensis* | Farmland | Trap success | 2000 | 0.00 |
| 1056 | Hebei Province | *Cricetulus barabensis* | Farmland | Trap success | 2001 | 0.00 |
| 1057 | Hebei Province | *Cricetulus barabensis* | Farmland | Trap success | 2002 | 0.00 |
| 1058 | Hebei Province | *Cricetulus barabensis* | Farmland | Trap success | 2003 | 0.13 |
| 1059 | Hebei Province | *Cricetulus barabensis* | Farmland | Trap success | 2004 | 0.02 |
| 1060 | Hebei Province | *Cricetulus barabensis* | Farmland | Trap success | 2005 | 0.02 |
| 1061 | Hebei Province | *Cricetulus barabensis* | Farmland | Trap success | 2006 | 0.32 |
| 1062 | Hebei Province | *Cricetulus barabensis* | Farmland | Trap success | 2007 | 0.44 |
| 1063 | Hebei Province | *Cricetulus barabensis* | Farmland | Trap success | 2008 | 0.17 |
| 1064 | Hebei Province | *Cricetulus barabensis* | Farmland | Trap success | 2009 | 0.66 |
| 1065 | Hebei Province | *Cricetulus barabensis* | Farmland | Trap success | 2010 | 0.39 |
| 1066 | Hebei Province | *Cricetulus barabensis* | Farmland | Trap success | 2011 | 0.31 |
| 1067 | Hebei Province | *Cricetulus barabensis* | Farmland | Trap success | 2012 | 0.07 |
| 1068 | Hebei Province | *Cricetulus barabensis* | Farmland | Trap success | 2013 | 0.10 |
| 1069 | Hebei Province | *Cricetulus barabensis* | Farmland | Trap success | 2014 | 0.16 |
| 1070 | Hebei Province | *Cricetulus barabensis* | Farmland | Trap success | 2015 | 0.00 |
| 1071 | Hebei Province | *Mus musculus* | Farmland | Trap success | 1992 | 1.00 |
| 1072 | Hebei Province | *Mus musculus* | Farmland | Trap success | 1993 | 0.61 |
| 1073 | Hebei Province | *Mus musculus* | Farmland | Trap success | 1994 | 0.11 |
| 1074 | Hebei Province | *Mus musculus* | Farmland | Trap success | 1995 | 0.00 |
| 1075 | Hebei Province | *Mus musculus* | Farmland | Trap success | 1996 | 0.00 |
| 1076 | Hebei Province | *Mus musculus* | Farmland | Trap success | 1997 | 0.00 |
| 1077 | Hebei Province | *Mus musculus* | Farmland | Trap success | 1998 | 0.35 |
| 1078 | Hebei Province | *Mus musculus* | Farmland | Trap success | 1999 | 0.28 |
| 1079 | Hebei Province | *Mus musculus* | Farmland | Trap success | 2000 | 0.34 |
| 1080 | Hebei Province | *Mus musculus* | Farmland | Trap success | 2001 | 0.07 |
| 1081 | Hebei Province | *Mus musculus* | Farmland | Trap success | 2002 | 0.00 |
| 1082 | Hebei Province | *Mus musculus* | Farmland | Trap success | 2003 | 0.00 |
| 1083 | Hebei Province | *Mus musculus* | Farmland | Trap success | 2004 | 0.07 |
| 1084 | Hebei Province | *Mus musculus* | Farmland | Trap success | 2005 | 0.07 |
| 1085 | Hebei Province | *Mus musculus* | Farmland | Trap success | 2006 | 0.55 |
| 1086 | Hebei Province | *Mus musculus* | Farmland | Trap success | 2007 | 0.48 |
| 1087 | Hebei Province | *Mus musculus* | Farmland | Trap success | 2008 | 0.00 |
| 1088 | Hebei Province | *Mus musculus* | Farmland | Trap success | 2009 | 0.14 |
| 1089 | Hebei Province | *Mus musculus* | Farmland | Trap success | 2010 | 0.00 |
| 1090 | Hebei Province | *Mus musculus* | Farmland | Trap success | 2011 | 0.00 |
| 1091 | Hebei Province | *Mus musculus* | Farmland | Trap success | 2012 | 0.00 |
| 1092 | Hebei Province | *Mus musculus* | Farmland | Trap success | 2013 | 0.00 |
| 1093 | Hebei Province | *Mus musculus* | Farmland | Trap success | 2014 | 0.14 |
| 1094 | Hebei Province | *Mus musculus* | Farmland | Trap success | 2015 | 0.00 |
| 1095 | Hebei Province | *Tscherskia triton* | Farmland | Trap success | 1992 | 0.15 |
| 1096 | Hebei Province | *Tscherskia triton* | Farmland | Trap success | 1993 | 0.60 |
| 1097 | Hebei Province | *Tscherskia triton* | Farmland | Trap success | 1994 | 0.68 |
| 1098 | Hebei Province | *Tscherskia triton* | Farmland | Trap success | 1995 | 0.53 |
| 1099 | Hebei Province | *Tscherskia triton* | Farmland | Trap success | 1996 | 0.91 |
| 1100 | Hebei Province | *Tscherskia triton* | Farmland | Trap success | 1997 | 1.00 |
| 1101 | Hebei Province | *Tscherskia triton* | Farmland | Trap success | 1998 | 0.55 |
| 1102 | Hebei Province | *Tscherskia triton* | Farmland | Trap success | 1999 | 0.62 |
| 1103 | Hebei Province | *Tscherskia triton* | Farmland | Trap success | 2000 | 0.30 |
| 1104 | Hebei Province | *Tscherskia triton* | Farmland | Trap success | 2001 | 0.29 |
| 1105 | Hebei Province | *Tscherskia triton* | Farmland | Trap success | 2002 | 0.45 |
| 1106 | Hebei Province | *Tscherskia triton* | Farmland | Trap success | 2003 | 0.70 |
| 1107 | Hebei Province | *Tscherskia triton* | Farmland | Trap success | 2004 | 0.32 |
| 1108 | Hebei Province | *Tscherskia triton* | Farmland | Trap success | 2005 | 0.21 |
| 1109 | Hebei Province | *Tscherskia triton* | Farmland | Trap success | 2006 | 0.14 |
| 1110 | Hebei Province | *Tscherskia triton* | Farmland | Trap success | 2007 | 0.05 |
| 1111 | Hebei Province | *Tscherskia triton* | Farmland | Trap success | 2008 | 0.01 |
| 1112 | Hebei Province | *Tscherskia triton* | Farmland | Trap success | 2009 | 0.01 |
| 1113 | Hebei Province | *Tscherskia triton* | Farmland | Trap success | 2010 | 0.05 |
| 1114 | Hebei Province | *Tscherskia triton* | Farmland | Trap success | 2011 | 0.06 |
| 1115 | Hebei Province | *Tscherskia triton* | Farmland | Trap success | 2012 | 0.01 |
| 1116 | Hebei Province | *Tscherskia triton* | Farmland | Trap success | 2013 | 0.02 |
| 1117 | Hebei Province | *Tscherskia triton* | Farmland | Trap success | 2014 | 0.01 |
| 1118 | Hebei Province | *Tscherskia triton* | Farmland | Trap success | 2015 | 0.00 |
| 1119 | Hebei Province | rodent | City | Trap success | 1988 | 1.00 |
| 1120 | Hebei Province | rodent | City | Trap success | 1991 | 0.66 |
| 1121 | Hebei Province | rodent | City | Trap success | 1992 | 0.70 |
| 1122 | Hebei Province | rodent | City | Trap success | 1993 | 0.25 |
| 1123 | Hebei Province | rodent | City | Trap success | 1994 | 0.67 |
| 1124 | Hebei Province | rodent | City | Trap success | 1995 | 0.35 |
| 1125 | Hebei Province | rodent | City | Trap success | 1996 | 0.56 |
| 1126 | Hebei Province | rodent | City | Trap success | 1997 | 0.42 |
| 1127 | Hebei Province | rodent | City | Trap success | 1998 | 0.58 |
| 1128 | Hebei Province | rodent | City | Trap success | 1999 | 0.49 |
| 1129 | Hebei Province | rodent | City | Trap success | 2000 | 0.17 |
| 1130 | Hebei Province | rodent | City | Trap success | 2001 | 0.29 |
| 1131 | Hebei Province | rodent | City | Trap success | 2002 | 0.22 |
| 1132 | Hebei Province | rodent | City | Trap success | 2003 | 0.18 |
| 1133 | Hebei Province | rodent | City | Trap success | 2004 | 0.36 |
| 1134 | Hebei Province | rodent | City | Trap success | 2005 | 0.20 |
| 1135 | Hebei Province | rodent | City | Trap success | 2006 | 0.00 |
| 1136 | Hebei Province | rodent | City | Trap success | 2007 | 0.09 |
| 1137 | Hebei Province | rodent | City | Trap success | 2008 | 0.00 |
| 1138 | Hebei Province | rodent | City | Trap success | 2009 | 0.01 |
| 1139 | Hebei Province | rodent | City | Trap success | 1984 | 1.00 |
| 1140 | Hebei Province | rodent | City | Trap success | 1985 | 0.93 |
| 1141 | Hebei Province | rodent | City | Trap success | 1986 | 0.19 |
| 1142 | Hebei Province | rodent | City | Trap success | 1987 | 0.08 |
| 1143 | Hebei Province | rodent | City | Trap success | 1988 | 0.00 |
| 1144 | Hebei Province | rodent | City | Trap success | 1989 | 0.00 |
| 1145 | Hebei Province | rodent | City | Trap success | 1990 | 0.03 |
| 1146 | Hebei Province | rodent | City | Trap success | 1991 | 0.11 |
| 1147 | Hebei Province | rodent | City | Trap success | 1992 | 0.44 |
| 1148 | Hebei Province | rodent | City | Trap success | 1993 | 0.37 |
| 1149 | Hebei Province | rodent | City | Trap success | 1994 | 0.48 |
| 1150 | Hebei Province | rodent | City | Trap success | 1995 | 0.30 |
| 1151 | Hebei Province | rodent | City | Trap success | 1996 | 0.60 |
| 1152 | Hebei Province | rodent | City | Trap success | 1997 | 0.16 |
| 1153 | Hebei Province | rodent | City | Trap success | 1998 | 0.31 |
| 1154 | Hebei Province | rodent | City | Trap success | 1999 | 0.59 |
| 1155 | Hebei Province | rodent | City | Trap success | 2000 | 0.29 |
| 1156 | Hebei Province | rodent | City | Trap success | 2001 | 0.16 |
| 1157 | Hebei Province | rodent | City | Trap success | 2002 | 0.17 |
| 1158 | Hebei Province | rodent | City | Trap success | 2003 | 0.07 |
| 1159 | Hebei Province | rodent | City | Trap success | 2004 | 0.10 |
| 1160 | Hebei Province | rodent | City | Trap success | 2005 | 0.02 |
| 1161 | Hebei Province | rodent | Farmland | Trap success | 1984 | 1.00 |
| 1162 | Hebei Province | rodent | Farmland | Trap success | 1985 | 0.39 |
| 1163 | Hebei Province | rodent | Farmland | Trap success | 1986 | 0.24 |
| 1164 | Hebei Province | rodent | Farmland | Trap success | 1987 | 0.00 |
| 1165 | Hebei Province | rodent | Farmland | Trap success | 1988 | 0.09 |
| 1166 | Hebei Province | rodent | Farmland | Trap success | 1989 | 0.11 |
| 1167 | Hebei Province | rodent | Farmland | Trap success | 1990 | 0.07 |
| 1168 | Hebei Province | rodent | Farmland | Trap success | 1991 | 0.30 |
| 1169 | Hebei Province | rodent | Farmland | Trap success | 1992 | 0.35 |
| 1170 | Hebei Province | rodent | Farmland | Trap success | 1993 | 0.19 |
| 1171 | Hebei Province | rodent | Farmland | Trap success | 1994 | 0.21 |
| 1172 | Hebei Province | rodent | Farmland | Trap success | 1995 | 0.30 |
| 1173 | Hebei Province | rodent | Farmland | Trap success | 1996 | 0.10 |
| 1174 | Hebei Province | rodent | Farmland | Trap success | 1997 | 0.06 |
| 1175 | Hebei Province | rodent | Farmland | Trap success | 1998 | 0.21 |
| 1176 | Hebei Province | rodent | Farmland | Trap success | 1999 | 0.39 |
| 1177 | Hebei Province | rodent | Farmland | Trap success | 2000 | 0.16 |
| 1178 | Hebei Province | rodent | Farmland | Trap success | 2001 | 0.12 |
| 1179 | Hebei Province | rodent | Farmland | Trap success | 2002 | 0.06 |
| 1180 | Hebei Province | rodent | Farmland | Trap success | 2003 | 0.08 |
| 1181 | Hebei Province | rodent | Farmland | Trap success | 2004 | 0.06 |
| 1182 | Hebei Province | rodent | Farmland | Trap success | 2005 | 0.03 |
| 1183 | Hebei Province | *Meriones unguiculatus* | Farmland | Population density | 1992 | 0.40 |
| 1184 | Hebei Province | *Meriones unguiculatus* | Farmland | Population density | 1993 | 0.32 |
| 1185 | Hebei Province | *Meriones unguiculatus* | Farmland | Population density | 1994 | 0.25 |
| 1186 | Hebei Province | *Meriones unguiculatus* | Farmland | Population density | 1995 | 0.42 |
| 1187 | Hebei Province | *Meriones unguiculatus* | Farmland | Population density | 1996 | 0.40 |
| 1188 | Hebei Province | *Meriones unguiculatus* | Farmland | Population density | 1997 | 1.00 |
| 1189 | Hebei Province | *Meriones unguiculatus* | Farmland | Population density | 1998 | 0.73 |
| 1190 | Hebei Province | *Meriones unguiculatus* | Farmland | Population density | 1999 | 0.62 |
| 1191 | Hebei Province | *Meriones unguiculatus* | Farmland | Population density | 2000 | 0.53 |
| 1192 | Hebei Province | *Meriones unguiculatus* | Farmland | Population density | 2001 | 0.97 |
| 1193 | Hebei Province | *Meriones unguiculatus* | Farmland | Population density | 2002 | 0.77 |
| 1194 | Hebei Province | *Meriones unguiculatus* | Farmland | Population density | 2003 | 0.70 |
| 1195 | Hebei Province | *Meriones unguiculatus* | Farmland | Population density | 2004 | 0.27 |
| 1196 | Hebei Province | *Meriones unguiculatus* | Farmland | Population density | 2005 | 0.36 |
| 1197 | Hebei Province | *Meriones unguiculatus* | Farmland | Population density | 2006 | 0.00 |
| 1198 | Hebei Province | *Meriones unguiculatus* | Farmland | Population density | 2007 | 0.09 |
| 1199 | Hebei Province | *Meriones unguiculatus* | Farmland | Population density | 2008 | 0.00 |
| 1200 | Hebei Province | *Meriones unguiculatus* | Farmland | Population density | 2009 | 0.00 |
| 1201 | Hebei Province | *Meriones unguiculatus* | Farmland | Population density | 2010 | 0.00 |
| 1202 | Hebei Province | *Meriones unguiculatus* | Farmland | Population density | 2011 | 0.00 |
| 1203 | Hebei Province | *Spermophilus dauricus* | Grassland – farmland mosaic | Population density | 1992 | 0.00 |
| 1204 | Hebei Province | *Spermophilus dauricus* | Grassland – farmland mosaic | Population density | 1993 | 0.00 |
| 1205 | Hebei Province | *Spermophilus dauricus* | Grassland – farmland mosaic | Population density | 1994 | 0.01 |
| 1206 | Hebei Province | *Spermophilus dauricus* | Grassland – farmland mosaic | Population density | 1995 | 0.00 |
| 1207 | Hebei Province | *Spermophilus dauricus* | Grassland – farmland mosaic | Population density | 1996 | 0.00 |
| 1208 | Hebei Province | *Spermophilus dauricus* | Grassland – farmland mosaic | Population density | 1997 | 0.00 |
| 1209 | Hebei Province | *Spermophilus dauricus* | Grassland – farmland mosaic | Population density | 1998 | 0.19 |
| 1210 | Hebei Province | *Spermophilus dauricus* | Grassland – farmland mosaic | Population density | 1999 | 0.00 |
| 1211 | Hebei Province | *Spermophilus dauricus* | Grassland – farmland mosaic | Population density | 2000 | 0.07 |
| 1212 | Hebei Province | *Spermophilus dauricus* | Grassland – farmland mosaic | Population density | 2001 | 0.07 |
| 1213 | Hebei Province | *Spermophilus dauricus* | Grassland – farmland mosaic | Population density | 2002 | 0.09 |
| 1214 | Hebei Province | *Spermophilus dauricus* | Grassland – farmland mosaic | Population density | 2003 | 0.24 |
| 1215 | Hebei Province | *Spermophilus dauricus* | Grassland – farmland mosaic | Population density | 2004 | 0.65 |
| 1216 | Hebei Province | *Spermophilus dauricus* | Grassland – farmland mosaic | Population density | 2005 | 0.57 |
| 1217 | Hebei Province | *Spermophilus dauricus* | Grassland – farmland mosaic | Population density | 2006 | 0.66 |
| 1218 | Hebei Province | *Spermophilus dauricus* | Grassland – farmland mosaic | Population density | 2007 | 0.71 |
| 1219 | Hebei Province | *Spermophilus dauricus* | Grassland – farmland mosaic | Population density | 2008 | 1.00 |
| 1220 | Hebei Province | *Spermophilus dauricus* | Grassland – farmland mosaic | Population density | 2009 | 0.62 |
| 1221 | Hebei Province | *Spermophilus dauricus* | Grassland – farmland mosaic | Population density | 2010 | 0.14 |
| 1222 | Hebei Province | *Spermophilus dauricus* | Grassland – farmland mosaic | Population density | 2011 | 0.28 |
| 1223 | Hebei Province | *Allactaga sibirica* | Grassland – farmland mosaic | Trap success | 1990 | 0.41 |
| 1224 | Hebei Province | *Allactaga sibirica* | Grassland – farmland mosaic | Trap success | 1991 | 0.48 |
| 1225 | Hebei Province | *Allactaga sibirica* | Grassland – farmland mosaic | Trap success | 1992 | 0.19 |
| 1226 | Hebei Province | *Allactaga sibirica* | Grassland – farmland mosaic | Trap success | 1993 | 0.48 |
| 1227 | Hebei Province | *Allactaga sibirica* | Grassland – farmland mosaic | Trap success | 1994 | 0.11 |
| 1228 | Hebei Province | *Allactaga sibirica* | Grassland – farmland mosaic | Trap success | 1995 | 0.15 |
| 1229 | Hebei Province | *Allactaga sibirica* | Grassland – farmland mosaic | Trap success | 1996 | 0.00 |
| 1230 | Hebei Province | *Allactaga sibirica* | Grassland – farmland mosaic | Trap success | 1997 | 0.00 |
| 1231 | Hebei Province | *Allactaga sibirica* | Grassland – farmland mosaic | Trap success | 1998 | 0.00 |
| 1232 | Hebei Province | *Allactaga sibirica* | Grassland – farmland mosaic | Trap success | 1999 | 0.00 |
| 1233 | Hebei Province | *Allactaga sibirica* | Grassland – farmland mosaic | Trap success | 2000 | 0.00 |
| 1234 | Hebei Province | *Allactaga sibirica* | Grassland – farmland mosaic | Trap success | 2001 | 0.00 |
| 1235 | Hebei Province | *Allactaga sibirica* | Grassland – farmland mosaic | Trap success | 2002 | 0.56 |
| 1236 | Hebei Province | *Allactaga sibirica* | Grassland – farmland mosaic | Trap success | 2003 | 0.00 |
| 1237 | Hebei Province | *Allactaga sibirica* | Grassland – farmland mosaic | Trap success | 2004 | 0.26 |
| 1238 | Hebei Province | *Allactaga sibirica* | Grassland – farmland mosaic | Trap success | 2005 | 1.00 |
| 1239 | Hebei Province | *Allactaga sibirica* | Grassland – farmland mosaic | Trap success | 2006 | 0.00 |
| 1240 | Hebei Province | *Allactaga sibirica* | Grassland – farmland mosaic | Trap success | 2007 | 0.11 |
| 1241 | Hebei Province | *Allactaga sibirica* | Grassland – farmland mosaic | Trap success | 2008 | 0.41 |
| 1242 | Hebei Province | *Allactaga sibirica* | Grassland – farmland mosaic | Trap success | 2009 | 0.22 |
| 1243 | Hebei Province | *Allactaga sibirica* | Grassland – farmland mosaic | Trap success | 2010 | 0.30 |
| 1244 | Hebei Province | *Cricetulus barabensis* | Grassland – farmland mosaic | Trap success | 1990 | 0.17 |
| 1245 | Hebei Province | *Cricetulus barabensis* | Grassland – farmland mosaic | Trap success | 1991 | 0.93 |
| 1246 | Hebei Province | *Cricetulus barabensis* | Grassland – farmland mosaic | Trap success | 1992 | 0.05 |
| 1247 | Hebei Province | *Cricetulus barabensis* | Grassland – farmland mosaic | Trap success | 1993 | 1.00 |
| 1248 | Hebei Province | *Cricetulus barabensis* | Grassland – farmland mosaic | Trap success | 1994 | 0.25 |
| 1249 | Hebei Province | *Cricetulus barabensis* | Grassland – farmland mosaic | Trap success | 1995 | 0.30 |
| 1250 | Hebei Province | *Cricetulus barabensis* | Grassland – farmland mosaic | Trap success | 1996 | 0.16 |
| 1251 | Hebei Province | *Cricetulus barabensis* | Grassland – farmland mosaic | Trap success | 1997 | 0.21 |
| 1252 | Hebei Province | *Cricetulus barabensis* | Grassland – farmland mosaic | Trap success | 1998 | 0.23 |
| 1253 | Hebei Province | *Cricetulus barabensis* | Grassland – farmland mosaic | Trap success | 1999 | 0.86 |
| 1254 | Hebei Province | *Cricetulus barabensis* | Grassland – farmland mosaic | Trap success | 2000 | 0.40 |
| 1255 | Hebei Province | *Cricetulus barabensis* | Grassland – farmland mosaic | Trap success | 2001 | 0.38 |
| 1256 | Hebei Province | *Cricetulus barabensis* | Grassland – farmland mosaic | Trap success | 2002 | 0.00 |
| 1257 | Hebei Province | *Cricetulus barabensis* | Grassland – farmland mosaic | Trap success | 2003 | 0.35 |
| 1258 | Hebei Province | *Cricetulus barabensis* | Grassland – farmland mosaic | Trap success | 2004 | 0.78 |
| 1259 | Hebei Province | *Cricetulus barabensis* | Grassland – farmland mosaic | Trap success | 2005 | 0.43 |
| 1260 | Hebei Province | *Cricetulus barabensis* | Grassland – farmland mosaic | Trap success | 2006 | 0.11 |
| 1261 | Hebei Province | *Cricetulus barabensis* | Grassland – farmland mosaic | Trap success | 2007 | 0.37 |
| 1262 | Hebei Province | *Cricetulus barabensis* | Grassland – farmland mosaic | Trap success | 2008 | 0.01 |
| 1263 | Hebei Province | *Cricetulus barabensis* | Grassland – farmland mosaic | Trap success | 2009 | 0.35 |
| 1264 | Hebei Province | *Cricetulus barabensis* | Grassland – farmland mosaic | Trap success | 2010 | 0.00 |
| 1265 | Hebei Province | *Meriones unguiculatus* | Grassland – farmland mosaic | Trap success | 1990 | 0.00 |
| 1266 | Hebei Province | *Meriones unguiculatus* | Grassland – farmland mosaic | Trap success | 1991 | 0.19 |
| 1267 | Hebei Province | *Meriones unguiculatus* | Grassland – farmland mosaic | Trap success | 1992 | 0.70 |
| 1268 | Hebei Province | *Meriones unguiculatus* | Grassland – farmland mosaic | Trap success | 1993 | 0.35 |
| 1269 | Hebei Province | *Meriones unguiculatus* | Grassland – farmland mosaic | Trap success | 1994 | 0.49 |
| 1270 | Hebei Province | *Meriones unguiculatus* | Grassland – farmland mosaic | Trap success | 1995 | 0.35 |
| 1271 | Hebei Province | *Meriones unguiculatus* | Grassland – farmland mosaic | Trap success | 1996 | 0.43 |
| 1272 | Hebei Province | *Meriones unguiculatus* | Grassland – farmland mosaic | Trap success | 1997 | 0.19 |
| 1273 | Hebei Province | *Meriones unguiculatus* | Grassland – farmland mosaic | Trap success | 1998 | 0.00 |
| 1274 | Hebei Province | *Meriones unguiculatus* | Grassland – farmland mosaic | Trap success | 1999 | 0.08 |
| 1275 | Hebei Province | *Meriones unguiculatus* | Grassland – farmland mosaic | Trap success | 2000 | 0.38 |
| 1276 | Hebei Province | *Meriones unguiculatus* | Grassland – farmland mosaic | Trap success | 2001 | 0.19 |
| 1277 | Hebei Province | *Meriones unguiculatus* | Grassland – farmland mosaic | Trap success | 2002 | 1.00 |
| 1278 | Hebei Province | *Meriones unguiculatus* | Grassland – farmland mosaic | Trap success | 2003 | 0.11 |
| 1279 | Hebei Province | *Meriones unguiculatus* | Grassland – farmland mosaic | Trap success | 2004 | 0.30 |
| 1280 | Hebei Province | *Meriones unguiculatus* | Grassland – farmland mosaic | Trap success | 2005 | 0.35 |
| 1281 | Hebei Province | *Meriones unguiculatus* | Grassland – farmland mosaic | Trap success | 2006 | 0.00 |
| 1282 | Hebei Province | *Meriones unguiculatus* | Grassland – farmland mosaic | Trap success | 2007 | 0.00 |
| 1283 | Hebei Province | *Meriones unguiculatus* | Grassland – farmland mosaic | Trap success | 2008 | 0.05 |
| 1284 | Hebei Province | *Meriones unguiculatus* | Grassland – farmland mosaic | Trap success | 2009 | 0.00 |
| 1285 | Hebei Province | *Meriones unguiculatus* | Grassland – farmland mosaic | Trap success | 2010 | 0.00 |
| 1286 | Hebei Province | *Phodopus sungorus* | Grassland – farmland mosaic | Trap success | 1990 | 0.04 |
| 1287 | Hebei Province | *Phodopus sungorus* | Grassland – farmland mosaic | Trap success | 1991 | 1.00 |
| 1288 | Hebei Province | *Phodopus sungorus* | Grassland – farmland mosaic | Trap success | 1992 | 0.00 |
| 1289 | Hebei Province | *Phodopus sungorus* | Grassland – farmland mosaic | Trap success | 1993 | 0.13 |
| 1290 | Hebei Province | *Phodopus sungorus* | Grassland – farmland mosaic | Trap success | 1994 | 0.19 |
| 1291 | Hebei Province | *Phodopus sungorus* | Grassland – farmland mosaic | Trap success | 1995 | 0.12 |
| 1292 | Hebei Province | *Phodopus sungorus* | Grassland – farmland mosaic | Trap success | 1996 | 0.09 |
| 1293 | Hebei Province | *Phodopus sungorus* | Grassland – farmland mosaic | Trap success | 1997 | 0.01 |
| 1294 | Hebei Province | *Phodopus sungorus* | Grassland – farmland mosaic | Trap success | 1998 | 0.03 |
| 1295 | Hebei Province | *Phodopus sungorus* | Grassland – farmland mosaic | Trap success | 1999 | 0.15 |
| 1296 | Hebei Province | *Phodopus sungorus* | Grassland – farmland mosaic | Trap success | 2000 | 0.02 |
| 1297 | Hebei Province | *Phodopus sungorus* | Grassland – farmland mosaic | Trap success | 2001 | 0.11 |
| 1298 | Hebei Province | *Phodopus sungorus* | Grassland – farmland mosaic | Trap success | 2002 | 0.13 |
| 1299 | Hebei Province | *Phodopus sungorus* | Grassland – farmland mosaic | Trap success | 2003 | 0.12 |
| 1300 | Hebei Province | *Phodopus sungorus* | Grassland – farmland mosaic | Trap success | 2004 | 0.39 |
| 1301 | Hebei Province | *Phodopus sungorus* | Grassland – farmland mosaic | Trap success | 2005 | 0.03 |
| 1302 | Hebei Province | *Phodopus sungorus* | Grassland – farmland mosaic | Trap success | 2006 | 0.10 |
| 1303 | Hebei Province | *Phodopus sungorus* | Grassland – farmland mosaic | Trap success | 2007 | 0.01 |
| 1304 | Hebei Province | *Phodopus sungorus* | Grassland – farmland mosaic | Trap success | 2008 | 0.01 |
| 1305 | Hebei Province | *Phodopus sungorus* | Grassland – farmland mosaic | Trap success | 2009 | 0.03 |
| 1306 | Hebei Province | *Phodopus sungorus* | Grassland – farmland mosaic | Trap success | 2010 | 0.01 |
| 1307 | Hebei Province | *Meriones unguiculatus* | Grassland – farmland mosaic | Population density | 1990 | 0.01 |
| 1308 | Hebei Province | *Meriones unguiculatus* | Grassland – farmland mosaic | Population density | 1991 | 0.15 |
| 1309 | Hebei Province | *Meriones unguiculatus* | Grassland – farmland mosaic | Population density | 1992 | 0.05 |
| 1310 | Hebei Province | *Meriones unguiculatus* | Grassland – farmland mosaic | Population density | 1993 | 0.33 |
| 1311 | Hebei Province | *Meriones unguiculatus* | Grassland – farmland mosaic | Population density | 1994 | 0.46 |
| 1312 | Hebei Province | *Meriones unguiculatus* | Grassland – farmland mosaic | Population density | 1995 | 0.40 |
| 1313 | Hebei Province | *Meriones unguiculatus* | Grassland – farmland mosaic | Population density | 1996 | 0.27 |
| 1314 | Hebei Province | *Meriones unguiculatus* | Grassland – farmland mosaic | Population density | 1997 | 0.26 |
| 1315 | Hebei Province | *Meriones unguiculatus* | Grassland – farmland mosaic | Population density | 1998 | 0.19 |
| 1316 | Hebei Province | *Meriones unguiculatus* | Grassland – farmland mosaic | Population density | 1999 | 0.16 |
| 1317 | Hebei Province | *Meriones unguiculatus* | Grassland – farmland mosaic | Population density | 2000 | 0.12 |
| 1318 | Hebei Province | *Meriones unguiculatus* | Grassland – farmland mosaic | Population density | 2001 | 0.61 |
| 1319 | Hebei Province | *Meriones unguiculatus* | Grassland – farmland mosaic | Population density | 2002 | 1.00 |
| 1320 | Hebei Province | *Meriones unguiculatus* | Grassland – farmland mosaic | Population density | 2003 | 0.48 |
| 1321 | Hebei Province | *Meriones unguiculatus* | Grassland – farmland mosaic | Population density | 2004 | 0.86 |
| 1322 | Hebei Province | *Meriones unguiculatus* | Grassland – farmland mosaic | Population density | 2005 | 0.26 |
| 1323 | Hebei Province | *Meriones unguiculatus* | Grassland – farmland mosaic | Population density | 2006 | 0.04 |
| 1324 | Hebei Province | *Meriones unguiculatus* | Grassland – farmland mosaic | Population density | 2007 | 0.02 |
| 1325 | Hebei Province | *Meriones unguiculatus* | Grassland – farmland mosaic | Population density | 2008 | 0.01 |
| 1326 | Hebei Province | *Meriones unguiculatus* | Grassland – farmland mosaic | Population density | 2009 | 0.00 |
| 1327 | Hebei Province | *Meriones unguiculatus* | Grassland – farmland mosaic | Population density | 2010 | 0.00 |
| 1328 | Hebei Province | *Meriones unguiculatus* | Grassland – farmland mosaic | Population density | 2011 | 0.01 |
| 1329 | Hebei Province | *Meriones unguiculatus* | Grassland – farmland mosaic | Population density | 2012 | 0.05 |
| 1330 | Hebei Province | *Meriones unguiculatus* | Grassland – farmland mosaic | Population density | 2013 | 0.13 |
| 1331 | Hebei Province | *Spermophilus dauricus* | Grassland – farmland mosaic | Population density | 1990 | 0.07 |
| 1332 | Hebei Province | *Spermophilus dauricus* | Grassland – farmland mosaic | Population density | 1991 | 0.00 |
| 1333 | Hebei Province | *Spermophilus dauricus* | Grassland – farmland mosaic | Population density | 1992 | 0.11 |
| 1334 | Hebei Province | *Spermophilus dauricus* | Grassland – farmland mosaic | Population density | 1993 | 0.29 |
| 1335 | Hebei Province | *Spermophilus dauricus* | Grassland – farmland mosaic | Population density | 1994 | 0.43 |
| 1336 | Hebei Province | *Spermophilus dauricus* | Grassland – farmland mosaic | Population density | 1995 | 0.20 |
| 1337 | Hebei Province | *Spermophilus dauricus* | Grassland – farmland mosaic | Population density | 1996 | 0.02 |
| 1338 | Hebei Province | *Spermophilus dauricus* | Grassland – farmland mosaic | Population density | 1997 | 0.91 |
| 1339 | Hebei Province | *Spermophilus dauricus* | Grassland – farmland mosaic | Population density | 1998 | 0.88 |
| 1340 | Hebei Province | *Spermophilus dauricus* | Grassland – farmland mosaic | Population density | 1999 | 0.93 |
| 1341 | Hebei Province | *Spermophilus dauricus* | Grassland – farmland mosaic | Population density | 2000 | 0.11 |
| 1342 | Hebei Province | *Spermophilus dauricus* | Grassland – farmland mosaic | Population density | 2001 | 0.89 |
| 1343 | Hebei Province | *Spermophilus dauricus* | Grassland – farmland mosaic | Population density | 2002 | 0.86 |
| 1344 | Hebei Province | *Spermophilus dauricus* | Grassland – farmland mosaic | Population density | 2003 | 0.13 |
| 1345 | Hebei Province | *Spermophilus dauricus* | Grassland – farmland mosaic | Population density | 2004 | 0.39 |
| 1346 | Hebei Province | *Spermophilus dauricus* | Grassland – farmland mosaic | Population density | 2005 | 0.21 |
| 1347 | Hebei Province | *Spermophilus dauricus* | Grassland – farmland mosaic | Population density | 2006 | 0.34 |
| 1348 | Hebei Province | *Spermophilus dauricus* | Grassland – farmland mosaic | Population density | 2007 | 0.14 |
| 1349 | Hebei Province | *Spermophilus dauricus* | Grassland – farmland mosaic | Population density | 2008 | 0.46 |
| 1350 | Hebei Province | *Spermophilus dauricus* | Grassland – farmland mosaic | Population density | 2009 | 1.00 |
| 1351 | Hebei Province | *Spermophilus dauricus* | Grassland – farmland mosaic | Population density | 2010 | 0.50 |
| 1352 | Hebei Province | *Spermophilus dauricus* | Grassland – farmland mosaic | Population density | 2011 | 0.91 |
| 1353 | Hebei Province | *Spermophilus dauricus* | Grassland – farmland mosaic | Population density | 2012 | 0.29 |
| 1354 | Hebei Province | *Spermophilus dauricus* | Grassland – farmland mosaic | Population density | 2013 | 0.66 |
| 1355 | Heilongjiang Province | *Spermophilus dauricus* | Grassland – farmland mosaic | Population density | 1990 | 0.23 |
| 1356 | Heilongjiang Province | *Spermophilus dauricus* | Grassland – farmland mosaic | Population density | 1991 | 0.69 |
| 1357 | Heilongjiang Province | *Spermophilus dauricus* | Grassland – farmland mosaic | Population density | 1992 | 1.00 |
| 1358 | Heilongjiang Province | *Spermophilus dauricus* | Grassland – farmland mosaic | Population density | 1993 | 0.65 |
| 1359 | Heilongjiang Province | *Spermophilus dauricus* | Grassland – farmland mosaic | Population density | 1994 | 0.17 |
| 1360 | Heilongjiang Province | *Spermophilus dauricus* | Grassland – farmland mosaic | Population density | 1995 | 0.42 |
| 1361 | Heilongjiang Province | *Spermophilus dauricus* | Grassland – farmland mosaic | Population density | 1996 | 0.33 |
| 1362 | Heilongjiang Province | *Spermophilus dauricus* | Grassland – farmland mosaic | Population density | 1997 | 0.30 |
| 1363 | Heilongjiang Province | *Spermophilus dauricus* | Grassland – farmland mosaic | Population density | 1998 | 0.34 |
| 1364 | Heilongjiang Province | *Spermophilus dauricus* | Grassland – farmland mosaic | Population density | 1999 | 0.33 |
| 1365 | Heilongjiang Province | *Spermophilus dauricus* | Grassland – farmland mosaic | Population density | 2000 | 0.28 |
| 1366 | Heilongjiang Province | *Spermophilus dauricus* | Grassland – farmland mosaic | Population density | 2001 | 0.11 |
| 1367 | Heilongjiang Province | *Spermophilus dauricus* | Grassland – farmland mosaic | Population density | 2002 | 0.32 |
| 1368 | Heilongjiang Province | *Spermophilus dauricus* | Grassland – farmland mosaic | Population density | 2003 | 0.28 |
| 1369 | Heilongjiang Province | *Spermophilus dauricus* | Grassland – farmland mosaic | Population density | 2004 | 0.24 |
| 1370 | Heilongjiang Province | *Spermophilus dauricus* | Grassland – farmland mosaic | Population density | 2005 | 0.13 |
| 1371 | Heilongjiang Province | *Spermophilus dauricus* | Grassland – farmland mosaic | Population density | 2006 | 0.28 |
| 1372 | Heilongjiang Province | *Spermophilus dauricus* | Grassland – farmland mosaic | Population density | 2007 | 0.06 |
| 1373 | Heilongjiang Province | *Spermophilus dauricus* | Grassland – farmland mosaic | Population density | 2008 | 0.06 |
| 1374 | Heilongjiang Province | *Spermophilus dauricus* | Grassland – farmland mosaic | Population density | 2009 | 0.03 |
| 1375 | Heilongjiang Province | *Spermophilus dauricus* | Grassland – farmland mosaic | Population density | 2010 | 0.01 |
| 1376 | Heilongjiang Province | *Spermophilus dauricus* | Grassland – farmland mosaic | Population density | 2011 | 0.01 |
| 1377 | Heilongjiang Province | *Spermophilus dauricus* | Grassland – farmland mosaic | Population density | 2012 | 0.00 |
| 1378 | Hubei Province | rodent | Farmland | Trap success | 1980 | 0.15 |
| 1379 | Hubei Province | rodent | Farmland | Trap success | 1981 | 0.26 |
| 1380 | Hubei Province | rodent | Farmland | Trap success | 1982 | 0.14 |
| 1381 | Hubei Province | rodent | Farmland | Trap success | 1983 | 0.45 |
| 1382 | Hubei Province | rodent | Farmland | Trap success | 1984 | 1.00 |
| 1383 | Hubei Province | rodent | Farmland | Trap success | 1985 | 0.20 |
| 1384 | Hubei Province | rodent | Farmland | Trap success | 1986 | 0.27 |
| 1385 | Hubei Province | rodent | Farmland | Trap success | 1987 | 0.19 |
| 1386 | Hubei Province | rodent | Farmland | Trap success | 1988 | 0.17 |
| 1387 | Hubei Province | rodent | Farmland | Trap success | 1989 | 0.26 |
| 1388 | Hubei Province | rodent | Farmland | Trap success | 1990 | 0.04 |
| 1389 | Hubei Province | rodent | Farmland | Trap success | 1991 | 0.04 |
| 1390 | Hubei Province | rodent | Farmland | Trap success | 1992 | 0.00 |
| 1391 | Hubei Province | rodent | Farmland | Trap success | 1993 | 0.06 |
| 1392 | Hubei Province | rodent | Farmland | Trap success | 1994 | 0.18 |
| 1393 | Hubei Province | rodent | Farmland | Trap success | 1995 | 0.62 |
| 1394 | Hubei Province | rodent | Farmland | Trap success | 1996 | 0.83 |
| 1395 | Hubei Province | rodent | Farmland | Trap success | 1997 | 0.67 |
| 1396 | Hubei Province | rodent | Farmland | Trap success | 1998 | 0.21 |
| 1397 | Hubei Province | rodent | Farmland | Trap success | 1999 | 0.30 |
| 1398 | Hubei Province | rodent | Farmland | Trap success | 2000 | 0.30 |
| 1399 | Jilin Province | *Spermophilus dauricus* | Grassland – farmland mosaic | Population density | 1990 | 0.88 |
| 1400 | Jilin Province | *Spermophilus dauricus* | Grassland – farmland mosaic | Population density | 1991 | 0.53 |
| 1401 | Jilin Province | *Spermophilus dauricus* | Grassland – farmland mosaic | Population density | 1992 | 0.57 |
| 1402 | Jilin Province | *Spermophilus dauricus* | Grassland – farmland mosaic | Population density | 1993 | 0.65 |
| 1403 | Jilin Province | *Spermophilus dauricus* | Grassland – farmland mosaic | Population density | 1994 | 0.77 |
| 1404 | Jilin Province | *Spermophilus dauricus* | Grassland – farmland mosaic | Population density | 1995 | 0.00 |
| 1405 | Jilin Province | *Spermophilus dauricus* | Grassland – farmland mosaic | Population density | 1996 | 1.00 |
| 1406 | Jilin Province | *Spermophilus dauricus* | Grassland – farmland mosaic | Population density | 1997 | 0.29 |
| 1407 | Jilin Province | *Spermophilus dauricus* | Grassland – farmland mosaic | Population density | 1998 | 0.63 |
| 1408 | Jilin Province | *Spermophilus dauricus* | Grassland – farmland mosaic | Population density | 1999 | 0.72 |
| 1409 | Jilin Province | *Spermophilus dauricus* | Grassland – farmland mosaic | Population density | 2000 | 0.21 |
| 1410 | Jilin Province | *Spermophilus dauricus* | Grassland – farmland mosaic | Population density | 2001 | 0.31 |
| 1411 | Jilin Province | *Spermophilus dauricus* | Grassland – farmland mosaic | Population density | 2002 | 0.29 |
| 1412 | Jilin Province | *Spermophilus dauricus* | Grassland – farmland mosaic | Population density | 2003 | 0.50 |
| 1413 | Jilin Province | *Spermophilus dauricus* | Grassland – farmland mosaic | Population density | 2004 | 0.30 |
| 1414 | Jilin Province | *Spermophilus dauricus* | Grassland – farmland mosaic | Population density | 2005 | 0.29 |
| 1415 | Jilin Province | *Spermophilus dauricus* | Grassland – farmland mosaic | Population density | 2006 | 0.10 |
| 1416 | Jilin Province | *Spermophilus dauricus* | Grassland – farmland mosaic | Population density | 2007 | 0.33 |
| 1417 | Jilin Province | *Spermophilus dauricus* | Grassland – farmland mosaic | Population density | 2008 | 0.12 |
| 1418 | Jilin Province | *Spermophilus dauricus* | Grassland – farmland mosaic | Population density | 2009 | 0.16 |
| 1419 | Jilin Province | *Spermophilus dauricus* | Grassland – farmland mosaic | Population density | 2010 | 0.33 |
| 1420 | Jilin Province | *Spermophilus dauricus* | Grassland – farmland mosaic | Population density | 2011 | 0.94 |
| 1421 | Jilin Province | *Spermophilus dauricus* | Grassland – farmland mosaic | Population density | 1981 | 0.94 |
| 1422 | Jilin Province | *Spermophilus dauricus* | Grassland – farmland mosaic | Population density | 1982 | 1.00 |
| 1423 | Jilin Province | *Spermophilus dauricus* | Grassland – farmland mosaic | Population density | 1983 | 0.48 |
| 1424 | Jilin Province | *Spermophilus dauricus* | Grassland – farmland mosaic | Population density | 1984 | 0.26 |
| 1425 | Jilin Province | *Spermophilus dauricus* | Grassland – farmland mosaic | Population density | 1985 | 0.19 |
| 1426 | Jilin Province | *Spermophilus dauricus* | Grassland – farmland mosaic | Population density | 1986 | 0.27 |
| 1427 | Jilin Province | *Spermophilus dauricus* | Grassland – farmland mosaic | Population density | 1987 | 0.14 |
| 1428 | Jilin Province | *Spermophilus dauricus* | Grassland – farmland mosaic | Population density | 1988 | 0.35 |
| 1429 | Jilin Province | *Spermophilus dauricus* | Grassland – farmland mosaic | Population density | 1989 | 0.38 |
| 1430 | Jilin Province | *Spermophilus dauricus* | Grassland – farmland mosaic | Population density | 1990 | 0.40 |
| 1431 | Jilin Province | *Spermophilus dauricus* | Grassland – farmland mosaic | Population density | 1991 | 0.24 |
| 1432 | Jilin Province | *Spermophilus dauricus* | Grassland – farmland mosaic | Population density | 1992 | 0.44 |
| 1433 | Jilin Province | *Spermophilus dauricus* | Grassland – farmland mosaic | Population density | 1993 | 0.23 |
| 1434 | Jilin Province | *Spermophilus dauricus* | Grassland – farmland mosaic | Population density | 1994 | 0.29 |
| 1435 | Jilin Province | *Spermophilus dauricus* | Grassland – farmland mosaic | Population density | 1995 | 0.27 |
| 1436 | Jilin Province | *Spermophilus dauricus* | Grassland – farmland mosaic | Population density | 1996 | 0.43 |
| 1437 | Jilin Province | *Spermophilus dauricus* | Grassland – farmland mosaic | Population density | 1997 | 0.19 |
| 1438 | Jilin Province | *Spermophilus dauricus* | Grassland – farmland mosaic | Population density | 1998 | 0.06 |
| 1439 | Jilin Province | *Spermophilus dauricus* | Grassland – farmland mosaic | Population density | 1999 | 0.35 |
| 1440 | Jilin Province | *Spermophilus dauricus* | Grassland – farmland mosaic | Population density | 2000 | 0.15 |
| 1441 | Jilin Province | *Spermophilus dauricus* | Grassland – farmland mosaic | Population density | 2001 | 0.12 |
| 1442 | Jilin Province | *Spermophilus dauricus* | Grassland – farmland mosaic | Population density | 2002 | 0.06 |
| 1443 | Jilin Province | *Spermophilus dauricus* | Grassland – farmland mosaic | Population density | 2003 | 0.11 |
| 1444 | Jilin Province | *Spermophilus dauricus* | Grassland – farmland mosaic | Population density | 2004 | 0.23 |
| 1445 | Jilin Province | *Spermophilus dauricus* | Grassland – farmland mosaic | Population density | 2005 | 0.14 |
| 1446 | Jilin Province | *Spermophilus dauricus* | Grassland – farmland mosaic | Population density | 2006 | 0.00 |
| 1447 | Jilin Province | *Spermophilus dauricus* | Grassland – farmland mosaic | Population density | 2007 | 0.00 |
| 1448 | Jilin Province | *Spermophilus dauricus* | Grassland – farmland mosaic | Population density | 2008 | 0.11 |
| 1449 | Jilin Province | *Spermophilus dauricus* | Grassland – farmland mosaic | Population density | 2009 | 0.05 |
| 1450 | Jilin Province | *Spermophilus dauricus* | Grassland – farmland mosaic | Population density | 2010 | 0.00 |
| 1451 | Jilin Province | *Spermophilus dauricus* | Grassland – farmland mosaic | Trap success | 1981 | 0.21 |
| 1452 | Jilin Province | *Spermophilus dauricus* | Grassland – farmland mosaic | Trap success | 1982 | 0.71 |
| 1453 | Jilin Province | *Spermophilus dauricus* | Grassland – farmland mosaic | Trap success | 1983 | 0.39 |
| 1454 | Jilin Province | *Spermophilus dauricus* | Grassland – farmland mosaic | Trap success | 1984 | 0.63 |
| 1455 | Jilin Province | *Spermophilus dauricus* | Grassland – farmland mosaic | Trap success | 1985 | 0.55 |
| 1456 | Jilin Province | *Spermophilus dauricus* | Grassland – farmland mosaic | Trap success | 1986 | 1.00 |
| 1457 | Jilin Province | *Spermophilus dauricus* | Grassland – farmland mosaic | Trap success | 1987 | 0.74 |
| 1458 | Jilin Province | *Spermophilus dauricus* | Grassland – farmland mosaic | Trap success | 1988 | 0.58 |
| 1459 | Jilin Province | *Spermophilus dauricus* | Grassland – farmland mosaic | Trap success | 1989 | 0.34 |
| 1460 | Jilin Province | *Spermophilus dauricus* | Grassland – farmland mosaic | Trap success | 1990 | 0.32 |
| 1461 | Jilin Province | *Spermophilus dauricus* | Grassland – farmland mosaic | Trap success | 1991 | 0.21 |
| 1462 | Jilin Province | *Spermophilus dauricus* | Grassland – farmland mosaic | Trap success | 1992 | 0.29 |
| 1463 | Jilin Province | *Spermophilus dauricus* | Grassland – farmland mosaic | Trap success | 1993 | 0.16 |
| 1464 | Jilin Province | *Spermophilus dauricus* | Grassland – farmland mosaic | Trap success | 1994 | 0.45 |
| 1465 | Jilin Province | *Spermophilus dauricus* | Grassland – farmland mosaic | Trap success | 1995 | 0.66 |
| 1466 | Jilin Province | *Spermophilus dauricus* | Grassland – farmland mosaic | Trap success | 1996 | 0.45 |
| 1467 | Jilin Province | *Spermophilus dauricus* | Grassland – farmland mosaic | Trap success | 1997 | 0.45 |
| 1468 | Jilin Province | *Spermophilus dauricus* | Grassland – farmland mosaic | Trap success | 1998 | 0.34 |
| 1469 | Jilin Province | *Spermophilus dauricus* | Grassland – farmland mosaic | Trap success | 1999 | 0.26 |
| 1470 | Jilin Province | *Spermophilus dauricus* | Grassland – farmland mosaic | Trap success | 2000 | 0.16 |
| 1471 | Jilin Province | *Spermophilus dauricus* | Grassland – farmland mosaic | Trap success | 2001 | 0.05 |
| 1472 | Jilin Province | *Spermophilus dauricus* | Grassland – farmland mosaic | Trap success | 2002 | 0.05 |
| 1473 | Jilin Province | *Spermophilus dauricus* | Grassland – farmland mosaic | Trap success | 2003 | 0.08 |
| 1474 | Jilin Province | *Spermophilus dauricus* | Grassland – farmland mosaic | Trap success | 2004 | 0.08 |
| 1475 | Jilin Province | *Spermophilus dauricus* | Grassland – farmland mosaic | Trap success | 2005 | 0.03 |
| 1476 | Jilin Province | *Spermophilus dauricus* | Grassland – farmland mosaic | Trap success | 2006 | 0.00 |
| 1477 | Jilin Province | *Spermophilus dauricus* | Grassland – farmland mosaic | Trap success | 2007 | 0.16 |
| 1478 | Jilin Province | *Spermophilus dauricus* | Grassland – farmland mosaic | Trap success | 2008 | 0.05 |
| 1479 | Jilin Province | *Spermophilus dauricus* | Grassland – farmland mosaic | Trap success | 2009 | 0.05 |
| 1480 | Jilin Province | *Spermophilus dauricus* | Grassland – farmland mosaic | Trap success | 2010 | 0.03 |
| 1481 | Jilin Province | *Spermophilus dauricus* | Grassland – farmland mosaic | Trap success | 2011 | 0.00 |
| 1482 | Jilin Province | *Spermophilus dauricus* | Grassland – farmland mosaic | Trap success | 2012 | 0.00 |
| 1483 | Jiangsu Province | rodent | City | Trap success | 1995 | 0.53 |
| 1484 | Jiangsu Province | rodent | City | Trap success | 1996 | 0.26 |
| 1485 | Jiangsu Province | rodent | City | Trap success | 1997 | 0.69 |
| 1486 | Jiangsu Province | rodent | City | Trap success | 1998 | 0.57 |
| 1487 | Jiangsu Province | rodent | City | Trap success | 1999 | 0.39 |
| 1488 | Jiangsu Province | rodent | City | Trap success | 2000 | 0.55 |
| 1489 | Jiangsu Province | rodent | City | Trap success | 2001 | 0.06 |
| 1490 | Jiangsu Province | rodent | City | Trap success | 2002 | 0.12 |
| 1491 | Jiangsu Province | rodent | City | Trap success | 2003 | 0.00 |
| 1492 | Jiangsu Province | rodent | City | Trap success | 2004 | 0.49 |
| 1493 | Jiangsu Province | rodent | City | Trap success | 2005 | 0.39 |
| 1494 | Jiangsu Province | rodent | City | Trap success | 2006 | 0.15 |
| 1495 | Jiangsu Province | rodent | City | Trap success | 2007 | 0.95 |
| 1496 | Jiangsu Province | rodent | City | Trap success | 2008 | 0.27 |
| 1497 | Jiangsu Province | rodent | City | Trap success | 2009 | 0.43 |
| 1498 | Jiangsu Province | rodent | City | Trap success | 2010 | 0.24 |
| 1499 | Jiangsu Province | rodent | City | Trap success | 2011 | 0.15 |
| 1500 | Jiangsu Province | rodent | City | Trap success | 2012 | 0.50 |
| 1501 | Jiangsu Province | rodent | City | Trap success | 2013 | 1.00 |
| 1502 | Jiangsu Province | rodent | City | Trap success | 2014 | 0.47 |
| 1503 | Liaoning Province | *Spermophilus dauricus* | Grassland – farmland mosaic | Population density | 1991 | 0.19 |
| 1504 | Liaoning Province | *Spermophilus dauricus* | Grassland – farmland mosaic | Population density | 1992 | 1.00 |
| 1505 | Liaoning Province | *Spermophilus dauricus* | Grassland – farmland mosaic | Population density | 1993 | 0.83 |
| 1506 | Liaoning Province | *Spermophilus dauricus* | Grassland – farmland mosaic | Population density | 1994 | 0.80 |
| 1507 | Liaoning Province | *Spermophilus dauricus* | Grassland – farmland mosaic | Population density | 1995 | 0.76 |
| 1508 | Liaoning Province | *Spermophilus dauricus* | Grassland – farmland mosaic | Population density | 1996 | 0.53 |
| 1509 | Liaoning Province | *Spermophilus dauricus* | Grassland – farmland mosaic | Population density | 1997 | 0.49 |
| 1510 | Liaoning Province | *Spermophilus dauricus* | Grassland – farmland mosaic | Population density | 1998 | 0.83 |
| 1511 | Liaoning Province | *Spermophilus dauricus* | Grassland – farmland mosaic | Population density | 1999 | 0.40 |
| 1512 | Liaoning Province | *Spermophilus dauricus* | Grassland – farmland mosaic | Population density | 2000 | 0.65 |
| 1513 | Liaoning Province | *Spermophilus dauricus* | Grassland – farmland mosaic | Population density | 2001 | 0.00 |
| 1514 | Liaoning Province | *Spermophilus dauricus* | Grassland – farmland mosaic | Population density | 2002 | 0.00 |
| 1515 | Liaoning Province | *Spermophilus dauricus* | Grassland – farmland mosaic | Population density | 2003 | 0.02 |
| 1516 | Liaoning Province | *Spermophilus dauricus* | Grassland – farmland mosaic | Population density | 2004 | 0.13 |
| 1517 | Liaoning Province | *Spermophilus dauricus* | Grassland – farmland mosaic | Population density | 2005 | 0.13 |
| 1518 | Liaoning Province | *Spermophilus dauricus* | Grassland – farmland mosaic | Population density | 2006 | 0.08 |
| 1519 | Liaoning Province | *Spermophilus dauricus* | Grassland – farmland mosaic | Population density | 2007 | 0.06 |
| 1520 | Liaoning Province | *Spermophilus dauricus* | Grassland – farmland mosaic | Population density | 2008 | 0.05 |
| 1521 | Liaoning Province | *Spermophilus dauricus* | Grassland – farmland mosaic | Population density | 2009 | 0.03 |
| 1522 | Liaoning Province | *Spermophilus dauricus* | Grassland – farmland mosaic | Population density | 2010 | 0.02 |
| 1523 | Liaoning Province | *Spermophilus dauricus* | Grassland – farmland mosaic | Population density | 2011 | 0.01 |
| 1524 | Liaoning Province | rodent | City | Trap success | 1985 | 0.85 |
| 1525 | Liaoning Province | rodent | City | Trap success | 1986 | 1.00 |
| 1526 | Liaoning Province | rodent | City | Trap success | 1987 | 0.31 |
| 1527 | Liaoning Province | rodent | City | Trap success | 1988 | 0.09 |
| 1528 | Liaoning Province | rodent | City | Trap success | 1989 | 0.08 |
| 1529 | Liaoning Province | rodent | City | Trap success | 1990 | 0.00 |
| 1530 | Liaoning Province | rodent | City | Trap success | 1991 | 0.27 |
| 1531 | Liaoning Province | rodent | City | Trap success | 1992 | 0.00 |
| 1532 | Liaoning Province | rodent | City | Trap success | 1993 | 0.09 |
| 1533 | Liaoning Province | rodent | City | Trap success | 1994 | 0.84 |
| 1534 | Liaoning Province | rodent | City | Trap success | 1995 | 0.65 |
| 1535 | Liaoning Province | rodent | City | Trap success | 1996 | 0.67 |
| 1536 | Liaoning Province | rodent | City | Trap success | 1997 | 0.33 |
| 1537 | Liaoning Province | rodent | City | Trap success | 1998 | 0.28 |
| 1538 | Liaoning Province | rodent | City | Trap success | 1999 | 0.37 |
| 1539 | Liaoning Province | rodent | City | Trap success | 2000 | 0.31 |
| 1540 | Liaoning Province | rodent | City | Trap success | 2001 | 0.30 |
| 1541 | Liaoning Province | rodent | City | Trap success | 2002 | 0.70 |
| 1542 | Liaoning Province | rodent | City | Trap success | 2003 | 0.31 |
| 1543 | Liaoning Province | rodent | City | Trap success | 2004 | 0.93 |
| 1544 | Liaoning Province | *Apodemus agrarius* | Farmland | Trap success | 1984 | 0.72 |
| 1545 | Liaoning Province | *Apodemus agrarius* | Farmland | Trap success | 1985 | 1.00 |
| 1546 | Liaoning Province | *Apodemus agrarius* | Farmland | Trap success | 1986 | 0.69 |
| 1547 | Liaoning Province | *Apodemus agrarius* | Farmland | Trap success | 1987 | 0.75 |
| 1548 | Liaoning Province | *Apodemus agrarius* | Farmland | Trap success | 1988 | 0.76 |
| 1549 | Liaoning Province | *Apodemus agrarius* | Farmland | Trap success | 1989 | 0.68 |
| 1550 | Liaoning Province | *Apodemus agrarius* | Farmland | Trap success | 1990 | 0.27 |
| 1551 | Liaoning Province | *Apodemus agrarius* | Farmland | Trap success | 1991 | 0.00 |
| 1552 | Liaoning Province | *Apodemus agrarius* | Farmland | Trap success | 1992 | 0.05 |
| 1553 | Liaoning Province | *Apodemus agrarius* | Farmland | Trap success | 1993 | 0.26 |
| 1554 | Liaoning Province | *Apodemus agrarius* | Farmland | Trap success | 1994 | 0.20 |
| 1555 | Liaoning Province | *Apodemus agrarius* | Farmland | Trap success | 1995 | 0.10 |
| 1556 | Liaoning Province | *Apodemus agrarius* | Farmland | Trap success | 1996 | 0.18 |
| 1557 | Liaoning Province | *Apodemus agrarius* | Farmland | Trap success | 1997 | 0.21 |
| 1558 | Liaoning Province | *Apodemus agrarius* | Farmland | Trap success | 1998 | 0.31 |
| 1559 | Liaoning Province | *Apodemus agrarius* | Farmland | Trap success | 1999 | 0.37 |
| 1560 | Liaoning Province | *Apodemus agrarius* | Farmland | Trap success | 2000 | 0.21 |
| 1561 | Liaoning Province | *Apodemus agrarius* | Farmland | Trap success | 2001 | 0.29 |
| 1562 | Liaoning Province | *Apodemus agrarius* | Farmland | Trap success | 2002 | 0.74 |
| 1563 | Liaoning Province | *Apodemus agrarius* | Farmland | Trap success | 2003 | 0.62 |
| 1564 | Liaoning Province | *Apodemus agrarius* | Farmland | Trap success | 2004 | 0.67 |
| 1565 | Liaoning Province | *Apodemus agrarius* | Farmland | Trap success | 2005 | 0.11 |
| 1566 | Liaoning Province | *Apodemus agrarius* | Farmland | Trap success | 2006 | 0.13 |
| 1567 | Liaoning Province | *Rattus norvegicus* | City | Trap success | 1984 | 0.46 |
| 1568 | Liaoning Province | *Rattus norvegicus* | City | Trap success | 1985 | 0.36 |
| 1569 | Liaoning Province | *Rattus norvegicus* | City | Trap success | 1986 | 0.27 |
| 1570 | Liaoning Province | *Rattus norvegicus* | City | Trap success | 1987 | 0.26 |
| 1571 | Liaoning Province | *Rattus norvegicus* | City | Trap success | 1988 | 0.17 |
| 1572 | Liaoning Province | *Rattus norvegicus* | City | Trap success | 1989 | 0.09 |
| 1573 | Liaoning Province | *Rattus norvegicus* | City | Trap success | 1990 | 0.18 |
| 1574 | Liaoning Province | *Rattus norvegicus* | City | Trap success | 1991 | 0.14 |
| 1575 | Liaoning Province | *Rattus norvegicus* | City | Trap success | 1992 | 0.23 |
| 1576 | Liaoning Province | *Rattus norvegicus* | City | Trap success | 1993 | 0.00 |
| 1577 | Liaoning Province | *Rattus norvegicus* | City | Trap success | 1994 | 0.04 |
| 1578 | Liaoning Province | *Rattus norvegicus* | City | Trap success | 1995 | 0.14 |
| 1579 | Liaoning Province | *Rattus norvegicus* | City | Trap success | 1996 | 0.15 |
| 1580 | Liaoning Province | *Rattus norvegicus* | City | Trap success | 1997 | 0.30 |
| 1581 | Liaoning Province | *Rattus norvegicus* | City | Trap success | 1998 | 0.36 |
| 1582 | Liaoning Province | *Rattus norvegicus* | City | Trap success | 1999 | 0.34 |
| 1583 | Liaoning Province | *Rattus norvegicus* | City | Trap success | 2000 | 0.42 |
| 1584 | Liaoning Province | *Rattus norvegicus* | City | Trap success | 2001 | 0.48 |
| 1585 | Liaoning Province | *Rattus norvegicus* | City | Trap success | 2002 | 0.64 |
| 1586 | Liaoning Province | *Rattus norvegicus* | City | Trap success | 2003 | 0.86 |
| 1587 | Liaoning Province | *Rattus norvegicus* | City | Trap success | 2004 | 1.00 |
| 1588 | Liaoning Province | *Rattus norvegicus* | City | Trap success | 2005 | 0.73 |
| 1589 | Liaoning Province | *Rattus norvegicus* | City | Trap success | 2006 | 0.16 |
| 1590 | Inner Mongolia Autonomous Region | *Allactaga sibirica* | Grassland | Trap success | 1984 | 0.11 |
| 1591 | Inner Mongolia Autonomous Region | *Allactaga sibirica* | Grassland | Trap success | 1985 | 0.26 |
| 1592 | Inner Mongolia Autonomous Region | *Allactaga sibirica* | Grassland | Trap success | 1986 | 0.54 |
| 1593 | Inner Mongolia Autonomous Region | *Allactaga sibirica* | Grassland | Trap success | 1987 | 1.00 |
| 1594 | Inner Mongolia Autonomous Region | *Allactaga sibirica* | Grassland | Trap success | 1988 | 0.77 |
| 1595 | Inner Mongolia Autonomous Region | *Allactaga sibirica* | Grassland | Trap success | 1989 | 0.60 |
| 1596 | Inner Mongolia Autonomous Region | *Allactaga sibirica* | Grassland | Trap success | 1990 | 0.93 |
| 1597 | Inner Mongolia Autonomous Region | *Allactaga sibirica* | Grassland | Trap success | 1991 | 0.72 |
| 1598 | Inner Mongolia Autonomous Region | *Allactaga sibirica* | Grassland | Trap success | 1992 | 0.20 |
| 1599 | Inner Mongolia Autonomous Region | *Allactaga sibirica* | Grassland | Trap success | 1993 | 0.15 |
| 1600 | Inner Mongolia Autonomous Region | *Allactaga sibirica* | Grassland | Trap success | 1994 | 0.00 |
| 1601 | Inner Mongolia Autonomous Region | *Allactaga sibirica* | Grassland | Trap success | 1995 | 0.00 |
| 1602 | Inner Mongolia Autonomous Region | *Allactaga sibirica* | Grassland | Trap success | 1996 | 0.00 |
| 1603 | Inner Mongolia Autonomous Region | *Allactaga sibirica* | Grassland | Trap success | 1997 | 0.00 |
| 1604 | Inner Mongolia Autonomous Region | *Allactaga sibirica* | Grassland | Trap success | 1998 | 0.22 |
| 1605 | Inner Mongolia Autonomous Region | *Allactaga sibirica* | Grassland | Trap success | 1999 | 0.19 |
| 1606 | Inner Mongolia Autonomous Region | *Allactaga sibirica* | Grassland | Trap success | 2000 | 0.18 |
| 1607 | Inner Mongolia Autonomous Region | *Allactaga sibirica* | Grassland | Trap success | 2001 | 0.27 |
| 1608 | Inner Mongolia Autonomous Region | *Allactaga sibirica* | Grassland | Trap success | 2002 | 0.00 |
| 1609 | Inner Mongolia Autonomous Region | *Allactaga sibirica* | Grassland | Trap success | 2003 | 0.09 |
| 1610 | Inner Mongolia Autonomous Region | *Allactaga sibirica* | Grassland | Trap success | 2004 | 0.15 |
| 1611 | Inner Mongolia Autonomous Region | *Allactaga sibirica* | Grassland | Trap success | 2005 | 0.00 |
| 1612 | Inner Mongolia Autonomous Region | *Allactaga sibirica* | Grassland | Trap success | 2006 | 0.00 |
| 1613 | Inner Mongolia Autonomous Region | *Allactaga sibirica* | Grassland | Trap success | 2007 | 0.00 |
| 1614 | Inner Mongolia Autonomous Region | *Meriones unguiculatus* | Grassland | Trap success | 1984 | 0.00 |
| 1615 | Inner Mongolia Autonomous Region | *Meriones unguiculatus* | Grassland | Trap success | 1985 | 0.02 |
| 1616 | Inner Mongolia Autonomous Region | *Meriones unguiculatus* | Grassland | Trap success | 1986 | 0.00 |
| 1617 | Inner Mongolia Autonomous Region | *Meriones unguiculatus* | Grassland | Trap success | 1987 | 0.02 |
| 1618 | Inner Mongolia Autonomous Region | *Meriones unguiculatus* | Grassland | Trap success | 1988 | 0.02 |
| 1619 | Inner Mongolia Autonomous Region | *Meriones unguiculatus* | Grassland | Trap success | 1989 | 0.03 |
| 1620 | Inner Mongolia Autonomous Region | *Meriones unguiculatus* | Grassland | Trap success | 1990 | 0.03 |
| 1621 | Inner Mongolia Autonomous Region | *Meriones unguiculatus* | Grassland | Trap success | 1991 | 0.02 |
| 1622 | Inner Mongolia Autonomous Region | *Meriones unguiculatus* | Grassland | Trap success | 1992 | 0.33 |
| 1623 | Inner Mongolia Autonomous Region | *Meriones unguiculatus* | Grassland | Trap success | 1993 | 0.48 |
| 1624 | Inner Mongolia Autonomous Region | *Meriones unguiculatus* | Grassland | Trap success | 1994 | 1.00 |
| 1625 | Inner Mongolia Autonomous Region | *Meriones unguiculatus* | Grassland | Trap success | 1995 | 0.78 |
| 1626 | Inner Mongolia Autonomous Region | *Meriones unguiculatus* | Grassland | Trap success | 1996 | 0.29 |
| 1627 | Inner Mongolia Autonomous Region | *Meriones unguiculatus* | Grassland | Trap success | 1997 | 0.15 |
| 1628 | Inner Mongolia Autonomous Region | *Meriones unguiculatus* | Grassland | Trap success | 1998 | 0.10 |
| 1629 | Inner Mongolia Autonomous Region | *Meriones unguiculatus* | Grassland | Trap success | 1999 | 0.06 |
| 1630 | Inner Mongolia Autonomous Region | *Meriones unguiculatus* | Grassland | Trap success | 2000 | 0.05 |
| 1631 | Inner Mongolia Autonomous Region | *Meriones unguiculatus* | Grassland | Trap success | 2001 | 0.08 |
| 1632 | Inner Mongolia Autonomous Region | *Meriones unguiculatus* | Grassland | Trap success | 2002 | 0.25 |
| 1633 | Inner Mongolia Autonomous Region | *Meriones unguiculatus* | Grassland | Trap success | 2003 | 0.02 |
| 1634 | Inner Mongolia Autonomous Region | *Meriones unguiculatus* | Grassland | Trap success | 2004 | 0.00 |
| 1635 | Inner Mongolia Autonomous Region | *Meriones unguiculatus* | Grassland | Trap success | 2005 | 0.00 |
| 1636 | Inner Mongolia Autonomous Region | *Meriones unguiculatus* | Grassland | Trap success | 2006 | 0.00 |
| 1637 | Inner Mongolia Autonomous Region | *Meriones unguiculatus* | Grassland | Trap success | 2007 | 0.00 |
| 1638 | Inner Mongolia Autonomous Region | *Cricetulus barabensis* | Grassland | Trap success | 1984 | 1.00 |
| 1639 | Inner Mongolia Autonomous Region | *Cricetulus barabensis* | Grassland | Trap success | 1985 | 0.53 |
| 1640 | Inner Mongolia Autonomous Region | *Cricetulus barabensis* | Grassland | Trap success | 1986 | 0.25 |
| 1641 | Inner Mongolia Autonomous Region | *Cricetulus barabensis* | Grassland | Trap success | 1987 | 0.13 |
| 1642 | Inner Mongolia Autonomous Region | *Cricetulus barabensis* | Grassland | Trap success | 1988 | 0.15 |
| 1643 | Inner Mongolia Autonomous Region | *Cricetulus barabensis* | Grassland | Trap success | 1989 | 0.11 |
| 1644 | Inner Mongolia Autonomous Region | *Cricetulus barabensis* | Grassland | Trap success | 1990 | 0.07 |
| 1645 | Inner Mongolia Autonomous Region | *Cricetulus barabensis* | Grassland | Trap success | 1991 | 0.12 |
| 1646 | Inner Mongolia Autonomous Region | *Cricetulus barabensis* | Grassland | Trap success | 1992 | 0.12 |
| 1647 | Inner Mongolia Autonomous Region | *Cricetulus barabensis* | Grassland | Trap success | 1993 | 0.13 |
| 1648 | Inner Mongolia Autonomous Region | *Cricetulus barabensis* | Grassland | Trap success | 1994 | 0.07 |
| 1649 | Inner Mongolia Autonomous Region | *Cricetulus barabensis* | Grassland | Trap success | 1995 | 0.00 |
| 1650 | Inner Mongolia Autonomous Region | *Cricetulus barabensis* | Grassland | Trap success | 1996 | 0.08 |
| 1651 | Inner Mongolia Autonomous Region | *Cricetulus barabensis* | Grassland | Trap success | 1997 | 0.24 |
| 1652 | Inner Mongolia Autonomous Region | *Cricetulus barabensis* | Grassland | Trap success | 1998 | 0.18 |
| 1653 | Inner Mongolia Autonomous Region | *Cricetulus barabensis* | Grassland | Trap success | 1999 | 0.19 |
| 1654 | Inner Mongolia Autonomous Region | *Cricetulus barabensis* | Grassland | Trap success | 2000 | 0.26 |
| 1655 | Inner Mongolia Autonomous Region | *Cricetulus barabensis* | Grassland | Trap success | 2001 | 0.28 |
| 1656 | Inner Mongolia Autonomous Region | *Cricetulus barabensis* | Grassland | Trap success | 2002 | 0.07 |
| 1657 | Inner Mongolia Autonomous Region | *Cricetulus barabensis* | Grassland | Trap success | 2003 | 0.19 |
| 1658 | Inner Mongolia Autonomous Region | *Cricetulus barabensis* | Grassland | Trap success | 2004 | 0.19 |
| 1659 | Inner Mongolia Autonomous Region | *Cricetulus barabensis* | Grassland | Trap success | 2005 | 0.17 |
| 1660 | Inner Mongolia Autonomous Region | *Cricetulus barabensis* | Grassland | Trap success | 2006 | 0.06 |
| 1661 | Inner Mongolia Autonomous Region | *Cricetulus barabensis* | Grassland | Trap success | 2007 | 0.07 |
| 1662 | Inner Mongolia Autonomous Region | *Cricetulus barabensis* | Grassland | Trap success | 1984 | 1.00 |
| 1663 | Inner Mongolia Autonomous Region | *Cricetulus barabensis* | Grassland | Trap success | 1985 | 0.53 |
| 1664 | Inner Mongolia Autonomous Region | *Cricetulus barabensis* | Grassland | Trap success | 1986 | 0.26 |
| 1665 | Inner Mongolia Autonomous Region | *Cricetulus barabensis* | Grassland | Trap success | 1987 | 0.14 |
| 1666 | Inner Mongolia Autonomous Region | *Cricetulus barabensis* | Grassland | Trap success | 1988 | 0.15 |
| 1667 | Inner Mongolia Autonomous Region | *Cricetulus barabensis* | Grassland | Trap success | 1989 | 0.12 |
| 1668 | Inner Mongolia Autonomous Region | *Cricetulus barabensis* | Grassland | Trap success | 1990 | 0.06 |
| 1669 | Inner Mongolia Autonomous Region | *Cricetulus barabensis* | Grassland | Trap success | 1991 | 0.12 |
| 1670 | Inner Mongolia Autonomous Region | *Cricetulus barabensis* | Grassland | Trap success | 1992 | 0.12 |
| 1671 | Inner Mongolia Autonomous Region | *Cricetulus barabensis* | Grassland | Trap success | 1993 | 0.13 |
| 1672 | Inner Mongolia Autonomous Region | *Cricetulus barabensis* | Grassland | Trap success | 1994 | 0.06 |
| 1673 | Inner Mongolia Autonomous Region | *Cricetulus barabensis* | Grassland | Trap success | 1995 | 0.00 |
| 1674 | Inner Mongolia Autonomous Region | *Cricetulus barabensis* | Grassland | Trap success | 1996 | 0.07 |
| 1675 | Inner Mongolia Autonomous Region | *Cricetulus barabensis* | Grassland | Trap success | 1997 | 0.23 |
| 1676 | Inner Mongolia Autonomous Region | *Cricetulus barabensis* | Grassland | Trap success | 1998 | 0.18 |
| 1677 | Inner Mongolia Autonomous Region | *Cricetulus barabensis* | Grassland | Trap success | 1999 | 0.20 |
| 1678 | Inner Mongolia Autonomous Region | *Cricetulus barabensis* | Grassland | Trap success | 2000 | 0.24 |
| 1679 | Inner Mongolia Autonomous Region | *Cricetulus barabensis* | Grassland | Trap success | 2001 | 0.26 |
| 1680 | Inner Mongolia Autonomous Region | *Cricetulus barabensis* | Grassland | Trap success | 2002 | 0.09 |
| 1681 | Inner Mongolia Autonomous Region | *Cricetulus barabensis* | Grassland | Trap success | 2003 | 0.17 |
| 1682 | Inner Mongolia Autonomous Region | *Marmota sibirica* | Grassland | Population density | 1981 | 0.00 |
| 1683 | Inner Mongolia Autonomous Region | *Marmota sibirica* | Grassland | Population density | 1982 | 0.12 |
| 1684 | Inner Mongolia Autonomous Region | *Marmota sibirica* | Grassland | Population density | 1983 | 0.23 |
| 1685 | Inner Mongolia Autonomous Region | *Marmota sibirica* | Grassland | Population density | 1984 | 0.48 |
| 1686 | Inner Mongolia Autonomous Region | *Marmota sibirica* | Grassland | Population density | 1985 | 1.00 |
| 1687 | Inner Mongolia Autonomous Region | *Marmota sibirica* | Grassland | Population density | 1986 | 0.15 |
| 1688 | Inner Mongolia Autonomous Region | *Marmota sibirica* | Grassland | Population density | 1987 | 0.02 |
| 1689 | Inner Mongolia Autonomous Region | *Marmota sibirica* | Grassland | Population density | 1988 | 0.06 |
| 1690 | Inner Mongolia Autonomous Region | *Marmota sibirica* | Grassland | Population density | 1989 | 0.00 |
| 1691 | Inner Mongolia Autonomous Region | *Marmota sibirica* | Grassland | Population density | 1990 | 0.00 |
| 1692 | Inner Mongolia Autonomous Region | *Marmota sibirica* | Grassland | Population density | 1991 | 0.00 |
| 1693 | Inner Mongolia Autonomous Region | *Marmota sibirica* | Grassland | Population density | 1992 | 0.01 |
| 1694 | Inner Mongolia Autonomous Region | *Marmota sibirica* | Grassland | Population density | 1993 | 0.00 |
| 1695 | Inner Mongolia Autonomous Region | *Marmota sibirica* | Grassland | Population density | 1994 | 0.00 |
| 1696 | Inner Mongolia Autonomous Region | *Marmota sibirica* | Grassland | Population density | 1995 | 0.00 |
| 1697 | Inner Mongolia Autonomous Region | *Marmota sibirica* | Grassland | Population density | 1996 | 0.00 |
| 1698 | Inner Mongolia Autonomous Region | *Marmota sibirica* | Grassland | Population density | 1997 | 0.00 |
| 1699 | Inner Mongolia Autonomous Region | *Marmota sibirica* | Grassland | Population density | 1998 | 0.00 |
| 1700 | Inner Mongolia Autonomous Region | *Marmota sibirica* | Grassland | Population density | 1999 | 0.01 |
| 1701 | Inner Mongolia Autonomous Region | *Marmota sibirica* | Grassland | Population density | 2000 | 0.00 |
| 1702 | Inner Mongolia Autonomous Region | *Marmota sibirica* | Grassland | Population density | 2001 | 0.01 |
| 1703 | Inner Mongolia Autonomous Region | *Marmota sibirica* | Grassland | Population density | 2002 | 0.17 |
| 1704 | Inner Mongolia Autonomous Region | *Marmota sibirica* | Grassland | Population density | 2003 | 0.01 |
| 1705 | Inner Mongolia Autonomous Region | *Marmota sibirica* | Grassland | Population density | 2004 | 0.17 |
| 1706 | Inner Mongolia Autonomous Region | *Marmota sibirica* | Grassland | Population density | 2005 | 0.00 |
| 1707 | Inner Mongolia Autonomous Region | *Marmota sibirica* | Grassland | Population density | 2006 | 0.00 |
| 1708 | Inner Mongolia Autonomous Region | *Marmota sibirica* | Grassland | Population density | 2007 | 0.03 |
| 1709 | Inner Mongolia Autonomous Region | *Marmota sibirica* | Grassland | Population density | 2008 | 0.08 |
| 1710 | Inner Mongolia Autonomous Region | *Marmota sibirica* | Grassland | Population density | 2009 | 0.01 |
| 1711 | Inner Mongolia Autonomous Region | *Marmota sibirica* | Grassland | Population density | 2010 | 0.01 |
| 1712 | Inner Mongolia Autonomous Region | *Spermophilus dauricus* | Grassland | Population density | 1981 | 0.00 |
| 1713 | Inner Mongolia Autonomous Region | *Spermophilus dauricus* | Grassland | Population density | 1982 | 0.40 |
| 1714 | Inner Mongolia Autonomous Region | *Spermophilus dauricus* | Grassland | Population density | 1985 | 0.41 |
| 1715 | Inner Mongolia Autonomous Region | *Spermophilus dauricus* | Grassland | Population density | 1986 | 0.09 |
| 1716 | Inner Mongolia Autonomous Region | *Spermophilus dauricus* | Grassland | Population density | 1987 | 0.07 |
| 1717 | Inner Mongolia Autonomous Region | *Spermophilus dauricus* | Grassland | Population density | 1988 | 0.20 |
| 1718 | Inner Mongolia Autonomous Region | *Spermophilus dauricus* | Grassland | Population density | 1989 | 0.41 |
| 1719 | Inner Mongolia Autonomous Region | *Spermophilus dauricus* | Grassland | Population density | 1990 | 0.35 |
| 1720 | Inner Mongolia Autonomous Region | *Spermophilus dauricus* | Grassland | Population density | 1993 | 0.03 |
| 1721 | Inner Mongolia Autonomous Region | *Spermophilus dauricus* | Grassland | Population density | 1994 | 0.22 |
| 1722 | Inner Mongolia Autonomous Region | *Spermophilus dauricus* | Grassland | Population density | 1995 | 0.22 |
| 1723 | Inner Mongolia Autonomous Region | *Spermophilus dauricus* | Grassland | Population density | 1996 | 1.00 |
| 1724 | Inner Mongolia Autonomous Region | *Spermophilus dauricus* | Grassland | Population density | 1997 | 0.43 |
| 1725 | Inner Mongolia Autonomous Region | *Spermophilus dauricus* | Grassland | Population density | 1998 | 0.45 |
| 1726 | Inner Mongolia Autonomous Region | *Spermophilus dauricus* | Grassland | Population density | 1999 | 0.35 |
| 1727 | Inner Mongolia Autonomous Region | *Spermophilus dauricus* | Grassland | Population density | 2000 | 0.49 |
| 1728 | Inner Mongolia Autonomous Region | *Spermophilus dauricus* | Grassland | Population density | 2001 | 0.03 |
| 1729 | Inner Mongolia Autonomous Region | *Spermophilus dauricus* | Grassland | Population density | 2002 | 0.18 |
| 1730 | Inner Mongolia Autonomous Region | *Spermophilus dauricus* | Grassland | Population density | 2003 | 0.20 |
| 1731 | Inner Mongolia Autonomous Region | *Spermophilus dauricus* | Grassland | Population density | 2004 | 0.99 |
| 1732 | Inner Mongolia Autonomous Region | *Spermophilus dauricus* | Grassland | Population density | 2005 | 0.48 |
| 1733 | Inner Mongolia Autonomous Region | *Spermophilus dauricus* | Grassland | Population density | 2006 | 0.39 |
| 1734 | Inner Mongolia Autonomous Region | *Spermophilus dauricus* | Grassland | Population density | 2007 | 0.27 |
| 1735 | Inner Mongolia Autonomous Region | *Spermophilus dauricus* | Grassland | Population density | 2008 | 0.43 |
| 1736 | Inner Mongolia Autonomous Region | *Spermophilus dauricus* | Grassland | Population density | 2009 | 0.48 |
| 1737 | Inner Mongolia Autonomous Region | *Spermophilus dauricus* | Grassland | Population density | 2010 | 0.36 |
| 1738 | Inner Mongolia Autonomous Region | *Spermophilus dauricus* | Grassland | Population density | 1990 | 0.15 |
| 1739 | Inner Mongolia Autonomous Region | *Spermophilus dauricus* | Grassland | Population density | 1991 | 0.02 |
| 1740 | Inner Mongolia Autonomous Region | *Spermophilus dauricus* | Grassland | Population density | 1992 | 0.03 |
| 1741 | Inner Mongolia Autonomous Region | *Spermophilus dauricus* | Grassland | Population density | 1993 | 0.12 |
| 1742 | Inner Mongolia Autonomous Region | *Spermophilus dauricus* | Grassland | Population density | 1994 | 0.13 |
| 1743 | Inner Mongolia Autonomous Region | *Spermophilus dauricus* | Grassland | Population density | 1995 | 0.07 |
| 1744 | Inner Mongolia Autonomous Region | *Spermophilus dauricus* | Grassland | Population density | 1996 | 0.04 |
| 1745 | Inner Mongolia Autonomous Region | *Spermophilus dauricus* | Grassland | Population density | 1997 | 0.02 |
| 1746 | Inner Mongolia Autonomous Region | *Spermophilus dauricus* | Grassland | Population density | 1998 | 0.00 |
| 1747 | Inner Mongolia Autonomous Region | *Spermophilus dauricus* | Grassland | Population density | 1999 | 0.13 |
| 1748 | Inner Mongolia Autonomous Region | *Spermophilus dauricus* | Grassland | Population density | 2000 | 0.15 |
| 1749 | Inner Mongolia Autonomous Region | *Spermophilus dauricus* | Grassland | Population density | 2001 | 0.09 |
| 1750 | Inner Mongolia Autonomous Region | *Spermophilus dauricus* | Grassland | Population density | 2002 | 0.16 |
| 1751 | Inner Mongolia Autonomous Region | *Spermophilus dauricus* | Grassland | Population density | 2003 | 0.11 |
| 1752 | Inner Mongolia Autonomous Region | *Spermophilus dauricus* | Grassland | Population density | 2004 | 0.10 |
| 1753 | Inner Mongolia Autonomous Region | *Spermophilus dauricus* | Grassland | Population density | 2005 | 0.07 |
| 1754 | Inner Mongolia Autonomous Region | *Spermophilus dauricus* | Grassland | Population density | 2006 | 0.03 |
| 1755 | Inner Mongolia Autonomous Region | *Spermophilus dauricus* | Grassland | Population density | 2007 | 0.06 |
| 1756 | Inner Mongolia Autonomous Region | *Spermophilus dauricus* | Grassland | Population density | 2008 | 0.05 |
| 1757 | Inner Mongolia Autonomous Region | *Spermophilus dauricus* | Grassland | Population density | 2009 | 0.04 |
| 1758 | Inner Mongolia Autonomous Region | *Spermophilus dauricus* | Grassland | Population density | 2010 | 1.00 |
| 1759 | Inner Mongolia Autonomous Region | *Spermophilus dauricus* | Grassland | Population density | 2011 | 0.13 |
| 1760 | Inner Mongolia Autonomous Region | *Spermophilus dauricus* | Grassland | Population density | 1980 | 0.04 |
| 1761 | Inner Mongolia Autonomous Region | *Spermophilus dauricus* | Grassland | Population density | 1981 | 0.00 |
| 1762 | Inner Mongolia Autonomous Region | *Spermophilus dauricus* | Grassland | Population density | 1982 | 0.10 |
| 1763 | Inner Mongolia Autonomous Region | *Spermophilus dauricus* | Grassland | Population density | 1983 | 0.23 |
| 1764 | Inner Mongolia Autonomous Region | *Spermophilus dauricus* | Grassland | Population density | 1984 | 0.17 |
| 1765 | Inner Mongolia Autonomous Region | *Spermophilus dauricus* | Grassland | Population density | 1985 | 0.40 |
| 1766 | Inner Mongolia Autonomous Region | *Spermophilus dauricus* | Grassland | Population density | 1986 | 0.39 |
| 1767 | Inner Mongolia Autonomous Region | *Spermophilus dauricus* | Grassland | Population density | 1987 | 0.37 |
| 1768 | Inner Mongolia Autonomous Region | *Spermophilus dauricus* | Grassland | Population density | 1988 | 0.44 |
| 1769 | Inner Mongolia Autonomous Region | *Spermophilus dauricus* | Grassland | Population density | 1989 | 0.32 |
| 1770 | Inner Mongolia Autonomous Region | *Spermophilus dauricus* | Grassland | Population density | 1990 | 0.15 |
| 1771 | Inner Mongolia Autonomous Region | *Spermophilus dauricus* | Grassland | Population density | 1991 | 0.30 |
| 1772 | Inner Mongolia Autonomous Region | *Spermophilus dauricus* | Grassland | Population density | 1992 | 0.69 |
| 1773 | Inner Mongolia Autonomous Region | *Spermophilus dauricus* | Grassland | Population density | 1993 | 0.81 |
| 1774 | Inner Mongolia Autonomous Region | *Spermophilus dauricus* | Grassland | Population density | 1994 | 1.00 |
| 1775 | Inner Mongolia Autonomous Region | *Spermophilus dauricus* | Grassland | Population density | 1995 | 0.96 |
| 1776 | Inner Mongolia Autonomous Region | *Spermophilus dauricus* | Grassland | Population density | 1996 | 0.84 |
| 1777 | Inner Mongolia Autonomous Region | *Spermophilus dauricus* | Grassland | Population density | 1997 | 0.60 |
| 1778 | Inner Mongolia Autonomous Region | *Spermophilus dauricus* | Grassland | Population density | 1998 | 0.77 |
| 1779 | Inner Mongolia Autonomous Region | *Spermophilus dauricus* | Grassland | Population density | 1999 | 0.76 |
| 1780 | Inner Mongolia Autonomous Region | *Spermophilus dauricus* | Grassland | Population density | 2000 | 0.58 |
| 1781 | Inner Mongolia Autonomous Region | *Spermophilus dauricus* | Grassland | Population density | 2001 | 0.59 |
| 1782 | Inner Mongolia Autonomous Region | *Spermophilus dauricus* | Grassland | Population density | 2002 | 0.65 |
| 1783 | Inner Mongolia Autonomous Region | *Spermophilus dauricus* | Grassland | Population density | 2003 | 0.65 |
| 1784 | Inner Mongolia Autonomous Region | *Spermophilus dauricus* | Grassland | Population density | 2004 | 0.56 |
| 1785 | Inner Mongolia Autonomous Region | *Spermophilus dauricus* | Grassland | Population density | 2005 | 0.58 |
| 1786 | Inner Mongolia Autonomous Region | *Spermophilus dauricus* | Grassland | Population density | 2006 | 0.28 |
| 1787 | Inner Mongolia Autonomous Region | *Spermophilus dauricus* | Grassland | Population density | 2007 | 0.38 |
| 1788 | Inner Mongolia Autonomous Region | *Spermophilus dauricus* | Grassland | Population density | 2008 | 0.46 |
| 1789 | Inner Mongolia Autonomous Region | *Spermophilus dauricus* | Grassland | Population density | 2009 | 0.49 |
| 1790 | Inner Mongolia Autonomous Region | *Spermophilus dauricus* | Grassland | Population density | 2010 | 0.19 |
| 1791 | Inner Mongolia Autonomous Region | *Spermophilus dauricus* | Grassland | Population density | 2011 | 0.08 |
| 1792 | Ningxia Hui Autonomous Region | *Spermophilus alaschanicus* | Grassland | Population density | 1980 | 0.20 |
| 1793 | Ningxia Hui Autonomous Region | *Spermophilus alaschanicus* | Grassland | Population density | 1981 | 0.09 |
| 1794 | Ningxia Hui Autonomous Region | *Spermophilus alaschanicus* | Grassland | Population density | 1982 | 0.03 |
| 1795 | Ningxia Hui Autonomous Region | *Spermophilus alaschanicus* | Grassland | Population density | 1983 | 0.00 |
| 1796 | Ningxia Hui Autonomous Region | *Spermophilus alaschanicus* | Grassland | Population density | 1984 | 0.03 |
| 1797 | Ningxia Hui Autonomous Region | *Spermophilus alaschanicus* | Grassland | Population density | 1985 | 0.03 |
| 1798 | Ningxia Hui Autonomous Region | *Spermophilus alaschanicus* | Grassland | Population density | 1986 | 0.06 |
| 1799 | Ningxia Hui Autonomous Region | *Spermophilus alaschanicus* | Grassland | Population density | 1987 | 0.03 |
| 1800 | Ningxia Hui Autonomous Region | *Spermophilus alaschanicus* | Grassland | Population density | 1988 | 0.06 |
| 1801 | Ningxia Hui Autonomous Region | *Spermophilus alaschanicus* | Grassland | Population density | 1989 | 0.03 |
| 1802 | Ningxia Hui Autonomous Region | *Spermophilus alaschanicus* | Grassland | Population density | 1990 | 0.03 |
| 1803 | Ningxia Hui Autonomous Region | *Spermophilus alaschanicus* | Grassland | Population density | 1991 | 0.06 |
| 1804 | Ningxia Hui Autonomous Region | *Spermophilus alaschanicus* | Grassland | Population density | 1992 | 0.14 |
| 1805 | Ningxia Hui Autonomous Region | *Spermophilus alaschanicus* | Grassland | Population density | 1993 | 0.09 |
| 1806 | Ningxia Hui Autonomous Region | *Spermophilus alaschanicus* | Grassland | Population density | 1994 | 0.23 |
| 1807 | Ningxia Hui Autonomous Region | *Spermophilus alaschanicus* | Grassland | Population density | 1995 | 0.20 |
| 1808 | Ningxia Hui Autonomous Region | *Spermophilus alaschanicus* | Grassland | Population density | 1996 | 0.77 |
| 1809 | Ningxia Hui Autonomous Region | *Spermophilus alaschanicus* | Grassland | Population density | 1997 | 0.49 |
| 1810 | Ningxia Hui Autonomous Region | *Spermophilus alaschanicus* | Grassland | Population density | 1998 | 0.46 |
| 1811 | Ningxia Hui Autonomous Region | *Spermophilus alaschanicus* | Grassland | Population density | 1999 | 1.00 |
| 1812 | Ningxia Hui Autonomous Region | *Spermophilus alaschanicus* | Grassland | Population density | 2000 | 0.77 |
| 1813 | Ningxia Hui Autonomous Region | *Spermophilus alaschanicus* | Grassland | Population density | 2001 | 0.69 |
| 1814 | Ningxia Hui Autonomous Region | *Spermophilus alaschanicus* | Grassland | Population density | 2002 | 0.66 |
| 1815 | Ningxia Hui Autonomous Region | *Spermophilus alaschanicus* | Grassland | Population density | 2003 | 0.80 |
| 1816 | Ningxia Hui Autonomous Region | *Spermophilus alaschanicus* | Grassland | Population density | 2004 | 0.86 |
| 1817 | Ningxia Hui Autonomous Region | *Spermophilus alaschanicus* | Grassland | Population density | 2005 | 0.71 |
| 1818 | Ningxia Hui Autonomous Region | *Spermophilus alaschanicus* | Grassland | Population density | 2006 | 0.60 |
| 1819 | Ningxia Hui Autonomous Region | *Spermophilus alaschanicus* | Grassland | Population density | 2007 | 0.40 |
| 1820 | Ningxia Hui Autonomous Region | *Spermophilus alaschanicus* | Grassland | Population density | 2008 | 0.09 |
| 1821 | Ningxia Hui Autonomous Region | *Spermophilus alaschanicus* | Grassland | Population density | 1986 | 0.21 |
| 1822 | Ningxia Hui Autonomous Region | *Spermophilus alaschanicus* | Grassland | Population density | 1987 | 0.00 |
| 1823 | Ningxia Hui Autonomous Region | *Spermophilus alaschanicus* | Grassland | Population density | 1988 | 0.13 |
| 1824 | Ningxia Hui Autonomous Region | *Spermophilus alaschanicus* | Grassland | Population density | 1989 | 0.07 |
| 1825 | Ningxia Hui Autonomous Region | *Spermophilus alaschanicus* | Grassland | Population density | 1990 | 0.08 |
| 1826 | Ningxia Hui Autonomous Region | *Spermophilus alaschanicus* | Grassland | Population density | 1991 | 0.08 |
| 1827 | Ningxia Hui Autonomous Region | *Spermophilus alaschanicus* | Grassland | Population density | 1992 | 0.05 |
| 1828 | Ningxia Hui Autonomous Region | *Spermophilus alaschanicus* | Grassland | Population density | 1993 | 0.10 |
| 1829 | Ningxia Hui Autonomous Region | *Spermophilus alaschanicus* | Grassland | Population density | 1994 | 0.18 |
| 1830 | Ningxia Hui Autonomous Region | *Spermophilus alaschanicus* | Grassland | Population density | 1995 | 0.43 |
| 1831 | Ningxia Hui Autonomous Region | *Spermophilus alaschanicus* | Grassland | Population density | 1996 | 0.24 |
| 1832 | Ningxia Hui Autonomous Region | *Spermophilus alaschanicus* | Grassland | Population density | 1997 | 0.29 |
| 1833 | Ningxia Hui Autonomous Region | *Spermophilus alaschanicus* | Grassland | Population density | 1998 | 0.43 |
| 1834 | Ningxia Hui Autonomous Region | *Spermophilus alaschanicus* | Grassland | Population density | 1999 | 1.00 |
| 1835 | Ningxia Hui Autonomous Region | *Spermophilus alaschanicus* | Grassland | Population density | 2000 | 0.93 |
| 1836 | Ningxia Hui Autonomous Region | *Spermophilus alaschanicus* | Grassland | Population density | 2001 | 0.74 |
| 1837 | Ningxia Hui Autonomous Region | *Spermophilus alaschanicus* | Grassland | Population density | 2002 | 0.73 |
| 1838 | Ningxia Hui Autonomous Region | *Spermophilus alaschanicus* | Grassland | Population density | 2003 | 0.59 |
| 1839 | Ningxia Hui Autonomous Region | *Spermophilus alaschanicus* | Grassland | Population density | 2004 | 0.37 |
| 1840 | Ningxia Hui Autonomous Region | *Spermophilus alaschanicus* | Grassland | Population density | 2005 | 0.30 |
| 1841 | Ningxia Hui Autonomous Region | *Spermophilus alaschanicus* | Grassland | Population density | 1980 | 0.17 |
| 1842 | Ningxia Hui Autonomous Region | *Spermophilus alaschanicus* | Grassland | Population density | 1981 | 0.09 |
| 1843 | Ningxia Hui Autonomous Region | *Spermophilus alaschanicus* | Grassland | Population density | 1982 | 0.03 |
| 1844 | Ningxia Hui Autonomous Region | *Spermophilus alaschanicus* | Grassland | Population density | 1983 | 0.00 |
| 1845 | Ningxia Hui Autonomous Region | *Spermophilus alaschanicus* | Grassland | Population density | 1984 | 0.03 |
| 1846 | Ningxia Hui Autonomous Region | *Spermophilus alaschanicus* | Grassland | Population density | 1985 | 0.03 |
| 1847 | Ningxia Hui Autonomous Region | *Spermophilus alaschanicus* | Grassland | Population density | 1986 | 0.06 |
| 1848 | Ningxia Hui Autonomous Region | *Spermophilus alaschanicus* | Grassland | Population density | 1987 | 0.03 |
| 1849 | Ningxia Hui Autonomous Region | *Spermophilus alaschanicus* | Grassland | Population density | 1988 | 0.06 |
| 1850 | Ningxia Hui Autonomous Region | *Spermophilus alaschanicus* | Grassland | Population density | 1989 | 0.03 |
| 1851 | Ningxia Hui Autonomous Region | *Spermophilus alaschanicus* | Grassland | Population density | 1990 | 0.03 |
| 1852 | Ningxia Hui Autonomous Region | *Spermophilus alaschanicus* | Grassland | Population density | 1991 | 0.06 |
| 1853 | Ningxia Hui Autonomous Region | *Spermophilus alaschanicus* | Grassland | Population density | 1992 | 0.14 |
| 1854 | Ningxia Hui Autonomous Region | *Spermophilus alaschanicus* | Grassland | Population density | 1993 | 0.09 |
| 1855 | Ningxia Hui Autonomous Region | *Spermophilus alaschanicus* | Grassland | Population density | 1994 | 0.23 |
| 1856 | Ningxia Hui Autonomous Region | *Spermophilus alaschanicus* | Grassland | Population density | 1995 | 0.20 |
| 1857 | Ningxia Hui Autonomous Region | *Spermophilus alaschanicus* | Grassland | Population density | 1996 | 0.77 |
| 1858 | Ningxia Hui Autonomous Region | *Spermophilus alaschanicus* | Grassland | Population density | 1997 | 0.49 |
| 1859 | Ningxia Hui Autonomous Region | *Spermophilus alaschanicus* | Grassland | Population density | 1998 | 0.46 |
| 1860 | Ningxia Hui Autonomous Region | *Spermophilus alaschanicus* | Grassland | Population density | 1999 | 1.00 |
| 1861 | Ningxia Hui Autonomous Region | rodent | City | Trap success | 1982 | 0.16 |
| 1862 | Ningxia Hui Autonomous Region | rodent | City | Trap success | 1983 | 0.04 |
| 1863 | Ningxia Hui Autonomous Region | rodent | City | Trap success | 1984 | 0.09 |
| 1864 | Ningxia Hui Autonomous Region | rodent | City | Trap success | 1985 | 0.03 |
| 1865 | Ningxia Hui Autonomous Region | rodent | City | Trap success | 1986 | 0.14 |
| 1866 | Ningxia Hui Autonomous Region | rodent | City | Trap success | 1987 | 0.07 |
| 1867 | Ningxia Hui Autonomous Region | rodent | City | Trap success | 1988 | 0.16 |
| 1868 | Ningxia Hui Autonomous Region | rodent | City | Trap success | 1989 | 0.19 |
| 1869 | Ningxia Hui Autonomous Region | rodent | City | Trap success | 1990 | 0.00 |
| 1870 | Ningxia Hui Autonomous Region | rodent | City | Trap success | 1991 | 0.83 |
| 1871 | Ningxia Hui Autonomous Region | rodent | City | Trap success | 1992 | 0.56 |
| 1872 | Ningxia Hui Autonomous Region | rodent | City | Trap success | 1993 | 0.31 |
| 1873 | Ningxia Hui Autonomous Region | rodent | City | Trap success | 1994 | 0.52 |
| 1874 | Ningxia Hui Autonomous Region | rodent | City | Trap success | 1995 | 0.52 |
| 1875 | Ningxia Hui Autonomous Region | rodent | City | Trap success | 1996 | 0.80 |
| 1876 | Ningxia Hui Autonomous Region | rodent | City | Trap success | 1997 | 0.78 |
| 1877 | Ningxia Hui Autonomous Region | rodent | City | Trap success | 1998 | 0.46 |
| 1878 | Ningxia Hui Autonomous Region | rodent | City | Trap success | 1999 | 0.44 |
| 1879 | Ningxia Hui Autonomous Region | rodent | City | Trap success | 2000 | 0.37 |
| 1880 | Ningxia Hui Autonomous Region | rodent | City | Trap success | 2001 | 1.00 |
| 1881 | Ningxia Hui Autonomous Region | rodent | Grassland – farmland mosaic | Trap success | 1982 | 0.21 |
| 1882 | Ningxia Hui Autonomous Region | rodent | Grassland – farmland mosaic | Trap success | 1983 | 0.00 |
| 1883 | Ningxia Hui Autonomous Region | rodent | Grassland – farmland mosaic | Trap success | 1984 | 0.02 |
| 1884 | Ningxia Hui Autonomous Region | rodent | Grassland – farmland mosaic | Trap success | 1985 | 0.08 |
| 1885 | Ningxia Hui Autonomous Region | rodent | Grassland – farmland mosaic | Trap success | 1986 | 0.04 |
| 1886 | Ningxia Hui Autonomous Region | rodent | Grassland – farmland mosaic | Trap success | 1987 | 0.12 |
| 1887 | Ningxia Hui Autonomous Region | rodent | Grassland – farmland mosaic | Trap success | 1988 | 0.09 |
| 1888 | Ningxia Hui Autonomous Region | rodent | Grassland – farmland mosaic | Trap success | 1989 | 0.31 |
| 1889 | Ningxia Hui Autonomous Region | rodent | Grassland – farmland mosaic | Trap success | 1990 | 0.27 |
| 1890 | Ningxia Hui Autonomous Region | rodent | Grassland – farmland mosaic | Trap success | 1991 | 0.79 |
| 1891 | Ningxia Hui Autonomous Region | rodent | Grassland – farmland mosaic | Trap success | 1992 | 0.78 |
| 1892 | Ningxia Hui Autonomous Region | rodent | Grassland – farmland mosaic | Trap success | 1993 | 0.62 |
| 1893 | Ningxia Hui Autonomous Region | rodent | Grassland – farmland mosaic | Trap success | 1994 | 0.88 |
| 1894 | Ningxia Hui Autonomous Region | rodent | Grassland – farmland mosaic | Trap success | 1995 | 0.86 |
| 1895 | Ningxia Hui Autonomous Region | rodent | Grassland – farmland mosaic | Trap success | 1996 | 1.00 |
| 1896 | Ningxia Hui Autonomous Region | rodent | Grassland – farmland mosaic | Trap success | 1997 | 0.99 |
| 1897 | Ningxia Hui Autonomous Region | rodent | Grassland – farmland mosaic | Trap success | 1998 | 0.70 |
| 1898 | Ningxia Hui Autonomous Region | rodent | Grassland – farmland mosaic | Trap success | 1999 | 0.76 |
| 1899 | Ningxia Hui Autonomous Region | rodent | Grassland – farmland mosaic | Trap success | 2000 | 1.00 |
| 1900 | Ningxia Hui Autonomous Region | rodent | Grassland – farmland mosaic | Trap success | 2001 | 0.97 |
| 1901 | Ningxia Hui Autonomous Region | *Spermophilus alaschanicus* | Grassland | Population density | 1981 | 0.11 |
| 1902 | Ningxia Hui Autonomous Region | *Spermophilus alaschanicus* | Grassland | Population density | 1982 | 0.03 |
| 1903 | Ningxia Hui Autonomous Region | *Spermophilus alaschanicus* | Grassland | Population density | 1983 | 0.00 |
| 1904 | Ningxia Hui Autonomous Region | *Spermophilus alaschanicus* | Grassland | Population density | 1984 | 0.03 |
| 1905 | Ningxia Hui Autonomous Region | *Spermophilus alaschanicus* | Grassland | Population density | 1985 | 0.03 |
| 1906 | Ningxia Hui Autonomous Region | *Spermophilus alaschanicus* | Grassland | Population density | 1986 | 0.06 |
| 1907 | Ningxia Hui Autonomous Region | *Spermophilus alaschanicus* | Grassland | Population density | 1987 | 0.03 |
| 1908 | Ningxia Hui Autonomous Region | *Spermophilus alaschanicus* | Grassland | Population density | 1988 | 0.06 |
| 1909 | Ningxia Hui Autonomous Region | *Spermophilus alaschanicus* | Grassland | Population density | 1989 | 0.03 |
| 1910 | Ningxia Hui Autonomous Region | *Spermophilus alaschanicus* | Grassland | Population density | 1990 | 0.07 |
| 1911 | Ningxia Hui Autonomous Region | *Spermophilus alaschanicus* | Grassland | Population density | 1991 | 0.07 |
| 1912 | Ningxia Hui Autonomous Region | *Spermophilus alaschanicus* | Grassland | Population density | 1992 | 0.15 |
| 1913 | Ningxia Hui Autonomous Region | *Spermophilus alaschanicus* | Grassland | Population density | 1993 | 0.07 |
| 1914 | Ningxia Hui Autonomous Region | *Spermophilus alaschanicus* | Grassland | Population density | 1994 | 0.21 |
| 1915 | Ningxia Hui Autonomous Region | *Spermophilus alaschanicus* | Grassland | Population density | 1995 | 0.29 |
| 1916 | Ningxia Hui Autonomous Region | *Spermophilus alaschanicus* | Grassland | Population density | 1996 | 0.76 |
| 1917 | Ningxia Hui Autonomous Region | *Spermophilus alaschanicus* | Grassland | Population density | 1997 | 0.50 |
| 1918 | Ningxia Hui Autonomous Region | *Spermophilus alaschanicus* | Grassland | Population density | 1998 | 0.46 |
| 1919 | Ningxia Hui Autonomous Region | *Spermophilus alaschanicus* | Grassland | Population density | 1999 | 1.00 |
| 1920 | Ningxia Hui Autonomous Region | *Spermophilus alaschanicus* | Grassland | Population density | 2000 | 0.75 |
| 1921 | Ningxia Hui Autonomous Region | *Spermophilus alaschanicus* | Grassland | Population density | 2001 | 0.65 |
| 1922 | Ningxia Hui Autonomous Region | *Spermophilus alaschanicus* | Grassland | Population density | 1981 | 0.30 |
| 1923 | Ningxia Hui Autonomous Region | *Spermophilus alaschanicus* | Grassland | Population density | 1982 | 0.12 |
| 1924 | Ningxia Hui Autonomous Region | *Spermophilus alaschanicus* | Grassland | Population density | 1983 | 0.01 |
| 1925 | Ningxia Hui Autonomous Region | *Spermophilus alaschanicus* | Grassland | Population density | 1984 | 0.08 |
| 1926 | Ningxia Hui Autonomous Region | *Spermophilus alaschanicus* | Grassland | Population density | 1985 | 0.11 |
| 1927 | Ningxia Hui Autonomous Region | *Spermophilus alaschanicus* | Grassland | Population density | 1986 | 0.14 |
| 1928 | Ningxia Hui Autonomous Region | *Spermophilus alaschanicus* | Grassland | Population density | 1987 | 0.11 |
| 1929 | Ningxia Hui Autonomous Region | *Spermophilus alaschanicus* | Grassland | Population density | 1988 | 0.34 |
| 1930 | Ningxia Hui Autonomous Region | *Spermophilus alaschanicus* | Grassland | Population density | 1989 | 0.35 |
| 1931 | Ningxia Hui Autonomous Region | *Spermophilus alaschanicus* | Grassland | Population density | 1990 | 0.33 |
| 1932 | Ningxia Hui Autonomous Region | *Spermophilus alaschanicus* | Grassland | Population density | 1991 | 0.53 |
| 1933 | Ningxia Hui Autonomous Region | *Spermophilus alaschanicus* | Grassland | Population density | 1992 | 0.62 |
| 1934 | Ningxia Hui Autonomous Region | *Spermophilus alaschanicus* | Grassland | Population density | 1993 | 0.74 |
| 1935 | Ningxia Hui Autonomous Region | *Spermophilus alaschanicus* | Grassland | Population density | 1994 | 0.89 |
| 1936 | Ningxia Hui Autonomous Region | *Spermophilus alaschanicus* | Grassland | Population density | 1995 | 0.62 |
| 1937 | Ningxia Hui Autonomous Region | *Spermophilus alaschanicus* | Grassland | Population density | 1996 | 0.99 |
| 1938 | Ningxia Hui Autonomous Region | *Spermophilus alaschanicus* | Grassland | Population density | 1997 | 0.99 |
| 1939 | Ningxia Hui Autonomous Region | *Spermophilus alaschanicus* | Grassland | Population density | 1998 | 0.97 |
| 1940 | Ningxia Hui Autonomous Region | *Spermophilus alaschanicus* | Grassland | Population density | 1999 | 1.00 |
| 1941 | Ningxia Hui Autonomous Region | *Spermophilus alaschanicus* | Grassland | Population density | 2000 | 0.79 |
| 1942 | Ningxia Hui Autonomous Region | *Spermophilus alaschanicus* | Grassland | Population density | 2001 | 0.79 |
| 1943 | Ningxia Hui Autonomous Region | *Spermophilus alaschanicus* | Grassland | Population density | 2002 | 0.80 |
| 1944 | Ningxia Hui Autonomous Region | *Spermophilus alaschanicus* | Grassland | Population density | 2003 | 0.64 |
| 1945 | Ningxia Hui Autonomous Region | *Spermophilus alaschanicus* | Grassland | Population density | 2004 | 0.61 |
| 1946 | Ningxia Hui Autonomous Region | *Spermophilus alaschanicus* | Grassland | Population density | 2005 | 0.61 |
| 1947 | Ningxia Hui Autonomous Region | *Spermophilus alaschanicus* | Grassland | Population density | 2006 | 0.74 |
| 1948 | Ningxia Hui Autonomous Region | *Spermophilus alaschanicus* | Grassland | Population density | 2007 | 0.67 |
| 1949 | Ningxia Hui Autonomous Region | *Spermophilus alaschanicus* | Grassland | Population density | 2008 | 0.34 |
| 1950 | Ningxia Hui Autonomous Region | *Spermophilus alaschanicus* | Grassland | Population density | 2009 | 0.18 |
| 1951 | Ningxia Hui Autonomous Region | *Spermophilus alaschanicus* | Grassland | Population density | 2010 | 0.00 |
| 1952 | Ningxia Hui Autonomous Region | *Spermophilus alaschanicus* | Grassland | Population density | 1985 | 0.01 |
| 1953 | Ningxia Hui Autonomous Region | *Spermophilus alaschanicus* | Grassland | Population density | 1986 | 0.00 |
| 1954 | Ningxia Hui Autonomous Region | *Spermophilus alaschanicus* | Grassland | Population density | 1987 | 0.05 |
| 1955 | Ningxia Hui Autonomous Region | *Spermophilus alaschanicus* | Grassland | Population density | 1988 | 0.09 |
| 1956 | Ningxia Hui Autonomous Region | *Spermophilus alaschanicus* | Grassland | Population density | 1989 | 0.10 |
| 1957 | Ningxia Hui Autonomous Region | *Spermophilus alaschanicus* | Grassland | Population density | 1990 | 0.05 |
| 1958 | Ningxia Hui Autonomous Region | *Spermophilus alaschanicus* | Grassland | Population density | 1991 | 0.14 |
| 1959 | Ningxia Hui Autonomous Region | *Spermophilus alaschanicus* | Grassland | Population density | 1992 | 0.20 |
| 1960 | Ningxia Hui Autonomous Region | *Spermophilus alaschanicus* | Grassland | Population density | 1993 | 0.17 |
| 1961 | Ningxia Hui Autonomous Region | *Spermophilus alaschanicus* | Grassland | Population density | 1994 | 0.19 |
| 1962 | Ningxia Hui Autonomous Region | *Spermophilus alaschanicus* | Grassland | Population density | 1995 | 0.27 |
| 1963 | Ningxia Hui Autonomous Region | *Spermophilus alaschanicus* | Grassland | Population density | 1996 | 0.34 |
| 1964 | Ningxia Hui Autonomous Region | *Spermophilus alaschanicus* | Grassland | Population density | 1997 | 0.38 |
| 1965 | Ningxia Hui Autonomous Region | *Spermophilus alaschanicus* | Grassland | Population density | 1998 | 0.41 |
| 1966 | Ningxia Hui Autonomous Region | *Spermophilus alaschanicus* | Grassland | Population density | 1999 | 0.53 |
| 1967 | Ningxia Hui Autonomous Region | *Spermophilus alaschanicus* | Grassland | Population density | 2000 | 0.81 |
| 1968 | Ningxia Hui Autonomous Region | *Spermophilus alaschanicus* | Grassland | Population density | 2001 | 0.90 |
| 1969 | Ningxia Hui Autonomous Region | *Spermophilus alaschanicus* | Grassland | Population density | 2002 | 1.00 |
| 1970 | Ningxia Hui Autonomous Region | *Spermophilus alaschanicus* | Grassland | Population density | 2003 | 0.98 |
| 1971 | Ningxia Hui Autonomous Region | *Spermophilus alaschanicus* | Grassland | Population density | 2004 | 0.87 |
| 1972 | Ningxia Hui Autonomous Region | *Spermophilus alaschanicus* | Grassland | Trap success | 1980 | 0.52 |
| 1973 | Ningxia Hui Autonomous Region | *Spermophilus alaschanicus* | Grassland | Trap success | 1981 | 0.72 |
| 1974 | Ningxia Hui Autonomous Region | *Spermophilus alaschanicus* | Grassland | Trap success | 1982 | 0.27 |
| 1975 | Ningxia Hui Autonomous Region | *Spermophilus alaschanicus* | Grassland | Trap success | 1983 | 0.00 |
| 1976 | Ningxia Hui Autonomous Region | *Spermophilus alaschanicus* | Grassland | Trap success | 1984 | 0.12 |
| 1977 | Ningxia Hui Autonomous Region | *Spermophilus alaschanicus* | Grassland | Trap success | 1985 | 0.06 |
| 1978 | Ningxia Hui Autonomous Region | *Spermophilus alaschanicus* | Grassland | Trap success | 1986 | 0.10 |
| 1979 | Ningxia Hui Autonomous Region | *Spermophilus alaschanicus* | Grassland | Trap success | 1987 | 0.11 |
| 1980 | Ningxia Hui Autonomous Region | *Spermophilus alaschanicus* | Grassland | Trap success | 1988 | 0.14 |
| 1981 | Ningxia Hui Autonomous Region | *Spermophilus alaschanicus* | Grassland | Trap success | 1989 | 0.26 |
| 1982 | Ningxia Hui Autonomous Region | *Spermophilus alaschanicus* | Grassland | Trap success | 1990 | 0.27 |
| 1983 | Ningxia Hui Autonomous Region | *Spermophilus alaschanicus* | Grassland | Trap success | 1991 | 0.53 |
| 1984 | Ningxia Hui Autonomous Region | *Spermophilus alaschanicus* | Grassland | Trap success | 1992 | 0.63 |
| 1985 | Ningxia Hui Autonomous Region | *Spermophilus alaschanicus* | Grassland | Trap success | 1993 | 0.74 |
| 1986 | Ningxia Hui Autonomous Region | *Spermophilus alaschanicus* | Grassland | Trap success | 1994 | 0.89 |
| 1987 | Ningxia Hui Autonomous Region | *Spermophilus alaschanicus* | Grassland | Trap success | 1995 | 0.96 |
| 1988 | Ningxia Hui Autonomous Region | *Spermophilus alaschanicus* | Grassland | Trap success | 1996 | 0.98 |
| 1989 | Ningxia Hui Autonomous Region | *Spermophilus alaschanicus* | Grassland | Trap success | 1997 | 0.99 |
| 1990 | Ningxia Hui Autonomous Region | *Spermophilus alaschanicus* | Grassland | Trap success | 1998 | 0.98 |
| 1991 | Ningxia Hui Autonomous Region | *Spermophilus alaschanicus* | Grassland | Trap success | 1999 | 1.00 |
| 1992 | Ningxia Hui Autonomous Region | *Spermophilus alaschanicus* | Grassland | Trap success | 2000 | 0.80 |
| 1993 | Ningxia Hui Autonomous Region | *Spermophilus alaschanicus* | Grassland | Trap success | 2001 | 0.78 |
| 1994 | Ningxia Hui Autonomous Region | *Spermophilus alaschanicus* | Grassland | Trap success | 2002 | 0.99 |
| 1995 | Ningxia Hui Autonomous Region | *Spermophilus alaschanicus* | Grassland | Trap success | 2003 | 0.80 |
| 1996 | Shandong Province | rodent | City | Trap success | 1984 | 0.59 |
| 1997 | Shandong Province | rodent | City | Trap success | 1985 | 0.60 |
| 1998 | Shandong Province | rodent | City | Trap success | 1986 | 0.91 |
| 1999 | Shandong Province | rodent | City | Trap success | 1987 | 0.27 |
| 2000 | Shandong Province | rodent | City | Trap success | 1988 | 0.14 |
| 2001 | Shandong Province | rodent | City | Trap success | 1989 | 0.03 |
| 2002 | Shandong Province | rodent | City | Trap success | 1990 | 0.00 |
| 2003 | Shandong Province | rodent | City | Trap success | 1991 | 0.07 |
| 2004 | Shandong Province | rodent | City | Trap success | 1992 | 0.14 |
| 2005 | Shandong Province | rodent | City | Trap success | 1993 | 0.08 |
| 2006 | Shandong Province | rodent | City | Trap success | 1994 | 0.12 |
| 2007 | Shandong Province | rodent | City | Trap success | 1995 | 0.31 |
| 2008 | Shandong Province | rodent | City | Trap success | 1996 | 0.48 |
| 2009 | Shandong Province | rodent | City | Trap success | 1997 | 0.40 |
| 2010 | Shandong Province | rodent | City | Trap success | 1998 | 0.68 |
| 2011 | Shandong Province | rodent | City | Trap success | 1999 | 0.77 |
| 2012 | Shandong Province | rodent | City | Trap success | 2000 | 0.79 |
| 2013 | Shandong Province | rodent | City | Trap success | 2001 | 0.80 |
| 2014 | Shandong Province | rodent | City | Trap success | 2002 | 0.82 |
| 2015 | Shandong Province | rodent | City | Trap success | 2003 | 1.00 |
| 2016 | Shandong Province | rodent | City | Trap success | 2004 | 0.91 |
| 2017 | Shandong Province | rodent | City | Trap success | 1982 | 0.54 |
| 2018 | Shandong Province | rodent | City | Trap success | 1983 | 0.37 |
| 2019 | Shandong Province | rodent | City | Trap success | 1984 | 0.48 |
| 2020 | Shandong Province | rodent | City | Trap success | 1985 | 0.51 |
| 2021 | Shandong Province | rodent | City | Trap success | 1986 | 0.47 |
| 2022 | Shandong Province | rodent | City | Trap success | 1987 | 0.27 |
| 2023 | Shandong Province | rodent | City | Trap success | 1988 | 0.36 |
| 2024 | Shandong Province | rodent | City | Trap success | 1989 | 0.51 |
| 2025 | Shandong Province | rodent | City | Trap success | 1990 | 0.44 |
| 2026 | Shandong Province | rodent | City | Trap success | 1991 | 0.45 |
| 2027 | Shandong Province | rodent | City | Trap success | 1992 | 0.64 |
| 2028 | Shandong Province | rodent | City | Trap success | 1993 | 0.96 |
| 2029 | Shandong Province | rodent | City | Trap success | 1994 | 1.00 |
| 2030 | Shandong Province | rodent | City | Trap success | 1995 | 0.73 |
| 2031 | Shandong Province | rodent | City | Trap success | 1996 | 0.58 |
| 2032 | Shandong Province | rodent | City | Trap success | 1997 | 0.60 |
| 2033 | Shandong Province | rodent | City | Trap success | 1998 | 0.58 |
| 2034 | Shandong Province | rodent | City | Trap success | 1999 | 0.16 |
| 2035 | Shandong Province | rodent | City | Trap success | 2000 | 0.20 |
| 2036 | Shandong Province | rodent | City | Trap success | 2001 | 0.13 |
| 2037 | Shandong Province | rodent | City | Trap success | 2002 | 0.07 |
| 2038 | Shandong Province | rodent | City | Trap success | 2003 | 0.03 |
| 2039 | Shandong Province | rodent | City | Trap success | 2004 | 0.14 |
| 2040 | Shandong Province | rodent | City | Trap success | 2005 | 0.00 |
| 2041 | Shandong Province | rodent | Farmland | Trap success | 1982 | 0.87 |
| 2042 | Shandong Province | rodent | Farmland | Trap success | 1983 | 1.00 |
| 2043 | Shandong Province | rodent | Farmland | Trap success | 1984 | 0.67 |
| 2044 | Shandong Province | rodent | Farmland | Trap success | 1985 | 0.54 |
| 2045 | Shandong Province | rodent | Farmland | Trap success | 1986 | 0.36 |
| 2046 | Shandong Province | rodent | Farmland | Trap success | 1987 | 0.48 |
| 2047 | Shandong Province | rodent | Farmland | Trap success | 1988 | 0.78 |
| 2048 | Shandong Province | rodent | Farmland | Trap success | 1989 | 0.65 |
| 2049 | Shandong Province | rodent | Farmland | Trap success | 1990 | 0.74 |
| 2050 | Shandong Province | rodent | Farmland | Trap success | 1991 | 0.75 |
| 2051 | Shandong Province | rodent | Farmland | Trap success | 1992 | 0.86 |
| 2052 | Shandong Province | rodent | Farmland | Trap success | 1993 | 0.82 |
| 2053 | Shandong Province | rodent | Farmland | Trap success | 1994 | 0.70 |
| 2054 | Shandong Province | rodent | Farmland | Trap success | 1995 | 0.57 |
| 2055 | Shandong Province | rodent | Farmland | Trap success | 1996 | 0.65 |
| 2056 | Shandong Province | rodent | Farmland | Trap success | 1997 | 0.44 |
| 2057 | Shandong Province | rodent | Farmland | Trap success | 1998 | 0.32 |
| 2058 | Shandong Province | rodent | Farmland | Trap success | 1999 | 0.15 |
| 2059 | Shandong Province | rodent | Farmland | Trap success | 2000 | 0.30 |
| 2060 | Shandong Province | rodent | Farmland | Trap success | 2001 | 0.14 |
| 2061 | Shandong Province | rodent | Farmland | Trap success | 2002 | 0.04 |
| 2062 | Shandong Province | rodent | Farmland | Trap success | 2003 | 0.11 |
| 2063 | Shandong Province | rodent | Farmland | Trap success | 2004 | 0.03 |
| 2064 | Shandong Province | rodent | Farmland | Trap success | 2005 | 0.00 |
| 2065 | Shandong Province | rodent | City | Trap success | 1983 | 0.79 |
| 2066 | Shandong Province | rodent | City | Trap success | 1984 | 0.91 |
| 2067 | Shandong Province | rodent | City | Trap success | 1985 | 1.00 |
| 2068 | Shandong Province | rodent | City | Trap success | 1986 | 0.99 |
| 2069 | Shandong Province | rodent | City | Trap success | 1987 | 0.53 |
| 2070 | Shandong Province | rodent | City | Trap success | 1988 | 0.58 |
| 2071 | Shandong Province | rodent | City | Trap success | 1989 | 0.23 |
| 2072 | Shandong Province | rodent | City | Trap success | 1990 | 0.14 |
| 2073 | Shandong Province | rodent | City | Trap success | 1991 | 0.19 |
| 2074 | Shandong Province | rodent | City | Trap success | 1992 | 0.27 |
| 2075 | Shandong Province | rodent | City | Trap success | 1993 | 0.15 |
| 2076 | Shandong Province | rodent | City | Trap success | 1994 | 0.24 |
| 2077 | Shandong Province | rodent | City | Trap success | 1995 | 0.39 |
| 2078 | Shandong Province | rodent | City | Trap success | 1996 | 0.22 |
| 2079 | Shandong Province | rodent | City | Trap success | 1997 | 0.25 |
| 2080 | Shandong Province | rodent | City | Trap success | 1998 | 0.33 |
| 2081 | Shandong Province | rodent | City | Trap success | 1999 | 0.29 |
| 2082 | Shandong Province | rodent | City | Trap success | 2000 | 0.16 |
| 2083 | Shandong Province | rodent | City | Trap success | 2001 | 0.11 |
| 2084 | Shandong Province | rodent | City | Trap success | 2002 | 0.07 |
| 2085 | Shandong Province | rodent | City | Trap success | 2003 | 0.00 |
| 2086 | Shandong Province | rodent | Farmland | Trap success | 1983 | 0.68 |
| 2087 | Shandong Province | rodent | Farmland | Trap success | 1984 | 0.89 |
| 2088 | Shandong Province | rodent | Farmland | Trap success | 1985 | 1.00 |
| 2089 | Shandong Province | rodent | Farmland | Trap success | 1986 | 0.64 |
| 2090 | Shandong Province | rodent | Farmland | Trap success | 1987 | 0.53 |
| 2091 | Shandong Province | rodent | Farmland | Trap success | 1988 | 0.38 |
| 2092 | Shandong Province | rodent | Farmland | Trap success | 1989 | 0.30 |
| 2093 | Shandong Province | rodent | Farmland | Trap success | 1990 | 0.12 |
| 2094 | Shandong Province | rodent | Farmland | Trap success | 1991 | 0.18 |
| 2095 | Shandong Province | rodent | Farmland | Trap success | 1992 | 0.17 |
| 2096 | Shandong Province | rodent | Farmland | Trap success | 1993 | 0.19 |
| 2097 | Shandong Province | rodent | Farmland | Trap success | 1994 | 0.22 |
| 2098 | Shandong Province | rodent | Farmland | Trap success | 1995 | 0.40 |
| 2099 | Shandong Province | rodent | Farmland | Trap success | 1996 | 0.22 |
| 2100 | Shandong Province | rodent | Farmland | Trap success | 1997 | 0.24 |
| 2101 | Shandong Province | rodent | Farmland | Trap success | 1998 | 0.20 |
| 2102 | Shandong Province | rodent | Farmland | Trap success | 1999 | 0.17 |
| 2103 | Shandong Province | rodent | Farmland | Trap success | 2000 | 0.11 |
| 2104 | Shandong Province | rodent | Farmland | Trap success | 2001 | 0.07 |
| 2105 | Shandong Province | rodent | Farmland | Trap success | 2002 | 0.05 |
| 2106 | Shandong Province | rodent | Farmland | Trap success | 2003 | 0.00 |
| 2107 | Shandong Province | rodent | City | Trap success | 1983 | 0.47 |
| 2108 | Shandong Province | rodent | City | Trap success | 1984 | 0.57 |
| 2109 | Shandong Province | rodent | City | Trap success | 1985 | 0.92 |
| 2110 | Shandong Province | rodent | City | Trap success | 1986 | 1.00 |
| 2111 | Shandong Province | rodent | City | Trap success | 1987 | 0.19 |
| 2112 | Shandong Province | rodent | City | Trap success | 1988 | 0.38 |
| 2113 | Shandong Province | rodent | City | Trap success | 1989 | 0.52 |
| 2114 | Shandong Province | rodent | City | Trap success | 1990 | 0.12 |
| 2115 | Shandong Province | rodent | City | Trap success | 1991 | 0.07 |
| 2116 | Shandong Province | rodent | City | Trap success | 1992 | 0.07 |
| 2117 | Shandong Province | rodent | City | Trap success | 1993 | 0.18 |
| 2118 | Shandong Province | rodent | City | Trap success | 1994 | 0.11 |
| 2119 | Shandong Province | rodent | City | Trap success | 1995 | 0.22 |
| 2120 | Shandong Province | rodent | City | Trap success | 1996 | 0.20 |
| 2121 | Shandong Province | rodent | City | Trap success | 1997 | 0.47 |
| 2122 | Shandong Province | rodent | City | Trap success | 1998 | 0.66 |
| 2123 | Shandong Province | rodent | City | Trap success | 1999 | 0.48 |
| 2124 | Shandong Province | rodent | City | Trap success | 2000 | 0.80 |
| 2125 | Shandong Province | rodent | City | Trap success | 2001 | 0.28 |
| 2126 | Shandong Province | rodent | City | Trap success | 2002 | 0.11 |
| 2127 | Shandong Province | rodent | City | Trap success | 2003 | 0.04 |
| 2128 | Shandong Province | rodent | City | Trap success | 2004 | 0.00 |
| 2129 | Shandong Province | rodent | Farmland | Trap success | 1983 | 0.03 |
| 2130 | Shandong Province | rodent | Farmland | Trap success | 1984 | 0.09 |
| 2131 | Shandong Province | rodent | Farmland | Trap success | 1985 | 0.24 |
| 2132 | Shandong Province | rodent | Farmland | Trap success | 1986 | 0.00 |
| 2133 | Shandong Province | rodent | Farmland | Trap success | 1987 | 0.28 |
| 2134 | Shandong Province | rodent | Farmland | Trap success | 1988 | 0.31 |
| 2135 | Shandong Province | rodent | Farmland | Trap success | 1989 | 0.27 |
| 2136 | Shandong Province | rodent | Farmland | Trap success | 1990 | 0.37 |
| 2137 | Shandong Province | rodent | Farmland | Trap success | 1991 | 0.25 |
| 2138 | Shandong Province | rodent | Farmland | Trap success | 1992 | 0.18 |
| 2139 | Shandong Province | rodent | Farmland | Trap success | 1993 | 0.21 |
| 2140 | Shandong Province | rodent | Farmland | Trap success | 1994 | 0.37 |
| 2141 | Shandong Province | rodent | Farmland | Trap success | 1995 | 0.40 |
| 2142 | Shandong Province | rodent | Farmland | Trap success | 1996 | 0.59 |
| 2143 | Shandong Province | rodent | Farmland | Trap success | 1997 | 0.45 |
| 2144 | Shandong Province | rodent | Farmland | Trap success | 1998 | 0.52 |
| 2145 | Shandong Province | rodent | Farmland | Trap success | 1999 | 0.68 |
| 2146 | Shandong Province | rodent | Farmland | Trap success | 2000 | 0.62 |
| 2147 | Shandong Province | rodent | Farmland | Trap success | 2001 | 0.53 |
| 2148 | Shandong Province | rodent | Farmland | Trap success | 2002 | 0.75 |
| 2149 | Shandong Province | rodent | Farmland | Trap success | 2003 | 0.92 |
| 2150 | Shandong Province | rodent | Farmland | Trap success | 2004 | 1.00 |
| 2151 | Shaanxi Province | *Meriones unguiculatus* | Grassland | Population density | 1983 | 0.01 |
| 2152 | Shaanxi Province | *Meriones unguiculatus* | Grassland | Population density | 1984 | 0.02 |
| 2153 | Shaanxi Province | *Meriones unguiculatus* | Grassland | Population density | 1985 | 0.68 |
| 2154 | Shaanxi Province | *Meriones unguiculatus* | Grassland | Population density | 1986 | 1.00 |
| 2155 | Shaanxi Province | *Meriones unguiculatus* | Grassland | Population density | 1987 | 0.22 |
| 2156 | Shaanxi Province | *Meriones unguiculatus* | Grassland | Population density | 1988 | 0.03 |
| 2157 | Shaanxi Province | *Meriones unguiculatus* | Grassland | Population density | 1989 | 0.01 |
| 2158 | Shaanxi Province | *Meriones unguiculatus* | Grassland | Population density | 1990 | 0.00 |
| 2159 | Shaanxi Province | *Meriones unguiculatus* | Grassland | Population density | 1991 | 0.01 |
| 2160 | Shaanxi Province | *Meriones unguiculatus* | Grassland | Population density | 1992 | 0.02 |
| 2161 | Shaanxi Province | *Meriones unguiculatus* | Grassland | Population density | 1993 | 0.03 |
| 2162 | Shaanxi Province | *Meriones unguiculatus* | Grassland | Population density | 1994 | 0.03 |
| 2163 | Shaanxi Province | *Meriones unguiculatus* | Grassland | Population density | 1995 | 0.07 |
| 2164 | Shaanxi Province | *Meriones unguiculatus* | Grassland | Population density | 1996 | 0.12 |
| 2165 | Shaanxi Province | *Meriones unguiculatus* | Grassland | Population density | 1997 | 0.13 |
| 2166 | Shaanxi Province | *Meriones unguiculatus* | Grassland | Population density | 1998 | 0.19 |
| 2167 | Shaanxi Province | *Meriones unguiculatus* | Grassland | Population density | 1999 | 0.29 |
| 2168 | Shaanxi Province | *Meriones unguiculatus* | Grassland | Population density | 2000 | 0.41 |
| 2169 | Shaanxi Province | *Meriones unguiculatus* | Grassland | Population density | 2001 | 0.13 |
| 2170 | Shaanxi Province | *Meriones unguiculatus* | Grassland | Population density | 2002 | 0.35 |
| 2171 | Shaanxi Province | *Meriones unguiculatus* | Grassland | Population density | 2003 | 0.53 |
| 2172 | Shaanxi Province | *Meriones unguiculatus* | Grassland | Population density | 2004 | 0.48 |
| 2173 | Shaanxi Province | *Meriones unguiculatus* | Grassland | Population density | 2005 | 0.39 |
| 2174 | Shaanxi Province | *Meriones unguiculatus* | Grassland | Population density | 2006 | 0.15 |
| 2175 | Shaanxi Province | *Meriones unguiculatus* | Grassland | Population density | 2007 | 0.18 |
| 2176 | Shaanxi Province | *Meriones unguiculatus* | Grassland | Population density | 2008 | 0.11 |
| 2177 | Shaanxi Province | *Meriones unguiculatus* | Grassland | Population density | 2009 | 0.17 |
| 2178 | Shaanxi Province | *Meriones unguiculatus* | Grassland | Population density | 2010 | 0.17 |
| 2179 | Shaanxi Province | rodent | City | Trap success | 1983 | 1.00 |
| 2180 | Shaanxi Province | rodent | City | Trap success | 1984 | 0.73 |
| 2181 | Shaanxi Province | rodent | City | Trap success | 1985 | 0.13 |
| 2182 | Shaanxi Province | rodent | City | Trap success | 1986 | 0.72 |
| 2183 | Shaanxi Province | rodent | City | Trap success | 1987 | 0.87 |
| 2184 | Shaanxi Province | rodent | City | Trap success | 1988 | 0.26 |
| 2185 | Shaanxi Province | rodent | City | Trap success | 1989 | 0.78 |
| 2186 | Shaanxi Province | rodent | City | Trap success | 1990 | 0.59 |
| 2187 | Shaanxi Province | rodent | City | Trap success | 1991 | 0.17 |
| 2188 | Shaanxi Province | rodent | City | Trap success | 1992 | 0.00 |
| 2189 | Shaanxi Province | rodent | City | Trap success | 1993 | 0.05 |
| 2190 | Shaanxi Province | rodent | City | Trap success | 1994 | 0.05 |
| 2191 | Shaanxi Province | rodent | City | Trap success | 1995 | 0.11 |
| 2192 | Shaanxi Province | rodent | City | Trap success | 1996 | 0.20 |
| 2193 | Shaanxi Province | rodent | City | Trap success | 1997 | 0.51 |
| 2194 | Shaanxi Province | rodent | City | Trap success | 1998 | 0.98 |
| 2195 | Shaanxi Province | rodent | City | Trap success | 1999 | 0.30 |
| 2196 | Shaanxi Province | rodent | City | Trap success | 2000 | 0.30 |
| 2197 | Shaanxi Province | rodent | City | Trap success | 2001 | 0.24 |
| 2198 | Shaanxi Province | rodent | City | Trap success | 2002 | 0.60 |
| 2199 | Shaanxi Province | rodent | City | Trap success | 2003 | 0.30 |
| 2200 | Shaanxi Province | rodent | City | Trap success | 2004 | 0.37 |
| 2201 | Shaanxi Province | rodent | City | Trap success | 2005 | 0.48 |
| 2202 | Shaanxi Province | rodent | Grassland – farmland mosaic | Trap success | 1983 | 0.01 |
| 2203 | Shaanxi Province | rodent | Grassland – farmland mosaic | Trap success | 1984 | 0.22 |
| 2204 | Shaanxi Province | rodent | Grassland – farmland mosaic | Trap success | 1985 | 0.12 |
| 2205 | Shaanxi Province | rodent | Grassland – farmland mosaic | Trap success | 1986 | 0.09 |
| 2206 | Shaanxi Province | rodent | Grassland – farmland mosaic | Trap success | 1987 | 0.09 |
| 2207 | Shaanxi Province | rodent | Grassland – farmland mosaic | Trap success | 1988 | 0.16 |
| 2208 | Shaanxi Province | rodent | Grassland – farmland mosaic | Trap success | 1989 | 0.08 |
| 2209 | Shaanxi Province | rodent | Grassland – farmland mosaic | Trap success | 1990 | 0.05 |
| 2210 | Shaanxi Province | rodent | Grassland – farmland mosaic | Trap success | 1991 | 0.02 |
| 2211 | Shaanxi Province | rodent | Grassland – farmland mosaic | Trap success | 1992 | 0.02 |
| 2212 | Shaanxi Province | rodent | Grassland – farmland mosaic | Trap success | 1993 | 0.08 |
| 2213 | Shaanxi Province | rodent | Grassland – farmland mosaic | Trap success | 1994 | 0.07 |
| 2214 | Shaanxi Province | rodent | Grassland – farmland mosaic | Trap success | 1995 | 0.19 |
| 2215 | Shaanxi Province | rodent | Grassland – farmland mosaic | Trap success | 1996 | 0.19 |
| 2216 | Shaanxi Province | rodent | Grassland – farmland mosaic | Trap success | 1997 | 0.43 |
| 2217 | Shaanxi Province | rodent | Grassland – farmland mosaic | Trap success | 1998 | 0.69 |
| 2218 | Shaanxi Province | rodent | Grassland – farmland mosaic | Trap success | 1999 | 0.40 |
| 2219 | Shaanxi Province | rodent | Grassland – farmland mosaic | Trap success | 2000 | 0.82 |
| 2220 | Shaanxi Province | rodent | Grassland – farmland mosaic | Trap success | 2001 | 0.43 |
| 2221 | Shaanxi Province | rodent | Grassland – farmland mosaic | Trap success | 2002 | 1.00 |
| 2222 | Shaanxi Province | rodent | Grassland – farmland mosaic | Trap success | 2003 | 0.66 |
| 2223 | Shaanxi Province | rodent | Grassland – farmland mosaic | Trap success | 2004 | 0.84 |
| 2224 | Shaanxi Province | rodent | Grassland – farmland mosaic | Trap success | 2005 | 0.33 |
| 2225 | Shaanxi Province | rodent | Grassland – farmland mosaic | Trap success | 2006 | 0.07 |
| 2226 | Shaanxi Province | rodent | Grassland – farmland mosaic | Trap success | 2007 | 0.06 |
| 2227 | Shaanxi Province | rodent | Grassland – farmland mosaic | Trap success | 2008 | 0.03 |
| 2228 | Shaanxi Province | rodent | Grassland – farmland mosaic | Trap success | 2009 | 0.00 |
| 2229 | Shaanxi Province | rodent | Grassland – farmland mosaic | Trap success | 2010 | 0.12 |
| 2230 | Shaanxi Province | rodent | Grassland – farmland mosaic | Trap success | 1983 | 0.01 |
| 2231 | Shaanxi Province | rodent | Grassland – farmland mosaic | Trap success | 1984 | 0.22 |
| 2232 | Shaanxi Province | rodent | Grassland – farmland mosaic | Trap success | 1985 | 0.13 |
| 2233 | Shaanxi Province | rodent | Grassland – farmland mosaic | Trap success | 1986 | 0.09 |
| 2234 | Shaanxi Province | rodent | Grassland – farmland mosaic | Trap success | 1987 | 0.10 |
| 2235 | Shaanxi Province | rodent | Grassland – farmland mosaic | Trap success | 1988 | 0.16 |
| 2236 | Shaanxi Province | rodent | Grassland – farmland mosaic | Trap success | 1989 | 0.08 |
| 2237 | Shaanxi Province | rodent | Grassland – farmland mosaic | Trap success | 1990 | 0.05 |
| 2238 | Shaanxi Province | rodent | Grassland – farmland mosaic | Trap success | 1991 | 0.03 |
| 2239 | Shaanxi Province | rodent | Grassland – farmland mosaic | Trap success | 1992 | 0.02 |
| 2240 | Shaanxi Province | rodent | Grassland – farmland mosaic | Trap success | 1993 | 0.08 |
| 2241 | Shaanxi Province | rodent | Grassland – farmland mosaic | Trap success | 1994 | 0.08 |
| 2242 | Shaanxi Province | rodent | Grassland – farmland mosaic | Trap success | 1995 | 0.19 |
| 2243 | Shaanxi Province | rodent | Grassland – farmland mosaic | Trap success | 1996 | 0.19 |
| 2244 | Shaanxi Province | rodent | Grassland – farmland mosaic | Trap success | 1997 | 0.44 |
| 2245 | Shaanxi Province | rodent | Grassland – farmland mosaic | Trap success | 1998 | 0.69 |
| 2246 | Shaanxi Province | rodent | Grassland – farmland mosaic | Trap success | 1999 | 0.39 |
| 2247 | Shaanxi Province | rodent | Grassland – farmland mosaic | Trap success | 2000 | 0.81 |
| 2248 | Shaanxi Province | rodent | Grassland – farmland mosaic | Trap success | 2001 | 0.43 |
| 2249 | Shaanxi Province | rodent | Grassland – farmland mosaic | Trap success | 2002 | 1.00 |
| 2250 | Shaanxi Province | rodent | Grassland – farmland mosaic | Trap success | 2003 | 0.66 |
| 2251 | Shaanxi Province | rodent | Grassland – farmland mosaic | Trap success | 2004 | 0.56 |
| 2252 | Shaanxi Province | rodent | Grassland – farmland mosaic | Trap success | 2005 | 0.33 |
| 2253 | Shaanxi Province | rodent | Grassland – farmland mosaic | Trap success | 2006 | 0.07 |
| 2254 | Shaanxi Province | rodent | Grassland – farmland mosaic | Trap success | 2007 | 0.06 |
| 2255 | Shaanxi Province | rodent | Grassland – farmland mosaic | Trap success | 2008 | 0.03 |
| 2256 | Shaanxi Province | rodent | Grassland – farmland mosaic | Trap success | 2009 | 0.00 |
| 2257 | Shaanxi Province | rodent | Grassland – farmland mosaic | Trap success | 2010 | 0.12 |
| 2258 | Shaanxi Province | rodent | Grassland – farmland mosaic | Trap success | 2011 | 0.10 |
| 2259 | Shaanxi Province | rodent | Grassland – farmland mosaic | Trap success | 2012 | 0.04 |
| 2260 | Shaanxi Province | rodent | Grassland – farmland mosaic | Population density | 1983 | 0.00 |
| 2261 | Shaanxi Province | rodent | Grassland – farmland mosaic | Population density | 1984 | 0.01 |
| 2262 | Shaanxi Province | rodent | Grassland – farmland mosaic | Population density | 1985 | 0.67 |
| 2263 | Shaanxi Province | rodent | Grassland – farmland mosaic | Population density | 1986 | 1.00 |
| 2264 | Shaanxi Province | rodent | Grassland – farmland mosaic | Population density | 1987 | 0.21 |
| 2265 | Shaanxi Province | rodent | Grassland – farmland mosaic | Population density | 1988 | 0.03 |
| 2266 | Shaanxi Province | rodent | Grassland – farmland mosaic | Population density | 1989 | 0.01 |
| 2267 | Shaanxi Province | rodent | Grassland – farmland mosaic | Population density | 1990 | 0.00 |
| 2268 | Shaanxi Province | rodent | Grassland – farmland mosaic | Population density | 1991 | 0.01 |
| 2269 | Shaanxi Province | rodent | Grassland – farmland mosaic | Population density | 1992 | 0.01 |
| 2270 | Shaanxi Province | rodent | Grassland – farmland mosaic | Population density | 1993 | 0.02 |
| 2271 | Shaanxi Province | rodent | Grassland – farmland mosaic | Population density | 1994 | 0.03 |
| 2272 | Shaanxi Province | rodent | Grassland – farmland mosaic | Population density | 1995 | 0.07 |
| 2273 | Shaanxi Province | rodent | Grassland – farmland mosaic | Population density | 1996 | 0.12 |
| 2274 | Shaanxi Province | rodent | Grassland – farmland mosaic | Population density | 1997 | 0.12 |
| 2275 | Shaanxi Province | rodent | Grassland – farmland mosaic | Population density | 1998 | 0.18 |
| 2276 | Shaanxi Province | rodent | Grassland – farmland mosaic | Population density | 1999 | 0.28 |
| 2277 | Shaanxi Province | rodent | Grassland – farmland mosaic | Population density | 2000 | 0.41 |
| 2278 | Shaanxi Province | rodent | Grassland – farmland mosaic | Population density | 2001 | 0.12 |
| 2279 | Shaanxi Province | rodent | Grassland – farmland mosaic | Population density | 2002 | 0.35 |
| 2280 | Shaanxi Province | rodent | Grassland – farmland mosaic | Population density | 2003 | 0.53 |
| 2281 | Shaanxi Province | rodent | Grassland – farmland mosaic | Population density | 2004 | 0.48 |
| 2282 | Shaanxi Province | rodent | Grassland – farmland mosaic | Population density | 2005 | 0.38 |
| 2283 | Shaanxi Province | rodent | Grassland – farmland mosaic | Population density | 2006 | 0.15 |
| 2284 | Shaanxi Province | rodent | Grassland – farmland mosaic | Population density | 2007 | 0.18 |
| 2285 | Shaanxi Province | rodent | Grassland – farmland mosaic | Population density | 2008 | 0.09 |
| 2286 | Shaanxi Province | rodent | Grassland – farmland mosaic | Population density | 2009 | 0.16 |
| 2287 | Shaanxi Province | rodent | Grassland – farmland mosaic | Population density | 2010 | 0.17 |
| 2288 | Shaanxi Province | rodent | Grassland – farmland mosaic | Population density | 2011 | 0.17 |
| 2289 | Shaanxi Province | rodent | Grassland – farmland mosaic | Population density | 2012 | 0.23 |
| 2290 | Shanghai | *Apodemus agrarius* | Farmland | Trap success | 1987 | 0.45 |
| 2291 | Shanghai | *Apodemus agrarius* | Farmland | Trap success | 1988 | 0.41 |
| 2292 | Shanghai | *Apodemus agrarius* | Farmland | Trap success | 1989 | 0.37 |
| 2293 | Shanghai | *Apodemus agrarius* | Farmland | Trap success | 1990 | 0.45 |
| 2294 | Shanghai | *Apodemus agrarius* | Farmland | Trap success | 1991 | 0.35 |
| 2295 | Shanghai | *Apodemus agrarius* | Farmland | Trap success | 1992 | 0.26 |
| 2296 | Shanghai | *Apodemus agrarius* | Farmland | Trap success | 1993 | 0.14 |
| 2297 | Shanghai | *Apodemus agrarius* | Farmland | Trap success | 1994 | 0.13 |
| 2298 | Shanghai | *Apodemus agrarius* | Farmland | Trap success | 1995 | 0.06 |
| 2299 | Shanghai | *Apodemus agrarius* | Farmland | Trap success | 1996 | 0.03 |
| 2300 | Shanghai | *Apodemus agrarius* | Farmland | Trap success | 1997 | 0.26 |
| 2301 | Shanghai | *Apodemus agrarius* | Farmland | Trap success | 1998 | 0.61 |
| 2302 | Shanghai | *Apodemus agrarius* | Farmland | Trap success | 1999 | 1.00 |
| 2303 | Shanghai | *Apodemus agrarius* | Farmland | Trap success | 2000 | 0.37 |
| 2304 | Shanghai | *Apodemus agrarius* | Farmland | Trap success | 2001 | 0.02 |
| 2305 | Shanghai | *Apodemus agrarius* | Farmland | Trap success | 2002 | 0.15 |
| 2306 | Shanghai | *Apodemus agrarius* | Farmland | Trap success | 2003 | 0.13 |
| 2307 | Shanghai | *Apodemus agrarius* | Farmland | Trap success | 2004 | 0.02 |
| 2308 | Shanghai | *Apodemus agrarius* | Farmland | Trap success | 2005 | 0.00 |
| 2309 | Shanghai | *Apodemus agrarius* | Farmland | Trap success | 2006 | 0.01 |
| 2310 | Shanghai | *Mus musculus* | Farmland | Trap success | 1987 | 0.00 |
| 2311 | Shanghai | *Mus musculus* | Farmland | Trap success | 1988 | 0.17 |
| 2312 | Shanghai | *Mus musculus* | Farmland | Trap success | 1989 | 1.00 |
| 2313 | Shanghai | *Mus musculus* | Farmland | Trap success | 1990 | 0.09 |
| 2314 | Shanghai | *Mus musculus* | Farmland | Trap success | 1991 | 0.96 |
| 2315 | Shanghai | *Mus musculus* | Farmland | Trap success | 1992 | 0.39 |
| 2316 | Shanghai | *Mus musculus* | Farmland | Trap success | 1993 | 0.00 |
| 2317 | Shanghai | *Mus musculus* | Farmland | Trap success | 1994 | 0.43 |
| 2318 | Shanghai | *Mus musculus* | Farmland | Trap success | 1995 | 0.83 |
| 2319 | Shanghai | *Mus musculus* | Farmland | Trap success | 1996 | 0.45 |
| 2320 | Shanghai | *Mus musculus* | Farmland | Trap success | 1997 | 0.36 |
| 2321 | Shanghai | *Mus musculus* | Farmland | Trap success | 1998 | 0.81 |
| 2322 | Shanghai | *Mus musculus* | Farmland | Trap success | 1999 | 0.63 |
| 2323 | Shanghai | *Mus musculus* | Farmland | Trap success | 2000 | 0.76 |
| 2324 | Shanghai | *Mus musculus* | Farmland | Trap success | 2001 | 0.00 |
| 2325 | Shanghai | *Mus musculus* | Farmland | Trap success | 2002 | 0.70 |
| 2326 | Shanghai | *Mus musculus* | Farmland | Trap success | 2003 | 0.59 |
| 2327 | Shanghai | *Mus musculus* | Farmland | Trap success | 2004 | 0.00 |
| 2328 | Shanghai | *Mus musculus* | Farmland | Trap success | 2005 | 0.00 |
| 2329 | Shanghai | *Mus musculus* | Farmland | Trap success | 2006 | 0.23 |
| 2330 | Shanghai | *Rattus norvegicus* | Farmland | Trap success | 1987 | 0.00 |
| 2331 | Shanghai | *Rattus norvegicus* | Farmland | Trap success | 1988 | 0.05 |
| 2332 | Shanghai | *Rattus norvegicus* | Farmland | Trap success | 1989 | 0.98 |
| 2333 | Shanghai | *Rattus norvegicus* | Farmland | Trap success | 1990 | 0.05 |
| 2334 | Shanghai | *Rattus norvegicus* | Farmland | Trap success | 1991 | 1.00 |
| 2335 | Shanghai | *Rattus norvegicus* | Farmland | Trap success | 1992 | 0.46 |
| 2336 | Shanghai | *Rattus norvegicus* | Farmland | Trap success | 1993 | 0.35 |
| 2337 | Shanghai | *Rattus norvegicus* | Farmland | Trap success | 1994 | 0.26 |
| 2338 | Shanghai | *Rattus norvegicus* | Farmland | Trap success | 1995 | 0.18 |
| 2339 | Shanghai | *Rattus norvegicus* | Farmland | Trap success | 1996 | 0.07 |
| 2340 | Shanghai | *Rattus norvegicus* | Farmland | Trap success | 1997 | 0.22 |
| 2341 | Shanghai | *Rattus norvegicus* | Farmland | Trap success | 1998 | 0.69 |
| 2342 | Shanghai | *Rattus norvegicus* | Farmland | Trap success | 1999 | 0.51 |
| 2343 | Shanghai | *Rattus norvegicus* | Farmland | Trap success | 2000 | 0.00 |
| 2344 | Shanghai | *Rattus norvegicus* | Farmland | Trap success | 2001 | 0.00 |
| 2345 | Shanghai | *Rattus norvegicus* | Farmland | Trap success | 2002 | 0.00 |
| 2346 | Shanghai | *Rattus norvegicus* | Farmland | Trap success | 2003 | 0.00 |
| 2347 | Shanghai | *Rattus norvegicus* | Farmland | Trap success | 2004 | 0.00 |
| 2348 | Shanghai | *Rattus norvegicus* | Farmland | Trap success | 2005 | 0.04 |
| 2349 | Shanghai | *Rattus norvegicus* | Farmland | Trap success | 2006 | 0.00 |
| 2350 | Shanghai | rodent | Farmland | Trap success | 1988 | 1.00 |
| 2351 | Shanghai | rodent | Farmland | Trap success | 1989 | 0.80 |
| 2352 | Shanghai | rodent | Farmland | Trap success | 1990 | 0.79 |
| 2353 | Shanghai | rodent | Farmland | Trap success | 1991 | 0.52 |
| 2354 | Shanghai | rodent | Farmland | Trap success | 1992 | 0.33 |
| 2355 | Shanghai | rodent | Farmland | Trap success | 1993 | 0.69 |
| 2356 | Shanghai | rodent | Farmland | Trap success | 1994 | 0.45 |
| 2357 | Shanghai | rodent | Farmland | Trap success | 1995 | 0.96 |
| 2358 | Shanghai | rodent | Farmland | Trap success | 1996 | 0.66 |
| 2359 | Shanghai | rodent | Farmland | Trap success | 1997 | 0.48 |
| 2360 | Shanghai | rodent | Farmland | Trap success | 1998 | 0.41 |
| 2361 | Shanghai | rodent | Farmland | Trap success | 1999 | 0.49 |
| 2362 | Shanghai | rodent | Farmland | Trap success | 2000 | 0.18 |
| 2363 | Shanghai | rodent | Farmland | Trap success | 2001 | 0.25 |
| 2364 | Shanghai | rodent | Farmland | Trap success | 2002 | 0.21 |
| 2365 | Shanghai | rodent | Farmland | Trap success | 2003 | 0.13 |
| 2366 | Shanghai | rodent | Farmland | Trap success | 2004 | 0.24 |
| 2367 | Shanghai | rodent | Farmland | Trap success | 2005 | 0.10 |
| 2368 | Shanghai | rodent | Farmland | Trap success | 2006 | 0.13 |
| 2369 | Shanghai | rodent | Farmland | Trap success | 2007 | 0.14 |
| 2370 | Shanghai | rodent | Farmland | Trap success | 2008 | 0.08 |
| 2371 | Shanghai | rodent | Farmland | Trap success | 2009 | 0.07 |
| 2372 | Shanghai | rodent | Farmland | Trap success | 2010 | 0.01 |
| 2373 | Shanghai | rodent | Farmland | Trap success | 2011 | 0.00 |
| 2374 | Shanghai | rodent | Farmland | Trap success | 2012 | 0.10 |
| 2375 | Shanghai | rodent | Farmland | Trap success | 2013 | 0.15 |
| 2376 | Sichuan Province | *Apodemus agrarius* | Farmland | Trap success | 1980 | 0.36 |
| 2377 | Sichuan Province | *Apodemus agrarius* | Farmland | Trap success | 1981 | 0.23 |
| 2378 | Sichuan Province | *Apodemus agrarius* | Farmland | Trap success | 1982 | 0.36 |
| 2379 | Sichuan Province | *Apodemus agrarius* | Farmland | Trap success | 1983 | 0.29 |
| 2380 | Sichuan Province | *Apodemus agrarius* | Farmland | Trap success | 1984 | 0.23 |
| 2381 | Sichuan Province | *Apodemus agrarius* | Farmland | Trap success | 1985 | 0.18 |
| 2382 | Sichuan Province | *Apodemus agrarius* | Farmland | Trap success | 1986 | 0.27 |
| 2383 | Sichuan Province | *Apodemus agrarius* | Farmland | Trap success | 1987 | 0.08 |
| 2384 | Sichuan Province | *Apodemus agrarius* | Farmland | Trap success | 1988 | 0.14 |
| 2385 | Sichuan Province | *Apodemus agrarius* | Farmland | Trap success | 1989 | 0.12 |
| 2386 | Sichuan Province | *Apodemus agrarius* | Farmland | Trap success | 1990 | 0.76 |
| 2387 | Sichuan Province | *Apodemus agrarius* | Farmland | Trap success | 1991 | 0.23 |
| 2388 | Sichuan Province | *Apodemus agrarius* | Farmland | Trap success | 1992 | 0.76 |
| 2389 | Sichuan Province | *Apodemus agrarius* | Farmland | Trap success | 1993 | 1.00 |
| 2390 | Sichuan Province | *Apodemus agrarius* | Farmland | Trap success | 1994 | 0.05 |
| 2391 | Sichuan Province | *Apodemus agrarius* | Farmland | Trap success | 1995 | 0.02 |
| 2392 | Sichuan Province | *Apodemus agrarius* | Farmland | Trap success | 1996 | 0.08 |
| 2393 | Sichuan Province | *Apodemus agrarius* | Farmland | Trap success | 1997 | 0.45 |
| 2394 | Sichuan Province | *Apodemus agrarius* | Farmland | Trap success | 1998 | 0.48 |
| 2395 | Sichuan Province | *Apodemus agrarius* | Farmland | Trap success | 1999 | 0.35 |
| 2396 | Sichuan Province | *Apodemus agrarius* | Farmland | Trap success | 2000 | 0.21 |
| 2397 | Sichuan Province | *Apodemus agrarius* | Farmland | Trap success | 2001 | 0.21 |
| 2398 | Sichuan Province | *Apodemus agrarius* | Farmland | Trap success | 2002 | 0.01 |
| 2399 | Sichuan Province | *Apodemus agrarius* | Farmland | Trap success | 2003 | 0.15 |
| 2400 | Sichuan Province | *Apodemus agrarius* | Farmland | Trap success | 2004 | 0.23 |
| 2401 | Sichuan Province | *Apodemus agrarius* | Farmland | Trap success | 2005 | 0.00 |
| 2402 | Sichuan Province | *Apodemus agrarius* | Farmland | Trap success | 2006 | 0.25 |
| 2403 | Sichuan Province | *Apodemus agrarius* | Farmland | Trap success | 2007 | 0.14 |
| 2404 | Sichuan Province | *Mus musculus* | City | Trap success | 1985 | 0.57 |
| 2405 | Sichuan Province | *Mus musculus* | City | Trap success | 1986 | 0.93 |
| 2406 | Sichuan Province | *Mus musculus* | City | Trap success | 1987 | 0.66 |
| 2407 | Sichuan Province | *Mus musculus* | City | Trap success | 1988 | 0.61 |
| 2408 | Sichuan Province | *Mus musculus* | City | Trap success | 1989 | 0.90 |
| 2409 | Sichuan Province | *Mus musculus* | City | Trap success | 1990 | 1.00 |
| 2410 | Sichuan Province | *Mus musculus* | City | Trap success | 1992 | 0.94 |
| 2411 | Sichuan Province | *Mus musculus* | City | Trap success | 1993 | 0.00 |
| 2412 | Sichuan Province | *Mus musculus* | City | Trap success | 1994 | 0.30 |
| 2413 | Sichuan Province | *Mus musculus* | City | Trap success | 1995 | 0.00 |
| 2414 | Sichuan Province | *Mus musculus* | City | Trap success | 1996 | 0.00 |
| 2415 | Sichuan Province | *Mus musculus* | City | Trap success | 1997 | 0.00 |
| 2416 | Sichuan Province | *Mus musculus* | City | Trap success | 1998 | 0.00 |
| 2417 | Sichuan Province | *Mus musculus* | City | Trap success | 1999 | 0.00 |
| 2418 | Sichuan Province | *Mus musculus* | City | Trap success | 2000 | 0.52 |
| 2419 | Sichuan Province | *Mus musculus* | City | Trap success | 2001 | 0.16 |
| 2420 | Sichuan Province | *Mus musculus* | City | Trap success | 2002 | 0.00 |
| 2421 | Sichuan Province | *Mus musculus* | City | Trap success | 2003 | 0.00 |
| 2422 | Sichuan Province | *Mus musculus* | City | Trap success | 2004 | 0.48 |
| 2423 | Sichuan Province | *Mus musculus* | City | Trap success | 2005 | 0.37 |
| 2424 | Sichuan Province | *Mus musculus* | City | Trap success | 2006 | 0.00 |
| 2425 | Sichuan Province | *Mus musculus* | City | Trap success | 2007 | 0.25 |
| 2426 | Sichuan Province | *Rattus norvegicus* | City | Trap success | 1985 | 0.79 |
| 2427 | Sichuan Province | *Rattus norvegicus* | City | Trap success | 1986 | 0.97 |
| 2428 | Sichuan Province | *Rattus norvegicus* | City | Trap success | 1987 | 0.26 |
| 2429 | Sichuan Province | *Rattus norvegicus* | City | Trap success | 1988 | 0.37 |
| 2430 | Sichuan Province | *Rattus norvegicus* | City | Trap success | 1989 | 0.57 |
| 2431 | Sichuan Province | *Rattus norvegicus* | City | Trap success | 1990 | 0.00 |
| 2432 | Sichuan Province | *Rattus norvegicus* | City | Trap success | 1992 | 0.43 |
| 2433 | Sichuan Province | *Rattus norvegicus* | City | Trap success | 1993 | 1.00 |
| 2434 | Sichuan Province | *Rattus norvegicus* | City | Trap success | 1994 | 0.54 |
| 2435 | Sichuan Province | *Rattus norvegicus* | City | Trap success | 1995 | 0.86 |
| 2436 | Sichuan Province | *Rattus norvegicus* | City | Trap success | 1996 | 0.39 |
| 2437 | Sichuan Province | *Rattus norvegicus* | City | Trap success | 1997 | 0.41 |
| 2438 | Sichuan Province | *Rattus norvegicus* | City | Trap success | 1998 | 0.79 |
| 2439 | Sichuan Province | *Rattus norvegicus* | City | Trap success | 1999 | 0.46 |
| 2440 | Sichuan Province | *Rattus norvegicus* | City | Trap success | 2000 | 0.44 |
| 2441 | Sichuan Province | *Rattus norvegicus* | City | Trap success | 2001 | 0.99 |
| 2442 | Sichuan Province | *Rattus norvegicus* | City | Trap success | 2002 | 0.37 |
| 2443 | Sichuan Province | *Rattus norvegicus* | City | Trap success | 2003 | 0.17 |
| 2444 | Sichuan Province | *Rattus norvegicus* | City | Trap success | 2004 | 0.61 |
| 2445 | Sichuan Province | *Rattus norvegicus* | City | Trap success | 2005 | 0.59 |
| 2446 | Sichuan Province | *Rattus norvegicus* | City | Trap success | 2006 | 0.50 |
| 2447 | Sichuan Province | *Rattus norvegicus* | City | Trap success | 2007 | 0.77 |
| 2448 | Sichuan Province | *Rattus norvegicus* | Farmland | Trap success | 1980 | 0.35 |
| 2449 | Sichuan Province | *Rattus norvegicus* | Farmland | Trap success | 1981 | 0.18 |
| 2450 | Sichuan Province | *Rattus norvegicus* | Farmland | Trap success | 1982 | 0.49 |
| 2451 | Sichuan Province | *Rattus norvegicus* | Farmland | Trap success | 1983 | 0.28 |
| 2452 | Sichuan Province | *Rattus norvegicus* | Farmland | Trap success | 1984 | 0.23 |
| 2453 | Sichuan Province | *Rattus norvegicus* | Farmland | Trap success | 1985 | 0.26 |
| 2454 | Sichuan Province | *Rattus norvegicus* | Farmland | Trap success | 1986 | 0.14 |
| 2455 | Sichuan Province | *Rattus norvegicus* | Farmland | Trap success | 1987 | 0.07 |
| 2456 | Sichuan Province | *Rattus norvegicus* | Farmland | Trap success | 1988 | 0.20 |
| 2457 | Sichuan Province | *Rattus norvegicus* | Farmland | Trap success | 1989 | 1.00 |
| 2458 | Sichuan Province | *Rattus norvegicus* | Farmland | Trap success | 1990 | 0.28 |
| 2459 | Sichuan Province | *Rattus norvegicus* | Farmland | Trap success | 1991 | 0.04 |
| 2460 | Sichuan Province | *Rattus norvegicus* | Farmland | Trap success | 1992 | 0.21 |
| 2461 | Sichuan Province | *Rattus norvegicus* | Farmland | Trap success | 1993 | 0.59 |
| 2462 | Sichuan Province | *Rattus norvegicus* | Farmland | Trap success | 1994 | 0.15 |
| 2463 | Sichuan Province | *Rattus norvegicus* | Farmland | Trap success | 1995 | 0.27 |
| 2464 | Sichuan Province | *Rattus norvegicus* | Farmland | Trap success | 1996 | 0.16 |
| 2465 | Sichuan Province | *Rattus norvegicus* | Farmland | Trap success | 1997 | 0.00 |
| 2466 | Sichuan Province | *Rattus norvegicus* | Farmland | Trap success | 1998 | 0.24 |
| 2467 | Sichuan Province | *Rattus norvegicus* | Farmland | Trap success | 1999 | 0.01 |
| 2468 | Sichuan Province | *Rattus norvegicus* | Farmland | Trap success | 2000 | 0.05 |
| 2469 | Sichuan Province | *Rattus norvegicus* | Farmland | Trap success | 2001 | 0.01 |
| 2470 | Sichuan Province | *Rattus norvegicus* | Farmland | Trap success | 2002 | 0.01 |
| 2471 | Sichuan Province | *Rattus norvegicus* | Farmland | Trap success | 2003 | 0.05 |
| 2472 | Sichuan Province | *Rattus norvegicus* | Farmland | Trap success | 2004 | 0.01 |
| 2473 | Sichuan Province | *Rattus norvegicus* | Farmland | Trap success | 2005 | 0.02 |
| 2474 | Sichuan Province | *Rattus norvegicus* | Farmland | Trap success | 2006 | 0.00 |
| 2475 | Sichuan Province | *Rattus norvegicus* | Farmland | Trap success | 2007 | 0.01 |
| 2476 | Sichuan Province | *Apodemus agrarius* | Farmland | Trap success | 1980 | 0.34 |
| 2477 | Sichuan Province | *Apodemus agrarius* | Farmland | Trap success | 1981 | 0.22 |
| 2478 | Sichuan Province | *Apodemus agrarius* | Farmland | Trap success | 1982 | 0.34 |
| 2479 | Sichuan Province | *Apodemus agrarius* | Farmland | Trap success | 1983 | 0.28 |
| 2480 | Sichuan Province | *Apodemus agrarius* | Farmland | Trap success | 1984 | 0.21 |
| 2481 | Sichuan Province | *Apodemus agrarius* | Farmland | Trap success | 1985 | 0.16 |
| 2482 | Sichuan Province | *Apodemus agrarius* | Farmland | Trap success | 1986 | 0.25 |
| 2483 | Sichuan Province | *Apodemus agrarius* | Farmland | Trap success | 1987 | 0.05 |
| 2484 | Sichuan Province | *Apodemus agrarius* | Farmland | Trap success | 1988 | 0.12 |
| 2485 | Sichuan Province | *Apodemus agrarius* | Farmland | Trap success | 1989 | 0.10 |
| 2486 | Sichuan Province | *Apodemus agrarius* | Farmland | Trap success | 1990 | 0.76 |
| 2487 | Sichuan Province | *Apodemus agrarius* | Farmland | Trap success | 1991 | 0.22 |
| 2488 | Sichuan Province | *Apodemus agrarius* | Farmland | Trap success | 1992 | 0.75 |
| 2489 | Sichuan Province | *Apodemus agrarius* | Farmland | Trap success | 1993 | 1.00 |
| 2490 | Sichuan Province | *Apodemus agrarius* | Farmland | Trap success | 1994 | 0.03 |
| 2491 | Sichuan Province | *Apodemus agrarius* | Farmland | Trap success | 1995 | 0.00 |
| 2492 | Sichuan Province | *Apodemus agrarius* | Farmland | Trap success | 1996 | 0.22 |
| 2493 | Sichuan Province | *Apodemus agrarius* | Farmland | Trap success | 1997 | 0.55 |
| 2494 | Sichuan Province | *Apodemus agrarius* | Farmland | Trap success | 1998 | 0.47 |
| 2495 | Sichuan Province | *Apodemus agrarius* | Farmland | Trap success | 1999 | 0.34 |
| 2496 | Sichuan Province | *Rattus norvegicus* | Farmland | Trap success | 1980 | 0.35 |
| 2497 | Sichuan Province | *Rattus norvegicus* | Farmland | Trap success | 1981 | 0.18 |
| 2498 | Sichuan Province | *Rattus norvegicus* | Farmland | Trap success | 1982 | 0.49 |
| 2499 | Sichuan Province | *Rattus norvegicus* | Farmland | Trap success | 1983 | 0.28 |
| 2500 | Sichuan Province | *Rattus norvegicus* | Farmland | Trap success | 1984 | 0.23 |
| 2501 | Sichuan Province | *Rattus norvegicus* | Farmland | Trap success | 1985 | 0.26 |
| 2502 | Sichuan Province | *Rattus norvegicus* | Farmland | Trap success | 1986 | 0.13 |
| 2503 | Sichuan Province | *Rattus norvegicus* | Farmland | Trap success | 1987 | 0.07 |
| 2504 | Sichuan Province | *Rattus norvegicus* | Farmland | Trap success | 1988 | 0.20 |
| 2505 | Sichuan Province | *Rattus norvegicus* | Farmland | Trap success | 1989 | 1.00 |
| 2506 | Sichuan Province | *Rattus norvegicus* | Farmland | Trap success | 1990 | 0.28 |
| 2507 | Sichuan Province | *Rattus norvegicus* | Farmland | Trap success | 1991 | 0.04 |
| 2508 | Sichuan Province | *Rattus norvegicus* | Farmland | Trap success | 1992 | 0.21 |
| 2509 | Sichuan Province | *Rattus norvegicus* | Farmland | Trap success | 1993 | 0.59 |
| 2510 | Sichuan Province | *Rattus norvegicus* | Farmland | Trap success | 1994 | 0.15 |
| 2511 | Sichuan Province | *Rattus norvegicus* | Farmland | Trap success | 1995 | 0.27 |
| 2512 | Sichuan Province | *Rattus norvegicus* | Farmland | Trap success | 1996 | 0.37 |
| 2513 | Sichuan Province | *Rattus norvegicus* | Farmland | Trap success | 1997 | 0.00 |
| 2514 | Sichuan Province | *Rattus norvegicus* | Farmland | Trap success | 1998 | 0.24 |
| 2515 | Sichuan Province | *Rattus norvegicus* | Farmland | Trap success | 1999 | 0.01 |
| 2516 | Sichuan Province | *Apodemus agrarius* | Farmland | Trap success | 1985 | 0.70 |
| 2517 | Sichuan Province | *Apodemus agrarius* | Farmland | Trap success | 1986 | 0.63 |
| 2518 | Sichuan Province | *Apodemus agrarius* | Farmland | Trap success | 1987 | 0.25 |
| 2519 | Sichuan Province | *Apodemus agrarius* | Farmland | Trap success | 1988 | 0.48 |
| 2520 | Sichuan Province | *Apodemus agrarius* | Farmland | Trap success | 1989 | 0.38 |
| 2521 | Sichuan Province | *Apodemus agrarius* | Farmland | Trap success | 1990 | 0.83 |
| 2522 | Sichuan Province | *Apodemus agrarius* | Farmland | Trap success | 1991 | 1.00 |
| 2523 | Sichuan Province | *Apodemus agrarius* | Farmland | Trap success | 1992 | 0.74 |
| 2524 | Sichuan Province | *Apodemus agrarius* | Farmland | Trap success | 1993 | 0.62 |
| 2525 | Sichuan Province | *Apodemus agrarius* | Farmland | Trap success | 1994 | 0.46 |
| 2526 | Sichuan Province | *Apodemus agrarius* | Farmland | Trap success | 1995 | 0.47 |
| 2527 | Sichuan Province | *Apodemus agrarius* | Farmland | Trap success | 1996 | 0.34 |
| 2528 | Sichuan Province | *Apodemus agrarius* | Farmland | Trap success | 1997 | 1.00 |
| 2529 | Sichuan Province | *Apodemus agrarius* | Farmland | Trap success | 1998 | 0.25 |
| 2530 | Sichuan Province | *Apodemus agrarius* | Farmland | Trap success | 1999 | 0.05 |
| 2531 | Sichuan Province | *Apodemus agrarius* | Farmland | Trap success | 2000 | 0.14 |
| 2532 | Sichuan Province | *Apodemus agrarius* | Farmland | Trap success | 2001 | 0.53 |
| 2533 | Sichuan Province | *Apodemus agrarius* | Farmland | Trap success | 2002 | 0.31 |
| 2534 | Sichuan Province | *Apodemus agrarius* | Farmland | Trap success | 2003 | 0.05 |
| 2535 | Sichuan Province | *Apodemus agrarius* | Farmland | Trap success | 2004 | 0.01 |
| 2536 | Sichuan Province | *Apodemus agrarius* | Farmland | Trap success | 2005 | 0.02 |
| 2537 | Sichuan Province | *Apodemus agrarius* | Farmland | Trap success | 2006 | 0.04 |
| 2538 | Sichuan Province | *Apodemus agrarius* | Farmland | Trap success | 2007 | 0.05 |
| 2539 | Sichuan Province | *Apodemus agrarius* | Farmland | Trap success | 2008 | 0.05 |
| 2540 | Sichuan Province | *Apodemus agrarius* | Farmland | Trap success | 2009 | 0.00 |
| 2541 | Sichuan Province | *Apodemus agrarius* | Farmland | Trap success | 2010 | 0.00 |
| 2542 | Sichuan Province | *Rattus norvegicus* | City | Trap success | 1985 | 0.75 |
| 2543 | Sichuan Province | *Rattus norvegicus* | City | Trap success | 1986 | 0.74 |
| 2544 | Sichuan Province | *Rattus norvegicus* | City | Trap success | 1987 | 0.17 |
| 2545 | Sichuan Province | *Rattus norvegicus* | City | Trap success | 1988 | 0.35 |
| 2546 | Sichuan Province | *Rattus norvegicus* | City | Trap success | 1989 | 0.62 |
| 2547 | Sichuan Province | *Rattus norvegicus* | City | Trap success | 1990 | 0.45 |
| 2548 | Sichuan Province | *Rattus norvegicus* | City | Trap success | 1991 | 0.83 |
| 2549 | Sichuan Province | *Rattus norvegicus* | City | Trap success | 1992 | 1.00 |
| 2550 | Sichuan Province | *Rattus norvegicus* | City | Trap success | 1993 | 0.63 |
| 2551 | Sichuan Province | *Rattus norvegicus* | City | Trap success | 1994 | 0.66 |
| 2552 | Sichuan Province | *Rattus norvegicus* | City | Trap success | 1995 | 0.87 |
| 2553 | Sichuan Province | *Rattus norvegicus* | City | Trap success | 1996 | 0.43 |
| 2554 | Sichuan Province | *Rattus norvegicus* | City | Trap success | 1997 | 0.44 |
| 2555 | Sichuan Province | *Rattus norvegicus* | City | Trap success | 1998 | 0.38 |
| 2556 | Sichuan Province | *Rattus norvegicus* | City | Trap success | 1999 | 0.19 |
| 2557 | Sichuan Province | *Rattus norvegicus* | City | Trap success | 2000 | 0.20 |
| 2558 | Sichuan Province | *Rattus norvegicus* | City | Trap success | 2001 | 0.57 |
| 2559 | Sichuan Province | *Rattus norvegicus* | City | Trap success | 2002 | 0.14 |
| 2560 | Sichuan Province | *Rattus norvegicus* | City | Trap success | 2003 | 0.07 |
| 2561 | Sichuan Province | *Rattus norvegicus* | City | Trap success | 2004 | 0.00 |
| 2562 | Sichuan Province | *Rattus norvegicus* | City | Trap success | 2005 | 0.02 |
| 2563 | Sichuan Province | *Rattus norvegicus* | City | Trap success | 2006 | 0.02 |
| 2564 | Sichuan Province | *Rattus norvegicus* | City | Trap success | 2007 | 0.15 |
| 2565 | Sichuan Province | *Rattus norvegicus* | City | Trap success | 2008 | 0.16 |
| 2566 | Sichuan Province | *Rattus norvegicus* | City | Trap success | 2009 | 0.11 |
| 2567 | Sichuan Province | *Rattus norvegicus* | City | Trap success | 2010 | 0.32 |
| 2568 | Sichuan Province | *Apodemus agrarius* | City | Relative population density | 1980 | 1.00 |
| 2569 | Sichuan Province | *Apodemus agrarius* | City | Relative population density | 1981 | 0.73 |
| 2570 | Sichuan Province | *Apodemus agrarius* | City | Relative population density | 1982 | 0.80 |
| 2571 | Sichuan Province | *Apodemus agrarius* | City | Relative population density | 1983 | 0.87 |
| 2572 | Sichuan Province | *Apodemus agrarius* | City | Relative population density | 1984 | 0.84 |
| 2573 | Sichuan Province | *Apodemus agrarius* | City | Relative population density | 1985 | 0.80 |
| 2574 | Sichuan Province | *Apodemus agrarius* | City | Relative population density | 1986 | 0.34 |
| 2575 | Sichuan Province | *Apodemus agrarius* | City | Relative population density | 1987 | 0.39 |
| 2576 | Sichuan Province | *Apodemus agrarius* | City | Relative population density | 1988 | 0.41 |
| 2577 | Sichuan Province | *Apodemus agrarius* | City | Relative population density | 1989 | 0.39 |
| 2578 | Sichuan Province | *Apodemus agrarius* | City | Relative population density | 1990 | 0.46 |
| 2579 | Sichuan Province | *Apodemus agrarius* | City | Relative population density | 1991 | 0.47 |
| 2580 | Sichuan Province | *Apodemus agrarius* | City | Relative population density | 1992 | 0.51 |
| 2581 | Sichuan Province | *Apodemus agrarius* | City | Relative population density | 1993 | 0.47 |
| 2582 | Sichuan Province | *Apodemus agrarius* | City | Relative population density | 1994 | 0.46 |
| 2583 | Sichuan Province | *Apodemus agrarius* | City | Relative population density | 1995 | 0.47 |
| 2584 | Sichuan Province | *Apodemus agrarius* | City | Relative population density | 1996 | 0.56 |
| 2585 | Sichuan Province | *Apodemus agrarius* | City | Relative population density | 1997 | 0.57 |
| 2586 | Sichuan Province | *Apodemus agrarius* | City | Relative population density | 1998 | 0.13 |
| 2587 | Sichuan Province | *Apodemus agrarius* | City | Relative population density | 1999 | 0.19 |
| 2588 | Sichuan Province | *Apodemus agrarius* | City | Relative population density | 2000 | 0.24 |
| 2589 | Sichuan Province | *Apodemus agrarius* | City | Relative population density | 2001 | 0.27 |
| 2590 | Sichuan Province | *Apodemus agrarius* | City | Relative population density | 2002 | 0.21 |
| 2591 | Sichuan Province | *Apodemus agrarius* | City | Relative population density | 2003 | 0.03 |
| 2592 | Sichuan Province | *Apodemus agrarius* | City | Relative population density | 2004 | 0.00 |
| 2593 | Sichuan Province | *Rattus nitidus* | City | Relative population density | 1980 | 0.00 |
| 2594 | Sichuan Province | *Rattus nitidus* | City | Relative population density | 1981 | 0.84 |
| 2595 | Sichuan Province | *Rattus nitidus* | City | Relative population density | 1982 | 0.95 |
| 2596 | Sichuan Province | *Rattus nitidus* | City | Relative population density | 1983 | 0.89 |
| 2597 | Sichuan Province | *Rattus nitidus* | City | Relative population density | 1984 | 0.68 |
| 2598 | Sichuan Province | *Rattus nitidus* | City | Relative population density | 1985 | 1.00 |
| 2599 | Sichuan Province | *Rattus nitidus* | City | Relative population density | 1986 | 0.21 |
| 2600 | Sichuan Province | *Rattus nitidus* | City | Relative population density | 1987 | 0.26 |
| 2601 | Sichuan Province | *Rattus nitidus* | City | Relative population density | 1988 | 0.21 |
| 2602 | Sichuan Province | *Rattus nitidus* | City | Relative population density | 1989 | 0.16 |
| 2603 | Sichuan Province | *Rattus nitidus* | City | Relative population density | 1990 | 0.21 |
| 2604 | Sichuan Province | *Rattus nitidus* | City | Relative population density | 1991 | 0.42 |
| 2605 | Sichuan Province | *Rattus nitidus* | City | Relative population density | 1992 | 0.42 |
| 2606 | Sichuan Province | *Rattus nitidus* | City | Relative population density | 1993 | 0.37 |
| 2607 | Sichuan Province | *Rattus nitidus* | City | Relative population density | 1994 | 0.47 |
| 2608 | Sichuan Province | *Rattus nitidus* | City | Relative population density | 1995 | 0.37 |
| 2609 | Sichuan Province | *Rattus nitidus* | City | Relative population density | 1996 | 0.16 |
| 2610 | Sichuan Province | *Rattus nitidus* | City | Relative population density | 1997 | 0.11 |
| 2611 | Sichuan Province | *Rattus nitidus* | City | Relative population density | 1998 | 0.11 |
| 2612 | Sichuan Province | *Rattus nitidus* | City | Relative population density | 1999 | 0.26 |
| 2613 | Sichuan Province | *Rattus nitidus* | City | Relative population density | 2000 | 0.16 |
| 2614 | Sichuan Province | *Rattus nitidus* | City | Relative population density | 2001 | 0.16 |
| 2615 | Sichuan Province | *Rattus nitidus* | City | Relative population density | 2002 | 0.26 |
| 2616 | Sichuan Province | *Rattus nitidus* | City | Relative population density | 2003 | 0.16 |
| 2617 | Sichuan Province | *Rattus nitidus* | City | Relative population density | 2004 | 0.16 |
| 2618 | Sichuan Province | *Rattus norvegicus* | City | Relative population density | 1980 | 0.05 |
| 2619 | Sichuan Province | *Rattus norvegicus* | City | Relative population density | 1981 | 0.67 |
| 2620 | Sichuan Province | *Rattus norvegicus* | City | Relative population density | 1982 | 0.75 |
| 2621 | Sichuan Province | *Rattus norvegicus* | City | Relative population density | 1983 | 0.76 |
| 2622 | Sichuan Province | *Rattus norvegicus* | City | Relative population density | 1984 | 0.56 |
| 2623 | Sichuan Province | *Rattus norvegicus* | City | Relative population density | 1985 | 0.67 |
| 2624 | Sichuan Province | *Rattus norvegicus* | City | Relative population density | 1986 | 0.45 |
| 2625 | Sichuan Province | *Rattus norvegicus* | City | Relative population density | 1987 | 0.56 |
| 2626 | Sichuan Province | *Rattus norvegicus* | City | Relative population density | 1988 | 0.51 |
| 2627 | Sichuan Province | *Rattus norvegicus* | City | Relative population density | 1989 | 0.42 |
| 2628 | Sichuan Province | *Rattus norvegicus* | City | Relative population density | 1990 | 0.45 |
| 2629 | Sichuan Province | *Rattus norvegicus* | City | Relative population density | 1991 | 0.93 |
| 2630 | Sichuan Province | *Rattus norvegicus* | City | Relative population density | 1992 | 0.96 |
| 2631 | Sichuan Province | *Rattus norvegicus* | City | Relative population density | 1993 | 1.00 |
| 2632 | Sichuan Province | *Rattus norvegicus* | City | Relative population density | 1994 | 0.96 |
| 2633 | Sichuan Province | *Rattus norvegicus* | City | Relative population density | 1995 | 0.98 |
| 2634 | Sichuan Province | *Rattus norvegicus* | City | Relative population density | 1996 | 0.27 |
| 2635 | Sichuan Province | *Rattus norvegicus* | City | Relative population density | 1997 | 0.25 |
| 2636 | Sichuan Province | *Rattus norvegicus* | City | Relative population density | 1998 | 0.24 |
| 2637 | Sichuan Province | *Rattus norvegicus* | City | Relative population density | 1999 | 0.31 |
| 2638 | Sichuan Province | *Rattus norvegicus* | City | Relative population density | 2000 | 0.25 |
| 2639 | Sichuan Province | *Rattus norvegicus* | City | Relative population density | 2001 | 0.25 |
| 2640 | Sichuan Province | *Rattus norvegicus* | City | Relative population density | 2002 | 0.24 |
| 2641 | Sichuan Province | *Rattus norvegicus* | City | Relative population density | 2003 | 0.04 |
| 2642 | Sichuan Province | *Rattus norvegicus* | City | Relative population density | 2004 | 0.00 |
| 2643 | Xinjiang Uygur Autonomous Region | *Rhombomys opimus* | Grassland | Population density | 1980 | 0.93 |
| 2644 | Xinjiang Uygur Autonomous Region | *Rhombomys opimus* | Grassland | Population density | 1984 | 0.22 |
| 2645 | Xinjiang Uygur Autonomous Region | *Rhombomys opimus* | Grassland | Population density | 1985 | 0.15 |
| 2646 | Xinjiang Uygur Autonomous Region | *Rhombomys opimus* | Grassland | Population density | 1986 | 0.06 |
| 2647 | Xinjiang Uygur Autonomous Region | *Rhombomys opimus* | Grassland | Population density | 1987 | 0.04 |
| 2648 | Xinjiang Uygur Autonomous Region | *Rhombomys opimus* | Grassland | Population density | 1988 | 0.05 |
| 2649 | Xinjiang Uygur Autonomous Region | *Rhombomys opimus* | Grassland | Population density | 1989 | 0.04 |
| 2650 | Xinjiang Uygur Autonomous Region | *Rhombomys opimus* | Grassland | Population density | 1990 | 0.00 |
| 2651 | Xinjiang Uygur Autonomous Region | *Rhombomys opimus* | Grassland | Population density | 1991 | 0.00 |
| 2652 | Xinjiang Uygur Autonomous Region | *Rhombomys opimus* | Grassland | Population density | 1992 | 1.00 |
| 2653 | Xinjiang Uygur Autonomous Region | *Rhombomys opimus* | Grassland | Population density | 1993 | 0.07 |
| 2654 | Xinjiang Uygur Autonomous Region | *Rhombomys opimus* | Grassland | Population density | 1994 | 0.16 |
| 2655 | Xinjiang Uygur Autonomous Region | *Rhombomys opimus* | Grassland | Population density | 1995 | 0.18 |
| 2656 | Xinjiang Uygur Autonomous Region | *Rhombomys opimus* | Grassland | Population density | 1996 | 0.23 |
| 2657 | Xinjiang Uygur Autonomous Region | *Rhombomys opimus* | Grassland | Population density | 1997 | 0.20 |
| 2658 | Xinjiang Uygur Autonomous Region | *Rhombomys opimus* | Grassland | Population density | 1998 | 0.14 |
| 2659 | Xinjiang Uygur Autonomous Region | *Rhombomys opimus* | Grassland | Population density | 1999 | 0.20 |
| 2660 | Xinjiang Uygur Autonomous Region | *Rhombomys opimus* | Grassland | Population density | 2000 | 0.35 |
| 2661 | Xinjiang Uygur Autonomous Region | *Rhombomys opimus* | Grassland | Population density | 2001 | 0.98 |
| 2662 | Xinjiang Uygur Autonomous Region | *Rhombomys opimus* | Grassland | Population density | 2002 | 0.56 |
| 2663 | Xinjiang Uygur Autonomous Region | *Rhombomys opimus* | Grassland | Population density | 2003 | 0.40 |
| 2664 | Xinjiang Uygur Autonomous Region | *Rhombomys opimus* | Grassland | Population density | 2004 | 0.22 |
| 2665 | Xinjiang Uygur Autonomous Region | *Rhombomys opimus* | Grassland | Population density | 2005 | 0.11 |
| 2666 | Xinjiang Uygur Autonomous Region | *Rhombomys opimus* | Grassland | Population density | 2006 | 0.06 |
| 2667 | Xinjiang Uygur Autonomous Region | *Rhombomys opimus* | Grassland | Population density | 2007 | 0.09 |
| 2668 | Xinjiang Uygur Autonomous Region | *Rhombomys opimus* | Grassland | Population density | 2008 | 0.05 |
| 2669 | Xinjiang Uygur Autonomous Region | *Rhombomys opimus* | Grassland | Population density | 2009 | 0.06 |
| 2670 | Yunnan Province | *Rattus tanezumi* | City | Trap success | 1982 | 0.64 |
| 2671 | Yunnan Province | *Rattus tanezumi* | City | Trap success | 1983 | 1.00 |
| 2672 | Yunnan Province | *Rattus tanezumi* | City | Trap success | 1984 | 0.96 |
| 2673 | Yunnan Province | *Rattus tanezumi* | City | Trap success | 1985 | 0.49 |
| 2674 | Yunnan Province | *Rattus tanezumi* | City | Trap success | 1986 | 0.14 |
| 2675 | Yunnan Province | *Rattus tanezumi* | City | Trap success | 1987 | 0.08 |
| 2676 | Yunnan Province | *Rattus tanezumi* | City | Trap success | 1988 | 0.10 |
| 2677 | Yunnan Province | *Rattus tanezumi* | City | Trap success | 1989 | 0.18 |
| 2678 | Yunnan Province | *Rattus tanezumi* | City | Trap success | 1990 | 0.04 |
| 2679 | Yunnan Province | *Rattus tanezumi* | City | Trap success | 1991 | 0.05 |
| 2680 | Yunnan Province | *Rattus tanezumi* | City | Trap success | 1992 | 0.02 |
| 2681 | Yunnan Province | *Rattus tanezumi* | City | Trap success | 1993 | 0.06 |
| 2682 | Yunnan Province | *Rattus tanezumi* | City | Trap success | 1994 | 0.24 |
| 2683 | Yunnan Province | *Rattus tanezumi* | City | Trap success | 1995 | 0.34 |
| 2684 | Yunnan Province | *Rattus tanezumi* | City | Trap success | 1996 | 0.10 |
| 2685 | Yunnan Province | *Rattus tanezumi* | City | Trap success | 1997 | 0.10 |
| 2686 | Yunnan Province | *Rattus tanezumi* | City | Trap success | 1998 | 0.12 |
| 2687 | Yunnan Province | *Rattus tanezumi* | City | Trap success | 1999 | 0.26 |
| 2688 | Yunnan Province | *Rattus tanezumi* | City | Trap success | 2000 | 0.21 |
| 2689 | Yunnan Province | *Rattus tanezumi* | City | Trap success | 2001 | 0.23 |
| 2690 | Yunnan Province | *Rattus tanezumi* | City | Trap success | 2002 | 0.26 |
| 2691 | Yunnan Province | *Rattus tanezumi* | City | Trap success | 2003 | 0.22 |
| 2692 | Yunnan Province | *Rattus tanezumi* | City | Trap success | 2004 | 0.14 |
| 2693 | Yunnan Province | *Rattus tanezumi* | City | Trap success | 2005 | 0.08 |
| 2694 | Yunnan Province | *Rattus tanezumi* | City | Trap success | 2006 | 0.00 |
| 2695 | Yunnan Province | *Rattus tanezumi* | City | Trap success | 2007 | 0.10 |
| 2696 | Yunnan Province | *Rattus tanezumi* | City | Trap success | 2008 | 0.30 |
| 2697 | Yunnan Province | *Rattus tanezumi* | City | Trap success | 2009 | 0.29 |
| 2698 | Zhejiang Province | rodent | Farmland | Trap success | 1985 | 0.30 |
| 2699 | Zhejiang Province | rodent | Farmland | Trap success | 1986 | 0.34 |
| 2700 | Zhejiang Province | rodent | Farmland | Trap success | 1987 | 0.50 |
| 2701 | Zhejiang Province | rodent | Farmland | Trap success | 1988 | 0.70 |
| 2702 | Zhejiang Province | rodent | Farmland | Trap success | 1989 | 0.41 |
| 2703 | Zhejiang Province | rodent | Farmland | Trap success | 1990 | 1.00 |
| 2704 | Zhejiang Province | rodent | Farmland | Trap success | 1991 | 0.70 |
| 2705 | Zhejiang Province | rodent | Farmland | Trap success | 1992 | 0.65 |
| 2706 | Zhejiang Province | rodent | Farmland | Trap success | 1993 | 0.61 |
| 2707 | Zhejiang Province | rodent | Farmland | Trap success | 1994 | 0.56 |
| 2708 | Zhejiang Province | rodent | Farmland | Trap success | 1995 | 0.24 |
| 2709 | Zhejiang Province | rodent | Farmland | Trap success | 1996 | 0.18 |
| 2710 | Zhejiang Province | rodent | Farmland | Trap success | 1997 | 0.31 |
| 2711 | Zhejiang Province | rodent | Farmland | Trap success | 1998 | 0.16 |
| 2712 | Zhejiang Province | rodent | Farmland | Trap success | 1999 | 0.01 |
| 2713 | Zhejiang Province | rodent | Farmland | Trap success | 2000 | 0.03 |
| 2714 | Zhejiang Province | rodent | Farmland | Trap success | 2001 | 0.04 |
| 2715 | Zhejiang Province | rodent | Farmland | Trap success | 2002 | 0.02 |
| 2716 | Zhejiang Province | rodent | Farmland | Trap success | 2003 | 0.01 |
| 2717 | Zhejiang Province | rodent | Farmland | Trap success | 2004 | 0.00 |
| 2718 | Zhejiang Province | rodent | City | Trap success | 1982 | 0.97 |
| 2719 | Zhejiang Province | rodent | City | Trap success | 1983 | 0.89 |
| 2720 | Zhejiang Province | rodent | City | Trap success | 1984 | 0.00 |
| 2721 | Zhejiang Province | rodent | City | Trap success | 1985 | 0.00 |
| 2722 | Zhejiang Province | rodent | City | Trap success | 1986 | 0.24 |
| 2723 | Zhejiang Province | rodent | City | Trap success | 1987 | 0.65 |
| 2724 | Zhejiang Province | rodent | City | Trap success | 1988 | 0.55 |
| 2725 | Zhejiang Province | rodent | City | Trap success | 1989 | 0.57 |
| 2726 | Zhejiang Province | rodent | City | Trap success | 1990 | 0.65 |
| 2727 | Zhejiang Province | rodent | City | Trap success | 1991 | 0.52 |
| 2728 | Zhejiang Province | rodent | City | Trap success | 1992 | 0.48 |
| 2729 | Zhejiang Province | rodent | City | Trap success | 1993 | 0.45 |
| 2730 | Zhejiang Province | rodent | City | Trap success | 1994 | 0.45 |
| 2731 | Zhejiang Province | rodent | City | Trap success | 1995 | 0.53 |
| 2732 | Zhejiang Province | rodent | City | Trap success | 1996 | 0.72 |
| 2733 | Zhejiang Province | rodent | City | Trap success | 1997 | 1.00 |
| 2734 | Zhejiang Province | rodent | City | Trap success | 1998 | 0.76 |
| 2735 | Zhejiang Province | rodent | City | Trap success | 1999 | 0.36 |
| 2736 | Zhejiang Province | rodent | City | Trap success | 2000 | 0.53 |
| 2737 | Zhejiang Province | rodent | City | Trap success | 2001 | 0.79 |
